# Systems pharmacology-based exploration reveals mechanisms of anti-steatotic effects of Jiang Zhi Granule on non-alcoholic fatty liver disease

**DOI:** 10.1038/s41598-018-31708-8

**Published:** 2018-09-12

**Authors:** Yiyuan Zheng, Miao Wang, Peiyong Zheng, Xudong Tang, Guang Ji

**Affiliations:** 1 Institute of Digestive Disease, Longhua Hospital, Shanghai University of Traditional Chinese Medicine, Shanghai, 200032 China; 2 Xiyuan Hospital of China Academy of Chinese Medical Sciences, Beijing, 100091 China

## Abstract

Non-alcoholic fatty liver disease (NAFLD), which is in parallel with the obesity epidemic, accounts for a large amount of all chronic liver disease. Jiang Zhi Granule (JZG), a clinically used herbal formula, is developed in accordance with traditional Chinese medicine (TCM) pathogenesis for treating patients with NAFLD. In previous studies, the anti-steatotic effects of JZG against NAFLD have been demonstrated, and in this study, a systems pharmacology approach was used to explore the pharmacological mechanisms of JZG by predicting the active compounds within the herbal formula and their corresponding therapeutic targets. Its therapeutic efficacy was confirmed in the beginning of this study, and JZG was shown to significantly improve hepatic dysfunction and lipid droplet accumulation in PA-treated hepatocytes. Systems pharmacology was then performed to identify the active compounds in as well as to predict the therapeutic targets of this Chinese herbal prescription. Enrichment analyses indicated that the mechanisms of the anti-steatotic effects of JZG against NAFLD might be associated with lipid droplet degradation *via* autophagy, and a series of *in vitro* and *in vivo* validation experiments was subsequently performed to confirm that JZG could activate autophagy though the mTOR signalling to improve NAFLD.

## Introduction

Non-alcoholic fatty liver disease (NAFLD), which accounts for responsible for a large amount of liver-related complications^1^, has been increasingly recognized as the most prevalent chronic liver disease^2^; approximately one-fourth of the worldwide adult population currently suffers from NAFLD^3,4^. The spectrum of NAFLD extends broadly and encompasses simple steatosis non-alcoholic fatty liver (NAFL) or non-alcoholic steatohepatitis (NASH), steatofibrosis, and occasional progression to cirrhosis and hepatocellular carcinoma^1^. Epidemiological investigations have shown that NAFLD is one of the three main causes of cirrhosis^2,5^, and NASH, which is the aggressive form of NAFLD, is rapidly becoming the leading cause of end-stage liver disease^6,7^.

Despite this major public health concern, the pathogenesis of NAFLD is still not fully understood. One suggested mechanism can be explained by the “two-hit” hypothesis in which steatosis resulting from a disturbance in energy metabolic balance is the core event for NAFLD (first hit)^8,9^. This event is then followed by oxidative stress and hepatocellular injury, which are considered related to the typical pathological features of liver dysfunction, including steatosis, inflammation, fibrosis, and cirrhosis (second hit)^10,11^. Furthermore, apart from lifestyle interventions, such as dietary caloric restriction and exercise (which are currently the cornerstone of therapy for NAFLD), there is still no approved pharmacotherapy for this disease, as no large study has shown any efficacy of pharmacological treatments^12,13^. Thus, it is imperative to explore and develop new pharmacotherapies for NAFLD.

As an alternative and complementary medicine, traditional Chinese medicine (TCM) has attracted a great deal of attention in recent years and somehow has provided effective additions to current standardized intervention strategies. Jiang Zhi Granule (JZG), a clinically used herbal formula developed in accordance with TCM pathogenesis, is composed of the following five medicinal herbs: *Herba Gynostemmatis* (HG)*, Folium Nelumbinis* (FN)*, Radix Salviae* (RS)*, Rhizoma Polygoni Cuspidati* (RPC) and *Herba Artemisiae Scopariae* (HAS)^14^; furthermore, there is clinical evidence regarding the safety and efficacy of this formula^15^. Our previous studies have demonstrated that JZG could significantly improve hepatic lipid accumulation both *in vitro* and *in vivo*^16^. However, the cellular and molecular pharmacological mechanisms of JZG are still unknown and deserve further investigation.

Generally, the complex compounds contained in most Chinese herbal medicines will bring about multi-targeted effects, which cannot be accurately detected by conventional methods. Therefore, a systems pharmacology approach, a novel strategy that has been recently used to clarify the synergistic effects as well as the mechanisms of multi-component and multi-targeted agents^17,18^, was performed in this study to predict the active compounds and therapeutic targets based on screening a range of candidate compounds, predicting multiple drug targets and conducting a network analysis^19,20^. In addition, a series of comprehensive analyses and experiments were performed to further elucidate the underlying mechanisms of the anti-steatotic effects of JZG against NAFLD.

## Results

### Pharmacodynamics identification

**Figure 1:**
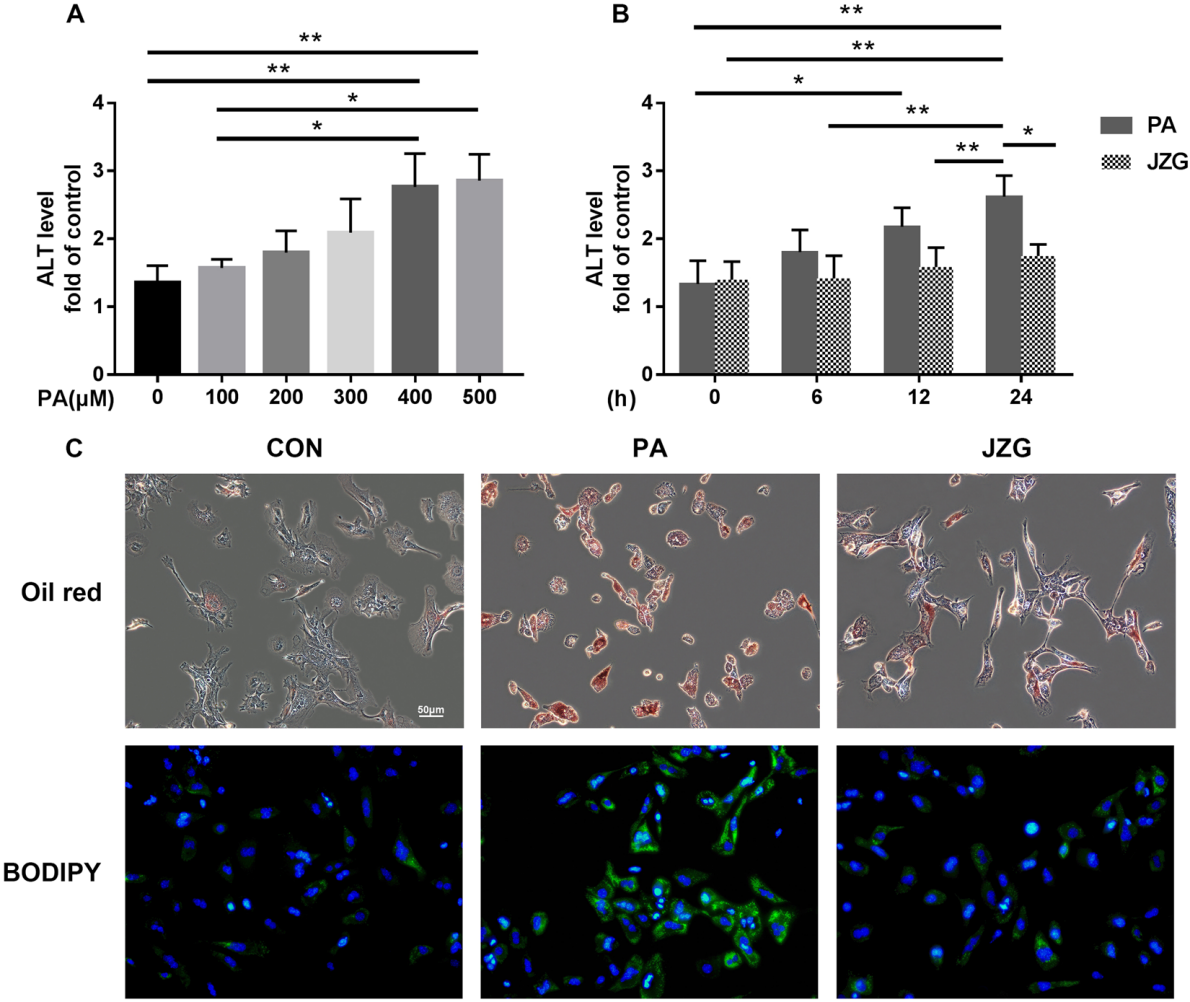
Pharmacodynamics investigation. The levels of ALT in the cell supernatants. (**A**) Hepatocytes were treated with various concentrations of PA for 24 h; (**B**) Hepatocytes were treated with 0.4 mM PA in the presence or absence of JZG (100 μg/mL) for different time periods; (**C**) Hepatocytes were treated with 0.4 mM PA in the presence or absence of JZG (100 μg/mL) for 24 h, and fluorescence staining with Oil Red O and BODIPY was performed to detect lipid droplets. ^*^P < 0.05, ^**^P < 0.01, ^***^P < 0.001.

To verify the anti-steatotic effects of JZG on the development of NAFLD, primary mouse hepatocytes harvested from wild-type C57BL/6 mice were used in this study. Hepatocytes were incubated with various concentrations of palmitic acid (PA) as well as in the presence or absence of JZG. Biochemical analyses confirmed the findings of our previous study^21^, in which the levels of alanine aminotransferase (ALT) in the cell supernatants were increased in a dose- and time-dependent manner, and JZG could mitigate the elevated ALT levels (Fig. 1A,B). These data show the cell injury induced by PA and the therapeutic effect of JZG on improving hepatic function. As the results showed no significant difference between PA doses of 0.4 and 0.5 mM and that the therapeutic effect was first evident at the 24-h time point, all subsequent experiments used hepatocytes treated with 0.4 mM PA in the presence or absence of JZG for 24 h.

Fluorescence staining with Oil Red O and BODIPY was performed as previously described to determine the cellular lipid accumulation, and the results showed that the accumulation of lipid droplets in hepatocytes was significantly increased in cells stimulated with PA and that this accumulation was obviously ameliorated by JZG (Fig. 1C), indicating either the inhibition of lipid droplet accumulation or the induction of lipid droplet degradation.

### Pharmacological mechanisms prediction

#### Candidate compound screening and target prediction for JZG

Since the efficacy of JZG has been successfully confirmed, systems pharmacology was performed next to identify the active compounds in JZG as well as to predict the therapeutic targets of these medicinal herbs. Oral bioavailability (OB), which represents the percentage of an orally administered dose of unchanged drug that reaches the systemic circulation and is often a key indicator to determine the drug-like properties of bioactive molecules as therapeutic agents^22,23^, was used as the screening index in this study to explore the active substance. In total, 58 potential compounds with an OB ≥ 30% were harvested from the herbal constituents of JZG. Additionally, 38 compounds with a lower OB value were also collected because of their extensive pharmacological activities, which were typical components of herbal drugs. Thus, 96 compounds from the five herbs were considered candidate compounds for further analyses; the numbers of potentially active compounds in HAS, RS, FN, RPC and HG were 21, 37, 25, 21 and 15, respectively (Supplementary Table S1). Among these, 23 compounds overlapped among the five herbs, which means that different herbal drugs contained in JZG shared similar ingredients with synergistic effects.

**Figure 2:**
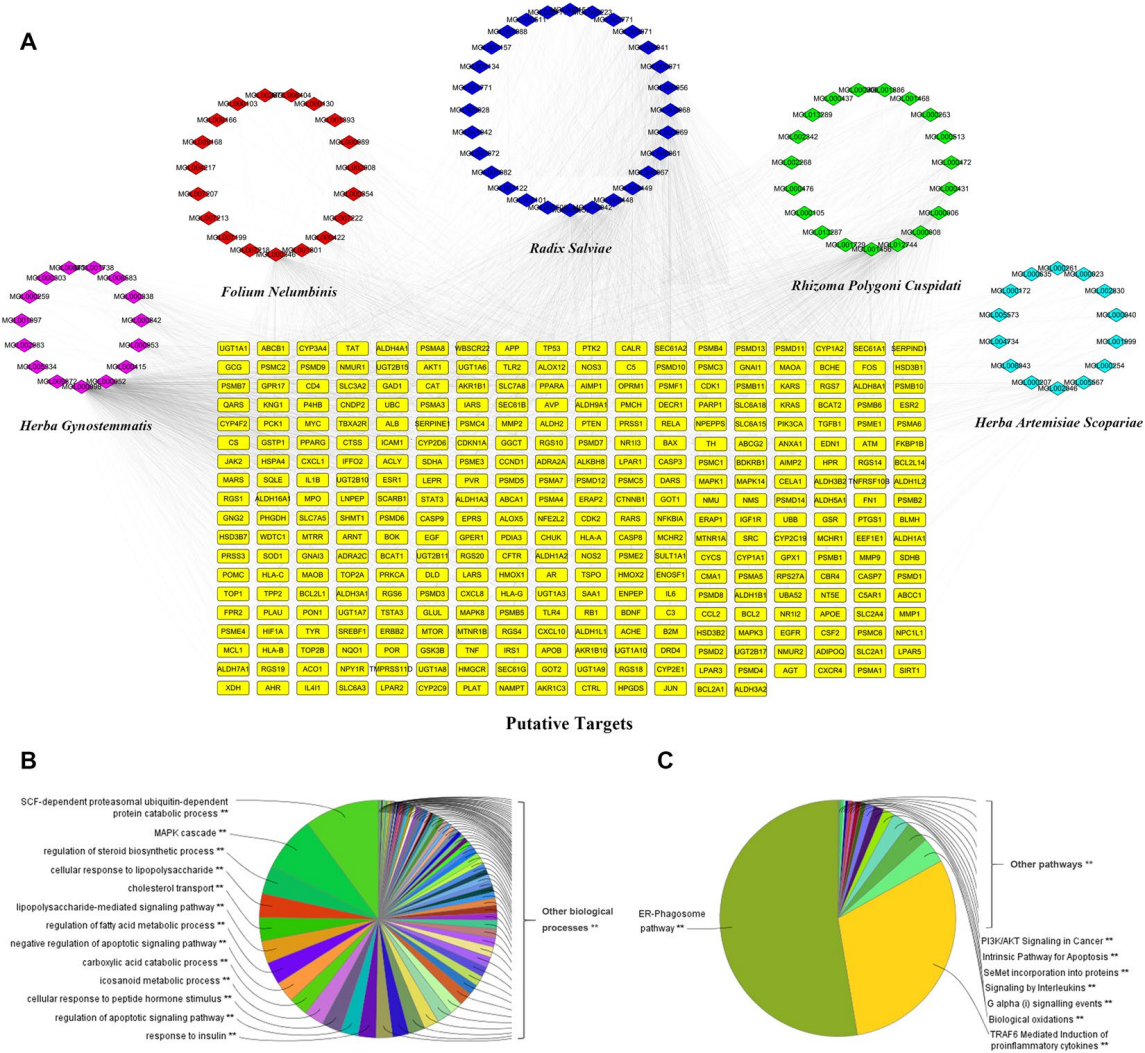
Candidate compound screening and target prediction for JZG. (**A**) A compound-target network was constructed to comprehend the complex interactions between all the candidate compounds in JZG and their corresponding targets; (**B**) Enrichment analysis was performed by ClueGO to investigate the biological processes of JZG; (**C**) Enrichment analysis was performed by ClueGO to investigate the signalling pathways of JZG.

As the effectiveness of TCM formulas depends on the synergistic effects between multiple compounds and their targets^24^, exploration of the therapeutic targets was also performed in this study by a comprehensive approach that integrated the available chemical, genomic and pharmacological information of the predicted targets. The results showed that 338 potential targets were predicted for the 96 candidate compounds, and the numbers of putative targets in HAS, RS, FN, RPC and HG were 192, 314, 253, 255 and 324, respectively (Supplementary Tables S2 and S3), suggesting that different compounds in JZG could regulate similar targets to exert synergistic effects. To better understand the complex interactions among all 96 compounds and their corresponding targets, a compound-target network, which contained 434 nodes and 3715 compound-target interactions (Fig. 2A), was constructed. The median value of degree centrality (DC), an index representative of the number of targets associated with it and is computed by the plug-in CytoNCA^25^, was 10. There were 223 candidate compounds that achieved this DC, indicating that most compounds influenced multiple targets to exert various effects.

To further clarify the possible mechanisms of the 96 candidate targets, ClueGO, a widely used Cytoscape plugin, was applied in this study to investigate the biological processes and signalling pathways^26^. Specifically, the biological processes were mainly related to stem cell factor (SCF)-dependent proteasomal ubiquitin-dependent protein catabolic process, MAPK cascade, regulation of steroid biosynthetic process, cellular response to lipopolysaccharide, cholesterol transport, lipopolysaccharide-mediated signalling pathway and regulation of fatty acid metabolic process (Fig. 2B). The signalling pathways mainly consisted of two groups: the ER-phagosome pathway and TRAF6-mediated induction of pro-inflammatory cytokines (Fig. 2C). Based on these data, a hypothesis was proposed that the mechanisms of the protective effects of JZG against the development of NAFLD were linked to the autophagy and anti-inflammatory pathways.

#### PPI network generation and putative target identification for JZG against NAFLD

**Figure 3:**
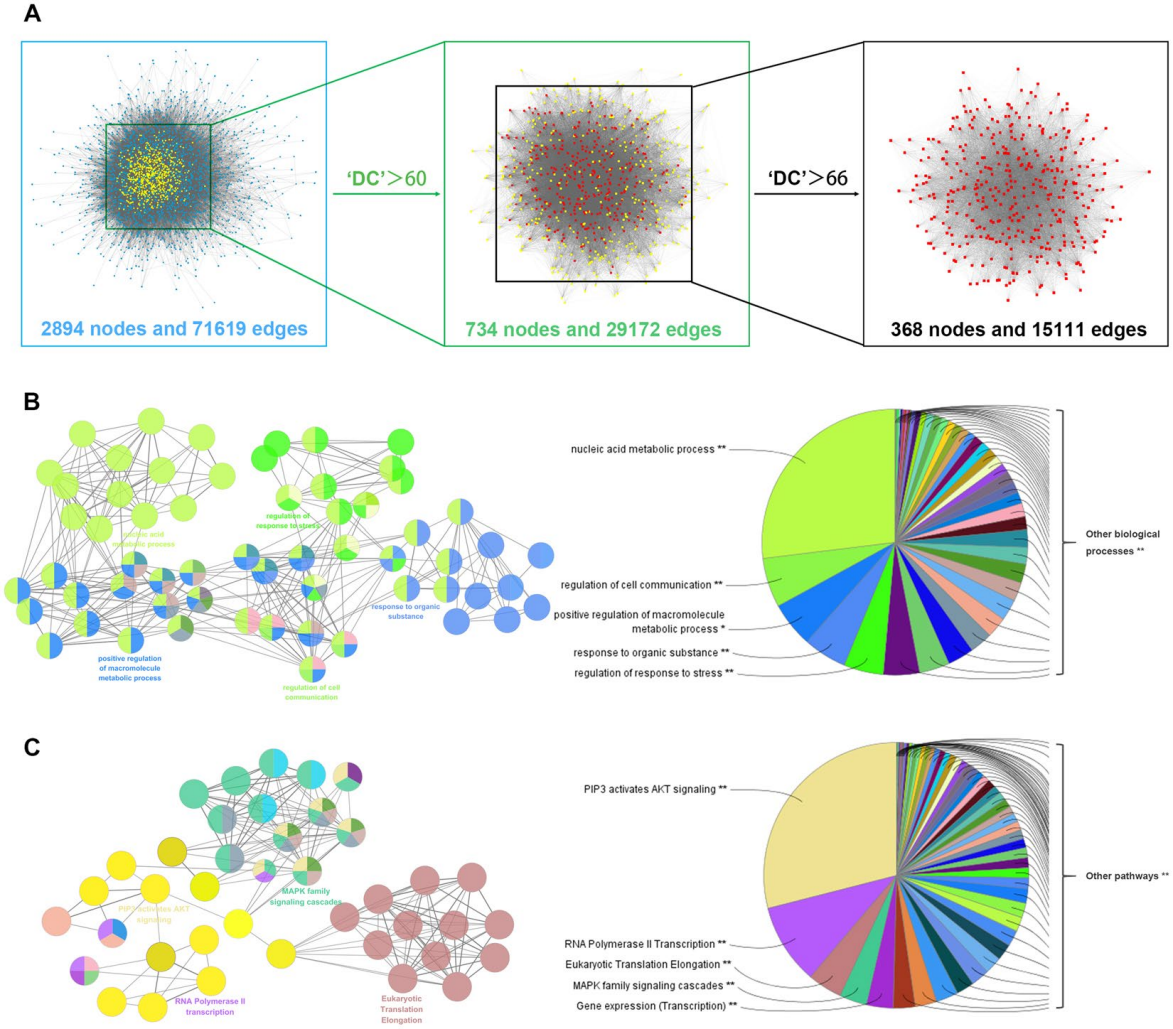
PPI network generation and putative target identification for JZG against NAFLD. (**A**) PPI networks were constructed for the putative targets of JZG as well as for known NAFLD-related targets, and the intersection of these two networks was used to identify candidate targets of JZG against NAFLD based on the DC value; (**B**) Enrichment analysis was performed using ClueGO to investigate the biological processes of JZG against NAFLD; (**C**) Enrichment analysis was performed using ClueGO to investigate the signalling pathways of JZG against NAFLD.

To obtain a better understanding of the mechanisms of JZG against NAFLD, 161 NAFLD-related targets were collected from the Genetic Association Database (GAD), Online Mendelian Inheritance in Man database (OMIM), Therapeutic Target Database (TTD) and GeneCards Database (Supplementary Table S4). Based on previous studies, protein-protein interaction (PPI) networks were constructed for the putative targets of JZG (8746 nodes and 195025 edges) and for known NAFLD-related targets (3379 nodes and 76045 edges). The intersection of the two networks contained 2894 nodes and 71619 edges, which was used to identify the candidate targets of JZG against NAFLD^19,27^. Finally, the candidate target screening was performed twice under the conditions of DC > 60 and DC > 66 (the median value of DC for each network), and the numbers of putative targets were 734 and 368, respectively (Fig. 3A).

ClueGO was applied once more to further elucidate the possible mechanisms of JZG against NAFLD, and the results were also classified into biological processes and signalling pathways. The biological processes mainly consisted of nucleic acid metabolic process, regulation of cell communication, positive regulation of macromolecule metabolic process, response to organic substance and regulation of response to stress (Fig. 3B), whereas the signalling pathways were mainly related to PIP3 activates AKT signalling, RNA polymerase II transcription, eukaryotic translation elongation and MAPK family signalling cascades (Fig. 3C). Since PIP3 activation of AKT signalling and the MAPK family signalling cascades are both upstream events of mTOR, a core target that is critically involved in autophagy induction and can suppress lipid droplet degradation, we postulated that the underlying mechanisms of the anti-steatotic effects of JZG against NAFLD were related to the activation of autophagy.

### Experimental validation

#### JZG could activate autophagy in hepatocytes

**Figure 4:**
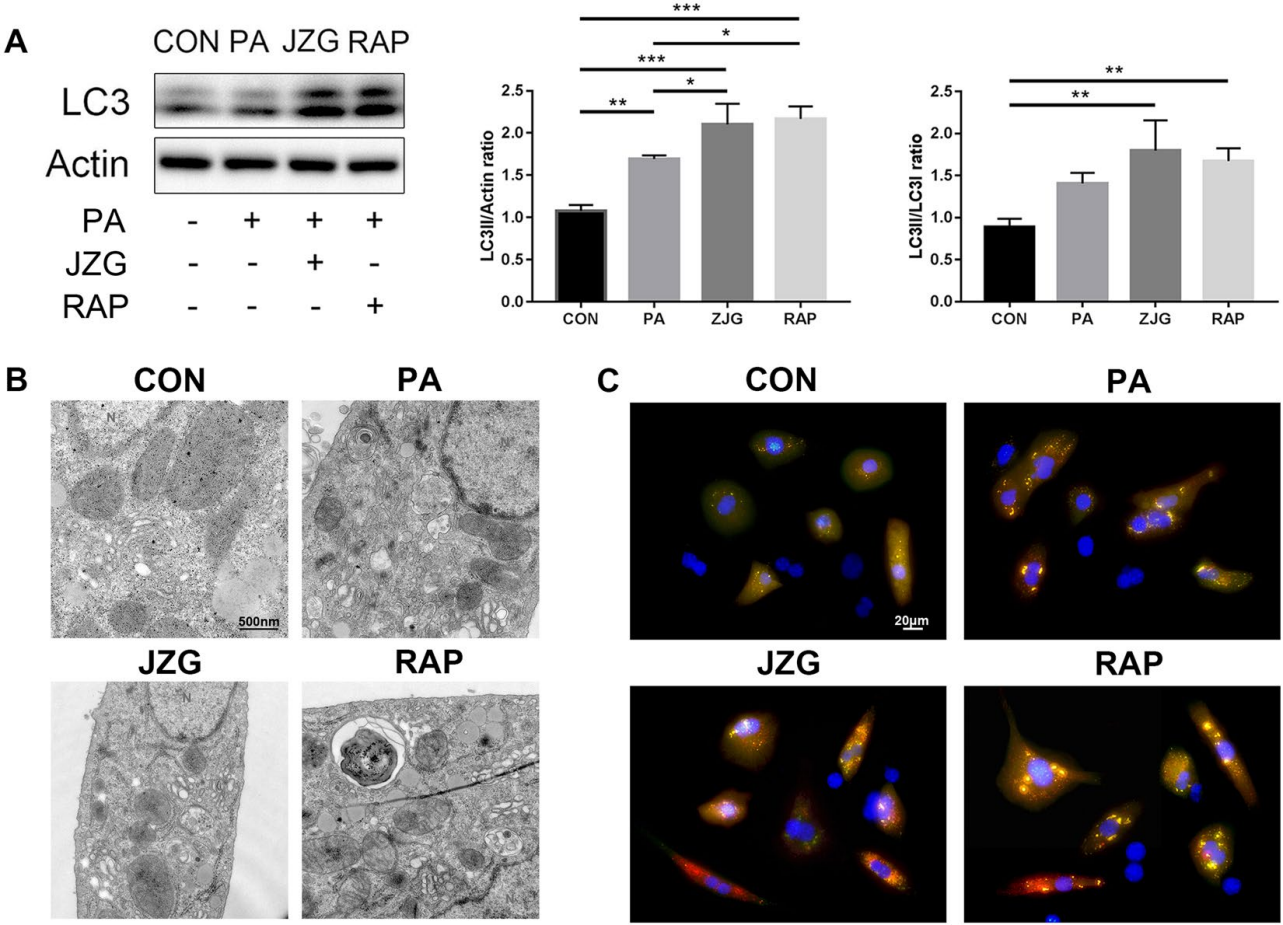
JZG could activate autophagy in hepatocytes. Hepatocytes were treated with PA (0.4 mM) in the presence or absence of either JZG (100 μg/mL) or rapamycin (2 μM) for 24 h. (**A**) Western blot analyses, the groupings were cropped from different gels subjected to identical conditions; (**B**) Autophagosomes were observed by TEM; (**C**) The progression of autophagosome formation was visualized by a cell line stably expressing mRFP-GFP-LC3. Data are expressed as the mean ± SE. ^*^P < 0.05, ^**^P < 0.01, ^***^P < 0.001.

To validate our hypothesis, hepatocytes were subjected to the conditions described above in the presence or absence of rapamycin (RAP), an inhibitor of mTOR, which served as a positive control. The results turned out that PA increased the ratio of LC3-II/actin expression and JZG could accelerate this process (Fig. 4A), indicating general activation of autophagy to degrade lipid droplets. In addition, the ratio of LC3-II/LC3-I expression was also enhanced, which means that autophagic flux was unobstructed. In addition, autophagosomes were observed in hepatocytes treated with PA, JZG and RAP by transmission electron microscopy (TEM) (Fig. 4B), confirming the previous findings.

A cell line stably expressing mRFP-GFP-LC3 was used in this study and diffuse cytoplasmic localization of yellow fluorescence presented undegraded LC3 was observed in hepatocytes (Fig. 4C). PA promoted the recruitment of autophagosomes (punctate fluorescence), however, degraded LC3 (red fluorescence) was rare in this group. By contrast, red fluorescence was largely present in the JZG and RAP groups, suggesting that autophagy was accelerated. Taken together, these results demonstrated that JZG could activate autophagy in hepatocytes.

#### JZG could regulate the mTOR signalling

**Figure 5:**
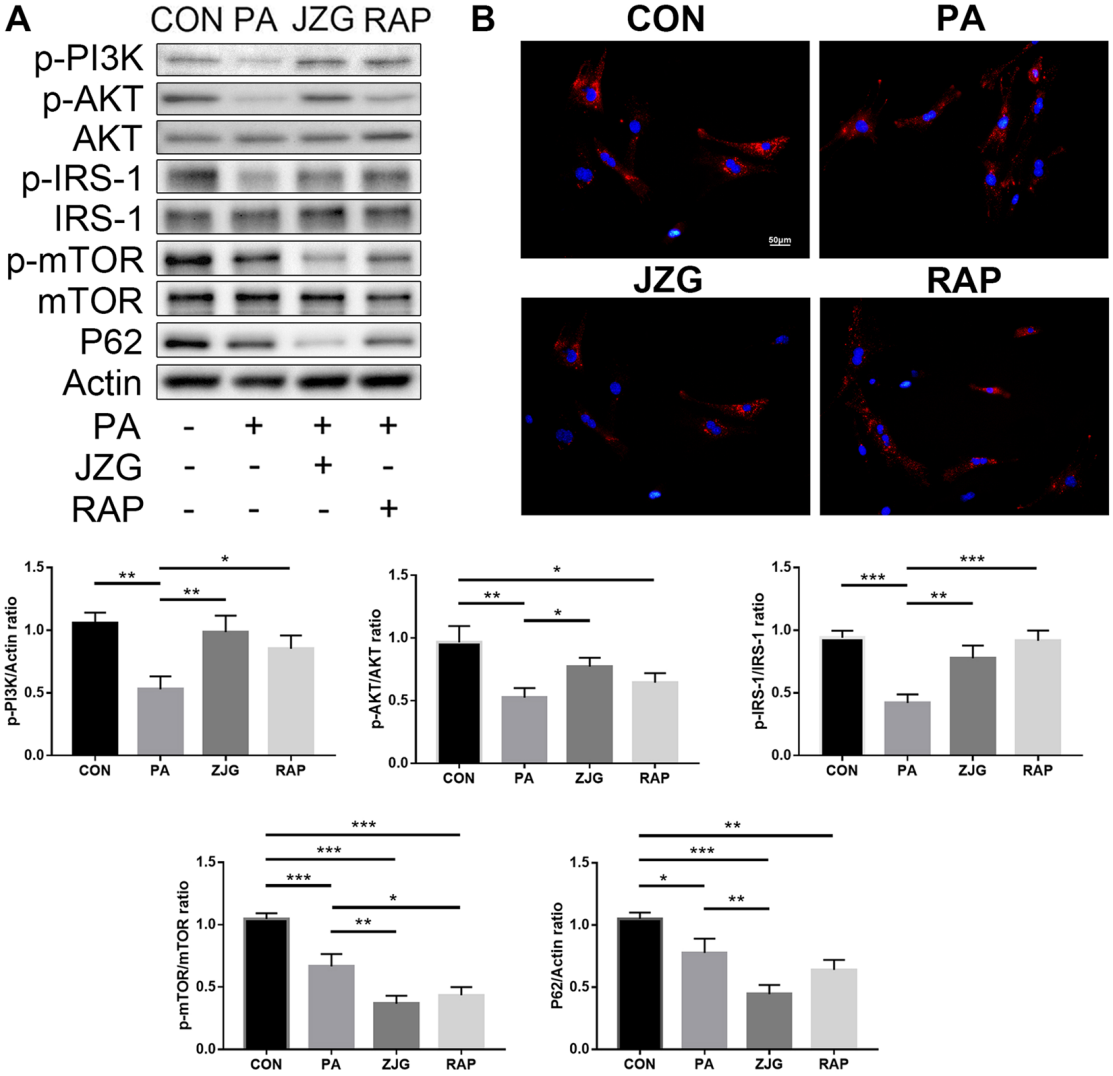
JZG could regulate the mTOR signalling. Hepatocytes were treated with PA (0.4 mM) in the presence or absence of either JZG (100 μg/mL) or rapamycin (2 μM) for 24 h. (**A**) Western blot analyses, the groupings were cropped from different gels subjected to identical conditions; (**B**) The progression of autophagic flux was visualized by a cell line stably expressing mCherry-p62. ^*^P < 0.05, ^**^P < 0.01, ^***^P < 0.001.

As systems pharmacology analyses revealed that the PIP3 activates AKT signalling accounts for the biggest weight in all signaling pathways, we examined the expression levels of PI3K, AKT and mTOR to confirm this observation. Interestingly, western blot analyses showed that the ratios of p-PI3K/actin, p-AKT/AKT and p-mTOR/mTOR expression were all decreased in hepatocytes treated with PA, however, JZG further decreased the p-mTOR/mTOR expression whereas elevated the expression levels of p-PI3K/actin and p-AKT/AKT (Fig. 5A). Since the activation of mTOR could negatively regulate the phosphorylation of IRS-1^28^, we postulated that JZG could inhibit the mTOR activation to up-regulate the IRS-1/PI3K/AKT signalling conversely as well as to activate autophagy.

To validate this hypothesis, we examined the expression level of IRS-1, and the result was in accordance with those of PI3K and AKT (Fig. 5A), suggesting the up-regulation of IRS-1 by JZG. Another cell line stably expressing mCherry-p62 was established, and the amount of red fluorescence was significantly reduced in the JZG and RAP groups (Fig. 5B), indicated that autophagic flux was unobstructed. The expression level of SQSTM1/p62 was examined to confirm that (Fig. 5A), and the result showed that autophagy was activated by JZG.

#### JZG protected mitochondrial integrity by ameliorating hepatic lipid accumulation

**Figure 6:**
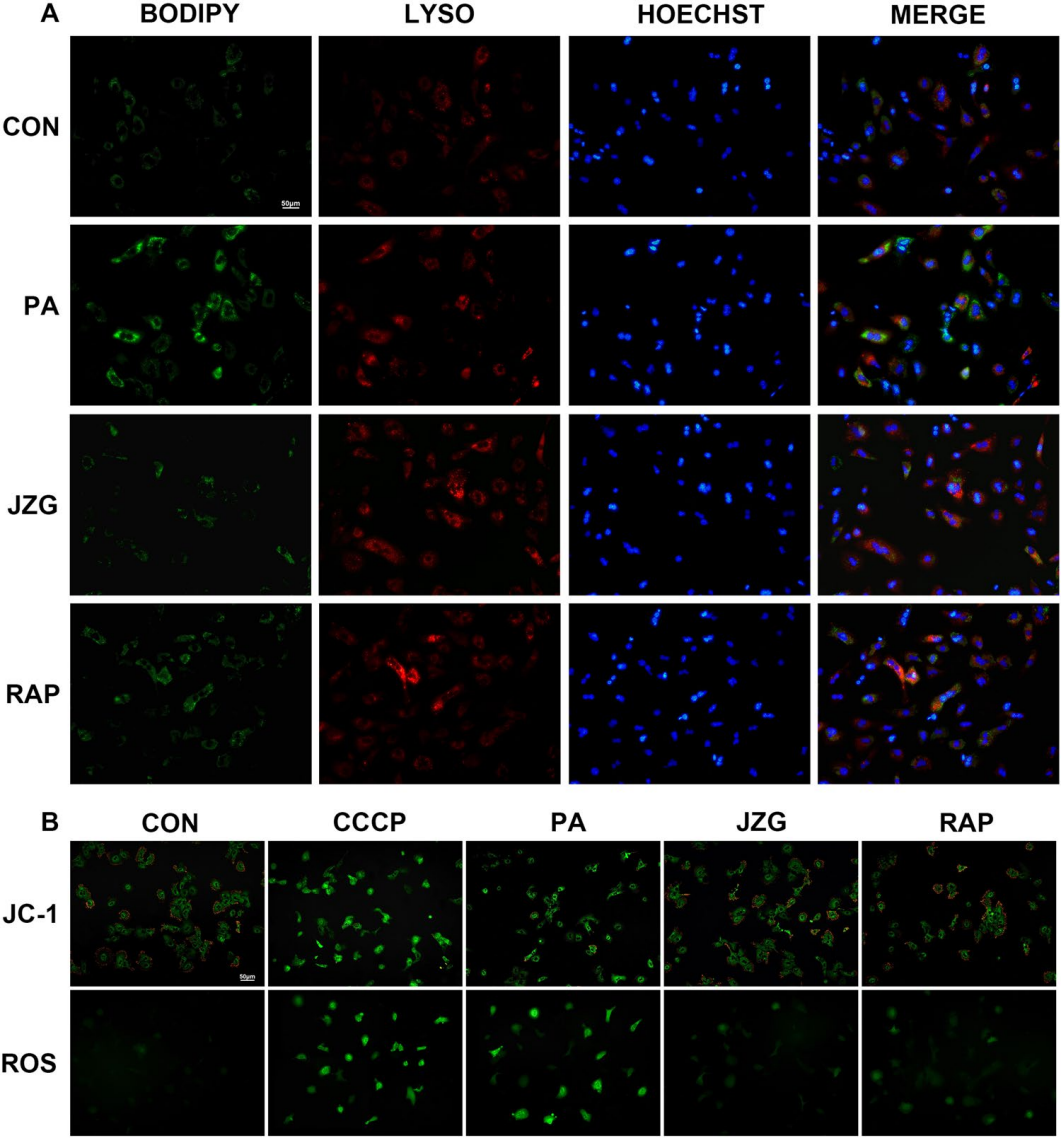
JZG protected mitochondrial integrity by ameliorating hepatic lipid accumulation. Hepatocytes were treated with PA (0.4 mM) in the presence or absence of either JZG (100 μg/mL) or rapamycin (2 μM) for 24 h. (**A**) Fluorescence staining with BODIPY and Lyso-Tracker was performed to demonstrate the co-localization of lipid droplets and lysosomes; (**B**): The mitochondrial integrity and ROS generation was detected by JC-1 and DCFH-DA, respectively.

As the anti-steatotic effects of JZG against NAFLD were confirmed to be related to the degradation of lipid droplets by autophagy, fluorescence staining using BODIPY and Lyso-Tracker was performed to demonstrate the localization of the lipid droplets and lysosomes, respectively. The green punctate fluorescence of lipid droplets in hepatocytes treated with PA was much stronger than that in cells treated with JZG, whereas the co-localization of red and green fluorescence was much weaker (Fig. 6A), indicating that JZG could promote the co-localization of lipid droplets and lysosomes for lipid droplet degradation.

Since mitochondrial injury and oxidative stress owing to lipid accumulation are considered typical pathological features during the progression of NAFLD, we then examined the mitochondrial membrane potential (MTP) and the ROS generation. Staining results revealed an obvious decrease of MTP and a considerably increased accumulation of ROS in hepatocytes treated with PA (Fig. 6B), indicating oxidative damage of mitochondria. And JZG significantly ameliorated these condition (Fig. 6B), suggesting the protective effect of JZG on mitochondrial integrity.

#### JZG could activate autophagy to improve NAFLD *in vivo*

**Figure 7:**
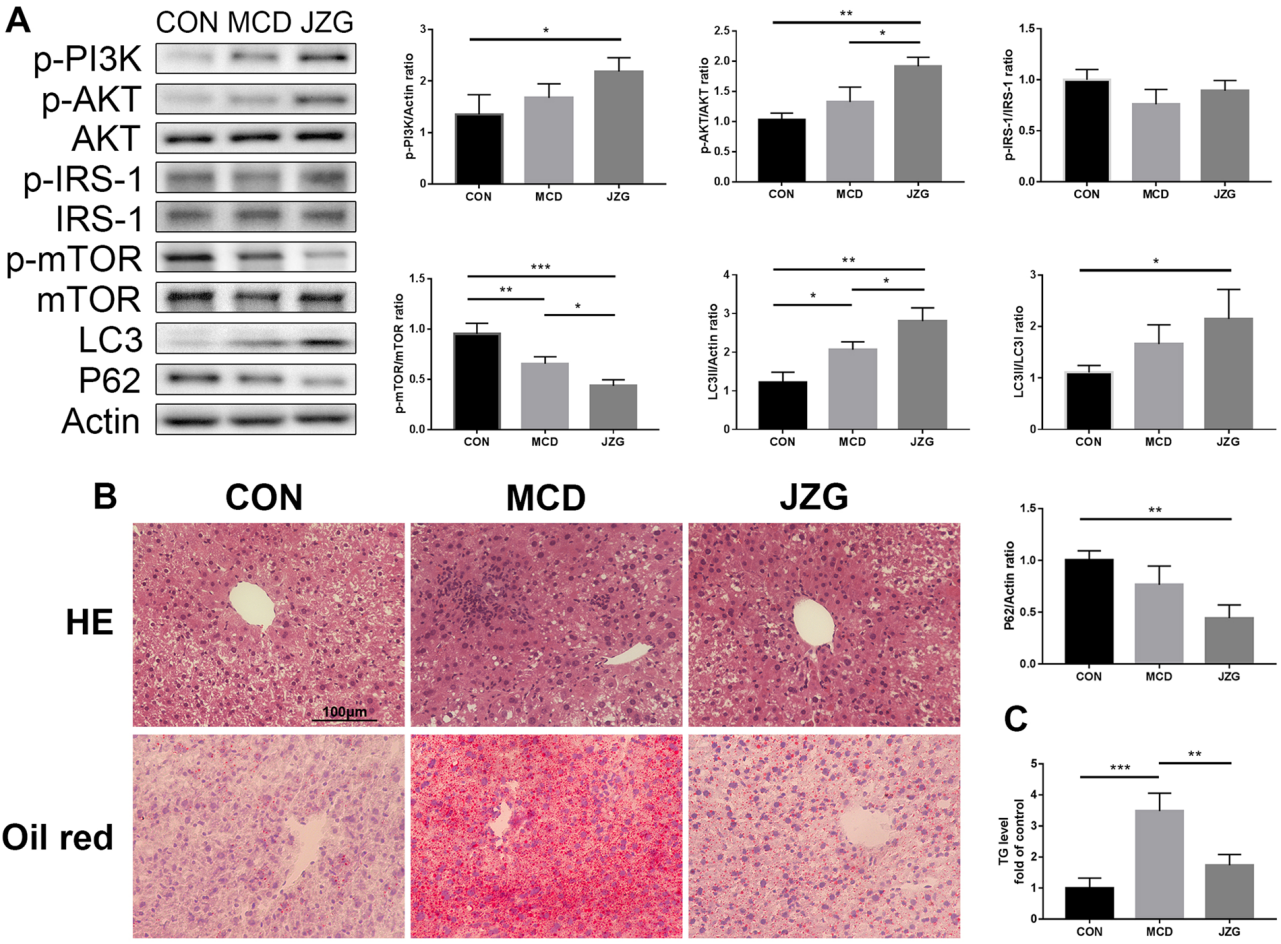
JZG could activate autophagy to improve NAFLD *in vivo*. Male C57BL/6 mice were fed either a standard or MCD diet for three weeks. Mice in the JZG group were administered JZG (994 mg/kg) once daily *via* oral gavage, whereas mice in the other two groups were administered normal saline (vehicle). (**A**) Western blot analyses, the expression of autophagy-related proteins in liver tissues of the mice and the groupings were cropped from different gels subjected to identical conditions; (**B**) Inflammation and lipid droplet in the liver was examined by Haematoxylin and Eosin (H&E) staining and Oil Red O staining, respectively; (**C**) Liver TG levels. ^*^P < 0.05, ^**^P < 0.01, ^***^P < 0.001.

A murine model of NAFLD induced by a methionine-choline-deficient (MCD) diet was employed in this study to assess whether autophagy is involved in the mechanism by which JZG ameliorates NAFLD^29^. As the IRS-1/PI3K/AKT signalling pathway is not affected in this animal model, the ratios of p-IRS-1/IRS-1, p-PI3K/actin and p-AKT/AKT were not significantly changed in the MCD group compared with the control group; however, the ratio of LC3-II/actin expression increased, and the p-mTOR/mTOR ratio decreased (Fig. 7A), indicating activation of autophagy. Changes in the LC3-II/LC3-I and p62/actin ratios were not significant, which might be due to the mild autophagy response; however, changes in the p-AKT/AKT, p-mTOR/mTOR and LC3-II/actin ratios were significant compared with those in the MCD group (Fig. 7A), which indicated that autophagy is accelerated by JZG in the NAFLD mouse model.

The results of the H&E and Oil Red staining showed that lipid droplet accumulation and inflammation were induced by an MCD diet but were ameliorated by JZG (Fig. 7B), which demonstrated both the anti-steatotic and anti-inflammatory effects of JZG against NAFLD. And the levels of triglyceride (TG) in liver tissues confirmed the histological results. Taken together, these results demonstrated that JZG could activate autophagy to improve NAFLD *in vivo*.

## Discussion

**Figure 8:**
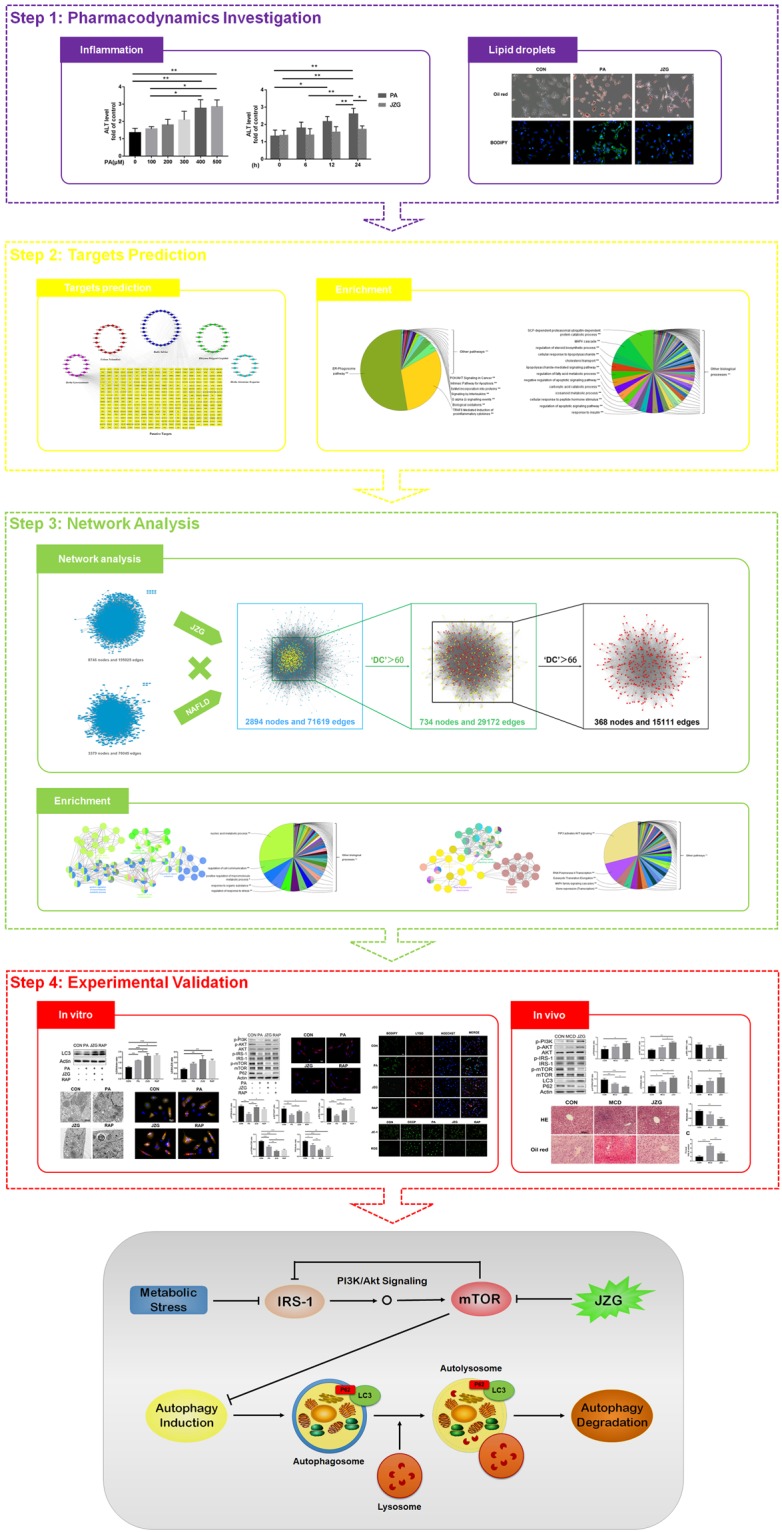
The schematic diagram of the systems pharmacology approach and the potential mechanisms of the anti-steatotic effects of JZG on NAFLD.

NAFLD, which is in parallel with the obesity epidemic, has been increasingly recognized as the most prevalent chronic liver disease^2^. As the anti-steatotic effects of JZG against NAFLD have been demonstrated, we explored the cellular and molecular pharmacological mechanisms using a systems pharmacology approach. The flow chart is shown in Fig. 8.

The therapeutic efficacy of JZG was confirmed in the beginning of this study, as JZG significantly improved hepatic dysfunction and lipid droplet accumulation in PA-treated hepatocytes. Systems pharmacology was then performed to identify the active compounds and to predict the therapeutic targets of this TCM prescription. Finally, 338 putative targets were predicted for 96 candidate compounds among these five herbs, and surprisingly, there were many compounds and targets that overlapped, suggesting that the different herbal components of JZG shared similar ingredients that could regulate similar targets to exert synergistic effects. However, the compound-target network of all 96 compounds and their corresponding targets revealed that most candidate compounds had a median DC value, indicating that the compounds could influence multiple targets to exert various effects.

PPI networks were constructed for the putative targets of JZG as well as for known NAFLD-related targets, and candidate target screening was performed based on the intersection of these two networks. Enrichment analyses were performed twice for candidate targets of either JZG or JZG against NAFLD, and the outcomes showed that the mechanisms of the protective effects of JZG against the development of NAFLD might be associated with lipid droplet degradation by autophagy (cholesterol transport, regulation of fatty acid metabolic process, positive regulation of macromolecule metabolic process, ER-phagosome pathway, PIP3 activates AKT signalling and MAPK family signalling cascades) and anti-inflammatory activity (regulation of steroid biosynthetic process, cellular response to lipopolysaccharide, lipopolysaccharide-mediated signalling pathway and anti-inflammatory pathways). As anti-inflammatory activities have been confirmed to not be involved in the anti-steatotic effects of JZG against NAFLD, this thesis focused on activation of autophagy.

Studies have shown that autophagy is a crucial cellular homeostatic mechanism that can regulate the level of metabolic factors in liver^30,31^. In the meantime, autophagy regulators have been proven effective in improving hepatic function and lipid accumulation^32^. Since the accumulation of lipid droplets due to energy metabolism dysfunction is a key event during the development of NAFLD, we performed experimental validation *in vitro* and *in vivo* to confirm the previous findings.

Autophagosomes as observed by TEM and LC3 expression were used to confirm the induction of autophagy in hepatocytes treated with either PA or JZG, while the autophagic flux was assessed by SQSTM1/p62 expression. As studies have suggested that the excessive lipid droplet accumulation might contribute to the p62 accumulation *via* suppression of autophagosome and lysosome fusion^33,34^, we postulated that autophagic flux could be obstructed by PA. However, no discovery of this phenomenon was found in this study, which might be attributed to the different time periods and treatment concentrations. A great number of signalling pathways have been discovered during the past decade, and we postulated that the mTOR signalling was critically involved in the observed activities of JZG-treated cells. Unlike our previous findings^21^, we confirmed that JZG could inhibit the mTOR activation to up-regulate the IRS-1/PI3K/AKT signalling conversely as well as to activate autophagy. Fluorescence staining with BODIPY and Lyso-Tracker indicated that JZG could promote the co-localization of lipid droplets and lysosomes for degradation, and we also measured the MTP and ROS generation to demonstrate that JZG could protect mitochondrial integrity against oxidative stress. Taken together, these results suggested that JZG could activate autophagy though the mTOR signalling to improve NAFLD (Fig. 8).

The limitations of this study should be acknowledged. The anti-inflammatory effects of JZG against NAFLD were also observed in the H&E-stained samples, which might be related to autophagy; however, this study was focused on the anti-steatotic effects of JZG. Future studies are still needed to explore other signalling pathways and regulators, as most TCM prescriptions could exert various therapeutic effects. Beyond that, the systems pharmacology approach identified the active compounds and predicted the therapeutic targets in these five herbs; however, the exact compounds and targets responsible for the anti-steatotic effects are still unknown and deserve further investigation. Thus, future studies to examine the synergistic therapeutic effects of specific compounds in JZG are necessary, and they may provide some insights to collaborative therapeutic approaches to improve the current standardized intervention strategy.

## Methods

### Systems pharmacology

The chemical compositions of all five herbs that constitute JZG were obtained from the Traditional Chinese Medicine System Pharmacology Database (TCMSP), and the oral bioavailability (OB) was used as the screening index to identify the candidate compounds^23^. Compounds with an OB ≥ 30% were harvested in this study, and others with a lower OB value but were the typical components of other herbal drugs were also manually retrieved owing to their extensive pharmacological activities^35^. Known NAFLD-related targets were collected from the Genetic Association Database (GAD), Online Mendelian Inheritance in Man database (OMIM), Therapeutic Target Database (TTD) and GeneCards Database, and protein-protein interaction (PPI) networks were constructed and intersected with the Cytoscape plug-in Bisogenet^36^. The degree centrality (DC) value, which represents the topological importance of a node in the network, was used to predict the putative targets, and the enrichment analyses were performed using the Cytoscape plugin ClueGO^26^.

### Animal models

Wild-type C57BL/6 J mice (6 weeks old) were purchased from Sippr-BK Laboratory Animal Co. Ltd. The mice were divided into three groups: control group (n = 10), which received a standard diet; the methionine-choline-deficient (MCD) group (n = 10), which received an MCD diet (consisting of 40% sucrose, 10% fat, and a lack of methionine and choline)^29^; and the JZG group (n = 10), which received an MCD diet supplemented with JZG (daily oral gavage of 994 mg/kg in saline). At three weeks after diet and drug administration, sodium pentobarbital was given as anaesthesia to the mice. Blood samples were drawn via cardiac puncture, while livers were excised as described previously^21^.

All animals were handled humanely throughout all the experiments, which were performed in accordance with the approved guidelines of the Experimental Animal Care and Ethics Committee of the Department of Laboratory Animal Science, Fudan University.

### Cell culture and establishment of stable fluorescent-expressing cell lines

Primary hepatocytes were harvested from wild-type C57BL/6 J mice. Livers were perfused, digested, separated, ground and filtrated to collect hepatocytes. Density gradient centrifugation with Nycodenz was performed to purify the hepatocytes, and 6-well plates were processed with gelatine to promote cell attachment. Hepatocytes were cultured following established protocols and infected with lentivirus as described previously^21^.

### Fluorescence staining

Lipid droplets were monitored using a BODIPY 493/503 staining kit as well as an Oil Red O staining kit, and lysosomes were stained with a Lyso-Tracker Deep Red staining kit. The MTP was detected using JC-1, and the ROS generation was determined by DCFH-DA. Pre-treated hepatocytes were processed according to the abovementioned protocols as described previously^21^.

### Western blot

Primary antibodies against p-AKT (phospho S473), AKT, p-IRS-1 (phospho S1101), IRS-1, LC3 and SQSTM1/p62 were purchased from CST, and against p-PI3K (phospho Y607), p-mTOR (phospho S2448), mTOR and actin were purchased from Abcam. The groupings of the blots were cropped from different gels subjected to identical conditions, and the full-length gels are shown in Supplementary Fig. S1.

### Transmission electron microscope (TEM)

Pre-treated hepatocytes were fixed with 3% glutaraldehyde at 4 °C overnight, processed, dehydrated, paraffin-embedded and sectioned to a 70-nm thickness. These ultra-thin cell sections were stained with uranyl acetate and lead citrate and were observed using TEM (JEOL-1200EX) recording by MORADA-G2.

### Histological staining

Liver tissues were processed and stained with Haematoxylin and Eosin (H&E) and Oil Red O as described previously^21^.

### Statistical analysis

All results were presented as the mean ± SEM, and the differences between groups were determined by ANOVA. As appropriate, a two-sided *P* value less than 0.05 was considered statistically significant. Statistical analyses were performed by GraphPad Prism.

## Electronic supplementary material

Supplementary Information file

## Data Availability

All data generated or analysed during this study are included in this published article (and its Supplementary Information file).
