# Supplementary material for: Systems pharmacology-based exploration reveals mechanisms of anti-steatotic effects of Jiang Zhi Granule on non-alcoholic fatty liver disease
Source: Sci Rep. 2018 Sep 12;8:13681. doi: 10.1038/s41598-018-31708-8 (PMC6135841; doi:10.1038/s41598-018-31708-8)
Supplement: Supplementary file 1 — Supplementary Information file [file 41598_2018_31708_MOESM1_ESM.docx]

**Systems pharmacology-based exploration reveals mechanisms of anti-steatotic effects of Jiang Zhi Granule on non-alcoholic fatty liver disease**

Yiyuan Zheng^1,+^, Miao Wang^1,+^, Peiyong Zheng^1^, Xudong Tang^2^ & Guang Ji^1^

^1^Institute of Digestive Disease, Longhua Hospital, Shanghai University of Traditional Chinese Medicine, Shanghai 200032, China

^2^Xiyuan Hospital of China Academy of Chinese Medical Sciences, Beijing 100091, China

^+^These authors contributed equally to this work

*Corresponding author

E-mail: [jiliver@vip.sina.com](mailto:jiliver@vip.sina.com); Tel.: +86-021-64385700

**Supplementary Table S1. Candidate compounds of each ingredient in JZG**

| **NO** | **Herbname** | **Chemname** | **Molecule ID** | **OB** | **CID** | **InChIKey** |
| --- | --- | --- | --- | --- | --- | --- |
| **1** | *Herba Artemisiae Scopariae* (Yin Chen, HAS) | beta-elemene | MOL000908 | 25.63 | 6918391 | OPFTUNCRGUEPRZ-QLFBSQMISA-N |
| **2** | *Herba Artemisiae Scopariae* (Yin Chen, HAS) | Butal | MOL004734 | 68.66 | 261 | ZTQSAGDEMFDKMZ-UHFFFAOYSA-N |
| **3** | *Herba Artemisiae Scopariae* (Yin Chen, HAS) | caffeic acid | MOL000223 | 25.76 | 689043 | QAIPRVGONGVQAS-DUXPYHPUSA-N |
| **4** | *Herba Artemisiae Scopariae* (Yin Chen, HAS) | capillarisin | MOL008043 | 57.56 | 5281342 | NTKNGUAZSFAKEE-UHFFFAOYSA-N |
| **5** | *Herba Artemisiae Scopariae* (Yin Chen, HAS) | caproic acid | MOL002046 | 73.08 | 8892 | FUZZWVXGSFPDMH-UHFFFAOYSA-N |
| **6** | *Herba Artemisiae Scopariae* (Yin Chen, HAS) | d-limonene | MOL000023 | 39.84 | 440917 | XMGQYMWWDOXHJM-JTQLQIEISA-N |
| **7** | *Herba Artemisiae Scopariae* (Yin Chen, HAS) | eugenol | MOL000254 | 56.24 | 3314 | RRAFCDWBNXTKKO-UHFFFAOYSA-N |
| **8** | *Herba Artemisiae Scopariae* (Yin Chen, HAS) | Furol | MOL000172 | 34.35 | 7362 | HYBBIBNJHNGZAN-UHFFFAOYSA-N |
| **9** | *Herba Artemisiae Scopariae* (Yin Chen, HAS) | Genkwanin | MOL005573 | 37.13 | 5281617 | JPMYFOBNRRGFNO-UHFFFAOYSA-N |
| **10** | *Herba Artemisiae Scopariae* (Yin Chen, HAS) | Hirsutrin | MOL000437 | 1.86 | 5280804 | OVSQVDMCBVZWGM-QSOFNFLRSA-N |
| **11** | *Herba Artemisiae Scopariae* (Yin Chen, HAS) | isorhamnetin | MOL000354 | 49.60 | 5281654 | IZQSVPBOUDKVDZ-UHFFFAOYSA-N |
| **12** | *Herba Artemisiae Scopariae (Yin Chen, HAS)* | Methyleugenol | MOL000207 | 73.36 | 7127 | ZYEMGPIYFIJGTP-UHFFFAOYSA-N |
| **13** | *Herba Artemisiae Scopariae (Yin Chen, HAS)* | Myristicin | MOL000261 | 17.99 | 4276 | BNWJOHGLIBDBOB-UHFFFAOYSA-N |
| **14** | *Herba Artemisiae Scopariae (Yin Chen, HAS)* | oleovitamin a | MOL005567 | 19.53 | 445354 | FPIPGXGPPPQFEQ-OVSJKPMPSA-N |
| **15** | *Herba Artemisiae Scopariae* (Yin Chen, HAS) | p-cresol | MOL002830 | 51.99 | 2879 | IWDCLRJOBJJRNH-UHFFFAOYSA-N |
| **16** | *Herba Artemisiae Scopariae* (Yin Chen, HAS) | quercetin | MOL000098 | 46.43 | 5280343 | REFJWTPEDVJJIY-UHFFFAOYSA-N |
| **17** | *Herba Artemisiae Scopariae* (Yin Chen, HAS) | rutin | MOL000415 | 3.20 | 5280805 | IKGXIBQEEMLURG-NVPNHPEKSA-N |
| **18** | *Herba Artemisiae Scopariae* (Yin Chen, HAS) | salicylic acid | MOL001801 | 32.13 | 338 | YGSDEFSMJLZEOE-UHFFFAOYSA-N |
| **19** | *Herba Artemisiae Scopariae* (Yin Chen, HAS) | scoparone | MOL001999 | 74.75 | 8417 | GUAFOGOEJLSQBT-UHFFFAOYSA-N |
| **20** | *Herba Artemisiae Scopariae* (Yin Chen, HAS) | Scopoletol | MOL000040 | 27.77 | 5280460 | RODXRVNMMDRFIK-UHFFFAOYSA-N |
| **21** | *Herba Artemisiae Scopariae* (Yin Chen, HAS) | vanillin | MOL000635 | 52.00 | 1183 | MWOOGOJBHIARFG-UHFFFAOYSA-N |
| **22** | *Radix Salviae* (Dan Shen, RS) | (2S)-3-(3,4-dihydroxyphenyl)-2-hydroxypropanoic acid | MOL007072 | 59.56 | 179536 | PAFLSMZLRSPALU-QMMMGPOBSA-N |
| **23** | *Radix Salviae* (Dan Shen, RS) | apigenin | MOL000008 | 23.06 | 5280443 | KZNIFHPLKGYRTM-UHFFFAOYSA-N |
| **24** | *Radix Salviae* (Dan Shen, RS) | Baicalin | MOL002776 | 40.12 | 64982 | IKIIZLYTISPENI-ZFORQUDYSA-N |
| **25** | *Radix Salviae* (Dan Shen, RS) | caffeic acid | MOL000223 | 25.76 | 689043 | QAIPRVGONGVQAS-DUXPYHPUSA-N |
| **26** | *Radix Salviae* (Dan Shen, RS) | cryptotanshinone | MOL007088 | 52.34 | 160254 | GVKKJJOMQCNPGB-JTQLQIEISA-N |
| **27** | *Radix Salviae* (Dan Shen, RS) | Danshenol A | MOL007082 | 56.97 | 3083514 | WQYKPUPMMFGHQW-QKVFXAPYSA-N |
| **28** | *Radix Salviae* (Dan Shen, RS) | danshensu | MOL007134 | 36.91 | 11600642 | PAFLSMZLRSPALU-MRVPVSSYSA-N |
| **29** | *Radix Salviae* (Dan Shen, RS) | dihydrotanshinone i | MOL007101 | 45.04 | 40785034 | HARGZZNYNSYSGJ-JTQLQIEISA-N |
| **30** | *Radix Salviae* (Dan Shen, RS) | DTY | MOL000056 | 57.55 | 6942100 | OUYCCCASQSFEME-QMMMGPOBSA-N |
| **31** | *Radix Salviae* (Dan Shen, RS) | Glucosol | MOL005508 | 15.86 | 6918774 | HFGSQOYIOKBQOW-ZSDYHTTISA-N |
| **32** | *Radix Salviae* (Dan Shen, RS) | Gulutamine | MOL000052 | 6.66 | 44272391 | WHUUTDBJXJRKMK-VKHMYHEASA-N |
| **33** | *Radix Salviae* (Dan Shen, RS) | h-Met-h | MOL005449 | 70.87 | 6992087 | FFEARJCKVFRZRR-BYPYZUCNSA-N |
| **34** | *Radix Salviae* (Dan Shen, RS) | isoferulic acid | MOL005928 | 50.83 | 736186 | QURCVMIEKCOAJU-HWKANZROSA-N |
| **35** | *Radix Salviae* (Dan Shen, RS) | isoimperatorin | MOL001942 | 45.46 | 68081 | IGWDEVSBEKYORK-UHFFFAOYSA-N |
| **36** | *Radix Salviae* (Dan Shen, RS) | Istidina | MOL000071 | 53.18 | 6971009 | HNDVDQJCIGZPNO-YFKPBYRVSA-N |
| **37** | *Radix Salviae* (Dan Shen, RS) | Leucinum | MOL005448 | 72.92 | 7045798 | ROHFNLRQFUQHCH-YFKPBYRVSA-N |
| **38** | *Radix Salviae* (Dan Shen, RS) | L-Ile | MOL000068 | 59.05 | 7043901 | AGPKZVBTJJNPAG-WHFBIAKZSA-N |
| **39** | *Radix Salviae* (Dan Shen, RS) | L-Lysin | MOL000055 | 29.33 | 5962 | KDXKERNSBIXSRK-YFKPBYRVSA-N |
| **40** | *Radix Salviae* (Dan Shen, RS) | LPG | MOL000042 | 87.69 | 7311724 | QNAYBMKLOCPYGJ-REOHCLBHSA-N |
| **41** | *Radix Salviae* (Dan Shen, RS) | L-Serin | MOL003969 | 98.47 | 6857581 | MTCFGRXMJLQNBG-REOHCLBHSA-N |
| **42** | *Radix Salviae* (Dan Shen, RS) | luteolin | MOL000006 | 36.16 | 5280445 | IQPNAANSBPBGFQ-UHFFFAOYSA-N |
| **43** | *Radix Salviae* (Dan Shen, RS) | luteolin-7-o-glucoside | MOL000009 | 7.29 | 5280637 | PEFNSGRTCBGNAN-QNDFHXLGSA-N |
| **44** | *Radix Salviae* (Dan Shen, RS) | L-Valin | MOL000067 | 53.33 | 6971018 | KZSNJWFQEVHDMF-BYPYZUCNSA-N |
| **45** | *Radix Salviae* (Dan Shen, RS) | Miltirone | MOL007122 | 38.76 | 160142 | FEFAIBOZOKSLJR-UHFFFAOYSA-N |
| **46** | *Radix Salviae* (Dan Shen, RS) | oleanolic acid | MOL000263 | 29.02 | 10494 | MIJYXULNPSFWEK-GTOFXWBISA-N |
| **47** | *Radix Salviae* (Dan Shen, RS) | PHA | MOL000041 | 41.62 | 6925665 | COLNVLDHVKWLRT-QMMMGPOBSA-N |
| **48** | *Radix Salviae* (Dan Shen, RS) | Physcion | MOL000476 | 22.29 | 10639 | FFWOKTFYGVYKIR-UHFFFAOYSA-N |
| **49** | *Radix Salviae* (Dan Shen, RS) | poriferast-5-en-3beta-ol | MOL001771 | 36.91 | 457801 | KZJWDPNRJALLNS-FBZNIEFRSA-N |
| **50** | *Radix Salviae* (Dan Shen, RS) | Prolinum | MOL000061 | 77.57 | 6971047 | ONIBWKKTOPOVIA-BYPYZUCNSA-N |
| **51** | *Radix Salviae* (Dan Shen, RS) | protocatechuic acid | MOL000105 | 25.37 | 72 | YQUVCSBJEUQKSH-UHFFFAOYSA-N |
| **52** | *Radix Salviae* (Dan Shen, RS) | rutin | MOL000415 | 3.20 | 5280805 | IKGXIBQEEMLURG-NVPNHPEKSA-N |
| **53** | *Radix Salviae* (Dan Shen, RS) | succinic acid | MOL000346 | 29.62 | 21952380 | KDYFGRWQOYBRFD-UHFFFAOYSA-N |
| **54** | *Radix Salviae* (Dan Shen, RS) | tanshinone i | MOL007157 | 29.27 | 114917 | AIGAZQPHXLWMOJ-UHFFFAOYSA-N |
| **55** | *Radix Salviae* (Dan Shen, RS) | tanshinone iia | MOL007154 | 49.89 | 164676 | HYXITZLLTYIPOF-UHFFFAOYSA-N |
| **56** | *Radix Salviae* (Dan Shen, RS) | Threonin | MOL003971 | 73.52 | 6971019 | AYFVYJQAPQTCCC-GBXIJSLDSA-N |
| **57** | *Radix Salviae* (Dan Shen, RS) | ursolic acid | MOL000511 | 16.77 | 64945 | WCGUUGGRBIKTOS-GPOJBZKASA-N |
| **58** | *Radix Salviae* (Dan Shen, RS) | VIV | MOL002771 | 14.26 | 14985 | GVJHHUAWPYXKBD-IEOSBIPESA-N |
| **59** | *Folium Nelumbinis* (He Ye, FN) | 3,4,5-trihydroxybenzoic acid | MOL000513 | 31.69 | 370 | LNTHITQWFMADLM-UHFFFAOYSA-N |
| **60** | *Folium Nelumbinis* (He Ye, FN) | anonaine | MOL009166 | 25.14 | N/A | VZTUKBKUWSHDFM-CYBMUJFWSA-N |
| **61** | *Folium Nelumbinis* (He Ye, FN) | CAM | MOL000130 | 67.17 | 159055 | DSSYKIVIOFKYAU-XCBNKYQSSA-N |
| **62** | *Folium Nelumbinis* (He Ye, FN) | catechol | MOL000089 | 29.86 | 289 | YCIMNLLNPGFGHC-UHFFFAOYSA-N |
| **63** | *Folium Nelumbinis* (He Ye, FN) | citric acid | MOL001456 | 56.22 | 19782904 | KRKNYBCHXYNGOX-UHFFFAOYSA-N |
| **64** | *Folium Nelumbinis* (He Ye, FN) | Hexenal | MOL002675 | 46.01 | 5281168 | MBDOYVRWFFCFHM-SNAWJCMRSA-N |
| **65** | *Folium Nelumbinis* (He Ye, FN) | higenamine | MOL006404 | 9.99 | N/A | WZRCQWQRFZITDX-UHFFFAOYSA-N |
| **66** | *Folium Nelumbinis* (He Ye, FN) | Hirsutrin | MOL000437 | 1.86 | 5280804 | OVSQVDMCBVZWGM-QSOFNFLRSA-N |
| **67** | *Folium Nelumbinis* (He Ye, FN) | isoliensinine | MOL009168 | 2.44 | 5274591 | AJPXZTKPPINUKN-FIRIVFDPSA-N |
| **68** | *Folium Nelumbinis* (He Ye, FN) | isorhamnetin | MOL000354 | 49.60 | 5281654 | IZQSVPBOUDKVDZ-UHFFFAOYSA-N |
| **69** | *Folium Nelumbinis* (He Ye, FN) | kaempferol | MOL000422 | 41.88 | 5280863 | IYRMWMYZSQPJKC-UHFFFAOYSA-N |
| **70** | *Folium Nelumbinis* (He Ye, FN) | liriodenine | MOL004217 | 14.83 | N/A | MUMCCPUVOAUBAN-UHFFFAOYSA-N |
| **71** | *Folium Nelumbinis* (He Ye, FN) | Machiline | MOL007207 | 79.64 | 440989 | LVVKXRQZSRUVPY-OAHLLOKOSA-N |
| **72** | *Folium Nelumbinis* (He Ye, FN) | MLT | MOL001468 | 59.62 | 222656 | BJEPYKJPYRNKOW-REOHCLBHSA-N |
| **73** | *Folium Nelumbinis* (He Ye, FN) | myricetin | MOL002008 | 13.75 | 5281672 | IKMDFBPHZNJCSN-UHFFFAOYSA-N |
| **74** | *Folium Nelumbinis* (He Ye, FN) | myristic acid | MOL001393 | 21.18 | 11005 | TUNFSRHWOTWDNC-UHFFFAOYSA-N |
| **75** | *Folium Nelumbinis* (He Ye, FN) | Nuciferin | MOL007213 | 34.43 | 10146 | ORJVQPIHKOARKV-OAHLLOKOSA-N |
| **76** | *Folium Nelumbinis* (He Ye, FN) | Pentenal | MOL007199 | 50.20 | 5364752 | DTCCTIQRPGSLPT-ONEGZZNKSA-N |
| **77** | *Folium Nelumbinis* (He Ye, FN) | PHB | MOL000103 | 30.15 | 3702506 | FJKROLUGYXJWQN-UHFFFAOYSA-N |
| **78** | *Folium Nelumbinis* (He Ye, FN) | quercetin | MOL000098 | 46.43 | 5280343 | REFJWTPEDVJJIY-UHFFFAOYSA-N |
| **79** | *Folium Nelumbinis* (He Ye, FN) | Remerin | MOL007218 | 40.75 | N/A | JCTYWRARKVGOBK-CQSZACIVSA-N |
| **80** | *Folium Nelumbinis* (He Ye, FN) | RNG | MOL007222 | 29.10 | 241 | UHOVQNZJYSORNB-UHFFFAOYSA-N |
| **81** | *Folium Nelumbinis* (He Ye, FN) | salicylic acid | MOL001801 | 32.13 | 338 | YGSDEFSMJLZEOE-UHFFFAOYSA-N |
| **82** | *Folium Nelumbinis* (He Ye, FN) | succinic acid | MOL000346 | 29.62 | 21952380 | KDYFGRWQOYBRFD-UHFFFAOYSA-N |
| **83** | *Folium Nelumbinis* (He Ye, FN) | Tar | MOL001886 | 66.38 | 439655 | FEWJPZIEWOKRBE-LWMBPPNESA-N |
| **84** | *Rhizoma Polygoni Cuspidati* (Hu Zhang, RPC) | (+)-Limacine | MOL002342 | 11.73 | N/A | IIQSJHUEZBTSAT-VMPREFPWSA-N |
| **85** | *Rhizoma Polygoni Cuspidati* (Hu Zhang, RPC) | 3,4,5-trihydroxybenzoic acid | MOL000513 | 31.69 | 370 | LNTHITQWFMADLM-UHFFFAOYSA-N |
| **86** | *Rhizoma Polygoni Cuspidati* (Hu Zhang, RPC) | apigenin | MOL000008 | 23.06 | 5280443 | KZNIFHPLKGYRTM-UHFFFAOYSA-N |
| **87** | *Rhizoma Polygoni Cuspidati* (Hu Zhang, RPC) | beta-sitosterol | MOL000358 | 36.91 | N/A | PRLRGBPWIXOTPG-UIJMBHLFSA-N |
| **88** | *Rhizoma Polygoni Cuspidati* (Hu Zhang, RPC) | citric acid | MOL001456 | 56.22 | 19782904 | KRKNYBCHXYNGOX-UHFFFAOYSA-N |
| **89** | *Rhizoma Polygoni Cuspidati* (Hu Zhang, RPC) | coumarin | MOL000431 | 29.17 | 323 | ZYGHJZDHTFUPRJ-UHFFFAOYSA-N |
| **90** | *Rhizoma Polygoni Cuspidati* (Hu Zhang, RPC) | Crysophanol | MOL001729 | 18.64 | 10208 | LQGUBLBATBMXHT-UHFFFAOYSA-N |
| **91** | *Rhizoma Polygoni Cuspidati* (Hu Zhang, RPC) | emodin | MOL000472 | 24.40 | 3220 | RHMXXJGYXNZAPX-UHFFFAOYSA-N |
| **92** | *Rhizoma Polygoni Cuspidati* (Hu Zhang, RPC) | Hirsutrin | MOL000437 | 1.86 | 5280804 | OVSQVDMCBVZWGM-QSOFNFLRSA-N |
| **93** | *Rhizoma Polygoni Cuspidati* (Hu Zhang, RPC) | luteolin | MOL000006 | 36.16 | 5280445 | IQPNAANSBPBGFQ-UHFFFAOYSA-N |
| **94** | *Rhizoma Polygoni Cuspidati* (Hu Zhang, RPC) | luteolin-7-o-glucoside | MOL000009 | 7.29 | 5280637 | PEFNSGRTCBGNAN-QNDFHXLGSA-N |
| **95** | *Rhizoma Polygoni Cuspidati* (Hu Zhang, RPC) | MLT | MOL001468 | 59.62 | 222656 | BJEPYKJPYRNKOW-REOHCLBHSA-N |
| **96** | *Rhizoma Polygoni Cuspidati* (Hu Zhang, RPC) | oleanolic acid | MOL000263 | 29.02 | 10494 | MIJYXULNPSFWEK-GTOFXWBISA-N |
| **97** | *Rhizoma Polygoni Cuspidati* (Hu Zhang, RPC) | Physcion | MOL000476 | 22.29 | 10639 | FFWOKTFYGVYKIR-UHFFFAOYSA-N |
| **98** | *Rhizoma Polygoni Cuspidati* (Hu Zhang, RPC) | Physovenine | MOL013287 | 106.21 | 442113 | LXTKNVLLWOLCOV-JSGCOSHPSA-N |
| **99** | *Rhizoma Polygoni Cuspidati* (Hu Zhang, RPC) | polydatin | MOL013289 | 21.44 | 5281718 | HSTZMXCBWJGKHG-CUYWLFDKSA-N |
| **100** | *Rhizoma Polygoni Cuspidati* (Hu Zhang, RPC) | protocatechuic acid | MOL000105 | 25.37 | 72 | YQUVCSBJEUQKSH-UHFFFAOYSA-N |
| **101** | *Rhizoma Polygoni Cuspidati* (Hu Zhang, RPC) | quercetin | MOL000098 | 46.43 | 5280343 | REFJWTPEDVJJIY-UHFFFAOYSA-N |
| **102** | *Rhizoma Polygoni Cuspidati* (Hu Zhang, RPC) | resveratrol | MOL012744 | 19.07 | 445154 | LUKBXSAWLPMMSZ-OWOJBTEDSA-N |
| **103** | *Rhizoma Polygoni Cuspidati* (Hu Zhang, RPC) | rhein | MOL002268 | 47.07 | 10168 | FCDLCPWAQCPTKC-UHFFFAOYSA-N |
| **104** | *Rhizoma Polygoni Cuspidati* (Hu Zhang, RPC) | Tar | MOL001886 | 66.38 | 439655 | FEWJPZIEWOKRBE-LWMBPPNESA-N |
| **105** | *Herba Gynostemmatis* (Jiao Gu Lan, HG) | (3S,5R,8R,9R,10R,12R,13R,14R,17S)-17-[(2S)-2-hydroxy-6-methylhept-5-en-2-yl]-4,4,8,10,14-pentamethyl-2,3,5,6,7,9,11,12,13,15,16,17-dodecahydro-1H-cyclopenta[a]phenanthrene-3,12-diol | MOL005334 | 29.69 | 11213350 | PYXFVCFISTUSOO-HKUCOEKDSA-N |
| **106** | *Herba Gynostemmatis* (Jiao Gu Lan, HG) | 3'-methyleriodictyol | MOL000338 | 51.61 | 12310452 | FTODBIPDTXRIGS-CYBMUJFWSA-N |
| **107** | *Herba Gynostemmatis* (Jiao Gu Lan, HG) | 6-o-malonyl-beta-methyl-d-glucopyranoside | MOL009872 | 10.90 | 5319243 | JHZXMTZJWGLQRP-PMZAMSJHSA-N |
| **108** | *Herba Gynostemmatis* (Jiao Gu Lan, HG) | beta-sitosterol | MOL000358 | 36.91 | N/A | PRLRGBPWIXOTPG-UIJMBHLFSA-N |
| **109** | *Herba Gynostemmatis* (Jiao Gu Lan, HG) | caprylic acid | MOL000303 | 16.40 | 379 | WWZKQHOCKIZLMA-UHFFFAOYSA-N |
| **110** | *Herba Gynostemmatis* (Jiao Gu Lan, HG) | CLR | MOL000953 | 37.87 | 5997 | HVYWMOMLDIMFJA-DPAQBDIFSA-N |
| **111** | *Herba Gynostemmatis* (Jiao Gu Lan, HG) | Guasol | MOL002983 | 51.60 | 460 | LHGVFZTZFXWLCP-UHFFFAOYSA-N |
| **112** | *Herba Gynostemmatis* (Jiao Gu Lan, HG) | Gulutamine | MOL000052 | 6.66 | 44272391 | WHUUTDBJXJRKMK-VKHMYHEASA-N |
| **113** | *Herba Gynostemmatis* (Jiao Gu Lan, HG) | Hyacinthin | MOL000714 | 38.65 | 998 | DTUQWGWMVIHBKE-UHFFFAOYSA-N |
| **114** | *Herba Gynostemmatis* (Jiao Gu Lan, HG) | mli | MOL001738 | 21.69 | 23511544 | OFOBLEOULBTSOW-UHFFFAOYSA-N |
| **115** | *Herba Gynostemmatis* (Jiao Gu Lan, HG) | o-Thymol | MOL000259 | 43.28 | 10364 | RECUKUPTGUEGMW-UHFFFAOYSA-N |
| **116** | *Herba Gynostemmatis* (Jiao Gu Lan, HG) | o-xylene | MOL001097 | 45.55 | 7237 | CTQNGGLPUBDAKN-UHFFFAOYSA-N |
| **117** | *Herba Gynostemmatis* (Jiao Gu Lan, HG) | quercetin | MOL000098 | 46.43 | 5280343 | REFJWTPEDVJJIY-UHFFFAOYSA-N |
| **118** | *Herba Gynostemmatis* (Jiao Gu Lan, HG) | rutin | MOL000415 | 3.20 | 5280805 | IKGXIBQEEMLURG-NVPNHPEKSA-N |
| **119** | *Herba Gynostemmatis* (Jiao Gu Lan, HG) | sucrose | MOL000842 | 7.17 | 5988 | CZMRCDWAGMRECN-UGDNZRGBSA-N |

**Supplementary Table S2. Potential Targets of each ingredient in JZG**

| **NO** | **Herbname** | **Candidate Compound Name** | **Uniprot ID** | **Target Gene Name** |
| --- | --- | --- | --- | --- |
| **1** | *Herba Artemisiae Scopariae* | beta-elemene | P42574 | CASP3 |
| **2** | *Herba Artemisiae Scopariae* | Butal | P15121 | AKR1B1 |
| **3** | *Herba Artemisiae Scopariae* | caffeic acid | P28482 | MAPK1 |
| **4** | *Herba Artemisiae Scopariae* | caffeic acid | P08253 | MMP2 |
| **5** | *Herba Artemisiae Scopariae* | caffeic acid | P05164 | MPO |
| **6** | *Herba Artemisiae Scopariae* | caffeic acid | P04040 | CAT |
| **7** | *Herba Artemisiae Scopariae* | caffeic acid | P14679 | TYR |
| **8** | *Herba Artemisiae Scopariae* | caffeic acid | P50225 | SULT1A1 |
| **9** | *Herba Artemisiae Scopariae* | caffeic acid | P03956 | MMP1 |
| **10** | *Herba Artemisiae Scopariae* | caffeic acid | Q9HAW8 | UGT1A10 |
| **11** | *Herba Artemisiae Scopariae* | caffeic acid | P45983 | MAPK8 |
| **12** | *Herba Artemisiae Scopariae* | caffeic acid | P14780 | MMP9 |
| **13** | *Herba Artemisiae Scopariae* | caffeic acid | Q9HAW7 | UGT1A7 |
| **14** | *Herba Artemisiae Scopariae* | caffeic acid | Q9HAW9 | UGT1A8 |
| **15** | *Herba Artemisiae Scopariae* | caffeic acid | P09917 | ALOX5 |
| **16** | *Herba Artemisiae Scopariae* | caffeic acid | P35503 | UGT1A3 |
| **17** | *Herba Artemisiae Scopariae* | capillarisin | P14780 | MMP9 |
| **18** | *Herba Artemisiae Scopariae* | caproic acid | P46663 | BDKRB1 |
| **19** | *Herba Artemisiae Scopariae* | caproic acid | Q99705 | MCHR1 |
| **20** | *Herba Artemisiae Scopariae* | caproic acid | Q9GZQ4 | NMUR2 |
| **21** | *Herba Artemisiae Scopariae* | caproic acid | P04083 | ANXA1 |
| **22** | *Herba Artemisiae Scopariae* | caproic acid | P42336 | PIK3CA |
| **23** | *Herba Artemisiae Scopariae* | caproic acid | P48645 | NMU |
| **24** | *Herba Artemisiae Scopariae* | caproic acid | P01042 | KNG1 |
| **25** | *Herba Artemisiae Scopariae* | caproic acid | Q13304 | GPR17 |
| **26** | *Herba Artemisiae Scopariae* | caproic acid | Q969V1 | MCHR2 |
| **27** | *Herba Artemisiae Scopariae* | caproic acid | O60235 | TMPRSS11D |
| **28** | *Herba Artemisiae Scopariae* | caproic acid | P05067 | APP |
| **29** | *Herba Artemisiae Scopariae* | caproic acid | P15121 | AKR1B1 |
| **30** | *Herba Artemisiae Scopariae* | caproic acid | Q9UNI1 | CELA1 |
| **31** | *Herba Artemisiae Scopariae* | caproic acid | Q9HB89 | NMUR1 |
| **32** | *Herba Artemisiae Scopariae* | caproic acid | Q9H1C0 | LPAR5 |
| **33** | *Herba Artemisiae Scopariae* | caproic acid | P20382 | PMCH |
| **34** | *Herba Artemisiae Scopariae* | caproic acid | P49795 | RGS19 |
| **35** | *Herba Artemisiae Scopariae* | caproic acid | P59768 | GNG2 |
| **36** | *Herba Artemisiae Scopariae* | caproic acid | P25090 | FPR2 |
| **37** | *Herba Artemisiae Scopariae* | caproic acid | P0DJI8 | SAA1 |
| **38** | *Herba Artemisiae Scopariae* | caproic acid | Q92633 | LPAR1 |
| **39** | *Herba Artemisiae Scopariae* | caproic acid | P01019 | AGT |
| **40** | *Herba Artemisiae Scopariae* | caproic acid | Q9NS28 | RGS18 |
| **41** | *Herba Artemisiae Scopariae* | caproic acid | Q9UBY5 | LPAR3 |
| **42** | *Herba Artemisiae Scopariae* | caproic acid | Q5H8A3 | NMS |
| **43** | *Herba Artemisiae Scopariae* | caproic acid | P05305 | EDN1 |
| **44** | *Herba Artemisiae Scopariae* | caproic acid | P01185 | AVP |
| **45** | *Herba Artemisiae Scopariae* | caproic acid | Q9HBW0 | LPAR2 |
| **46** | *Herba Artemisiae Scopariae* | caproic acid | P01275 | GCG |
| **47** | *Herba Artemisiae Scopariae* | caproic acid | P21731 | TBXA2R |
| **48** | *Herba Artemisiae Scopariae* | d-limonene | P11712 | CYP2C9 |
| **49** | *Herba Artemisiae Scopariae* | d-limonene | Q9UBK8 | MTRR |
| **50** | *Herba Artemisiae Scopariae* | d-limonene | P37231 | PPARG |
| **51** | *Herba Artemisiae Scopariae* | d-limonene | P29474 | NOS3 |
| **52** | *Herba Artemisiae Scopariae* | d-limonene | P35228 | NOS2 |
| **53** | *Herba Artemisiae Scopariae* | d-limonene | P33261 | CYP2C19 |
| **54** | *Herba Artemisiae Scopariae* | d-limonene | P16435 | POR |
| **55** | *Herba Artemisiae Scopariae* | eugenol | O60235 | TMPRSS11D |
| **56** | *Herba Artemisiae Scopariae* | eugenol | P22309 | UGT1A1 |
| **57** | *Herba Artemisiae Scopariae* | eugenol | P42574 | CASP3 |
| **58** | *Herba Artemisiae Scopariae* | eugenol | P15559 | NQO1 |
| **59** | *Herba Artemisiae Scopariae* | eugenol | O75795 | UGT2B17 |
| **60** | *Herba Artemisiae Scopariae* | eugenol | P55211 | CASP9 |
| **61** | *Herba Artemisiae Scopariae* | eugenol | P21397 | MAOA |
| **62** | *Herba Artemisiae Scopariae* | eugenol | P54855 | UGT2B15 |
| **63** | *Herba Artemisiae Scopariae* | eugenol | Q9HAW8 | UGT1A10 |
| **64** | *Herba Artemisiae Scopariae* | eugenol | O60656 | UGT1A9 |
| **65** | *Herba Artemisiae Scopariae* | eugenol | P10635 | CYP2D6 |
| **66** | *Herba Artemisiae Scopariae* | eugenol | Q9HAW7 | UGT1A7 |
| **67** | *Herba Artemisiae Scopariae* | eugenol | Q9HAW9 | UGT1A8 |
| **68** | *Herba Artemisiae Scopariae* | eugenol | P09917 | ALOX5 |
| **69** | *Herba Artemisiae Scopariae* | eugenol | P04798 | CYP1A1 |
| **70** | *Herba Artemisiae Scopariae* | Furol | Q16698 | DECR1 |
| **71** | *Herba Artemisiae Scopariae* | Furol | P30838 | ALDH3A1 |
| **72** | *Herba Artemisiae Scopariae* | Furol | P51648 | ALDH3A2 |
| **73** | *Herba Artemisiae Scopariae* | Genkwanin | P05177 | CYP1A2 |
| **74** | *Herba Artemisiae Scopariae* | Genkwanin | P04798 | CYP1A1 |
| **75** | *Herba Artemisiae Scopariae* | Hirsutrin | P42574 | CASP3 |
| **76** | *Herba Artemisiae Scopariae* | Hirsutrin | P04798 | CYP1A1 |
| **77** | *Herba Artemisiae Scopariae* | Hirsutrin | P18825 | ADRA2C |
| **78** | *Herba Artemisiae Scopariae* | isorhamnetin | P09601 | HMOX1 |
| **79** | *Herba Artemisiae Scopariae* | isorhamnetin | Q9UBK8 | MTRR |
| **80** | *Herba Artemisiae Scopariae* | isorhamnetin | P08183 | ABCB1 |
| **81** | *Herba Artemisiae Scopariae* | isorhamnetin | P31749 | AKT1 |
| **82** | *Herba Artemisiae Scopariae* | isorhamnetin | P29474 | NOS3 |
| **83** | *Herba Artemisiae Scopariae* | isorhamnetin | P35228 | NOS2 |
| **84** | *Herba Artemisiae Scopariae* | isorhamnetin | P45983 | MAPK8 |
| **85** | *Herba Artemisiae Scopariae* | isorhamnetin | P04798 | CYP1A1 |
| **86** | *Herba Artemisiae Scopariae* | isorhamnetin | P33527 | ABCC1 |
| **87** | *Herba Artemisiae Scopariae* | isorhamnetin | P16435 | POR |
| **88** | *Herba Artemisiae Scopariae* | isorhamnetin | Q04206 | RELA |
| **89** | *Herba Artemisiae Scopariae* | isorhamnetin | P47989 | XDH |
| **90** | *Herba Artemisiae Scopariae* | methyleugenol | P09211 | GSTP1 |
| **91** | *Herba Artemisiae Scopariae* | myristicin | O60760 | HPGDS |
| **92** | *Herba Artemisiae Scopariae* | myristicin | P42574 | CASP3 |
| **93** | *Herba Artemisiae Scopariae* | myristicin | P05177 | CYP1A2 |
| **94** | *Herba Artemisiae Scopariae* | myristicin | P09874 | PARP1 |
| **95** | *Herba Artemisiae Scopariae* | myristicin | P04798 | CYP1A1 |
| **96** | *Herba Artemisiae Scopariae* | oleovitamin a | P01730 | CD4 |
| **97** | *Herba Artemisiae Scopariae* | oleovitamin a | P54136 | RARS |
| **98** | *Herba Artemisiae Scopariae* | oleovitamin a | P04114 | APOB |
| **99** | *Herba Artemisiae Scopariae* | oleovitamin a | P04040 | CAT |
| **100** | *Herba Artemisiae Scopariae* | oleovitamin a | O94788 | ALDH1A2 |
| **101** | *Herba Artemisiae Scopariae* | oleovitamin a | P02649 | APOE |
| **102** | *Herba Artemisiae Scopariae* | oleovitamin a | Q8WTV0 | SCARB1 |
| **103** | *Herba Artemisiae Scopariae* | oleovitamin a | P14679 | TYR |
| **104** | *Herba Artemisiae Scopariae* | oleovitamin a | O60235 | TMPRSS11D |
| **105** | *Herba Artemisiae Scopariae* | oleovitamin a | P15121 | AKR1B1 |
| **106** | *Herba Artemisiae Scopariae* | oleovitamin a | P02768 | ALB |
| **107** | *Herba Artemisiae Scopariae* | oleovitamin a | P00352 | ALDH1A1 |
| **108** | *Herba Artemisiae Scopariae* | oleovitamin a | P42574 | CASP3 |
| **109** | *Herba Artemisiae Scopariae* | oleovitamin a | P03956 | MMP1 |
| **110** | *Herba Artemisiae Scopariae* | oleovitamin a | P47895 | ALDH1A3 |
| **111** | *Herba Artemisiae Scopariae* | oleovitamin a | P35222 | CTNNB1 |
| **112** | *Herba Artemisiae Scopariae* | oleovitamin a | P51648 | ALDH3A2 |
| **113** | *Herba Artemisiae Scopariae* | oleovitamin a | P02751 | FN1 |
| **114** | *Herba Artemisiae Scopariae* | oleovitamin a | O60218 | AKR1B10 |
| **115** | *Herba Artemisiae Scopariae* | oleovitamin a | P08195 | SLC3A2 |
| **116** | *Herba Artemisiae Scopariae* | oleovitamin a | P05305 | EDN1 |
| **117** | *Herba Artemisiae Scopariae* | oleovitamin a | P07101 | TH |
| **118** | *Herba Artemisiae Scopariae* | oleovitamin a | P23560 | BDNF |
| **119** | *Herba Artemisiae Scopariae* | p-cresol | P05121 | SERPINE1 |
| **120** | *Herba Artemisiae Scopariae* | p-cresol | P05181 | CYP2E1 |
| **121** | *Herba Artemisiae Scopariae* | p-cresol | P22303 | ACHE |
| **122** | *Herba Artemisiae Scopariae* | p-cresol | P42574 | CASP3 |
| **123** | *Herba Artemisiae Scopariae* | p-cresol | P05177 | CYP1A2 |
| **124** | *Herba Artemisiae Scopariae* | p-cresol | Q14790 | CASP8 |
| **125** | *Herba Artemisiae Scopariae* | p-cresol | P10635 | CYP2D6 |
| **126** | *Herba Artemisiae Scopariae* | p-cresol | P05412 | JUN |
| **127** | *Herba Artemisiae Scopariae* | p-cresol | P23560 | BDNF |
| **128** | *Herba Artemisiae Scopariae* | quercetin | P13569 | CFTR |
| **129** | *Herba Artemisiae Scopariae* | quercetin | P21917 | DRD4 |
| **130** | *Herba Artemisiae Scopariae* | quercetin | P03372 | ESR1 |
| **131** | *Herba Artemisiae Scopariae* | quercetin | Q96EB6 | SIRT1 |
| **132** | *Herba Artemisiae Scopariae* | quercetin | P05546 | SERPIND1 |
| **133** | *Herba Artemisiae Scopariae* | quercetin | P28482 | MAPK1 |
| **134** | *Herba Artemisiae Scopariae* | quercetin | P09601 | HMOX1 |
| **135** | *Herba Artemisiae Scopariae* | quercetin | P25963 | NFKBIA |
| **136** | *Herba Artemisiae Scopariae* | quercetin | P08253 | MMP2 |
| **137** | *Herba Artemisiae Scopariae* | quercetin | P00750 | PLAT |
| **138** | *Herba Artemisiae Scopariae* | quercetin | P00390 | GSR |
| **139** | *Herba Artemisiae Scopariae* | quercetin | P78329 | CYP4F2 |
| **140** | *Herba Artemisiae Scopariae* | quercetin | P27169 | PON1 |
| **141** | *Herba Artemisiae Scopariae* | quercetin | P43490 | NAMPT |
| **142** | *Herba Artemisiae Scopariae* | quercetin | P05164 | MPO |
| **143** | *Herba Artemisiae Scopariae* | quercetin | P13500 | CCL2 |
| **144** | *Herba Artemisiae Scopariae* | quercetin | Q16539 | MAPK14 |
| **145** | *Herba Artemisiae Scopariae* | quercetin | P04114 | APOB |
| **146** | *Herba Artemisiae Scopariae* | quercetin | Q9UNQ0 | ABCG2 |
| **147** | *Herba Artemisiae Scopariae* | quercetin | P04040 | CAT |
| **148** | *Herba Artemisiae Scopariae* | quercetin | P35869 | AHR |
| **149** | *Herba Artemisiae Scopariae* | quercetin | P38936 | CDKN1A |
| **150** | *Herba Artemisiae Scopariae* | quercetin | P18054 | ALOX12 |
| **151** | *Herba Artemisiae Scopariae* | quercetin | P05181 | CYP2E1 |
| **152** | *Herba Artemisiae Scopariae* | quercetin | P01116 | KRAS |
| **153** | *Herba Artemisiae Scopariae* | quercetin | P21589 | NT5E |
| **154** | *Herba Artemisiae Scopariae* | quercetin | P05231 | IL6 |
| **155** | *Herba Artemisiae Scopariae* | quercetin | O60603 | TLR2 |
| **156** | *Herba Artemisiae Scopariae* | quercetin | P11712 | CYP2C9 |
| **157** | *Herba Artemisiae Scopariae* | quercetin | Q07869 | PPARA |
| **158** | *Herba Artemisiae Scopariae* | quercetin | P27361 | MAPK3 |
| **159** | *Herba Artemisiae Scopariae* | quercetin | P14679 | TYR |
| **160** | *Herba Artemisiae Scopariae* | quercetin | P01584 | IL1B |
| **161** | *Herba Artemisiae Scopariae* | quercetin | P42336 | PIK3CA |
| **162** | *Herba Artemisiae Scopariae* | quercetin | P06276 | BCHE |
| **163** | *Herba Artemisiae Scopariae* | quercetin | P05362 | ICAM1 |
| **164** | *Herba Artemisiae Scopariae* | quercetin | P36537 | UGT2B10 |
| **165** | *Herba Artemisiae Scopariae* | quercetin | P08183 | ABCB1 |
| **166** | *Herba Artemisiae Scopariae* | quercetin | Q14534 | SQLE |
| **167** | *Herba Artemisiae Scopariae* | quercetin | P24941 | CDK2 |
| **168** | *Herba Artemisiae Scopariae* | quercetin | P06400 | RB1 |
| **169** | *Herba Artemisiae Scopariae* | quercetin | Q16548 | BCL2A1 |
| **170** | *Herba Artemisiae Scopariae* | quercetin | P08069 | IGF1R |
| **171** | *Herba Artemisiae Scopariae* | quercetin | P04637 | TP53 |
| **172** | *Herba Artemisiae Scopariae* | quercetin | P31749 | AKT1 |
| **173** | *Herba Artemisiae Scopariae* | quercetin | P00533 | EGFR |
| **174** | *Herba Artemisiae Scopariae* | quercetin | O14763 | TNFRSF10B |
| **175** | *Herba Artemisiae Scopariae* | quercetin | O60235 | TMPRSS11D |
| **176** | *Herba Artemisiae Scopariae* | quercetin | P15121 | AKR1B1 |
| **177** | *Herba Artemisiae Scopariae* | quercetin | Q9UHC9 | NPC1L1 |
| **178** | *Herba Artemisiae Scopariae* | quercetin | Q07812 | BAX |
| **179** | *Herba Artemisiae Scopariae* | quercetin | O60760 | HPGDS |
| **180** | *Herba Artemisiae Scopariae* | quercetin | P04141 | CSF2 |
| **181** | *Herba Artemisiae Scopariae* | quercetin | P29474 | NOS3 |
| **182** | *Herba Artemisiae Scopariae* | quercetin | Q9H2F3 | HSD3B7 |
| **183** | *Herba Artemisiae Scopariae* | quercetin | Q07817 | BCL2L1 |
| **184** | *Herba Artemisiae Scopariae* | quercetin | P34932 | HSPA4 |
| **185** | *Herba Artemisiae Scopariae* | quercetin | P19224 | UGT1A6 |
| **186** | *Herba Artemisiae Scopariae* | quercetin | Q8N4T8 | CBR4 |
| **187** | *Herba Artemisiae Scopariae* | quercetin | P22309 | UGT1A1 |
| **188** | *Herba Artemisiae Scopariae* | quercetin | P35568 | IRS1 |
| **189** | *Herba Artemisiae Scopariae* | quercetin | P02778 | CXCL10 |
| **190** | *Herba Artemisiae Scopariae* | quercetin | P01100 | FOS |
| **191** | *Herba Artemisiae Scopariae* | quercetin | P10145 | CXCL8 |
| **192** | *Herba Artemisiae Scopariae* | quercetin | P99999 | CYCS |
| **193** | *Herba Artemisiae Scopariae* | quercetin | Q9BZR8 | BCL2L14 |
| **194** | *Herba Artemisiae Scopariae* | quercetin | P42574 | CASP3 |
| **195** | *Herba Artemisiae Scopariae* | quercetin | Q9UMX3 | BOK |
| **196** | *Herba Artemisiae Scopariae* | quercetin | P15559 | NQO1 |
| **197** | *Herba Artemisiae Scopariae* | quercetin | O75795 | UGT2B17 |
| **198** | *Herba Artemisiae Scopariae* | quercetin | Q15848 | ADIPOQ |
| **199** | *Herba Artemisiae Scopariae* | quercetin | P14672 | SLC2A4 |
| **200** | *Herba Artemisiae Scopariae* | quercetin | P50225 | SULT1A1 |
| **201** | *Herba Artemisiae Scopariae* | quercetin | P03956 | MMP1 |
| **202** | *Herba Artemisiae Scopariae* | quercetin | P49841 | GSK3B |
| **203** | *Herba Artemisiae Scopariae* | quercetin | P35228 | NOS2 |
| **204** | *Herba Artemisiae Scopariae* | quercetin | P10415 | BCL2 |
| **205** | *Herba Artemisiae Scopariae* | quercetin | P55211 | CASP9 |
| **206** | *Herba Artemisiae Scopariae* | quercetin | P48357 | LEPR |
| **207** | *Herba Artemisiae Scopariae* | quercetin | O75469 | NR1I2 |
| **208** | *Herba Artemisiae Scopariae* | quercetin | P08684 | CYP3A4 |
| **209** | *Herba Artemisiae Scopariae* | quercetin | Q16665 | HIF1A |
| **210** | *Herba Artemisiae Scopariae* | quercetin | P21397 | MAOA |
| **211** | *Herba Artemisiae Scopariae* | quercetin | P54855 | UGT2B15 |
| **212** | *Herba Artemisiae Scopariae* | quercetin | Q05397 | PTK2 |
| **213** | *Herba Artemisiae Scopariae* | quercetin | P05177 | CYP1A2 |
| **214** | *Herba Artemisiae Scopariae* | quercetin | Q9HAW8 | UGT1A10 |
| **215** | *Herba Artemisiae Scopariae* | quercetin | Q92731 | ESR2 |
| **216** | *Herba Artemisiae Scopariae* | quercetin | P35222 | CTNNB1 |
| **217** | *Herba Artemisiae Scopariae* | quercetin | O60656 | UGT1A9 |
| **218** | *Herba Artemisiae Scopariae* | quercetin | P12931 | SRC |
| **219** | *Herba Artemisiae Scopariae* | quercetin | Q14790 | CASP8 |
| **220** | *Herba Artemisiae Scopariae* | quercetin | P27540 | ARNT |
| **221** | *Herba Artemisiae Scopariae* | quercetin | P45983 | MAPK8 |
| **222** | *Herba Artemisiae Scopariae* | quercetin | P10635 | CYP2D6 |
| **223** | *Herba Artemisiae Scopariae* | quercetin | P42345 | MTOR |
| **224** | *Herba Artemisiae Scopariae* | quercetin | P23219 | PTGS1 |
| **225** | *Herba Artemisiae Scopariae* | quercetin | P25929 | NPY1R |
| **226** | *Herba Artemisiae Scopariae* | quercetin | P09874 | PARP1 |
| **227** | *Herba Artemisiae Scopariae* | quercetin | Q14994 | NR1I3 |
| **228** | *Herba Artemisiae Scopariae* | quercetin | Q07820 | MCL1 |
| **229** | *Herba Artemisiae Scopariae* | quercetin | P55210 | CASP7 |
| **230** | *Herba Artemisiae Scopariae* | quercetin | P14060 | HSD3B1 |
| **231** | *Herba Artemisiae Scopariae* | quercetin | P26439 | HSD3B2 |
| **232** | *Herba Artemisiae Scopariae* | quercetin | O15111 | CHUK |
| **233** | *Herba Artemisiae Scopariae* | quercetin | P05412 | JUN |
| **234** | *Herba Artemisiae Scopariae* | quercetin | P14780 | MMP9 |
| **235** | *Herba Artemisiae Scopariae* | quercetin | P00749 | PLAU |
| **236** | *Herba Artemisiae Scopariae* | quercetin | Q9HAW7 | UGT1A7 |
| **237** | *Herba Artemisiae Scopariae* | quercetin | Q9HAW9 | UGT1A8 |
| **238** | *Herba Artemisiae Scopariae* | quercetin | O00206 | TLR4 |
| **239** | *Herba Artemisiae Scopariae* | quercetin | P09917 | ALOX5 |
| **240** | *Herba Artemisiae Scopariae* | quercetin | P10275 | AR |
| **241** | *Herba Artemisiae Scopariae* | quercetin | O95477 | ABCA1 |
| **242** | *Herba Artemisiae Scopariae* | quercetin | P27338 | MAOB |
| **243** | *Herba Artemisiae Scopariae* | quercetin | P05305 | EDN1 |
| **244** | *Herba Artemisiae Scopariae* | quercetin | P47989 | XDH |
| **245** | *Herba Artemisiae Scopariae* | quercetin | P04798 | CYP1A1 |
| **246** | *Herba Artemisiae Scopariae* | quercetin | P42330 | AKR1C3 |
| **247** | *Herba Artemisiae Scopariae* | quercetin | O60674 | JAK2 |
| **248** | *Herba Artemisiae Scopariae* | quercetin | P06493 | CDK1 |
| **249** | *Herba Artemisiae Scopariae* | quercetin | Q16236 | NFE2L2 |
| **250** | *Herba Artemisiae Scopariae* | quercetin | P09211 | GSTP1 |
| **251** | *Herba Artemisiae Scopariae* | quercetin | P33527 | ABCC1 |
| **252** | *Herba Artemisiae Scopariae* | quercetin | P61073 | CXCR4 |
| **253** | *Herba Artemisiae Scopariae* | quercetin | O75310 | UGT2B11 |
| **254** | *Herba Artemisiae Scopariae* | quercetin | Q02880 | TOP2B |
| **255** | *Herba Artemisiae Scopariae* | quercetin | P01375 | TNF |
| **256** | *Herba Artemisiae Scopariae* | quercetin | Q13630 | TSTA3 |
| **257** | *Herba Artemisiae Scopariae* | quercetin | P07203 | GPX1 |
| **258** | *Herba Artemisiae Scopariae* | quercetin | P11388 | TOP2A |
| **259** | *Herba Artemisiae Scopariae* | quercetin | P23560 | BDNF |
| **260** | *Herba Artemisiae Scopariae* | quercetin | P11166 | SLC2A1 |
| **261** | *Herba Artemisiae Scopariae* | quercetin | P35503 | UGT1A3 |
| **262** | *Herba Artemisiae Scopariae* | quercetin | P16435 | POR |
| **263** | *Herba Artemisiae Scopariae* | quercetin | P40313 | CTRL |
| **264** | *Herba Artemisiae Scopariae* | rutin | P28482 | MAPK1 |
| **265** | *Herba Artemisiae Scopariae* | rutin | P00390 | GSR |
| **266** | *Herba Artemisiae Scopariae* | rutin | P13500 | CCL2 |
| **267** | *Herba Artemisiae Scopariae* | rutin | P04040 | CAT |
| **268** | *Herba Artemisiae Scopariae* | rutin | P35869 | AHR |
| **269** | *Herba Artemisiae Scopariae* | rutin | P05091 | ALDH2 |
| **270** | *Herba Artemisiae Scopariae* | rutin | P27361 | MAPK3 |
| **271** | *Herba Artemisiae Scopariae* | rutin | Q9UBK8 | MTRR |
| **272** | *Herba Artemisiae Scopariae* | rutin | P00533 | EGFR |
| **273** | *Herba Artemisiae Scopariae* | rutin | P08913 | ADRA2A |
| **274** | *Herba Artemisiae Scopariae* | rutin | O60235 | TMPRSS11D |
| **275** | *Herba Artemisiae Scopariae* | rutin | P29474 | NOS3 |
| **276** | *Herba Artemisiae Scopariae* | rutin | P34932 | HSPA4 |
| **277** | *Herba Artemisiae Scopariae* | rutin | P22303 | ACHE |
| **278** | *Herba Artemisiae Scopariae* | rutin | P02778 | CXCL10 |
| **279** | *Herba Artemisiae Scopariae* | rutin | P10145 | CXCL8 |
| **280** | *Herba Artemisiae Scopariae* | rutin | P42574 | CASP3 |
| **281** | *Herba Artemisiae Scopariae* | rutin | P35228 | NOS2 |
| **282** | *Herba Artemisiae Scopariae* | rutin | P07237 | P4HB |
| **283** | *Herba Artemisiae Scopariae* | rutin | P36956 | SREBF1 |
| **284** | *Herba Artemisiae Scopariae* | rutin | P27540 | ARNT |
| **285** | *Herba Artemisiae Scopariae* | rutin | P55210 | CASP7 |
| **286** | *Herba Artemisiae Scopariae* | rutin | P42330 | AKR1C3 |
| **287** | *Herba Artemisiae Scopariae* | rutin | P18825 | ADRA2C |
| **288** | *Herba Artemisiae Scopariae* | rutin | P16435 | POR |
| **289** | *Herba Artemisiae Scopariae* | salicylic acid | P05164 | MPO |
| **290** | *Herba Artemisiae Scopariae* | salicylic acid | P38936 | CDKN1A |
| **291** | *Herba Artemisiae Scopariae* | salicylic acid | P18054 | ALOX12 |
| **292** | *Herba Artemisiae Scopariae* | salicylic acid | P05181 | CYP2E1 |
| **293** | *Herba Artemisiae Scopariae* | salicylic acid | Q8WTV0 | SCARB1 |
| **294** | *Herba Artemisiae Scopariae* | salicylic acid | P04637 | TP53 |
| **295** | *Herba Artemisiae Scopariae* | salicylic acid | O60235 | TMPRSS11D |
| **296** | *Herba Artemisiae Scopariae* | salicylic acid | P02768 | ALB |
| **297** | *Herba Artemisiae Scopariae* | salicylic acid | P99999 | CYCS |
| **298** | *Herba Artemisiae Scopariae* | salicylic acid | P42574 | CASP3 |
| **299** | *Herba Artemisiae Scopariae* | salicylic acid | P48357 | LEPR |
| **300** | *Herba Artemisiae Scopariae* | salicylic acid | Q16665 | HIF1A |
| **301** | *Herba Artemisiae Scopariae* | salicylic acid | Q9HAW8 | UGT1A10 |
| **302** | *Herba Artemisiae Scopariae* | salicylic acid | P23219 | PTGS1 |
| **303** | *Herba Artemisiae Scopariae* | salicylic acid | P09874 | PARP1 |
| **304** | *Herba Artemisiae Scopariae* | salicylic acid | Q9HAW7 | UGT1A7 |
| **305** | *Herba Artemisiae Scopariae* | salicylic acid | Q9HAW9 | UGT1A8 |
| **306** | *Herba Artemisiae Scopariae* | salicylic acid | P47989 | XDH |
| **307** | *Herba Artemisiae Scopariae* | salicylic acid | P42330 | AKR1C3 |
| **308** | *Herba Artemisiae Scopariae* | salicylic acid | Q96BT7 | ALKBH8 |
| **309** | *Herba Artemisiae Scopariae* | salicylic acid | O43709 | WBSCR22 |
| **310** | *Herba Artemisiae Scopariae* | salicylic acid | P35503 | UGT1A3 |
| **311** | *Herba Artemisiae Scopariae* | salicylic acid | P09917 | ALOX5 |
| **312** | *Herba Artemisiae Scopariae* | salicylic acid | P27169 | PON1 |
| **313** | *Herba Artemisiae Scopariae* | salicylic acid | P05305 | EDN1 |
| **314** | *Herba Artemisiae Scopariae* | salicylic acid | P04040 | CAT |
| **315** | *Herba Artemisiae Scopariae* | salicylic acid | P00441 | SOD1 |
| **316** | *Herba Artemisiae Scopariae* | salicylic acid | P17735 | TAT |
| **317** | *Herba Artemisiae Scopariae* | salicylic acid | P09211 | GSTP1 |
| **318** | *Herba Artemisiae Scopariae* | salicylic acid | Q04206 | RELA |
| **319** | *Herba Artemisiae Scopariae* | scoparone | Q16698 | DECR1 |
| **320** | *Herba Artemisiae Scopariae* | scoparone | Q9UBK8 | MTRR |
| **321** | *Herba Artemisiae Scopariae* | scoparone | P29474 | NOS3 |
| **322** | *Herba Artemisiae Scopariae* | scoparone | P35228 | NOS2 |
| **323** | *Herba Artemisiae Scopariae* | scoparone | P16435 | POR |
| **324** | *Herba Artemisiae Scopariae* | scoparone | P14679 | TYR |
| **325** | *Herba Artemisiae Scopariae* | scoparone | Q04206 | RELA |
| **326** | *Herba Artemisiae Scopariae* | scoparone | P25963 | NFKBIA |
| **327** | *Herba Artemisiae Scopariae* | scoparone | P10145 | CXCL8 |
| **328** | *Herba Artemisiae Scopariae* | scoparone | P13500 | CCL2 |
| **329** | *Herba Artemisiae Scopariae* | Scopoletol | P04040 | CAT |
| **330** | *Herba Artemisiae Scopariae* | Scopoletol | P19224 | UGT1A6 |
| **331** | *Herba Artemisiae Scopariae* | Scopoletol | P22309 | UGT1A1 |
| **332** | *Herba Artemisiae Scopariae* | Scopoletol | P42574 | CASP3 |
| **333** | *Herba Artemisiae Scopariae* | Scopoletol | P54855 | UGT2B15 |
| **334** | *Herba Artemisiae Scopariae* | Scopoletol | Q9HAW8 | UGT1A10 |
| **335** | *Herba Artemisiae Scopariae* | Scopoletol | O60656 | UGT1A9 |
| **336** | *Herba Artemisiae Scopariae* | Scopoletol | Q9HAW7 | UGT1A7 |
| **337** | *Herba Artemisiae Scopariae* | Scopoletol | Q9HAW9 | UGT1A8 |
| **338** | *Herba Artemisiae Scopariae* | Scopoletol | P35503 | UGT1A3 |
| **339** | *Herba Artemisiae Scopariae* | vanillin | Q9HAW8 | UGT1A10 |
| **340** | *Herba Artemisiae Scopariae* | vanillin | P14780 | MMP9 |
| **341** | *Herba Artemisiae Scopariae* | vanillin | Q9HAW7 | UGT1A7 |
| **342** | *Herba Artemisiae Scopariae* | vanillin | Q9HAW9 | UGT1A8 |
| **343** | *Herba Artemisiae Scopariae* | vanillin | P35503 | UGT1A3 |
| **344** | *Radix Salviae* | (2S)-3-(3,4-dihydroxyphenyl)-2-hydroxypropanoic acid | P09601 | HMOX1 |
| **345** | *Radix Salviae* | (2S)-3-(3,4-dihydroxyphenyl)-2-hydroxypropanoic acid | P00749 | PLAU |
| **346** | *Radix Salviae* | apigenin | P13569 | CFTR |
| **347** | *Radix Salviae* | apigenin | P03372 | ESR1 |
| **348** | *Radix Salviae* | apigenin | P28482 | MAPK1 |
| **349** | *Radix Salviae* | apigenin | P24385 | CCND1 |
| **350** | *Radix Salviae* | apigenin | P04040 | CAT |
| **351** | *Radix Salviae* | apigenin | P27361 | MAPK3 |
| **352** | *Radix Salviae* | apigenin | P01189 | POMC |
| **353** | *Radix Salviae* | apigenin | P01133 | EGF |
| **354** | *Radix Salviae* | apigenin | P08183 | ABCB1 |
| **355** | *Radix Salviae* | apigenin | Q14534 | SQLE |
| **356** | *Radix Salviae* | apigenin | P04637 | TP53 |
| **357** | *Radix Salviae* | apigenin | P31749 | AKT1 |
| **358** | *Radix Salviae* | apigenin | O14763 | TNFRSF10B |
| **359** | *Radix Salviae* | apigenin | P19224 | UGT1A6 |
| **360** | *Radix Salviae* | apigenin | P22303 | ACHE |
| **361** | *Radix Salviae* | apigenin | P22309 | UGT1A1 |
| **362** | *Radix Salviae* | apigenin | P42574 | CASP3 |
| **363** | *Radix Salviae* | apigenin | P50225 | SULT1A1 |
| **364** | *Radix Salviae* | apigenin | P10415 | BCL2 |
| **365** | *Radix Salviae* | apigenin | P55211 | CASP9 |
| **366** | *Radix Salviae* | apigenin | O75469 | NR1I2 |
| **367** | *Radix Salviae* | apigenin | P08684 | CYP3A4 |
| **368** | *Radix Salviae* | apigenin | Q16665 | HIF1A |
| **369** | *Radix Salviae* | apigenin | P21397 | MAOA |
| **370** | *Radix Salviae* | apigenin | P54855 | UGT2B15 |
| **371** | *Radix Salviae* | apigenin | Q05397 | PTK2 |
| **372** | *Radix Salviae* | apigenin | P05177 | CYP1A2 |
| **373** | *Radix Salviae* | apigenin | Q9HAW8 | UGT1A10 |
| **374** | *Radix Salviae* | apigenin | Q92731 | ESR2 |
| **375** | *Radix Salviae* | apigenin | O60656 | UGT1A9 |
| **376** | *Radix Salviae* | apigenin | Q42521 | GAD1 |
| **377** | *Radix Salviae* | apigenin | P12931 | SRC |
| **378** | *Radix Salviae* | apigenin | Q14790 | CASP8 |
| **379** | *Radix Salviae* | apigenin | P09874 | PARP1 |
| **380** | *Radix Salviae* | apigenin | P55210 | CASP7 |
| **381** | *Radix Salviae* | apigenin | P14780 | MMP9 |
| **382** | *Radix Salviae* | apigenin | Q9HAW7 | UGT1A7 |
| **383** | *Radix Salviae* | apigenin | Q9HAW9 | UGT1A8 |
| **384** | *Radix Salviae* | apigenin | P27338 | MAOB |
| **385** | *Radix Salviae* | apigenin | P04798 | CYP1A1 |
| **386** | *Radix Salviae* | apigenin | P06493 | CDK1 |
| **387** | *Radix Salviae* | apigenin | P33527 | ABCC1 |
| **388** | *Radix Salviae* | apigenin | P61073 | CXCR4 |
| **389** | *Radix Salviae* | apigenin | Q02880 | TOP2B |
| **390** | *Radix Salviae* | apigenin | P01375 | TNF |
| **391** | *Radix Salviae* | apigenin | P11388 | TOP2A |
| **392** | *Radix Salviae* | apigenin | P11166 | SLC2A1 |
| **393** | *Radix Salviae* | Baicalin | P05181 | CYP2E1 |
| **394** | *Radix Salviae* | Baicalin | P21589 | NT5E |
| **395** | *Radix Salviae* | Baicalin | O60603 | TLR2 |
| **396** | *Radix Salviae* | Baicalin | Q9UBK8 | MTRR |
| **397** | *Radix Salviae* | Baicalin | P01133 | EGF |
| **398** | *Radix Salviae* | Baicalin | P29474 | NOS3 |
| **399** | *Radix Salviae* | Baicalin | P42574 | CASP3 |
| **400** | *Radix Salviae* | Baicalin | P35228 | NOS2 |
| **401** | *Radix Salviae* | Baicalin | P08684 | CYP3A4 |
| **402** | *Radix Salviae* | Baicalin | Q16665 | HIF1A |
| **403** | *Radix Salviae* | Baicalin | Q14790 | CASP8 |
| **404** | *Radix Salviae* | Baicalin | O00206 | TLR4 |
| **405** | *Radix Salviae* | Baicalin | P09917 | ALOX5 |
| **406** | *Radix Salviae* | Baicalin | P01106 | MYC |
| **407** | *Radix Salviae* | Baicalin | P01375 | TNF |
| **408** | *Radix Salviae* | Baicalin | P16435 | POR |
| **409** | *Radix Salviae* | caffeic acid | P28482 | MAPK1 |
| **410** | *Radix Salviae* | caffeic acid | P08253 | MMP2 |
| **411** | *Radix Salviae* | caffeic acid | P05164 | MPO |
| **412** | *Radix Salviae* | caffeic acid | P04040 | CAT |
| **413** | *Radix Salviae* | caffeic acid | P14679 | TYR |
| **414** | *Radix Salviae* | caffeic acid | P50225 | SULT1A1 |
| **415** | *Radix Salviae* | caffeic acid | P03956 | MMP1 |
| **416** | *Radix Salviae* | caffeic acid | Q9HAW8 | UGT1A10 |
| **417** | *Radix Salviae* | caffeic acid | P45983 | MAPK8 |
| **418** | *Radix Salviae* | caffeic acid | P14780 | MMP9 |
| **419** | *Radix Salviae* | caffeic acid | Q9HAW7 | UGT1A7 |
| **420** | *Radix Salviae* | caffeic acid | Q9HAW9 | UGT1A8 |
| **421** | *Radix Salviae* | caffeic acid | P09917 | ALOX5 |
| **422** | *Radix Salviae* | caffeic acid | P35503 | UGT1A3 |
| **423** | *Radix Salviae* | cryptotanshinone | P06276 | BCHE |
| **424** | *Radix Salviae* | cryptotanshinone | P40763 | STAT3 |
| **425** | *Radix Salviae* | cryptotanshinone | P22303 | ACHE |
| **426** | *Radix Salviae* | cryptotanshinone | O75469 | NR1I2 |
| **427** | *Radix Salviae* | cryptotanshinone | P05305 | EDN1 |
| **428** | *Radix Salviae* | cryptotanshinone | P05067 | APP |
| **429** | *Radix Salviae* | cryptotanshinone | P24385 | CCND1 |
| **430** | *Radix Salviae* | cryptotanshinone | Q07817 | BCL2L1 |
| **431** | *Radix Salviae* | cryptotanshinone | P01375 | TNF |
| **432** | *Radix Salviae* | cryptotanshinone | Q04206 | RELA |
| **433** | *Radix Salviae* | Danshenol A | P15121 | AKR1B1 |
| **434** | *Radix Salviae* | Danshenol A | O60218 | AKR1B10 |
| **435** | *Radix Salviae* | danshensu | P09601 | HMOX1 |
| **436** | *Radix Salviae* | danshensu | P00749 | PLAU |
| **437** | *Radix Salviae* | danshensu | P01137 | TGFB1 |
| **438** | *Radix Salviae* | danshensu | P00441 | SOD1 |
| **439** | *Radix Salviae* | danshensu | Q04206 | RELA |
| **440** | *Radix Salviae* | dihydrotanshinone i | P22303 | ACHE |
| **441** | *Radix Salviae* | DTY | P25789 | PSMA4 |
| **442** | *Radix Salviae* | DTY | P43686 | PSMC4 |
| **443** | *Radix Salviae* | DTY | Q16401 | PSMD5 |
| **444** | *Radix Salviae* | DTY | P48556 | PSMD8 |
| **445** | *Radix Salviae* | DTY | P25788 | PSMA3 |
| **446** | *Radix Salviae* | DTY | Q9UL46 | PSME2 |
| **447** | *Radix Salviae* | DTY | O75832 | PSMD10 |
| **448** | *Radix Salviae* | DTY | P51665 | PSMD7 |
| **449** | *Radix Salviae* | DTY | P60468 | SEC61B |
| **450** | *Radix Salviae* | DTY | Q9UIQ6 | LNPEP |
| **451** | *Radix Salviae* | DTY | P61619 | SEC61A1 |
| **452** | *Radix Salviae* | DTY | P00505 | GOT2 |
| **453** | *Radix Salviae* | DTY | Q99436 | PSMB7 |
| **454** | *Radix Salviae* | DTY | P62191 | PSMC1 |
| **455** | *Radix Salviae* | DTY | P60900 | PSMA6 |
| **456** | *Radix Salviae* | DTY | Q01650 | SLC7A5 |
| **457** | *Radix Salviae* | DTY | O00231 | PSMD11 |
| **458** | *Radix Salviae* | DTY | Q13867 | BLMH |
| **459** | *Radix Salviae* | DTY | P20618 | PSMB1 |
| **460** | *Radix Salviae* | DTY | P14679 | TYR |
| **461** | *Radix Salviae* | DTY | O43242 | PSMD3 |
| **462** | *Radix Salviae* | DTY | P40763 | STAT3 |
| **463** | *Radix Salviae* | DTY | Q07075 | ENPEP |
| **464** | *Radix Salviae* | DTY | P01133 | EGF |
| **465** | *Radix Salviae* | DTY | P04626 | ERBB2 |
| **466** | *Radix Salviae* | DTY | P31749 | AKT1 |
| **467** | *Radix Salviae* | DTY | P28072 | PSMB6 |
| **468** | *Radix Salviae* | DTY | P28066 | PSMA5 |
| **469** | *Radix Salviae* | DTY | P62979 | RPS27A |
| **470** | *Radix Salviae* | DTY | O75223 | GGCT |
| **471** | *Radix Salviae* | DTY | P00533 | EGFR |
| **472** | *Radix Salviae* | DTY | P28070 | PSMB4 |
| **473** | *Radix Salviae* | DTY | P35998 | PSMC2 |
| **474** | *Radix Salviae* | DTY | P61289 | PSME3 |
| **475** | *Radix Salviae* | DTY | Q15008 | PSMD6 |
| **476** | *Radix Salviae* | DTY | Q9NZ08 | ERAP1 |
| **477** | *Radix Salviae* | DTY | Q9H9S3 | SEC61A2 |
| **478** | *Radix Salviae* | DTY | P17980 | PSMC3 |
| **479** | *Radix Salviae* | DTY | P30101 | PDIA3 |
| **480** | *Radix Salviae* | DTY | P0CG47 | UBB |
| **481** | *Radix Salviae* | DTY | P35568 | IRS1 |
| **482** | *Radix Salviae* | DTY | Q99460 | PSMD1 |
| **483** | *Radix Salviae* | DTY | Q13200 | PSMD2 |
| **484** | *Radix Salviae* | DTY | P62195 | PSMC5 |
| **485** | *Radix Salviae* | DTY | Q8TAA3 | PSMA8 |
| **486** | *Radix Salviae* | DTY | P55786 | NPEPPS |
| **487** | *Radix Salviae* | DTY | Q9UHI5 | SLC7A8 |
| **488** | *Radix Salviae* | DTY | P27797 | CALR |
| **489** | *Radix Salviae* | DTY | Q96KP4 | CNDP2 |
| **490** | *Radix Salviae* | DTY | Q92530 | PSMF1 |
| **491** | *Radix Salviae* | DTY | P60059 | SEC61G |
| **492** | *Radix Salviae* | DTY | Q96RQ9 | IL4I1 |
| **493** | *Radix Salviae* | DTY | P0CG48 | UBC |
| **494** | *Radix Salviae* | DTY | Q7L5Y1 | ENOSF1 |
| **495** | *Radix Salviae* | DTY | P17735 | TAT |
| **496** | *Radix Salviae* | DTY | O00232 | PSMD12 |
| **497** | *Radix Salviae* | DTY | P12931 | SRC |
| **498** | *Radix Salviae* | DTY | P40306 | PSMB10 |
| **499** | *Radix Salviae* | DTY | P17693 | HLA-G |
| **500** | *Radix Salviae* | DTY | P28074 | PSMB5 |
| **501** | *Radix Salviae* | DTY | P55036 | PSMD4 |
| **502** | *Radix Salviae* | DTY | P25774 | CTSS |
| **503** | *Radix Salviae* | DTY | P17174 | GOT1 |
| **504** | *Radix Salviae* | DTY | O14818 | PSMA7 |
| **505** | *Radix Salviae* | DTY | P60484 | PTEN |
| **506** | *Radix Salviae* | DTY | P49721 | PSMB2 |
| **507** | *Radix Salviae* | DTY | P10275 | AR |
| **508** | *Radix Salviae* | DTY | P29144 | TPP2 |
| **509** | *Radix Salviae* | DTY | P30499 | HLA-C |
| **510** | *Radix Salviae* | DTY | P30443 | HLA-A |
| **511** | *Radix Salviae* | DTY | P08195 | SLC3A2 |
| **512** | *Radix Salviae* | DTY | P01185 | AVP |
| **513** | *Radix Salviae* | DTY | P07101 | TH |
| **514** | *Radix Salviae* | DTY | O60674 | JAK2 |
| **515** | *Radix Salviae* | DTY | Q06323 | PSME1 |
| **516** | *Radix Salviae* | DTY | Q14997 | PSME4 |
| **517** | *Radix Salviae* | DTY | A5LHX3 | PSMB11 |
| **518** | *Radix Salviae* | DTY | O00487 | PSMD14 |
| **519** | *Radix Salviae* | DTY | P62987 | UBA52 |
| **520** | *Radix Salviae* | DTY | Q9UNM6 | PSMD13 |
| **521** | *Radix Salviae* | DTY | P01889 | HLA-B |
| **522** | *Radix Salviae* | DTY | Q6P179 | ERAP2 |
| **523** | *Radix Salviae* | DTY | P62333 | PSMC6 |
| **524** | *Radix Salviae* | DTY | P15151 | PVR |
| **525** | *Radix Salviae* | DTY | P25786 | PSMA1 |
| **526** | *Radix Salviae* | DTY | O00233 | PSMD9 |
| **527** | *Radix Salviae* | DTY | P61769 | B2M |
| **528** | *Radix Salviae* | Glucosol | P35222 | CTNNB1 |
| **529** | *Radix Salviae* | Gulutamine | P25789 | PSMA4 |
| **530** | *Radix Salviae* | Gulutamine | P43686 | PSMC4 |
| **531** | *Radix Salviae* | Gulutamine | P21917 | DRD4 |
| **532** | *Radix Salviae* | Gulutamine | P09622 | DLD |
| **533** | *Radix Salviae* | Gulutamine | Q16401 | PSMD5 |
| **534** | *Radix Salviae* | Gulutamine | P48556 | PSMD8 |
| **535** | *Radix Salviae* | Gulutamine | P25788 | PSMA3 |
| **536** | *Radix Salviae* | Gulutamine | P46663 | BDKRB1 |
| **537** | *Radix Salviae* | Gulutamine | Q9UL46 | PSME2 |
| **538** | *Radix Salviae* | Gulutamine | O75832 | PSMD10 |
| **539** | *Radix Salviae* | Gulutamine | P51665 | PSMD7 |
| **540** | *Radix Salviae* | Gulutamine | P00390 | GSR |
| **541** | *Radix Salviae* | Gulutamine | Q13155 | AIMP2 |
| **542** | *Radix Salviae* | Gulutamine | P60468 | SEC61B |
| **543** | *Radix Salviae* | Gulutamine | P01031 | C5 |
| **544** | *Radix Salviae* | Gulutamine | P30838 | ALDH3A1 |
| **545** | *Radix Salviae* | Gulutamine | Q9UIQ6 | LNPEP |
| **546** | *Radix Salviae* | Gulutamine | P54136 | RARS |
| **547** | *Radix Salviae* | Gulutamine | P04040 | CAT |
| **548** | *Radix Salviae* | Gulutamine | P61619 | SEC61A1 |
| **549** | *Radix Salviae* | Gulutamine | P00505 | GOT2 |
| **550** | *Radix Salviae* | Gulutamine | P01024 | C3 |
| **551** | *Radix Salviae* | Gulutamine | Q99705 | MCHR1 |
| **552** | *Radix Salviae* | Gulutamine | O94788 | ALDH1A2 |
| **553** | *Radix Salviae* | Gulutamine | P48448 | ALDH3B2 |
| **554** | *Radix Salviae* | Gulutamine | Q9GZQ4 | NMUR2 |
| **555** | *Radix Salviae* | Gulutamine | P49286 | MTNR1B |
| **556** | *Radix Salviae* | Gulutamine | P04083 | ANXA1 |
| **557** | *Radix Salviae* | Gulutamine | Q3SY69 | ALDH1L2 |
| **558** | *Radix Salviae* | Gulutamine | Q99436 | PSMB7 |
| **559** | *Radix Salviae* | Gulutamine | P62191 | PSMC1 |
| **560** | *Radix Salviae* | Gulutamine | P60900 | PSMA6 |
| **561** | *Radix Salviae* | Gulutamine | O00231 | PSMD11 |
| **562** | *Radix Salviae* | Gulutamine | Q13867 | BLMH |
| **563** | *Radix Salviae* | Gulutamine | P05091 | ALDH2 |
| **564** | *Radix Salviae* | Gulutamine | P56192 | MARS |
| **565** | *Radix Salviae* | Gulutamine | P20618 | PSMB1 |
| **566** | *Radix Salviae* | Gulutamine | P42336 | PIK3CA |
| **567** | *Radix Salviae* | Gulutamine | P14868 | DARS |
| **568** | *Radix Salviae* | Gulutamine | P48645 | NMU |
| **569** | *Radix Salviae* | Gulutamine | O43242 | PSMD3 |
| **570** | *Radix Salviae* | Gulutamine | P01189 | POMC |
| **571** | *Radix Salviae* | Gulutamine | P01042 | KNG1 |
| **572** | *Radix Salviae* | Gulutamine | Q07075 | ENPEP |
| **573** | *Radix Salviae* | Gulutamine | Q9H2A2 | ALDH8A1 |
| **574** | *Radix Salviae* | Gulutamine | P28072 | PSMB6 |
| **575** | *Radix Salviae* | Gulutamine | P28066 | PSMA5 |
| **576** | *Radix Salviae* | Gulutamine | P62979 | RPS27A |
| **577** | *Radix Salviae* | Gulutamine | Q13304 | GPR17 |
| **578** | *Radix Salviae* | Gulutamine | O75223 | GGCT |
| **579** | *Radix Salviae* | Gulutamine | P08913 | ADRA2A |
| **580** | *Radix Salviae* | Gulutamine | Q969V1 | MCHR2 |
| **581** | *Radix Salviae* | Gulutamine | P05067 | APP |
| **582** | *Radix Salviae* | Gulutamine | P15121 | AKR1B1 |
| **583** | *Radix Salviae* | Gulutamine | P28070 | PSMB4 |
| **584** | *Radix Salviae* | Gulutamine | P30038 | ALDH4A1 |
| **585** | *Radix Salviae* | Gulutamine | P35998 | PSMC2 |
| **586** | *Radix Salviae* | Gulutamine | Q8IZ83 | ALDH16A1 |
| **587** | *Radix Salviae* | Gulutamine | P61289 | PSME3 |
| **588** | *Radix Salviae* | Gulutamine | P02768 | ALB |
| **589** | *Radix Salviae* | Gulutamine | Q15008 | PSMD6 |
| **590** | *Radix Salviae* | Gulutamine | Q9NZ08 | ERAP1 |
| **591** | *Radix Salviae* | Gulutamine | O76081 | RGS20 |
| **592** | *Radix Salviae* | Gulutamine | Q99527 | GPER1 |
| **593** | *Radix Salviae* | Gulutamine | P00352 | ALDH1A1 |
| **594** | *Radix Salviae* | Gulutamine | Q9H9S3 | SEC61A2 |
| **595** | *Radix Salviae* | Gulutamine | P17980 | PSMC3 |
| **596** | *Radix Salviae* | Gulutamine | P30101 | PDIA3 |
| **597** | *Radix Salviae* | Gulutamine | P48039 | MTNR1A |
| **598** | *Radix Salviae* | Gulutamine | P0CG47 | UBB |
| **599** | *Radix Salviae* | Gulutamine | P02778 | CXCL10 |
| **600** | *Radix Salviae* | Gulutamine | Q9HB89 | NMUR1 |
| **601** | *Radix Salviae* | Gulutamine | P10145 | CXCL8 |
| **602** | *Radix Salviae* | Gulutamine | P47897 | QARS |
| **603** | *Radix Salviae* | Gulutamine | P15104 | GLUL |
| **604** | *Radix Salviae* | Gulutamine | Q99460 | PSMD1 |
| **605** | *Radix Salviae* | Gulutamine | Q13200 | PSMD2 |
| **606** | *Radix Salviae* | Gulutamine | P62195 | PSMC5 |
| **607** | *Radix Salviae* | Gulutamine | P42574 | CASP3 |
| **608** | *Radix Salviae* | Gulutamine | Q8TAA3 | PSMA8 |
| **609** | *Radix Salviae* | Gulutamine | P51649 | ALDH5A1 |
| **610** | *Radix Salviae* | Gulutamine | P34896 | SHMT1 |
| **611** | *Radix Salviae* | Gulutamine | P35558 | PCK1 |
| **612** | *Radix Salviae* | Gulutamine | P55786 | NPEPPS |
| **613** | *Radix Salviae* | Gulutamine | P27797 | CALR |
| **614** | *Radix Salviae* | Gulutamine | O15382 | BCAT2 |
| **615** | *Radix Salviae* | Gulutamine | Q15046 | KARS |
| **616** | *Radix Salviae* | Gulutamine | Q96KP4 | CNDP2 |
| **617** | *Radix Salviae* | Gulutamine | Q92530 | PSMF1 |
| **618** | *Radix Salviae* | Gulutamine | Q9H1C0 | LPAR5 |
| **619** | *Radix Salviae* | Gulutamine | P20382 | PMCH |
| **620** | *Radix Salviae* | Gulutamine | P47895 | ALDH1A3 |
| **621** | *Radix Salviae* | Gulutamine | P49795 | RGS19 |
| **622** | *Radix Salviae* | Gulutamine | P59768 | GNG2 |
| **623** | *Radix Salviae* | Gulutamine | P25090 | FPR2 |
| **624** | *Radix Salviae* | Gulutamine | P60059 | SEC61G |
| **625** | *Radix Salviae* | Gulutamine | P63096 | GNAI1 |
| **626** | *Radix Salviae* | Gulutamine | P0CG48 | UBC |
| **627** | *Radix Salviae* | Gulutamine | P51648 | ALDH3A2 |
| **628** | *Radix Salviae* | Gulutamine | Q7L5Y1 | ENOSF1 |
| **629** | *Radix Salviae* | Gulutamine | P49189 | ALDH9A1 |
| **630** | *Radix Salviae* | Gulutamine | P21730 | C5AR1 |
| **631** | *Radix Salviae* | Gulutamine | P17735 | TAT |
| **632** | *Radix Salviae* | Gulutamine | O00232 | PSMD12 |
| **633** | *Radix Salviae* | Gulutamine | P0DJI8 | SAA1 |
| **634** | *Radix Salviae* | Gulutamine | Q42521 | GAD1 |
| **635** | *Radix Salviae* | Gulutamine | P40306 | PSMB10 |
| **636** | *Radix Salviae* | Gulutamine | Q92633 | LPAR1 |
| **637** | *Radix Salviae* | Gulutamine | O60218 | AKR1B10 |
| **638** | *Radix Salviae* | Gulutamine | P17693 | HLA-G |
| **639** | *Radix Salviae* | Gulutamine | P25929 | NPY1R |
| **640** | *Radix Salviae* | Gulutamine | Q8N5D0 | WDTC1 |
| **641** | *Radix Salviae* | Gulutamine | P28074 | PSMB5 |
| **642** | *Radix Salviae* | Gulutamine | P49802 | RGS7 |
| **643** | *Radix Salviae* | Gulutamine | P01019 | AGT |
| **644** | *Radix Salviae* | Gulutamine | P07814 | EPRS |
| **645** | *Radix Salviae* | Gulutamine | Q08116 | RGS1 |
| **646** | *Radix Salviae* | Gulutamine | Q9NS28 | RGS18 |
| **647** | *Radix Salviae* | Gulutamine | P55036 | PSMD4 |
| **648** | *Radix Salviae* | Gulutamine | P25774 | CTSS |
| **649** | *Radix Salviae* | Gulutamine | O43665 | RGS10 |
| **650** | *Radix Salviae* | Gulutamine | O43175 | PHGDH |
| **651** | *Radix Salviae* | Gulutamine | P08754 | GNAI3 |
| **652** | *Radix Salviae* | Gulutamine | P17174 | GOT1 |
| **653** | *Radix Salviae* | Gulutamine | Q9UBY5 | LPAR3 |
| **654** | *Radix Salviae* | Gulutamine | O14818 | PSMA7 |
| **655** | *Radix Salviae* | Gulutamine | P49721 | PSMB2 |
| **656** | *Radix Salviae* | Gulutamine | P09917 | ALOX5 |
| **657** | *Radix Salviae* | Gulutamine | P41252 | IARS |
| **658** | *Radix Salviae* | Gulutamine | P29144 | TPP2 |
| **659** | *Radix Salviae* | Gulutamine | P30499 | HLA-C |
| **660** | *Radix Salviae* | Gulutamine | P30443 | HLA-A |
| **661** | *Radix Salviae* | Gulutamine | Q5H8A3 | NMS |
| **662** | *Radix Salviae* | Gulutamine | P30837 | ALDH1B1 |
| **663** | *Radix Salviae* | Gulutamine | P08195 | SLC3A2 |
| **664** | *Radix Salviae* | Gulutamine | P05305 | EDN1 |
| **665** | *Radix Salviae* | Gulutamine | O43324 | EEF1E1 |
| **666** | *Radix Salviae* | Gulutamine | P01185 | AVP |
| **667** | *Radix Salviae* | Gulutamine | P42330 | AKR1C3 |
| **668** | *Radix Salviae* | Gulutamine | Q06323 | PSME1 |
| **669** | *Radix Salviae* | Gulutamine | Q96BT7 | ALKBH8 |
| **670** | *Radix Salviae* | Gulutamine | O75891 | ALDH1L1 |
| **671** | *Radix Salviae* | Gulutamine | Q9P2J5 | LARS |
| **672** | *Radix Salviae* | Gulutamine | Q12904 | AIMP1 |
| **673** | *Radix Salviae* | Gulutamine | P09341 | CXCL1 |
| **674** | *Radix Salviae* | Gulutamine | P33527 | ABCC1 |
| **675** | *Radix Salviae* | Gulutamine | Q14997 | PSME4 |
| **676** | *Radix Salviae* | Gulutamine | Q9HBW0 | LPAR2 |
| **677** | *Radix Salviae* | Gulutamine | P18825 | ADRA2C |
| **678** | *Radix Salviae* | Gulutamine | A5LHX3 | PSMB11 |
| **679** | *Radix Salviae* | Gulutamine | O43566 | RGS14 |
| **680** | *Radix Salviae* | Gulutamine | O00487 | PSMD14 |
| **681** | *Radix Salviae* | Gulutamine | P61073 | CXCR4 |
| **682** | *Radix Salviae* | Gulutamine | P49419 | ALDH7A1 |
| **683** | *Radix Salviae* | Gulutamine | P01275 | GCG |
| **684** | *Radix Salviae* | Gulutamine | Q5TF58 | IFFO2 |
| **685** | *Radix Salviae* | Gulutamine | P62987 | UBA52 |
| **686** | *Radix Salviae* | Gulutamine | P21731 | TBXA2R |
| **687** | *Radix Salviae* | Gulutamine | P35372 | OPRM1 |
| **688** | *Radix Salviae* | Gulutamine | Q9UNM6 | PSMD13 |
| **689** | *Radix Salviae* | Gulutamine | P49798 | RGS4 |
| **690** | *Radix Salviae* | Gulutamine | P01889 | HLA-B |
| **691** | *Radix Salviae* | Gulutamine | Q6P179 | ERAP2 |
| **692** | *Radix Salviae* | Gulutamine | O43709 | WBSCR22 |
| **693** | *Radix Salviae* | Gulutamine | P62333 | PSMC6 |
| **694** | *Radix Salviae* | Gulutamine | P15151 | PVR |
| **695** | *Radix Salviae* | Gulutamine | P25786 | PSMA1 |
| **696** | *Radix Salviae* | Gulutamine | O00233 | PSMD9 |
| **697** | *Radix Salviae* | Gulutamine | P54687 | BCAT1 |
| **698** | *Radix Salviae* | Gulutamine | P49758 | RGS6 |
| **699** | *Radix Salviae* | Gulutamine | P61769 | B2M |
| **700** | *Radix Salviae* | h-Met-h | P25789 | PSMA4 |
| **701** | *Radix Salviae* | h-Met-h | P43686 | PSMC4 |
| **702** | *Radix Salviae* | h-Met-h | Q16401 | PSMD5 |
| **703** | *Radix Salviae* | h-Met-h | P48556 | PSMD8 |
| **704** | *Radix Salviae* | h-Met-h | P25788 | PSMA3 |
| **705** | *Radix Salviae* | h-Met-h | Q9UL46 | PSME2 |
| **706** | *Radix Salviae* | h-Met-h | O75832 | PSMD10 |
| **707** | *Radix Salviae* | h-Met-h | P51665 | PSMD7 |
| **708** | *Radix Salviae* | h-Met-h | P00390 | GSR |
| **709** | *Radix Salviae* | h-Met-h | Q13155 | AIMP2 |
| **710** | *Radix Salviae* | h-Met-h | P60468 | SEC61B |
| **711** | *Radix Salviae* | h-Met-h | Q9UIQ6 | LNPEP |
| **712** | *Radix Salviae* | h-Met-h | P54136 | RARS |
| **713** | *Radix Salviae* | h-Met-h | P04040 | CAT |
| **714** | *Radix Salviae* | h-Met-h | P61619 | SEC61A1 |
| **715** | *Radix Salviae* | h-Met-h | Q99436 | PSMB7 |
| **716** | *Radix Salviae* | h-Met-h | P62191 | PSMC1 |
| **717** | *Radix Salviae* | h-Met-h | P60900 | PSMA6 |
| **718** | *Radix Salviae* | h-Met-h | Q01650 | SLC7A5 |
| **719** | *Radix Salviae* | h-Met-h | O00231 | PSMD11 |
| **720** | *Radix Salviae* | h-Met-h | Q13867 | BLMH |
| **721** | *Radix Salviae* | h-Met-h | P56192 | MARS |
| **722** | *Radix Salviae* | h-Met-h | P20618 | PSMB1 |
| **723** | *Radix Salviae* | h-Met-h | P14679 | TYR |
| **724** | *Radix Salviae* | h-Met-h | P14868 | DARS |
| **725** | *Radix Salviae* | h-Met-h | O43242 | PSMD3 |
| **726** | *Radix Salviae* | h-Met-h | Q9UBK8 | MTRR |
| **727** | *Radix Salviae* | h-Met-h | Q9H2J7 | SLC6A15 |
| **728** | *Radix Salviae* | h-Met-h | P00441 | SOD1 |
| **729** | *Radix Salviae* | h-Met-h | Q01959 | SLC6A3 |
| **730** | *Radix Salviae* | h-Met-h | P28072 | PSMB6 |
| **731** | *Radix Salviae* | h-Met-h | P28066 | PSMA5 |
| **732** | *Radix Salviae* | h-Met-h | P62979 | RPS27A |
| **733** | *Radix Salviae* | h-Met-h | O75223 | GGCT |
| **734** | *Radix Salviae* | h-Met-h | P28070 | PSMB4 |
| **735** | *Radix Salviae* | h-Met-h | P35998 | PSMC2 |
| **736** | *Radix Salviae* | h-Met-h | P61289 | PSME3 |
| **737** | *Radix Salviae* | h-Met-h | P02768 | ALB |
| **738** | *Radix Salviae* | h-Met-h | Q15008 | PSMD6 |
| **739** | *Radix Salviae* | h-Met-h | Q9NZ08 | ERAP1 |
| **740** | *Radix Salviae* | h-Met-h | Q9H9S3 | SEC61A2 |
| **741** | *Radix Salviae* | h-Met-h | P17980 | PSMC3 |
| **742** | *Radix Salviae* | h-Met-h | P30101 | PDIA3 |
| **743** | *Radix Salviae* | h-Met-h | P0CG47 | UBB |
| **744** | *Radix Salviae* | h-Met-h | P47897 | QARS |
| **745** | *Radix Salviae* | h-Met-h | P15104 | GLUL |
| **746** | *Radix Salviae* | h-Met-h | Q99460 | PSMD1 |
| **747** | *Radix Salviae* | h-Met-h | Q13200 | PSMD2 |
| **748** | *Radix Salviae* | h-Met-h | P62195 | PSMC5 |
| **749** | *Radix Salviae* | h-Met-h | Q8TAA3 | PSMA8 |
| **750** | *Radix Salviae* | h-Met-h | P34896 | SHMT1 |
| **751** | *Radix Salviae* | h-Met-h | P55786 | NPEPPS |
| **752** | *Radix Salviae* | h-Met-h | Q9UHI5 | SLC7A8 |
| **753** | *Radix Salviae* | h-Met-h | P27797 | CALR |
| **754** | *Radix Salviae* | h-Met-h | Q96N87 | SLC6A18 |
| **755** | *Radix Salviae* | h-Met-h | Q15046 | KARS |
| **756** | *Radix Salviae* | h-Met-h | Q96KP4 | CNDP2 |
| **757** | *Radix Salviae* | h-Met-h | Q92530 | PSMF1 |
| **758** | *Radix Salviae* | h-Met-h | P30536 | TSPO |
| **759** | *Radix Salviae* | h-Met-h | P60059 | SEC61G |
| **760** | *Radix Salviae* | h-Met-h | Q96RQ9 | IL4I1 |
| **761** | *Radix Salviae* | h-Met-h | P0CG48 | UBC |
| **762** | *Radix Salviae* | h-Met-h | P17735 | TAT |
| **763** | *Radix Salviae* | h-Met-h | O00232 | PSMD12 |
| **764** | *Radix Salviae* | h-Met-h | Q42521 | GAD1 |
| **765** | *Radix Salviae* | h-Met-h | P40306 | PSMB10 |
| **766** | *Radix Salviae* | h-Met-h | P17693 | HLA-G |
| **767** | *Radix Salviae* | h-Met-h | Q8N5D0 | WDTC1 |
| **768** | *Radix Salviae* | h-Met-h | P28074 | PSMB5 |
| **769** | *Radix Salviae* | h-Met-h | P07814 | EPRS |
| **770** | *Radix Salviae* | h-Met-h | P55036 | PSMD4 |
| **771** | *Radix Salviae* | h-Met-h | P25774 | CTSS |
| **772** | *Radix Salviae* | h-Met-h | P17174 | GOT1 |
| **773** | *Radix Salviae* | h-Met-h | O14818 | PSMA7 |
| **774** | *Radix Salviae* | h-Met-h | P49721 | PSMB2 |
| **775** | *Radix Salviae* | h-Met-h | P41252 | IARS |
| **776** | *Radix Salviae* | h-Met-h | P29144 | TPP2 |
| **777** | *Radix Salviae* | h-Met-h | P30499 | HLA-C |
| **778** | *Radix Salviae* | h-Met-h | P30443 | HLA-A |
| **779** | *Radix Salviae* | h-Met-h | P08195 | SLC3A2 |
| **780** | *Radix Salviae* | h-Met-h | O43324 | EEF1E1 |
| **781** | *Radix Salviae* | h-Met-h | Q06323 | PSME1 |
| **782** | *Radix Salviae* | h-Met-h | Q9P2J5 | LARS |
| **783** | *Radix Salviae* | h-Met-h | Q12904 | AIMP1 |
| **784** | *Radix Salviae* | h-Met-h | Q14997 | PSME4 |
| **785** | *Radix Salviae* | h-Met-h | A5LHX3 | PSMB11 |
| **786** | *Radix Salviae* | h-Met-h | O00487 | PSMD14 |
| **787** | *Radix Salviae* | h-Met-h | P62987 | UBA52 |
| **788** | *Radix Salviae* | h-Met-h | Q9UNM6 | PSMD13 |
| **789** | *Radix Salviae* | h-Met-h | P01889 | HLA-B |
| **790** | *Radix Salviae* | h-Met-h | Q6P179 | ERAP2 |
| **791** | *Radix Salviae* | h-Met-h | P62333 | PSMC6 |
| **792** | *Radix Salviae* | h-Met-h | P15151 | PVR |
| **793** | *Radix Salviae* | h-Met-h | P25786 | PSMA1 |
| **794** | *Radix Salviae* | h-Met-h | O00233 | PSMD9 |
| **795** | *Radix Salviae* | h-Met-h | P61769 | B2M |
| **796** | *Radix Salviae* | isoferulic acid | P21399 | ACO1 |
| **797** | *Radix Salviae* | isoferulic acid | P09341 | CXCL1 |
| **798** | *Radix Salviae* | isoimperatorin | P05177 | CYP1A2 |
| **799** | *Radix Salviae* | isoimperatorin | P10635 | CYP2D6 |
| **800** | *Radix Salviae* | Istidina | P25789 | PSMA4 |
| **801** | *Radix Salviae* | Istidina | P43686 | PSMC4 |
| **802** | *Radix Salviae* | Istidina | Q16401 | PSMD5 |
| **803** | *Radix Salviae* | Istidina | P48556 | PSMD8 |
| **804** | *Radix Salviae* | Istidina | P25788 | PSMA3 |
| **805** | *Radix Salviae* | Istidina | Q9UL46 | PSME2 |
| **806** | *Radix Salviae* | Istidina | O75832 | PSMD10 |
| **807** | *Radix Salviae* | Istidina | P51665 | PSMD7 |
| **808** | *Radix Salviae* | Istidina | P30519 | HMOX2 |
| **809** | *Radix Salviae* | Istidina | P27169 | PON1 |
| **810** | *Radix Salviae* | Istidina | P60468 | SEC61B |
| **811** | *Radix Salviae* | Istidina | Q9UIQ6 | LNPEP |
| **812** | *Radix Salviae* | Istidina | P04040 | CAT |
| **813** | *Radix Salviae* | Istidina | P61619 | SEC61A1 |
| **814** | *Radix Salviae* | Istidina | Q99436 | PSMB7 |
| **815** | *Radix Salviae* | Istidina | P62191 | PSMC1 |
| **816** | *Radix Salviae* | Istidina | P60900 | PSMA6 |
| **817** | *Radix Salviae* | Istidina | Q01650 | SLC7A5 |
| **818** | *Radix Salviae* | Istidina | O00231 | PSMD11 |
| **819** | *Radix Salviae* | Istidina | Q13867 | BLMH |
| **820** | *Radix Salviae* | Istidina | P20618 | PSMB1 |
| **821** | *Radix Salviae* | Istidina | P14679 | TYR |
| **822** | *Radix Salviae* | Istidina | P06276 | BCHE |
| **823** | *Radix Salviae* | Istidina | O43242 | PSMD3 |
| **824** | *Radix Salviae* | Istidina | P28072 | PSMB6 |
| **825** | *Radix Salviae* | Istidina | P28066 | PSMA5 |
| **826** | *Radix Salviae* | Istidina | P62979 | RPS27A |
| **827** | *Radix Salviae* | Istidina | O75223 | GGCT |
| **828** | *Radix Salviae* | Istidina | P28070 | PSMB4 |
| **829** | *Radix Salviae* | Istidina | P35998 | PSMC2 |
| **830** | *Radix Salviae* | Istidina | P61289 | PSME3 |
| **831** | *Radix Salviae* | Istidina | P02768 | ALB |
| **832** | *Radix Salviae* | Istidina | Q15008 | PSMD6 |
| **833** | *Radix Salviae* | Istidina | Q9NZ08 | ERAP1 |
| **834** | *Radix Salviae* | Istidina | Q9H9S3 | SEC61A2 |
| **835** | *Radix Salviae* | Istidina | P17980 | PSMC3 |
| **836** | *Radix Salviae* | Istidina | P30101 | PDIA3 |
| **837** | *Radix Salviae* | Istidina | P0CG47 | UBB |
| **838** | *Radix Salviae* | Istidina | P15104 | GLUL |
| **839** | *Radix Salviae* | Istidina | Q99460 | PSMD1 |
| **840** | *Radix Salviae* | Istidina | P21399 | ACO1 |
| **841** | *Radix Salviae* | Istidina | Q13200 | PSMD2 |
| **842** | *Radix Salviae* | Istidina | P62195 | PSMC5 |
| **843** | *Radix Salviae* | Istidina | Q8TAA3 | PSMA8 |
| **844** | *Radix Salviae* | Istidina | P55786 | NPEPPS |
| **845** | *Radix Salviae* | Istidina | Q9UHI5 | SLC7A8 |
| **846** | *Radix Salviae* | Istidina | P27797 | CALR |
| **847** | *Radix Salviae* | Istidina | Q96KP4 | CNDP2 |
| **848** | *Radix Salviae* | Istidina | Q92530 | PSMF1 |
| **849** | *Radix Salviae* | Istidina | P60059 | SEC61G |
| **850** | *Radix Salviae* | Istidina | P0CG48 | UBC |
| **851** | *Radix Salviae* | Istidina | P17735 | TAT |
| **852** | *Radix Salviae* | Istidina | O00232 | PSMD12 |
| **853** | *Radix Salviae* | Istidina | P40306 | PSMB10 |
| **854** | *Radix Salviae* | Istidina | P17693 | HLA-G |
| **855** | *Radix Salviae* | Istidina | Q8N5D0 | WDTC1 |
| **856** | *Radix Salviae* | Istidina | P28074 | PSMB5 |
| **857** | *Radix Salviae* | Istidina | P55036 | PSMD4 |
| **858** | *Radix Salviae* | Istidina | P25774 | CTSS |
| **859** | *Radix Salviae* | Istidina | O14818 | PSMA7 |
| **860** | *Radix Salviae* | Istidina | P49721 | PSMB2 |
| **861** | *Radix Salviae* | Istidina | P29144 | TPP2 |
| **862** | *Radix Salviae* | Istidina | P30499 | HLA-C |
| **863** | *Radix Salviae* | Istidina | P30443 | HLA-A |
| **864** | *Radix Salviae* | Istidina | P08195 | SLC3A2 |
| **865** | *Radix Salviae* | Istidina | P07101 | TH |
| **866** | *Radix Salviae* | Istidina | Q06323 | PSME1 |
| **867** | *Radix Salviae* | Istidina | Q14997 | PSME4 |
| **868** | *Radix Salviae* | Istidina | A5LHX3 | PSMB11 |
| **869** | *Radix Salviae* | Istidina | O00487 | PSMD14 |
| **870** | *Radix Salviae* | Istidina | P62987 | UBA52 |
| **871** | *Radix Salviae* | Istidina | Q9UNM6 | PSMD13 |
| **872** | *Radix Salviae* | Istidina | P01889 | HLA-B |
| **873** | *Radix Salviae* | Istidina | Q6P179 | ERAP2 |
| **874** | *Radix Salviae* | Istidina | O43709 | WBSCR22 |
| **875** | *Radix Salviae* | Istidina | P62333 | PSMC6 |
| **876** | *Radix Salviae* | Istidina | P15151 | PVR |
| **877** | *Radix Salviae* | Istidina | P25786 | PSMA1 |
| **878** | *Radix Salviae* | Istidina | O00233 | PSMD9 |
| **879** | *Radix Salviae* | Istidina | P61769 | B2M |
| **880** | *Radix Salviae* | Leucinum | P01730 | CD4 |
| **881** | *Radix Salviae* | Leucinum | P25789 | PSMA4 |
| **882** | *Radix Salviae* | Leucinum | P43686 | PSMC4 |
| **883** | *Radix Salviae* | Leucinum | P09622 | DLD |
| **884** | *Radix Salviae* | Leucinum | Q16401 | PSMD5 |
| **885** | *Radix Salviae* | Leucinum | P48556 | PSMD8 |
| **886** | *Radix Salviae* | Leucinum | P25788 | PSMA3 |
| **887** | *Radix Salviae* | Leucinum | Q9UL46 | PSME2 |
| **888** | *Radix Salviae* | Leucinum | O75832 | PSMD10 |
| **889** | *Radix Salviae* | Leucinum | P51665 | PSMD7 |
| **890** | *Radix Salviae* | Leucinum | Q13155 | AIMP2 |
| **891** | *Radix Salviae* | Leucinum | P60468 | SEC61B |
| **892** | *Radix Salviae* | Leucinum | Q9UIQ6 | LNPEP |
| **893** | *Radix Salviae* | Leucinum | P54136 | RARS |
| **894** | *Radix Salviae* | Leucinum | P04040 | CAT |
| **895** | *Radix Salviae* | Leucinum | P61619 | SEC61A1 |
| **896** | *Radix Salviae* | Leucinum | P05231 | IL6 |
| **897** | *Radix Salviae* | Leucinum | Q99436 | PSMB7 |
| **898** | *Radix Salviae* | Leucinum | O60603 | TLR2 |
| **899** | *Radix Salviae* | Leucinum | P62191 | PSMC1 |
| **900** | *Radix Salviae* | Leucinum | P60900 | PSMA6 |
| **901** | *Radix Salviae* | Leucinum | Q01650 | SLC7A5 |
| **902** | *Radix Salviae* | Leucinum | O00231 | PSMD11 |
| **903** | *Radix Salviae* | Leucinum | Q13867 | BLMH |
| **904** | *Radix Salviae* | Leucinum | P56192 | MARS |
| **905** | *Radix Salviae* | Leucinum | P20618 | PSMB1 |
| **906** | *Radix Salviae* | Leucinum | P14868 | DARS |
| **907** | *Radix Salviae* | Leucinum | O43242 | PSMD3 |
| **908** | *Radix Salviae* | Leucinum | Q9H2J7 | SLC6A15 |
| **909** | *Radix Salviae* | Leucinum | P06400 | RB1 |
| **910** | *Radix Salviae* | Leucinum | P04637 | TP53 |
| **911** | *Radix Salviae* | Leucinum | P00441 | SOD1 |
| **912** | *Radix Salviae* | Leucinum | P31749 | AKT1 |
| **913** | *Radix Salviae* | Leucinum | Q01959 | SLC6A3 |
| **914** | *Radix Salviae* | Leucinum | P28072 | PSMB6 |
| **915** | *Radix Salviae* | Leucinum | P28066 | PSMA5 |
| **916** | *Radix Salviae* | Leucinum | P62979 | RPS27A |
| **917** | *Radix Salviae* | Leucinum | O75223 | GGCT |
| **918** | *Radix Salviae* | Leucinum | P28070 | PSMB4 |
| **919** | *Radix Salviae* | Leucinum | P35998 | PSMC2 |
| **920** | *Radix Salviae* | Leucinum | P61289 | PSME3 |
| **921** | *Radix Salviae* | Leucinum | P02768 | ALB |
| **922** | *Radix Salviae* | Leucinum | Q15008 | PSMD6 |
| **923** | *Radix Salviae* | Leucinum | Q9NZ08 | ERAP1 |
| **924** | *Radix Salviae* | Leucinum | Q9H9S3 | SEC61A2 |
| **925** | *Radix Salviae* | Leucinum | P17980 | PSMC3 |
| **926** | *Radix Salviae* | Leucinum | P30101 | PDIA3 |
| **927** | *Radix Salviae* | Leucinum | P0CG47 | UBB |
| **928** | *Radix Salviae* | Leucinum | P35568 | IRS1 |
| **929** | *Radix Salviae* | Leucinum | P01100 | FOS |
| **930** | *Radix Salviae* | Leucinum | P47897 | QARS |
| **931** | *Radix Salviae* | Leucinum | P15104 | GLUL |
| **932** | *Radix Salviae* | Leucinum | Q99460 | PSMD1 |
| **933** | *Radix Salviae* | Leucinum | P21399 | ACO1 |
| **934** | *Radix Salviae* | Leucinum | Q13200 | PSMD2 |
| **935** | *Radix Salviae* | Leucinum | P62195 | PSMC5 |
| **936** | *Radix Salviae* | Leucinum | Q8TAA3 | PSMA8 |
| **937** | *Radix Salviae* | Leucinum | P55786 | NPEPPS |
| **938** | *Radix Salviae* | Leucinum | Q9UHI5 | SLC7A8 |
| **939** | *Radix Salviae* | Leucinum | P27797 | CALR |
| **940** | *Radix Salviae* | Leucinum | O15382 | BCAT2 |
| **941** | *Radix Salviae* | Leucinum | Q96N87 | SLC6A18 |
| **942** | *Radix Salviae* | Leucinum | Q15046 | KARS |
| **943** | *Radix Salviae* | Leucinum | Q96KP4 | CNDP2 |
| **944** | *Radix Salviae* | Leucinum | Q92530 | PSMF1 |
| **945** | *Radix Salviae* | Leucinum | P60059 | SEC61G |
| **946** | *Radix Salviae* | Leucinum | P0CG48 | UBC |
| **947** | *Radix Salviae* | Leucinum | Q7L5Y1 | ENOSF1 |
| **948** | *Radix Salviae* | Leucinum | P02751 | FN1 |
| **949** | *Radix Salviae* | Leucinum | O00232 | PSMD12 |
| **950** | *Radix Salviae* | Leucinum | P12931 | SRC |
| **951** | *Radix Salviae* | Leucinum | P40306 | PSMB10 |
| **952** | *Radix Salviae* | Leucinum | P17693 | HLA-G |
| **953** | *Radix Salviae* | Leucinum | P45983 | MAPK8 |
| **954** | *Radix Salviae* | Leucinum | P42345 | MTOR |
| **955** | *Radix Salviae* | Leucinum | P28074 | PSMB5 |
| **956** | *Radix Salviae* | Leucinum | P07814 | EPRS |
| **957** | *Radix Salviae* | Leucinum | P55036 | PSMD4 |
| **958** | *Radix Salviae* | Leucinum | P25774 | CTSS |
| **959** | *Radix Salviae* | Leucinum | O14818 | PSMA7 |
| **960** | *Radix Salviae* | Leucinum | P05412 | JUN |
| **961** | *Radix Salviae* | Leucinum | P49721 | PSMB2 |
| **962** | *Radix Salviae* | Leucinum | O00206 | TLR4 |
| **963** | *Radix Salviae* | Leucinum | P41252 | IARS |
| **964** | *Radix Salviae* | Leucinum | P29144 | TPP2 |
| **965** | *Radix Salviae* | Leucinum | P30499 | HLA-C |
| **966** | *Radix Salviae* | Leucinum | P30443 | HLA-A |
| **967** | *Radix Salviae* | Leucinum | P08195 | SLC3A2 |
| **968** | *Radix Salviae* | Leucinum | P01106 | MYC |
| **969** | *Radix Salviae* | Leucinum | O43324 | EEF1E1 |
| **970** | *Radix Salviae* | Leucinum | Q06323 | PSME1 |
| **971** | *Radix Salviae* | Leucinum | Q9P2J5 | LARS |
| **972** | *Radix Salviae* | Leucinum | Q12904 | AIMP1 |
| **973** | *Radix Salviae* | Leucinum | Q16236 | NFE2L2 |
| **974** | *Radix Salviae* | Leucinum | Q14997 | PSME4 |
| **975** | *Radix Salviae* | Leucinum | A5LHX3 | PSMB11 |
| **976** | *Radix Salviae* | Leucinum | O00487 | PSMD14 |
| **977** | *Radix Salviae* | Leucinum | P01275 | GCG |
| **978** | *Radix Salviae* | Leucinum | P62987 | UBA52 |
| **979** | *Radix Salviae* | Leucinum | Q9UNM6 | PSMD13 |
| **980** | *Radix Salviae* | Leucinum | P01375 | TNF |
| **981** | *Radix Salviae* | Leucinum | P01889 | HLA-B |
| **982** | *Radix Salviae* | Leucinum | Q6P179 | ERAP2 |
| **983** | *Radix Salviae* | Leucinum | P62333 | PSMC6 |
| **984** | *Radix Salviae* | Leucinum | P15151 | PVR |
| **985** | *Radix Salviae* | Leucinum | P17252 | PRKCA |
| **986** | *Radix Salviae* | Leucinum | P25786 | PSMA1 |
| **987** | *Radix Salviae* | Leucinum | O00233 | PSMD9 |
| **988** | *Radix Salviae* | Leucinum | P54687 | BCAT1 |
| **989** | *Radix Salviae* | Leucinum | P61769 | B2M |
| **990** | *Radix Salviae* | L-Ile | P25789 | PSMA4 |
| **991** | *Radix Salviae* | L-Ile | P43686 | PSMC4 |
| **992** | *Radix Salviae* | L-Ile | P09622 | DLD |
| **993** | *Radix Salviae* | L-Ile | Q16401 | PSMD5 |
| **994** | *Radix Salviae* | L-Ile | P48556 | PSMD8 |
| **995** | *Radix Salviae* | L-Ile | P25788 | PSMA3 |
| **996** | *Radix Salviae* | L-Ile | Q9UL46 | PSME2 |
| **997** | *Radix Salviae* | L-Ile | O75832 | PSMD10 |
| **998** | *Radix Salviae* | L-Ile | P51665 | PSMD7 |
| **999** | *Radix Salviae* | L-Ile | Q13155 | AIMP2 |
| **1000** | *Radix Salviae* | L-Ile | P60468 | SEC61B |
| **1001** | *Radix Salviae* | L-Ile | Q9UIQ6 | LNPEP |
| **1002** | *Radix Salviae* | L-Ile | P54136 | RARS |
| **1003** | *Radix Salviae* | L-Ile | P61619 | SEC61A1 |
| **1004** | *Radix Salviae* | L-Ile | Q99436 | PSMB7 |
| **1005** | *Radix Salviae* | L-Ile | P62191 | PSMC1 |
| **1006** | *Radix Salviae* | L-Ile | P60900 | PSMA6 |
| **1007** | *Radix Salviae* | L-Ile | Q01650 | SLC7A5 |
| **1008** | *Radix Salviae* | L-Ile | O00231 | PSMD11 |
| **1009** | *Radix Salviae* | L-Ile | Q13867 | BLMH |
| **1010** | *Radix Salviae* | L-Ile | P56192 | MARS |
| **1011** | *Radix Salviae* | L-Ile | P20618 | PSMB1 |
| **1012** | *Radix Salviae* | L-Ile | P14868 | DARS |
| **1013** | *Radix Salviae* | L-Ile | O43242 | PSMD3 |
| **1014** | *Radix Salviae* | L-Ile | Q9H2J7 | SLC6A15 |
| **1015** | *Radix Salviae* | L-Ile | P28072 | PSMB6 |
| **1016** | *Radix Salviae* | L-Ile | P28066 | PSMA5 |
| **1017** | *Radix Salviae* | L-Ile | P62979 | RPS27A |
| **1018** | *Radix Salviae* | L-Ile | O75223 | GGCT |
| **1019** | *Radix Salviae* | L-Ile | O60235 | TMPRSS11D |
| **1020** | *Radix Salviae* | L-Ile | P05067 | APP |
| **1021** | *Radix Salviae* | L-Ile | P28070 | PSMB4 |
| **1022** | *Radix Salviae* | L-Ile | P35998 | PSMC2 |
| **1023** | *Radix Salviae* | L-Ile | P61289 | PSME3 |
| **1024** | *Radix Salviae* | L-Ile | Q15008 | PSMD6 |
| **1025** | *Radix Salviae* | L-Ile | Q9NZ08 | ERAP1 |
| **1026** | *Radix Salviae* | L-Ile | Q9H9S3 | SEC61A2 |
| **1027** | *Radix Salviae* | L-Ile | P17980 | PSMC3 |
| **1028** | *Radix Salviae* | L-Ile | P30101 | PDIA3 |
| **1029** | *Radix Salviae* | L-Ile | P0CG47 | UBB |
| **1030** | *Radix Salviae* | L-Ile | P47897 | QARS |
| **1031** | *Radix Salviae* | L-Ile | P07477 | PRSS1 |
| **1032** | *Radix Salviae* | L-Ile | Q99460 | PSMD1 |
| **1033** | *Radix Salviae* | L-Ile | Q13200 | PSMD2 |
| **1034** | *Radix Salviae* | L-Ile | P62195 | PSMC5 |
| **1035** | *Radix Salviae* | L-Ile | Q8TAA3 | PSMA8 |
| **1036** | *Radix Salviae* | L-Ile | P55786 | NPEPPS |
| **1037** | *Radix Salviae* | L-Ile | Q9UHI5 | SLC7A8 |
| **1038** | *Radix Salviae* | L-Ile | P27797 | CALR |
| **1039** | *Radix Salviae* | L-Ile | O15382 | BCAT2 |
| **1040** | *Radix Salviae* | L-Ile | Q96N87 | SLC6A18 |
| **1041** | *Radix Salviae* | L-Ile | Q15046 | KARS |
| **1042** | *Radix Salviae* | L-Ile | Q96KP4 | CNDP2 |
| **1043** | *Radix Salviae* | L-Ile | Q92530 | PSMF1 |
| **1044** | *Radix Salviae* | L-Ile | P60059 | SEC61G |
| **1045** | *Radix Salviae* | L-Ile | Q96RQ9 | IL4I1 |
| **1046** | *Radix Salviae* | L-Ile | P0CG48 | UBC |
| **1047** | *Radix Salviae* | L-Ile | Q7L5Y1 | ENOSF1 |
| **1048** | *Radix Salviae* | L-Ile | O00232 | PSMD12 |
| **1049** | *Radix Salviae* | L-Ile | P40306 | PSMB10 |
| **1050** | *Radix Salviae* | L-Ile | P17693 | HLA-G |
| **1051** | *Radix Salviae* | L-Ile | P35030 | PRSS3 |
| **1052** | *Radix Salviae* | L-Ile | P28074 | PSMB5 |
| **1053** | *Radix Salviae* | L-Ile | P07814 | EPRS |
| **1054** | *Radix Salviae* | L-Ile | P55036 | PSMD4 |
| **1055** | *Radix Salviae* | L-Ile | P25774 | CTSS |
| **1056** | *Radix Salviae* | L-Ile | O14818 | PSMA7 |
| **1057** | *Radix Salviae* | L-Ile | P49721 | PSMB2 |
| **1058** | *Radix Salviae* | L-Ile | P41252 | IARS |
| **1059** | *Radix Salviae* | L-Ile | P29144 | TPP2 |
| **1060** | *Radix Salviae* | L-Ile | P30499 | HLA-C |
| **1061** | *Radix Salviae* | L-Ile | P30443 | HLA-A |
| **1062** | *Radix Salviae* | L-Ile | P08195 | SLC3A2 |
| **1063** | *Radix Salviae* | L-Ile | O43324 | EEF1E1 |
| **1064** | *Radix Salviae* | L-Ile | Q06323 | PSME1 |
| **1065** | *Radix Salviae* | L-Ile | Q9P2J5 | LARS |
| **1066** | *Radix Salviae* | L-Ile | Q12904 | AIMP1 |
| **1067** | *Radix Salviae* | L-Ile | Q14997 | PSME4 |
| **1068** | *Radix Salviae* | L-Ile | A5LHX3 | PSMB11 |
| **1069** | *Radix Salviae* | L-Ile | O00487 | PSMD14 |
| **1070** | *Radix Salviae* | L-Ile | P62987 | UBA52 |
| **1071** | *Radix Salviae* | L-Ile | Q9UNM6 | PSMD13 |
| **1072** | *Radix Salviae* | L-Ile | P01889 | HLA-B |
| **1073** | *Radix Salviae* | L-Ile | Q6P179 | ERAP2 |
| **1074** | *Radix Salviae* | L-Ile | P62333 | PSMC6 |
| **1075** | *Radix Salviae* | L-Ile | P15151 | PVR |
| **1076** | *Radix Salviae* | L-Ile | P25786 | PSMA1 |
| **1077** | *Radix Salviae* | L-Ile | O00233 | PSMD9 |
| **1078** | *Radix Salviae* | L-Ile | P00739 | HPR |
| **1079** | *Radix Salviae* | L-Ile | P54687 | BCAT1 |
| **1080** | *Radix Salviae* | L-Ile | P61769 | B2M |
| **1081** | *Radix Salviae* | L-Lysin | P01730 | CD4 |
| **1082** | *Radix Salviae* | L-Lysin | P25789 | PSMA4 |
| **1083** | *Radix Salviae* | L-Lysin | P43686 | PSMC4 |
| **1084** | *Radix Salviae* | L-Lysin | P03372 | ESR1 |
| **1085** | *Radix Salviae* | L-Lysin | Q16401 | PSMD5 |
| **1086** | *Radix Salviae* | L-Lysin | Q96EB6 | SIRT1 |
| **1087** | *Radix Salviae* | L-Lysin | P48556 | PSMD8 |
| **1088** | *Radix Salviae* | L-Lysin | P25788 | PSMA3 |
| **1089** | *Radix Salviae* | L-Lysin | P46663 | BDKRB1 |
| **1090** | *Radix Salviae* | L-Lysin | Q9UL46 | PSME2 |
| **1091** | *Radix Salviae* | L-Lysin | O75832 | PSMD10 |
| **1092** | *Radix Salviae* | L-Lysin | P51665 | PSMD7 |
| **1093** | *Radix Salviae* | L-Lysin | P30519 | HMOX2 |
| **1094** | *Radix Salviae* | L-Lysin | P00750 | PLAT |
| **1095** | *Radix Salviae* | L-Lysin | Q13155 | AIMP2 |
| **1096** | *Radix Salviae* | L-Lysin | P60468 | SEC61B |
| **1097** | *Radix Salviae* | L-Lysin | Q9UIQ6 | LNPEP |
| **1098** | *Radix Salviae* | L-Lysin | P54136 | RARS |
| **1099** | *Radix Salviae* | L-Lysin | Q9UNQ0 | ABCG2 |
| **1100** | *Radix Salviae* | L-Lysin | P04040 | CAT |
| **1101** | *Radix Salviae* | L-Lysin | P61619 | SEC61A1 |
| **1102** | *Radix Salviae* | L-Lysin | Q99705 | MCHR1 |
| **1103** | *Radix Salviae* | L-Lysin | P23946 | CMA1 |
| **1104** | *Radix Salviae* | L-Lysin | Q9GZQ4 | NMUR2 |
| **1105** | *Radix Salviae* | L-Lysin | P04083 | ANXA1 |
| **1106** | *Radix Salviae* | L-Lysin | Q99436 | PSMB7 |
| **1107** | *Radix Salviae* | L-Lysin | O60603 | TLR2 |
| **1108** | *Radix Salviae* | L-Lysin | P62191 | PSMC1 |
| **1109** | *Radix Salviae* | L-Lysin | P60900 | PSMA6 |
| **1110** | *Radix Salviae* | L-Lysin | O00231 | PSMD11 |
| **1111** | *Radix Salviae* | L-Lysin | Q13867 | BLMH |
| **1112** | *Radix Salviae* | L-Lysin | P56192 | MARS |
| **1113** | *Radix Salviae* | L-Lysin | P20618 | PSMB1 |
| **1114** | *Radix Salviae* | L-Lysin | P27361 | MAPK3 |
| **1115** | *Radix Salviae* | L-Lysin | P42336 | PIK3CA |
| **1116** | *Radix Salviae* | L-Lysin | P14868 | DARS |
| **1117** | *Radix Salviae* | L-Lysin | P48645 | NMU |
| **1118** | *Radix Salviae* | L-Lysin | O43242 | PSMD3 |
| **1119** | *Radix Salviae* | L-Lysin | P40763 | STAT3 |
| **1120** | *Radix Salviae* | L-Lysin | P01042 | KNG1 |
| **1121** | *Radix Salviae* | L-Lysin | Q07075 | ENPEP |
| **1122** | *Radix Salviae* | L-Lysin | P06400 | RB1 |
| **1123** | *Radix Salviae* | L-Lysin | P04637 | TP53 |
| **1124** | *Radix Salviae* | L-Lysin | P04626 | ERBB2 |
| **1125** | *Radix Salviae* | L-Lysin | P31749 | AKT1 |
| **1126** | *Radix Salviae* | L-Lysin | P28072 | PSMB6 |
| **1127** | *Radix Salviae* | L-Lysin | P28066 | PSMA5 |
| **1128** | *Radix Salviae* | L-Lysin | P62979 | RPS27A |
| **1129** | *Radix Salviae* | L-Lysin | Q13304 | GPR17 |
| **1130** | *Radix Salviae* | L-Lysin | O75223 | GGCT |
| **1131** | *Radix Salviae* | L-Lysin | P00533 | EGFR |
| **1132** | *Radix Salviae* | L-Lysin | Q13315 | ATM |
| **1133** | *Radix Salviae* | L-Lysin | Q969V1 | MCHR2 |
| **1134** | *Radix Salviae* | L-Lysin | O60235 | TMPRSS11D |
| **1135** | *Radix Salviae* | L-Lysin | P05067 | APP |
| **1136** | *Radix Salviae* | L-Lysin | P28070 | PSMB4 |
| **1137** | *Radix Salviae* | L-Lysin | P35998 | PSMC2 |
| **1138** | *Radix Salviae* | L-Lysin | P61289 | PSME3 |
| **1139** | *Radix Salviae* | L-Lysin | Q9UNI1 | CELA1 |
| **1140** | *Radix Salviae* | L-Lysin | P02768 | ALB |
| **1141** | *Radix Salviae* | L-Lysin | Q15008 | PSMD6 |
| **1142** | *Radix Salviae* | L-Lysin | Q9NZ08 | ERAP1 |
| **1143** | *Radix Salviae* | L-Lysin | Q9H9S3 | SEC61A2 |
| **1144** | *Radix Salviae* | L-Lysin | P17980 | PSMC3 |
| **1145** | *Radix Salviae* | L-Lysin | P30101 | PDIA3 |
| **1146** | *Radix Salviae* | L-Lysin | Q07817 | BCL2L1 |
| **1147** | *Radix Salviae* | L-Lysin | P34932 | HSPA4 |
| **1148** | *Radix Salviae* | L-Lysin | P0CG47 | UBB |
| **1149** | *Radix Salviae* | L-Lysin | P35568 | IRS1 |
| **1150** | *Radix Salviae* | L-Lysin | Q9HB89 | NMUR1 |
| **1151** | *Radix Salviae* | L-Lysin | P47897 | QARS |
| **1152** | *Radix Salviae* | L-Lysin | P99999 | CYCS |
| **1153** | *Radix Salviae* | L-Lysin | P15104 | GLUL |
| **1154** | *Radix Salviae* | L-Lysin | P07477 | PRSS1 |
| **1155** | *Radix Salviae* | L-Lysin | Q99460 | PSMD1 |
| **1156** | *Radix Salviae* | L-Lysin | Q13200 | PSMD2 |
| **1157** | *Radix Salviae* | L-Lysin | P62195 | PSMC5 |
| **1158** | *Radix Salviae* | L-Lysin | P42574 | CASP3 |
| **1159** | *Radix Salviae* | L-Lysin | Q8TAA3 | PSMA8 |
| **1160** | *Radix Salviae* | L-Lysin | P55786 | NPEPPS |
| **1161** | *Radix Salviae* | L-Lysin | P27797 | CALR |
| **1162** | *Radix Salviae* | L-Lysin | Q15046 | KARS |
| **1163** | *Radix Salviae* | L-Lysin | Q96KP4 | CNDP2 |
| **1164** | *Radix Salviae* | L-Lysin | Q92530 | PSMF1 |
| **1165** | *Radix Salviae* | L-Lysin | Q9H1C0 | LPAR5 |
| **1166** | *Radix Salviae* | L-Lysin | P30536 | TSPO |
| **1167** | *Radix Salviae* | L-Lysin | P20382 | PMCH |
| **1168** | *Radix Salviae* | L-Lysin | P49795 | RGS19 |
| **1169** | *Radix Salviae* | L-Lysin | P59768 | GNG2 |
| **1170** | *Radix Salviae* | L-Lysin | P25090 | FPR2 |
| **1171** | *Radix Salviae* | L-Lysin | P60059 | SEC61G |
| **1172** | *Radix Salviae* | L-Lysin | P0CG48 | UBC |
| **1173** | *Radix Salviae* | L-Lysin | Q7L5Y1 | ENOSF1 |
| **1174** | *Radix Salviae* | L-Lysin | P02751 | FN1 |
| **1175** | *Radix Salviae* | L-Lysin | O00232 | PSMD12 |
| **1176** | *Radix Salviae* | L-Lysin | P0DJI8 | SAA1 |
| **1177** | *Radix Salviae* | L-Lysin | P12931 | SRC |
| **1178** | *Radix Salviae* | L-Lysin | P40306 | PSMB10 |
| **1179** | *Radix Salviae* | L-Lysin | Q92633 | LPAR1 |
| **1180** | *Radix Salviae* | L-Lysin | P17693 | HLA-G |
| **1181** | *Radix Salviae* | L-Lysin | P45983 | MAPK8 |
| **1182** | *Radix Salviae* | L-Lysin | P35030 | PRSS3 |
| **1183** | *Radix Salviae* | L-Lysin | Q8N5D0 | WDTC1 |
| **1184** | *Radix Salviae* | L-Lysin | P28074 | PSMB5 |
| **1185** | *Radix Salviae* | L-Lysin | P01019 | AGT |
| **1186** | *Radix Salviae* | L-Lysin | P07814 | EPRS |
| **1187** | *Radix Salviae* | L-Lysin | Q9NS28 | RGS18 |
| **1188** | *Radix Salviae* | L-Lysin | P55036 | PSMD4 |
| **1189** | *Radix Salviae* | L-Lysin | P25774 | CTSS |
| **1190** | *Radix Salviae* | L-Lysin | Q9UBY5 | LPAR3 |
| **1191** | *Radix Salviae* | L-Lysin | O14818 | PSMA7 |
| **1192** | *Radix Salviae* | L-Lysin | P05412 | JUN |
| **1193** | *Radix Salviae* | L-Lysin | P00749 | PLAU |
| **1194** | *Radix Salviae* | L-Lysin | P49721 | PSMB2 |
| **1195** | *Radix Salviae* | L-Lysin | O00206 | TLR4 |
| **1196** | *Radix Salviae* | L-Lysin | P41252 | IARS |
| **1197** | *Radix Salviae* | L-Lysin | P29144 | TPP2 |
| **1198** | *Radix Salviae* | L-Lysin | P30499 | HLA-C |
| **1199** | *Radix Salviae* | L-Lysin | P30443 | HLA-A |
| **1200** | *Radix Salviae* | L-Lysin | Q5H8A3 | NMS |
| **1201** | *Radix Salviae* | L-Lysin | P01106 | MYC |
| **1202** | *Radix Salviae* | L-Lysin | P05305 | EDN1 |
| **1203** | *Radix Salviae* | L-Lysin | O43324 | EEF1E1 |
| **1204** | *Radix Salviae* | L-Lysin | P01185 | AVP |
| **1205** | *Radix Salviae* | L-Lysin | Q06323 | PSME1 |
| **1206** | *Radix Salviae* | L-Lysin | Q9P2J5 | LARS |
| **1207** | *Radix Salviae* | L-Lysin | Q12904 | AIMP1 |
| **1208** | *Radix Salviae* | L-Lysin | P06493 | CDK1 |
| **1209** | *Radix Salviae* | L-Lysin | Q14997 | PSME4 |
| **1210** | *Radix Salviae* | L-Lysin | Q9HBW0 | LPAR2 |
| **1211** | *Radix Salviae* | L-Lysin | A5LHX3 | PSMB11 |
| **1212** | *Radix Salviae* | L-Lysin | O00487 | PSMD14 |
| **1213** | *Radix Salviae* | L-Lysin | P01275 | GCG |
| **1214** | *Radix Salviae* | L-Lysin | P62987 | UBA52 |
| **1215** | *Radix Salviae* | L-Lysin | P21731 | TBXA2R |
| **1216** | *Radix Salviae* | L-Lysin | P35372 | OPRM1 |
| **1217** | *Radix Salviae* | L-Lysin | Q02880 | TOP2B |
| **1218** | *Radix Salviae* | L-Lysin | Q9UNM6 | PSMD13 |
| **1219** | *Radix Salviae* | L-Lysin | P01375 | TNF |
| **1220** | *Radix Salviae* | L-Lysin | P01889 | HLA-B |
| **1221** | *Radix Salviae* | L-Lysin | Q6P179 | ERAP2 |
| **1222** | *Radix Salviae* | L-Lysin | P62333 | PSMC6 |
| **1223** | *Radix Salviae* | L-Lysin | P15151 | PVR |
| **1224** | *Radix Salviae* | L-Lysin | P11388 | TOP2A |
| **1225** | *Radix Salviae* | L-Lysin | P25786 | PSMA1 |
| **1226** | *Radix Salviae* | L-Lysin | O00233 | PSMD9 |
| **1227** | *Radix Salviae* | L-Lysin | P00739 | HPR |
| **1228** | *Radix Salviae* | L-Lysin | P61769 | B2M |
| **1229** | *Radix Salviae* | L-Lysin | P40313 | CTRL |
| **1230** | *Radix Salviae* | LPG | P01730 | CD4 |
| **1231** | *Radix Salviae* | LPG | P25789 | PSMA4 |
| **1232** | *Radix Salviae* | LPG | P43686 | PSMC4 |
| **1233** | *Radix Salviae* | LPG | Q16401 | PSMD5 |
| **1234** | *Radix Salviae* | LPG | P48556 | PSMD8 |
| **1235** | *Radix Salviae* | LPG | P25788 | PSMA3 |
| **1236** | *Radix Salviae* | LPG | Q9UL46 | PSME2 |
| **1237** | *Radix Salviae* | LPG | O75832 | PSMD10 |
| **1238** | *Radix Salviae* | LPG | P51665 | PSMD7 |
| **1239** | *Radix Salviae* | LPG | P00390 | GSR |
| **1240** | *Radix Salviae* | LPG | Q13155 | AIMP2 |
| **1241** | *Radix Salviae* | LPG | P60468 | SEC61B |
| **1242** | *Radix Salviae* | LPG | P30838 | ALDH3A1 |
| **1243** | *Radix Salviae* | LPG | P24385 | CCND1 |
| **1244** | *Radix Salviae* | LPG | Q9UIQ6 | LNPEP |
| **1245** | *Radix Salviae* | LPG | P04040 | CAT |
| **1246** | *Radix Salviae* | LPG | P61619 | SEC61A1 |
| **1247** | *Radix Salviae* | LPG | P00505 | GOT2 |
| **1248** | *Radix Salviae* | LPG | O94788 | ALDH1A2 |
| **1249** | *Radix Salviae* | LPG | P02649 | APOE |
| **1250** | *Radix Salviae* | LPG | P48448 | ALDH3B2 |
| **1251** | *Radix Salviae* | LPG | P01116 | KRAS |
| **1252** | *Radix Salviae* | LPG | Q3SY69 | ALDH1L2 |
| **1253** | *Radix Salviae* | LPG | P05231 | IL6 |
| **1254** | *Radix Salviae* | LPG | Q99436 | PSMB7 |
| **1255** | *Radix Salviae* | LPG | P62191 | PSMC1 |
| **1256** | *Radix Salviae* | LPG | P60900 | PSMA6 |
| **1257** | *Radix Salviae* | LPG | O00231 | PSMD11 |
| **1258** | *Radix Salviae* | LPG | Q13867 | BLMH |
| **1259** | *Radix Salviae* | LPG | P05091 | ALDH2 |
| **1260** | *Radix Salviae* | LPG | P20618 | PSMB1 |
| **1261** | *Radix Salviae* | LPG | P06276 | BCHE |
| **1262** | *Radix Salviae* | LPG | O43242 | PSMD3 |
| **1263** | *Radix Salviae* | LPG | P01189 | POMC |
| **1264** | *Radix Salviae* | LPG | Q07075 | ENPEP |
| **1265** | *Radix Salviae* | LPG | Q9H2A2 | ALDH8A1 |
| **1266** | *Radix Salviae* | LPG | Q9H2J7 | SLC6A15 |
| **1267** | *Radix Salviae* | LPG | P04637 | TP53 |
| **1268** | *Radix Salviae* | LPG | P31749 | AKT1 |
| **1269** | *Radix Salviae* | LPG | Q01959 | SLC6A3 |
| **1270** | *Radix Salviae* | LPG | P28072 | PSMB6 |
| **1271** | *Radix Salviae* | LPG | P28066 | PSMA5 |
| **1272** | *Radix Salviae* | LPG | P62979 | RPS27A |
| **1273** | *Radix Salviae* | LPG | O75223 | GGCT |
| **1274** | *Radix Salviae* | LPG | Q13315 | ATM |
| **1275** | *Radix Salviae* | LPG | O60235 | TMPRSS11D |
| **1276** | *Radix Salviae* | LPG | P28070 | PSMB4 |
| **1277** | *Radix Salviae* | LPG | P30038 | ALDH4A1 |
| **1278** | *Radix Salviae* | LPG | P35998 | PSMC2 |
| **1279** | *Radix Salviae* | LPG | Q8IZ83 | ALDH16A1 |
| **1280** | *Radix Salviae* | LPG | P61289 | PSME3 |
| **1281** | *Radix Salviae* | LPG | Q9UNI1 | CELA1 |
| **1282** | *Radix Salviae* | LPG | O60760 | HPGDS |
| **1283** | *Radix Salviae* | LPG | P02768 | ALB |
| **1284** | *Radix Salviae* | LPG | Q15008 | PSMD6 |
| **1285** | *Radix Salviae* | LPG | Q9NZ08 | ERAP1 |
| **1286** | *Radix Salviae* | LPG | P29474 | NOS3 |
| **1287** | *Radix Salviae* | LPG | P00352 | ALDH1A1 |
| **1288** | *Radix Salviae* | LPG | Q9H9S3 | SEC61A2 |
| **1289** | *Radix Salviae* | LPG | P17980 | PSMC3 |
| **1290** | *Radix Salviae* | LPG | P30101 | PDIA3 |
| **1291** | *Radix Salviae* | LPG | P0CG47 | UBB |
| **1292** | *Radix Salviae* | LPG | P35568 | IRS1 |
| **1293** | *Radix Salviae* | LPG | P15104 | GLUL |
| **1294** | *Radix Salviae* | LPG | Q99460 | PSMD1 |
| **1295** | *Radix Salviae* | LPG | Q13200 | PSMD2 |
| **1296** | *Radix Salviae* | LPG | P62195 | PSMC5 |
| **1297** | *Radix Salviae* | LPG | Q8TAA3 | PSMA8 |
| **1298** | *Radix Salviae* | LPG | P51649 | ALDH5A1 |
| **1299** | *Radix Salviae* | LPG | P55786 | NPEPPS |
| **1300** | *Radix Salviae* | LPG | Q9UHI5 | SLC7A8 |
| **1301** | *Radix Salviae* | LPG | P27797 | CALR |
| **1302** | *Radix Salviae* | LPG | Q96N87 | SLC6A18 |
| **1303** | *Radix Salviae* | LPG | Q15046 | KARS |
| **1304** | *Radix Salviae* | LPG | Q96KP4 | CNDP2 |
| **1305** | *Radix Salviae* | LPG | Q92530 | PSMF1 |
| **1306** | *Radix Salviae* | LPG | P30536 | TSPO |
| **1307** | *Radix Salviae* | LPG | P47895 | ALDH1A3 |
| **1308** | *Radix Salviae* | LPG | P60059 | SEC61G |
| **1309** | *Radix Salviae* | LPG | Q96RQ9 | IL4I1 |
| **1310** | *Radix Salviae* | LPG | P0CG48 | UBC |
| **1311** | *Radix Salviae* | LPG | P51648 | ALDH3A2 |
| **1312** | *Radix Salviae* | LPG | Q7L5Y1 | ENOSF1 |
| **1313** | *Radix Salviae* | LPG | P49189 | ALDH9A1 |
| **1314** | *Radix Salviae* | LPG | O00232 | PSMD12 |
| **1315** | *Radix Salviae* | LPG | Q42521 | GAD1 |
| **1316** | *Radix Salviae* | LPG | P40306 | PSMB10 |
| **1317** | *Radix Salviae* | LPG | P17693 | HLA-G |
| **1318** | *Radix Salviae* | LPG | P11387 | TOP1 |
| **1319** | *Radix Salviae* | LPG | P28074 | PSMB5 |
| **1320** | *Radix Salviae* | LPG | P55036 | PSMD4 |
| **1321** | *Radix Salviae* | LPG | P25774 | CTSS |
| **1322** | *Radix Salviae* | LPG | P17174 | GOT1 |
| **1323** | *Radix Salviae* | LPG | O14818 | PSMA7 |
| **1324** | *Radix Salviae* | LPG | P05412 | JUN |
| **1325** | *Radix Salviae* | LPG | P60484 | PTEN |
| **1326** | *Radix Salviae* | LPG | P00749 | PLAU |
| **1327** | *Radix Salviae* | LPG | P49721 | PSMB2 |
| **1328** | *Radix Salviae* | LPG | P29144 | TPP2 |
| **1329** | *Radix Salviae* | LPG | P30499 | HLA-C |
| **1330** | *Radix Salviae* | LPG | P30443 | HLA-A |
| **1331** | *Radix Salviae* | LPG | P30837 | ALDH1B1 |
| **1332** | *Radix Salviae* | LPG | P08195 | SLC3A2 |
| **1333** | *Radix Salviae* | LPG | Q06323 | PSME1 |
| **1334** | *Radix Salviae* | LPG | O75891 | ALDH1L1 |
| **1335** | *Radix Salviae* | LPG | Q14997 | PSME4 |
| **1336** | *Radix Salviae* | LPG | A5LHX3 | PSMB11 |
| **1337** | *Radix Salviae* | LPG | O00487 | PSMD14 |
| **1338** | *Radix Salviae* | LPG | P49419 | ALDH7A1 |
| **1339** | *Radix Salviae* | LPG | P01275 | GCG |
| **1340** | *Radix Salviae* | LPG | Q5TF58 | IFFO2 |
| **1341** | *Radix Salviae* | LPG | P62987 | UBA52 |
| **1342** | *Radix Salviae* | LPG | Q9UNM6 | PSMD13 |
| **1343** | *Radix Salviae* | LPG | P01889 | HLA-B |
| **1344** | *Radix Salviae* | LPG | Q6P179 | ERAP2 |
| **1345** | *Radix Salviae* | LPG | P62333 | PSMC6 |
| **1346** | *Radix Salviae* | LPG | P15151 | PVR |
| **1347** | *Radix Salviae* | LPG | P07203 | GPX1 |
| **1348** | *Radix Salviae* | LPG | P25786 | PSMA1 |
| **1349** | *Radix Salviae* | LPG | O00233 | PSMD9 |
| **1350** | *Radix Salviae* | LPG | P00739 | HPR |
| **1351** | *Radix Salviae* | LPG | P61769 | B2M |
| **1352** | *Radix Salviae* | LPG | P40313 | CTRL |
| **1353** | *Radix Salviae* | L-Serin | P25789 | PSMA4 |
| **1354** | *Radix Salviae* | L-Serin | P43686 | PSMC4 |
| **1355** | *Radix Salviae* | L-Serin | Q16401 | PSMD5 |
| **1356** | *Radix Salviae* | L-Serin | P48556 | PSMD8 |
| **1357** | *Radix Salviae* | L-Serin | P25788 | PSMA3 |
| **1358** | *Radix Salviae* | L-Serin | Q9UL46 | PSME2 |
| **1359** | *Radix Salviae* | L-Serin | O75832 | PSMD10 |
| **1360** | *Radix Salviae* | L-Serin | P51665 | PSMD7 |
| **1361** | *Radix Salviae* | L-Serin | P60468 | SEC61B |
| **1362** | *Radix Salviae* | L-Serin | P30838 | ALDH3A1 |
| **1363** | *Radix Salviae* | L-Serin | Q9UIQ6 | LNPEP |
| **1364** | *Radix Salviae* | L-Serin | P61619 | SEC61A1 |
| **1365** | *Radix Salviae* | L-Serin | O94788 | ALDH1A2 |
| **1366** | *Radix Salviae* | L-Serin | P02649 | APOE |
| **1367** | *Radix Salviae* | L-Serin | P48448 | ALDH3B2 |
| **1368** | *Radix Salviae* | L-Serin | Q3SY69 | ALDH1L2 |
| **1369** | *Radix Salviae* | L-Serin | Q99436 | PSMB7 |
| **1370** | *Radix Salviae* | L-Serin | P62191 | PSMC1 |
| **1371** | *Radix Salviae* | L-Serin | P60900 | PSMA6 |
| **1372** | *Radix Salviae* | L-Serin | O00231 | PSMD11 |
| **1373** | *Radix Salviae* | L-Serin | Q13867 | BLMH |
| **1374** | *Radix Salviae* | L-Serin | P05091 | ALDH2 |
| **1375** | *Radix Salviae* | L-Serin | P20618 | PSMB1 |
| **1376** | *Radix Salviae* | L-Serin | O43242 | PSMD3 |
| **1377** | *Radix Salviae* | L-Serin | Q07075 | ENPEP |
| **1378** | *Radix Salviae* | L-Serin | Q9H2A2 | ALDH8A1 |
| **1379** | *Radix Salviae* | L-Serin | Q9H2J7 | SLC6A15 |
| **1380** | *Radix Salviae* | L-Serin | P04637 | TP53 |
| **1381** | *Radix Salviae* | L-Serin | P28072 | PSMB6 |
| **1382** | *Radix Salviae* | L-Serin | P28066 | PSMA5 |
| **1383** | *Radix Salviae* | L-Serin | P62979 | RPS27A |
| **1384** | *Radix Salviae* | L-Serin | O75223 | GGCT |
| **1385** | *Radix Salviae* | L-Serin | Q13315 | ATM |
| **1386** | *Radix Salviae* | L-Serin | P28070 | PSMB4 |
| **1387** | *Radix Salviae* | L-Serin | P30038 | ALDH4A1 |
| **1388** | *Radix Salviae* | L-Serin | P35998 | PSMC2 |
| **1389** | *Radix Salviae* | L-Serin | Q8IZ83 | ALDH16A1 |
| **1390** | *Radix Salviae* | L-Serin | P61289 | PSME3 |
| **1391** | *Radix Salviae* | L-Serin | Q15008 | PSMD6 |
| **1392** | *Radix Salviae* | L-Serin | Q9NZ08 | ERAP1 |
| **1393** | *Radix Salviae* | L-Serin | P00352 | ALDH1A1 |
| **1394** | *Radix Salviae* | L-Serin | Q9H9S3 | SEC61A2 |
| **1395** | *Radix Salviae* | L-Serin | P17980 | PSMC3 |
| **1396** | *Radix Salviae* | L-Serin | P30101 | PDIA3 |
| **1397** | *Radix Salviae* | L-Serin | P0CG47 | UBB |
| **1398** | *Radix Salviae* | L-Serin | Q99460 | PSMD1 |
| **1399** | *Radix Salviae* | L-Serin | Q13200 | PSMD2 |
| **1400** | *Radix Salviae* | L-Serin | P62195 | PSMC5 |
| **1401** | *Radix Salviae* | L-Serin | Q8TAA3 | PSMA8 |
| **1402** | *Radix Salviae* | L-Serin | P51649 | ALDH5A1 |
| **1403** | *Radix Salviae* | L-Serin | P34896 | SHMT1 |
| **1404** | *Radix Salviae* | L-Serin | P55786 | NPEPPS |
| **1405** | *Radix Salviae* | L-Serin | Q9UHI5 | SLC7A8 |
| **1406** | *Radix Salviae* | L-Serin | P27797 | CALR |
| **1407** | *Radix Salviae* | L-Serin | Q96N87 | SLC6A18 |
| **1408** | *Radix Salviae* | L-Serin | Q96KP4 | CNDP2 |
| **1409** | *Radix Salviae* | L-Serin | Q92530 | PSMF1 |
| **1410** | *Radix Salviae* | L-Serin | P47895 | ALDH1A3 |
| **1411** | *Radix Salviae* | L-Serin | P60059 | SEC61G |
| **1412** | *Radix Salviae* | L-Serin | P0CG48 | UBC |
| **1413** | *Radix Salviae* | L-Serin | P51648 | ALDH3A2 |
| **1414** | *Radix Salviae* | L-Serin | Q7L5Y1 | ENOSF1 |
| **1415** | *Radix Salviae* | L-Serin | P49189 | ALDH9A1 |
| **1416** | *Radix Salviae* | L-Serin | O00232 | PSMD12 |
| **1417** | *Radix Salviae* | L-Serin | P40306 | PSMB10 |
| **1418** | *Radix Salviae* | L-Serin | P17693 | HLA-G |
| **1419** | *Radix Salviae* | L-Serin | P28074 | PSMB5 |
| **1420** | *Radix Salviae* | L-Serin | P55036 | PSMD4 |
| **1421** | *Radix Salviae* | L-Serin | P25774 | CTSS |
| **1422** | *Radix Salviae* | L-Serin | O43175 | PHGDH |
| **1423** | *Radix Salviae* | L-Serin | O14818 | PSMA7 |
| **1424** | *Radix Salviae* | L-Serin | P49721 | PSMB2 |
| **1425** | *Radix Salviae* | L-Serin | P29144 | TPP2 |
| **1426** | *Radix Salviae* | L-Serin | P30499 | HLA-C |
| **1427** | *Radix Salviae* | L-Serin | P30443 | HLA-A |
| **1428** | *Radix Salviae* | L-Serin | P30837 | ALDH1B1 |
| **1429** | *Radix Salviae* | L-Serin | P08195 | SLC3A2 |
| **1430** | *Radix Salviae* | L-Serin | Q06323 | PSME1 |
| **1431** | *Radix Salviae* | L-Serin | O75891 | ALDH1L1 |
| **1432** | *Radix Salviae* | L-Serin | Q14997 | PSME4 |
| **1433** | *Radix Salviae* | L-Serin | A5LHX3 | PSMB11 |
| **1434** | *Radix Salviae* | L-Serin | O00487 | PSMD14 |
| **1435** | *Radix Salviae* | L-Serin | P49419 | ALDH7A1 |
| **1436** | *Radix Salviae* | L-Serin | Q5TF58 | IFFO2 |
| **1437** | *Radix Salviae* | L-Serin | P62987 | UBA52 |
| **1438** | *Radix Salviae* | L-Serin | Q9UNM6 | PSMD13 |
| **1439** | *Radix Salviae* | L-Serin | P01889 | HLA-B |
| **1440** | *Radix Salviae* | L-Serin | Q6P179 | ERAP2 |
| **1441** | *Radix Salviae* | L-Serin | P62333 | PSMC6 |
| **1442** | *Radix Salviae* | L-Serin | P15151 | PVR |
| **1443** | *Radix Salviae* | L-Serin | P25786 | PSMA1 |
| **1444** | *Radix Salviae* | L-Serin | O00233 | PSMD9 |
| **1445** | *Radix Salviae* | L-Serin | P61769 | B2M |
| **1446** | *Radix Salviae* | luteolin | P28482 | MAPK1 |
| **1447** | *Radix Salviae* | luteolin | P09601 | HMOX1 |
| **1448** | *Radix Salviae* | luteolin | P01031 | C5 |
| **1449** | *Radix Salviae* | luteolin | P01024 | C3 |
| **1450** | *Radix Salviae* | luteolin | P27361 | MAPK3 |
| **1451** | *Radix Salviae* | luteolin | P01584 | IL1B |
| **1452** | *Radix Salviae* | luteolin | P40763 | STAT3 |
| **1453** | *Radix Salviae* | luteolin | Q9UBK8 | MTRR |
| **1454** | *Radix Salviae* | luteolin | P24941 | CDK2 |
| **1455** | *Radix Salviae* | luteolin | P04637 | TP53 |
| **1456** | *Radix Salviae* | luteolin | P04626 | ERBB2 |
| **1457** | *Radix Salviae* | luteolin | P31749 | AKT1 |
| **1458** | *Radix Salviae* | luteolin | P00533 | EGFR |
| **1459** | *Radix Salviae* | luteolin | O14763 | TNFRSF10B |
| **1460** | *Radix Salviae* | luteolin | P15121 | AKR1B1 |
| **1461** | *Radix Salviae* | luteolin | P37231 | PPARG |
| **1462** | *Radix Salviae* | luteolin | P29474 | NOS3 |
| **1463** | *Radix Salviae* | luteolin | Q07817 | BCL2L1 |
| **1464** | *Radix Salviae* | luteolin | P35568 | IRS1 |
| **1465** | *Radix Salviae* | luteolin | P01100 | FOS |
| **1466** | *Radix Salviae* | luteolin | P42574 | CASP3 |
| **1467** | *Radix Salviae* | luteolin | P35558 | PCK1 |
| **1468** | *Radix Salviae* | luteolin | P35228 | NOS2 |
| **1469** | *Radix Salviae* | luteolin | P55211 | CASP9 |
| **1470** | *Radix Salviae* | luteolin | Q05397 | PTK2 |
| **1471** | *Radix Salviae* | luteolin | Q92731 | ESR2 |
| **1472** | *Radix Salviae* | luteolin | P02751 | FN1 |
| **1473** | *Radix Salviae* | luteolin | P45983 | MAPK8 |
| **1474** | *Radix Salviae* | luteolin | P11387 | TOP1 |
| **1475** | *Radix Salviae* | luteolin | P42345 | MTOR |
| **1476** | *Radix Salviae* | luteolin | P01019 | AGT |
| **1477** | *Radix Salviae* | luteolin | P55210 | CASP7 |
| **1478** | *Radix Salviae* | luteolin | P05412 | JUN |
| **1479** | *Radix Salviae* | luteolin | P14780 | MMP9 |
| **1480** | *Radix Salviae* | luteolin | O00206 | TLR4 |
| **1481** | *Radix Salviae* | luteolin | P47989 | XDH |
| **1482** | *Radix Salviae* | luteolin | P04798 | CYP1A1 |
| **1483** | *Radix Salviae* | luteolin | Q16236 | NFE2L2 |
| **1484** | *Radix Salviae* | luteolin | P33527 | ABCC1 |
| **1485** | *Radix Salviae* | luteolin | P01375 | TNF |
| **1486** | *Radix Salviae* | luteolin | P35503 | UGT1A3 |
| **1487** | *Radix Salviae* | luteolin | P16435 | POR |
| **1488** | *Radix Salviae* | luteolin-7-o-glucoside | P09601 | HMOX1 |
| **1489** | *Radix Salviae* | luteolin-7-o-glucoside | Q9UBK8 | MTRR |
| **1490** | *Radix Salviae* | luteolin-7-o-glucoside | P29474 | NOS3 |
| **1491** | *Radix Salviae* | luteolin-7-o-glucoside | P35228 | NOS2 |
| **1492** | *Radix Salviae* | luteolin-7-o-glucoside | P16435 | POR |
| **1493** | *Radix Salviae* | L-Valin | P25789 | PSMA4 |
| **1494** | *Radix Salviae* | L-Valin | P43686 | PSMC4 |
| **1495** | *Radix Salviae* | L-Valin | P09622 | DLD |
| **1496** | *Radix Salviae* | L-Valin | Q16401 | PSMD5 |
| **1497** | *Radix Salviae* | L-Valin | P48556 | PSMD8 |
| **1498** | *Radix Salviae* | L-Valin | P25788 | PSMA3 |
| **1499** | *Radix Salviae* | L-Valin | Q9UL46 | PSME2 |
| **1500** | *Radix Salviae* | L-Valin | O75832 | PSMD10 |
| **1501** | *Radix Salviae* | L-Valin | P51665 | PSMD7 |
| **1502** | *Radix Salviae* | L-Valin | P60468 | SEC61B |
| **1503** | *Radix Salviae* | L-Valin | P30838 | ALDH3A1 |
| **1504** | *Radix Salviae* | L-Valin | Q9UIQ6 | LNPEP |
| **1505** | *Radix Salviae* | L-Valin | P61619 | SEC61A1 |
| **1506** | *Radix Salviae* | L-Valin | O94788 | ALDH1A2 |
| **1507** | *Radix Salviae* | L-Valin | P48448 | ALDH3B2 |
| **1508** | *Radix Salviae* | L-Valin | Q3SY69 | ALDH1L2 |
| **1509** | *Radix Salviae* | L-Valin | Q99436 | PSMB7 |
| **1510** | *Radix Salviae* | L-Valin | P62191 | PSMC1 |
| **1511** | *Radix Salviae* | L-Valin | P60900 | PSMA6 |
| **1512** | *Radix Salviae* | L-Valin | Q01650 | SLC7A5 |
| **1513** | *Radix Salviae* | L-Valin | O00231 | PSMD11 |
| **1514** | *Radix Salviae* | L-Valin | Q13867 | BLMH |
| **1515** | *Radix Salviae* | L-Valin | P05091 | ALDH2 |
| **1516** | *Radix Salviae* | L-Valin | P20618 | PSMB1 |
| **1517** | *Radix Salviae* | L-Valin | O43242 | PSMD3 |
| **1518** | *Radix Salviae* | L-Valin | Q9H2A2 | ALDH8A1 |
| **1519** | *Radix Salviae* | L-Valin | Q9H2J7 | SLC6A15 |
| **1520** | *Radix Salviae* | L-Valin | P28072 | PSMB6 |
| **1521** | *Radix Salviae* | L-Valin | P28066 | PSMA5 |
| **1522** | *Radix Salviae* | L-Valin | P62979 | RPS27A |
| **1523** | *Radix Salviae* | L-Valin | O75223 | GGCT |
| **1524** | *Radix Salviae* | L-Valin | O60235 | TMPRSS11D |
| **1525** | *Radix Salviae* | L-Valin | P05067 | APP |
| **1526** | *Radix Salviae* | L-Valin | P28070 | PSMB4 |
| **1527** | *Radix Salviae* | L-Valin | P30038 | ALDH4A1 |
| **1528** | *Radix Salviae* | L-Valin | P35998 | PSMC2 |
| **1529** | *Radix Salviae* | L-Valin | Q8IZ83 | ALDH16A1 |
| **1530** | *Radix Salviae* | L-Valin | P61289 | PSME3 |
| **1531** | *Radix Salviae* | L-Valin | Q15008 | PSMD6 |
| **1532** | *Radix Salviae* | L-Valin | Q9NZ08 | ERAP1 |
| **1533** | *Radix Salviae* | L-Valin | P00352 | ALDH1A1 |
| **1534** | *Radix Salviae* | L-Valin | Q9H9S3 | SEC61A2 |
| **1535** | *Radix Salviae* | L-Valin | P17980 | PSMC3 |
| **1536** | *Radix Salviae* | L-Valin | P30101 | PDIA3 |
| **1537** | *Radix Salviae* | L-Valin | P0CG47 | UBB |
| **1538** | *Radix Salviae* | L-Valin | P07477 | PRSS1 |
| **1539** | *Radix Salviae* | L-Valin | Q99460 | PSMD1 |
| **1540** | *Radix Salviae* | L-Valin | Q13200 | PSMD2 |
| **1541** | *Radix Salviae* | L-Valin | P62195 | PSMC5 |
| **1542** | *Radix Salviae* | L-Valin | Q8TAA3 | PSMA8 |
| **1543** | *Radix Salviae* | L-Valin | P51649 | ALDH5A1 |
| **1544** | *Radix Salviae* | L-Valin | P55786 | NPEPPS |
| **1545** | *Radix Salviae* | L-Valin | Q9UHI5 | SLC7A8 |
| **1546** | *Radix Salviae* | L-Valin | P27797 | CALR |
| **1547** | *Radix Salviae* | L-Valin | O15382 | BCAT2 |
| **1548** | *Radix Salviae* | L-Valin | Q96N87 | SLC6A18 |
| **1549** | *Radix Salviae* | L-Valin | Q96KP4 | CNDP2 |
| **1550** | *Radix Salviae* | L-Valin | Q92530 | PSMF1 |
| **1551** | *Radix Salviae* | L-Valin | P47895 | ALDH1A3 |
| **1552** | *Radix Salviae* | L-Valin | P60059 | SEC61G |
| **1553** | *Radix Salviae* | L-Valin | P0CG48 | UBC |
| **1554** | *Radix Salviae* | L-Valin | P51648 | ALDH3A2 |
| **1555** | *Radix Salviae* | L-Valin | Q7L5Y1 | ENOSF1 |
| **1556** | *Radix Salviae* | L-Valin | P49189 | ALDH9A1 |
| **1557** | *Radix Salviae* | L-Valin | O00232 | PSMD12 |
| **1558** | *Radix Salviae* | L-Valin | P40306 | PSMB10 |
| **1559** | *Radix Salviae* | L-Valin | P17693 | HLA-G |
| **1560** | *Radix Salviae* | L-Valin | P35030 | PRSS3 |
| **1561** | *Radix Salviae* | L-Valin | P28074 | PSMB5 |
| **1562** | *Radix Salviae* | L-Valin | P55036 | PSMD4 |
| **1563** | *Radix Salviae* | L-Valin | P25774 | CTSS |
| **1564** | *Radix Salviae* | L-Valin | O14818 | PSMA7 |
| **1565** | *Radix Salviae* | L-Valin | P49721 | PSMB2 |
| **1566** | *Radix Salviae* | L-Valin | P41252 | IARS |
| **1567** | *Radix Salviae* | L-Valin | P29144 | TPP2 |
| **1568** | *Radix Salviae* | L-Valin | P30499 | HLA-C |
| **1569** | *Radix Salviae* | L-Valin | P30443 | HLA-A |
| **1570** | *Radix Salviae* | L-Valin | P30837 | ALDH1B1 |
| **1571** | *Radix Salviae* | L-Valin | P08195 | SLC3A2 |
| **1572** | *Radix Salviae* | L-Valin | Q06323 | PSME1 |
| **1573** | *Radix Salviae* | L-Valin | O75891 | ALDH1L1 |
| **1574** | *Radix Salviae* | L-Valin | Q14997 | PSME4 |
| **1575** | *Radix Salviae* | L-Valin | A5LHX3 | PSMB11 |
| **1576** | *Radix Salviae* | L-Valin | O00487 | PSMD14 |
| **1577** | *Radix Salviae* | L-Valin | P49419 | ALDH7A1 |
| **1578** | *Radix Salviae* | L-Valin | Q5TF58 | IFFO2 |
| **1579** | *Radix Salviae* | L-Valin | P62987 | UBA52 |
| **1580** | *Radix Salviae* | L-Valin | Q9UNM6 | PSMD13 |
| **1581** | *Radix Salviae* | L-Valin | P01889 | HLA-B |
| **1582** | *Radix Salviae* | L-Valin | Q6P179 | ERAP2 |
| **1583** | *Radix Salviae* | L-Valin | P62333 | PSMC6 |
| **1584** | *Radix Salviae* | L-Valin | P15151 | PVR |
| **1585** | *Radix Salviae* | L-Valin | P25786 | PSMA1 |
| **1586** | *Radix Salviae* | L-Valin | O00233 | PSMD9 |
| **1587** | *Radix Salviae* | L-Valin | P54687 | BCAT1 |
| **1588** | *Radix Salviae* | L-Valin | P61769 | B2M |
| **1589** | *Radix Salviae* | miltirone | P33261 | CYP2C19 |
| **1590** | *Radix Salviae* | oleanolic acid | P43490 | NAMPT |
| **1591** | *Radix Salviae* | oleanolic acid | Q07869 | PPARA |
| **1592** | *Radix Salviae* | oleanolic acid | P36537 | UGT2B10 |
| **1593** | *Radix Salviae* | oleanolic acid | P42574 | CASP3 |
| **1594** | *Radix Salviae* | oleanolic acid | P15559 | NQO1 |
| **1595** | *Radix Salviae* | oleanolic acid | P08684 | CYP3A4 |
| **1596** | *Radix Salviae* | oleanolic acid | P05177 | CYP1A2 |
| **1597** | *Radix Salviae* | oleanolic acid | Q14790 | CASP8 |
| **1598** | *Radix Salviae* | oleanolic acid | O60218 | AKR1B10 |
| **1599** | *Radix Salviae* | oleanolic acid | P11387 | TOP1 |
| **1600** | *Radix Salviae* | oleanolic acid | Q16236 | NFE2L2 |
| **1601** | *Radix Salviae* | oleanolic acid | O75310 | UGT2B11 |
| **1602** | *Radix Salviae* | oleanolic acid | P11388 | TOP2A |
| **1603** | *Radix Salviae* | PHA | P25789 | PSMA4 |
| **1604** | *Radix Salviae* | PHA | P43686 | PSMC4 |
| **1605** | *Radix Salviae* | PHA | Q16401 | PSMD5 |
| **1606** | *Radix Salviae* | PHA | P48556 | PSMD8 |
| **1607** | *Radix Salviae* | PHA | P25788 | PSMA3 |
| **1608** | *Radix Salviae* | PHA | Q9UL46 | PSME2 |
| **1609** | *Radix Salviae* | PHA | O75832 | PSMD10 |
| **1610** | *Radix Salviae* | PHA | P51665 | PSMD7 |
| **1611** | *Radix Salviae* | PHA | P60468 | SEC61B |
| **1612** | *Radix Salviae* | PHA | Q9UIQ6 | LNPEP |
| **1613** | *Radix Salviae* | PHA | P61619 | SEC61A1 |
| **1614** | *Radix Salviae* | PHA | P00505 | GOT2 |
| **1615** | *Radix Salviae* | PHA | Q99436 | PSMB7 |
| **1616** | *Radix Salviae* | PHA | P62191 | PSMC1 |
| **1617** | *Radix Salviae* | PHA | P60900 | PSMA6 |
| **1618** | *Radix Salviae* | PHA | Q01650 | SLC7A5 |
| **1619** | *Radix Salviae* | PHA | O00231 | PSMD11 |
| **1620** | *Radix Salviae* | PHA | Q13867 | BLMH |
| **1621** | *Radix Salviae* | PHA | P20618 | PSMB1 |
| **1622** | *Radix Salviae* | PHA | P14679 | TYR |
| **1623** | *Radix Salviae* | PHA | O43242 | PSMD3 |
| **1624** | *Radix Salviae* | PHA | Q07075 | ENPEP |
| **1625** | *Radix Salviae* | PHA | P28072 | PSMB6 |
| **1626** | *Radix Salviae* | PHA | P28066 | PSMA5 |
| **1627** | *Radix Salviae* | PHA | P62979 | RPS27A |
| **1628** | *Radix Salviae* | PHA | O75223 | GGCT |
| **1629** | *Radix Salviae* | PHA | P28070 | PSMB4 |
| **1630** | *Radix Salviae* | PHA | P35998 | PSMC2 |
| **1631** | *Radix Salviae* | PHA | P61289 | PSME3 |
| **1632** | *Radix Salviae* | PHA | Q9UNI1 | CELA1 |
| **1633** | *Radix Salviae* | PHA | Q15008 | PSMD6 |
| **1634** | *Radix Salviae* | PHA | Q9NZ08 | ERAP1 |
| **1635** | *Radix Salviae* | PHA | Q9H9S3 | SEC61A2 |
| **1636** | *Radix Salviae* | PHA | P17980 | PSMC3 |
| **1637** | *Radix Salviae* | PHA | P30101 | PDIA3 |
| **1638** | *Radix Salviae* | PHA | P0CG47 | UBB |
| **1639** | *Radix Salviae* | PHA | Q99460 | PSMD1 |
| **1640** | *Radix Salviae* | PHA | Q13200 | PSMD2 |
| **1641** | *Radix Salviae* | PHA | P62195 | PSMC5 |
| **1642** | *Radix Salviae* | PHA | Q8TAA3 | PSMA8 |
| **1643** | *Radix Salviae* | PHA | P55786 | NPEPPS |
| **1644** | *Radix Salviae* | PHA | Q9UHI5 | SLC7A8 |
| **1645** | *Radix Salviae* | PHA | P27797 | CALR |
| **1646** | *Radix Salviae* | PHA | Q96KP4 | CNDP2 |
| **1647** | *Radix Salviae* | PHA | Q92530 | PSMF1 |
| **1648** | *Radix Salviae* | PHA | P60059 | SEC61G |
| **1649** | *Radix Salviae* | PHA | Q96RQ9 | IL4I1 |
| **1650** | *Radix Salviae* | PHA | P0CG48 | UBC |
| **1651** | *Radix Salviae* | PHA | Q7L5Y1 | ENOSF1 |
| **1652** | *Radix Salviae* | PHA | P17735 | TAT |
| **1653** | *Radix Salviae* | PHA | O00232 | PSMD12 |
| **1654** | *Radix Salviae* | PHA | P40306 | PSMB10 |
| **1655** | *Radix Salviae* | PHA | P17693 | HLA-G |
| **1656** | *Radix Salviae* | PHA | P28074 | PSMB5 |
| **1657** | *Radix Salviae* | PHA | P55036 | PSMD4 |
| **1658** | *Radix Salviae* | PHA | P25774 | CTSS |
| **1659** | *Radix Salviae* | PHA | P17174 | GOT1 |
| **1660** | *Radix Salviae* | PHA | O14818 | PSMA7 |
| **1661** | *Radix Salviae* | PHA | P49721 | PSMB2 |
| **1662** | *Radix Salviae* | PHA | P29144 | TPP2 |
| **1663** | *Radix Salviae* | PHA | P30499 | HLA-C |
| **1664** | *Radix Salviae* | PHA | P30443 | HLA-A |
| **1665** | *Radix Salviae* | PHA | P08195 | SLC3A2 |
| **1666** | *Radix Salviae* | PHA | P01185 | AVP |
| **1667** | *Radix Salviae* | PHA | P07101 | TH |
| **1668** | *Radix Salviae* | PHA | Q06323 | PSME1 |
| **1669** | *Radix Salviae* | PHA | Q12904 | AIMP1 |
| **1670** | *Radix Salviae* | PHA | Q14997 | PSME4 |
| **1671** | *Radix Salviae* | PHA | A5LHX3 | PSMB11 |
| **1672** | *Radix Salviae* | PHA | O00487 | PSMD14 |
| **1673** | *Radix Salviae* | PHA | P62987 | UBA52 |
| **1674** | *Radix Salviae* | PHA | Q9UNM6 | PSMD13 |
| **1675** | *Radix Salviae* | PHA | P01889 | HLA-B |
| **1676** | *Radix Salviae* | PHA | Q6P179 | ERAP2 |
| **1677** | *Radix Salviae* | PHA | P62333 | PSMC6 |
| **1678** | *Radix Salviae* | PHA | P15151 | PVR |
| **1679** | *Radix Salviae* | PHA | P25786 | PSMA1 |
| **1680** | *Radix Salviae* | PHA | O00233 | PSMD9 |
| **1681** | *Radix Salviae* | PHA | P61769 | B2M |
| **1682** | *Radix Salviae* | Physcion | O60656 | UGT1A9 |
| **1683** | *Radix Salviae* | poriferast-5-en-3beta-ol | P05362 | ICAM1 |
| **1684** | *Radix Salviae* | poriferast-5-en-3beta-ol | P42574 | CASP3 |
| **1685** | *Radix Salviae* | poriferast-5-en-3beta-ol | P55211 | CASP9 |
| **1686** | *Radix Salviae* | poriferast-5-en-3beta-ol | P09874 | PARP1 |
| **1687** | *Radix Salviae* | Prolinum | P25789 | PSMA4 |
| **1688** | *Radix Salviae* | Prolinum | P43686 | PSMC4 |
| **1689** | *Radix Salviae* | Prolinum | Q16401 | PSMD5 |
| **1690** | *Radix Salviae* | Prolinum | P48556 | PSMD8 |
| **1691** | *Radix Salviae* | Prolinum | P25788 | PSMA3 |
| **1692** | *Radix Salviae* | Prolinum | Q9UL46 | PSME2 |
| **1693** | *Radix Salviae* | Prolinum | O75832 | PSMD10 |
| **1694** | *Radix Salviae* | Prolinum | P51665 | PSMD7 |
| **1695** | *Radix Salviae* | Prolinum | Q13155 | AIMP2 |
| **1696** | *Radix Salviae* | Prolinum | P60468 | SEC61B |
| **1697** | *Radix Salviae* | Prolinum | P30838 | ALDH3A1 |
| **1698** | *Radix Salviae* | Prolinum | Q9UIQ6 | LNPEP |
| **1699** | *Radix Salviae* | Prolinum | P54136 | RARS |
| **1700** | *Radix Salviae* | Prolinum | P61619 | SEC61A1 |
| **1701** | *Radix Salviae* | Prolinum | O94788 | ALDH1A2 |
| **1702** | *Radix Salviae* | Prolinum | P48448 | ALDH3B2 |
| **1703** | *Radix Salviae* | Prolinum | Q3SY69 | ALDH1L2 |
| **1704** | *Radix Salviae* | Prolinum | Q99436 | PSMB7 |
| **1705** | *Radix Salviae* | Prolinum | P62191 | PSMC1 |
| **1706** | *Radix Salviae* | Prolinum | P60900 | PSMA6 |
| **1707** | *Radix Salviae* | Prolinum | O00231 | PSMD11 |
| **1708** | *Radix Salviae* | Prolinum | Q13867 | BLMH |
| **1709** | *Radix Salviae* | Prolinum | P05091 | ALDH2 |
| **1710** | *Radix Salviae* | Prolinum | P56192 | MARS |
| **1711** | *Radix Salviae* | Prolinum | P20618 | PSMB1 |
| **1712** | *Radix Salviae* | Prolinum | P14868 | DARS |
| **1713** | *Radix Salviae* | Prolinum | O43242 | PSMD3 |
| **1714** | *Radix Salviae* | Prolinum | Q9H2A2 | ALDH8A1 |
| **1715** | *Radix Salviae* | Prolinum | Q9H2J7 | SLC6A15 |
| **1716** | *Radix Salviae* | Prolinum | P28072 | PSMB6 |
| **1717** | *Radix Salviae* | Prolinum | P28066 | PSMA5 |
| **1718** | *Radix Salviae* | Prolinum | P62979 | RPS27A |
| **1719** | *Radix Salviae* | Prolinum | O75223 | GGCT |
| **1720** | *Radix Salviae* | Prolinum | P28070 | PSMB4 |
| **1721** | *Radix Salviae* | Prolinum | P30038 | ALDH4A1 |
| **1722** | *Radix Salviae* | Prolinum | P35998 | PSMC2 |
| **1723** | *Radix Salviae* | Prolinum | Q8IZ83 | ALDH16A1 |
| **1724** | *Radix Salviae* | Prolinum | P61289 | PSME3 |
| **1725** | *Radix Salviae* | Prolinum | Q15008 | PSMD6 |
| **1726** | *Radix Salviae* | Prolinum | Q9NZ08 | ERAP1 |
| **1727** | *Radix Salviae* | Prolinum | P00352 | ALDH1A1 |
| **1728** | *Radix Salviae* | Prolinum | Q9H9S3 | SEC61A2 |
| **1729** | *Radix Salviae* | Prolinum | P17980 | PSMC3 |
| **1730** | *Radix Salviae* | Prolinum | P30101 | PDIA3 |
| **1731** | *Radix Salviae* | Prolinum | P0CG47 | UBB |
| **1732** | *Radix Salviae* | Prolinum | P47897 | QARS |
| **1733** | *Radix Salviae* | Prolinum | Q99460 | PSMD1 |
| **1734** | *Radix Salviae* | Prolinum | Q13200 | PSMD2 |
| **1735** | *Radix Salviae* | Prolinum | P62195 | PSMC5 |
| **1736** | *Radix Salviae* | Prolinum | Q8TAA3 | PSMA8 |
| **1737** | *Radix Salviae* | Prolinum | P51649 | ALDH5A1 |
| **1738** | *Radix Salviae* | Prolinum | P55786 | NPEPPS |
| **1739** | *Radix Salviae* | Prolinum | P27797 | CALR |
| **1740** | *Radix Salviae* | Prolinum | Q15046 | KARS |
| **1741** | *Radix Salviae* | Prolinum | Q96KP4 | CNDP2 |
| **1742** | *Radix Salviae* | Prolinum | Q92530 | PSMF1 |
| **1743** | *Radix Salviae* | Prolinum | P47895 | ALDH1A3 |
| **1744** | *Radix Salviae* | Prolinum | P60059 | SEC61G |
| **1745** | *Radix Salviae* | Prolinum | P0CG48 | UBC |
| **1746** | *Radix Salviae* | Prolinum | P51648 | ALDH3A2 |
| **1747** | *Radix Salviae* | Prolinum | Q7L5Y1 | ENOSF1 |
| **1748** | *Radix Salviae* | Prolinum | P49189 | ALDH9A1 |
| **1749** | *Radix Salviae* | Prolinum | O00232 | PSMD12 |
| **1750** | *Radix Salviae* | Prolinum | P40306 | PSMB10 |
| **1751** | *Radix Salviae* | Prolinum | P17693 | HLA-G |
| **1752** | *Radix Salviae* | Prolinum | P28074 | PSMB5 |
| **1753** | *Radix Salviae* | Prolinum | P07814 | EPRS |
| **1754** | *Radix Salviae* | Prolinum | P55036 | PSMD4 |
| **1755** | *Radix Salviae* | Prolinum | P25774 | CTSS |
| **1756** | *Radix Salviae* | Prolinum | O14818 | PSMA7 |
| **1757** | *Radix Salviae* | Prolinum | P49721 | PSMB2 |
| **1758** | *Radix Salviae* | Prolinum | P41252 | IARS |
| **1759** | *Radix Salviae* | Prolinum | P29144 | TPP2 |
| **1760** | *Radix Salviae* | Prolinum | P30499 | HLA-C |
| **1761** | *Radix Salviae* | Prolinum | P30443 | HLA-A |
| **1762** | *Radix Salviae* | Prolinum | P30837 | ALDH1B1 |
| **1763** | *Radix Salviae* | Prolinum | O43324 | EEF1E1 |
| **1764** | *Radix Salviae* | Prolinum | Q06323 | PSME1 |
| **1765** | *Radix Salviae* | Prolinum | O75891 | ALDH1L1 |
| **1766** | *Radix Salviae* | Prolinum | Q9P2J5 | LARS |
| **1767** | *Radix Salviae* | Prolinum | Q12904 | AIMP1 |
| **1768** | *Radix Salviae* | Prolinum | Q14997 | PSME4 |
| **1769** | *Radix Salviae* | Prolinum | A5LHX3 | PSMB11 |
| **1770** | *Radix Salviae* | Prolinum | O00487 | PSMD14 |
| **1771** | *Radix Salviae* | Prolinum | P49419 | ALDH7A1 |
| **1772** | *Radix Salviae* | Prolinum | Q5TF58 | IFFO2 |
| **1773** | *Radix Salviae* | Prolinum | P62987 | UBA52 |
| **1774** | *Radix Salviae* | Prolinum | Q9UNM6 | PSMD13 |
| **1775** | *Radix Salviae* | Prolinum | P01889 | HLA-B |
| **1776** | *Radix Salviae* | Prolinum | Q6P179 | ERAP2 |
| **1777** | *Radix Salviae* | Prolinum | P62333 | PSMC6 |
| **1778** | *Radix Salviae* | Prolinum | P15151 | PVR |
| **1779** | *Radix Salviae* | Prolinum | P25786 | PSMA1 |
| **1780** | *Radix Salviae* | Prolinum | O00233 | PSMD9 |
| **1781** | *Radix Salviae* | Prolinum | P61769 | B2M |
| **1782** | *Radix Salviae* | protocatechuic acid | P05164 | MPO |
| **1783** | *Radix Salviae* | rutin | Q04206 | RELA |
| **1784** | *Radix Salviae* | rutin | P01375 | TNF |
| **1785** | *Radix Salviae* | rutin | P04035 | HMGCR |
| **1786** | *Radix Salviae* | rutin | P16435 | POR |
| **1787** | *Radix Salviae* | rutin | P42574 | CASP3 |
| **1788** | *Radix Salviae* | rutin | P09917 | ALOX5 |
| **1789** | *Radix Salviae* | rutin | P05231 | IL6 |
| **1790** | *Radix Salviae* | rutin | P21730 | C5AR1 |
| **1791** | *Radix Salviae* | rutin | P21731 | TBXA2R |
| **1792** | *Radix Salviae* | rutin | P04040 | CAT |
| **1793** | *Radix Salviae* | rutin | P00441 | SOD1 |
| **1794** | *Radix Salviae* | rutin | P35228 | NOS2 |
| **1795** | *Radix Salviae* | rutin | P09211 | GSTP1 |
| **1796** | *Radix Salviae* | rutin | P01584 | IL1B |
| **1797** | *Radix Salviae* | rutin | P10145 | CXCL8 |
| **1798** | *Radix Salviae* | succinic acid | P21917 | DRD4 |
| **1799** | *Radix Salviae* | succinic acid | P09622 | DLD |
| **1800** | *Radix Salviae* | succinic acid | P05546 | SERPIND1 |
| **1801** | *Radix Salviae* | succinic acid | P46663 | BDKRB1 |
| **1802** | *Radix Salviae* | succinic acid | Q16698 | DECR1 |
| **1803** | *Radix Salviae* | succinic acid | P00750 | PLAT |
| **1804** | *Radix Salviae* | succinic acid | P00390 | GSR |
| **1805** | *Radix Salviae* | succinic acid | P01137 | TGFB1 |
| **1806** | *Radix Salviae* | succinic acid | P05121 | SERPINE1 |
| **1807** | *Radix Salviae* | succinic acid | P01031 | C5 |
| **1808** | *Radix Salviae* | succinic acid | P30838 | ALDH3A1 |
| **1809** | *Radix Salviae* | succinic acid | P04040 | CAT |
| **1810** | *Radix Salviae* | succinic acid | P00505 | GOT2 |
| **1811** | *Radix Salviae* | succinic acid | P01024 | C3 |
| **1812** | *Radix Salviae* | succinic acid | Q99705 | MCHR1 |
| **1813** | *Radix Salviae* | succinic acid | O94788 | ALDH1A2 |
| **1814** | *Radix Salviae* | succinic acid | P23946 | CMA1 |
| **1815** | *Radix Salviae* | succinic acid | P53396 | ACLY |
| **1816** | *Radix Salviae* | succinic acid | P48448 | ALDH3B2 |
| **1817** | *Radix Salviae* | succinic acid | Q9GZQ4 | NMUR2 |
| **1818** | *Radix Salviae* | succinic acid | P49286 | MTNR1B |
| **1819** | *Radix Salviae* | succinic acid | P04083 | ANXA1 |
| **1820** | *Radix Salviae* | succinic acid | Q3SY69 | ALDH1L2 |
| **1821** | *Radix Salviae* | succinic acid | P05091 | ALDH2 |
| **1822** | *Radix Salviae* | succinic acid | P48645 | NMU |
| **1823** | *Radix Salviae* | succinic acid | P01189 | POMC |
| **1824** | *Radix Salviae* | succinic acid | P31040 | SDHA |
| **1825** | *Radix Salviae* | succinic acid | P01042 | KNG1 |
| **1826** | *Radix Salviae* | succinic acid | Q9H2A2 | ALDH8A1 |
| **1827** | *Radix Salviae* | succinic acid | Q13304 | GPR17 |
| **1828** | *Radix Salviae* | succinic acid | P08913 | ADRA2A |
| **1829** | *Radix Salviae* | succinic acid | Q969V1 | MCHR2 |
| **1830** | *Radix Salviae* | succinic acid | O60235 | TMPRSS11D |
| **1831** | *Radix Salviae* | succinic acid | P05067 | APP |
| **1832** | *Radix Salviae* | succinic acid | P30038 | ALDH4A1 |
| **1833** | *Radix Salviae* | succinic acid | Q8IZ83 | ALDH16A1 |
| **1834** | *Radix Salviae* | succinic acid | Q9UNI1 | CELA1 |
| **1835** | *Radix Salviae* | succinic acid | P02768 | ALB |
| **1836** | *Radix Salviae* | succinic acid | O76081 | RGS20 |
| **1837** | *Radix Salviae* | succinic acid | Q99527 | GPER1 |
| **1838** | *Radix Salviae* | succinic acid | P00352 | ALDH1A1 |
| **1839** | *Radix Salviae* | succinic acid | P48039 | MTNR1A |
| **1840** | *Radix Salviae* | succinic acid | P02778 | CXCL10 |
| **1841** | *Radix Salviae* | succinic acid | Q9HB89 | NMUR1 |
| **1842** | *Radix Salviae* | succinic acid | P10145 | CXCL8 |
| **1843** | *Radix Salviae* | succinic acid | P99999 | CYCS |
| **1844** | *Radix Salviae* | succinic acid | P07477 | PRSS1 |
| **1845** | *Radix Salviae* | succinic acid | P21399 | ACO1 |
| **1846** | *Radix Salviae* | succinic acid | P51649 | ALDH5A1 |
| **1847** | *Radix Salviae* | succinic acid | P34896 | SHMT1 |
| **1848** | *Radix Salviae* | succinic acid | P07237 | P4HB |
| **1849** | *Radix Salviae* | succinic acid | Q9H1C0 | LPAR5 |
| **1850** | *Radix Salviae* | succinic acid | P20382 | PMCH |
| **1851** | *Radix Salviae* | succinic acid | P47895 | ALDH1A3 |
| **1852** | *Radix Salviae* | succinic acid | P49795 | RGS19 |
| **1853** | *Radix Salviae* | succinic acid | P59768 | GNG2 |
| **1854** | *Radix Salviae* | succinic acid | Q16665 | HIF1A |
| **1855** | *Radix Salviae* | succinic acid | P25090 | FPR2 |
| **1856** | *Radix Salviae* | succinic acid | P28290 | CS |
| **1857** | *Radix Salviae* | succinic acid | P63096 | GNAI1 |
| **1858** | *Radix Salviae* | succinic acid | P51648 | ALDH3A2 |
| **1859** | *Radix Salviae* | succinic acid | P49189 | ALDH9A1 |
| **1860** | *Radix Salviae* | succinic acid | P02751 | FN1 |
| **1861** | *Radix Salviae* | succinic acid | P21730 | C5AR1 |
| **1862** | *Radix Salviae* | succinic acid | P0DJI8 | SAA1 |
| **1863** | *Radix Salviae* | succinic acid | Q42521 | GAD1 |
| **1864** | *Radix Salviae* | succinic acid | Q92633 | LPAR1 |
| **1865** | *Radix Salviae* | succinic acid | P35030 | PRSS3 |
| **1866** | *Radix Salviae* | succinic acid | P25929 | NPY1R |
| **1867** | *Radix Salviae* | succinic acid | Q8N5D0 | WDTC1 |
| **1868** | *Radix Salviae* | succinic acid | P49802 | RGS7 |
| **1869** | *Radix Salviae* | succinic acid | P01019 | AGT |
| **1870** | *Radix Salviae* | succinic acid | Q08116 | RGS1 |
| **1871** | *Radix Salviae* | succinic acid | Q9NS28 | RGS18 |
| **1872** | *Radix Salviae* | succinic acid | O43665 | RGS10 |
| **1873** | *Radix Salviae* | succinic acid | P08754 | GNAI3 |
| **1874** | *Radix Salviae* | succinic acid | P17174 | GOT1 |
| **1875** | *Radix Salviae* | succinic acid | Q9UBY5 | LPAR3 |
| **1876** | *Radix Salviae* | succinic acid | P60484 | PTEN |
| **1877** | *Radix Salviae* | succinic acid | P00749 | PLAU |
| **1878** | *Radix Salviae* | succinic acid | P21912 | SDHB |
| **1879** | *Radix Salviae* | succinic acid | Q5H8A3 | NMS |
| **1880** | *Radix Salviae* | succinic acid | P30837 | ALDH1B1 |
| **1881** | *Radix Salviae* | succinic acid | P68106 | FKBP1B |
| **1882** | *Radix Salviae* | succinic acid | Q96BT7 | ALKBH8 |
| **1883** | *Radix Salviae* | succinic acid | O75891 | ALDH1L1 |
| **1884** | *Radix Salviae* | succinic acid | P09341 | CXCL1 |
| **1885** | *Radix Salviae* | succinic acid | Q9HBW0 | LPAR2 |
| **1886** | *Radix Salviae* | succinic acid | P18825 | ADRA2C |
| **1887** | *Radix Salviae* | succinic acid | O43566 | RGS14 |
| **1888** | *Radix Salviae* | succinic acid | P61073 | CXCR4 |
| **1889** | *Radix Salviae* | succinic acid | P49419 | ALDH7A1 |
| **1890** | *Radix Salviae* | succinic acid | Q5TF58 | IFFO2 |
| **1891** | *Radix Salviae* | succinic acid | P35372 | OPRM1 |
| **1892** | *Radix Salviae* | succinic acid | P49798 | RGS4 |
| **1893** | *Radix Salviae* | succinic acid | P00739 | HPR |
| **1894** | *Radix Salviae* | succinic acid | P49758 | RGS6 |
| **1895** | *Radix Salviae* | succinic acid | P40313 | CTRL |
| **1896** | *Radix Salviae* | tanshinone i | P24385 | CCND1 |
| **1897** | *Radix Salviae* | tanshinone i | O60235 | TMPRSS11D |
| **1898** | *Radix Salviae* | tanshinone i | P29474 | NOS3 |
| **1899** | *Radix Salviae* | tanshinone i | P42574 | CASP3 |
| **1900** | *Radix Salviae* | tanshinone i | O75469 | NR1I2 |
| **1901** | *Radix Salviae* | tanshinone i | P05177 | CYP1A2 |
| **1902** | *Radix Salviae* | tanshinone i | P33261 | CYP2C19 |
| **1903** | *Radix Salviae* | tanshinone i | P05362 | ICAM1 |
| **1904** | *Radix Salviae* | tanshinone iia | P08253 | MMP2 |
| **1905** | *Radix Salviae* | tanshinone iia | P24385 | CCND1 |
| **1906** | *Radix Salviae* | tanshinone iia | P05305 | EDN1 |
| **1907** | *Radix Salviae* | tanshinone iia | O75469 | NR1I2 |
| **1908** | *Radix Salviae* | tanshinone iia | P08684 | CYP3A4 |
| **1909** | *Radix Salviae* | tanshinone iia | P04637 | TP53 |
| **1910** | *Radix Salviae* | tanshinone iia | P38936 | CDKN1A |
| **1911** | *Radix Salviae* | tanshinone iia | P14780 | MMP9 |
| **1912** | *Radix Salviae* | tanshinone iia | P25963 | NFKBIA |
| **1913** | *Radix Salviae* | tanshinone iia | Q04206 | RELA |
| **1914** | *Radix Salviae* | tanshinone iia | P05412 | JUN |
| **1915** | *Radix Salviae* | tanshinone iia | P01100 | FOS |
| **1916** | *Radix Salviae* | tanshinone iia | P04798 | CYP1A1 |
| **1917** | *Radix Salviae* | tanshinone iia | P10415 | BCL2 |
| **1918** | *Radix Salviae* | tanshinone iia | P01106 | MYC |
| **1919** | *Radix Salviae* | tanshinone iia | P12931 | SRC |
| **1920** | *Radix Salviae* | tanshinone iia | O60235 | TMPRSS11D |
| **1921** | *Radix Salviae* | tanshinone iia | P29474 | NOS3 |
| **1922** | *Radix Salviae* | tanshinone iia | P42574 | CASP3 |
| **1923** | *Radix Salviae* | tanshinone iia | P05177 | CYP1A2 |
| **1924** | *Radix Salviae* | tanshinone iia | P33261 | CYP2C19 |
| **1925** | *Radix Salviae* | Threonin | P25789 | PSMA4 |
| **1926** | *Radix Salviae* | Threonin | P43686 | PSMC4 |
| **1927** | *Radix Salviae* | Threonin | Q16401 | PSMD5 |
| **1928** | *Radix Salviae* | Threonin | P48556 | PSMD8 |
| **1929** | *Radix Salviae* | Threonin | P25788 | PSMA3 |
| **1930** | *Radix Salviae* | Threonin | Q9UL46 | PSME2 |
| **1931** | *Radix Salviae* | Threonin | O75832 | PSMD10 |
| **1932** | *Radix Salviae* | Threonin | P51665 | PSMD7 |
| **1933** | *Radix Salviae* | Threonin | P60468 | SEC61B |
| **1934** | *Radix Salviae* | Threonin | Q9UIQ6 | LNPEP |
| **1935** | *Radix Salviae* | Threonin | P61619 | SEC61A1 |
| **1936** | *Radix Salviae* | Threonin | Q99436 | PSMB7 |
| **1937** | *Radix Salviae* | Threonin | P62191 | PSMC1 |
| **1938** | *Radix Salviae* | Threonin | P60900 | PSMA6 |
| **1939** | *Radix Salviae* | Threonin | O00231 | PSMD11 |
| **1940** | *Radix Salviae* | Threonin | Q13867 | BLMH |
| **1941** | *Radix Salviae* | Threonin | P20618 | PSMB1 |
| **1942** | *Radix Salviae* | Threonin | O43242 | PSMD3 |
| **1943** | *Radix Salviae* | Threonin | Q9H2J7 | SLC6A15 |
| **1944** | *Radix Salviae* | Threonin | P28072 | PSMB6 |
| **1945** | *Radix Salviae* | Threonin | P28066 | PSMA5 |
| **1946** | *Radix Salviae* | Threonin | P62979 | RPS27A |
| **1947** | *Radix Salviae* | Threonin | O75223 | GGCT |
| **1948** | *Radix Salviae* | Threonin | P28070 | PSMB4 |
| **1949** | *Radix Salviae* | Threonin | P35998 | PSMC2 |
| **1950** | *Radix Salviae* | Threonin | P61289 | PSME3 |
| **1951** | *Radix Salviae* | Threonin | Q15008 | PSMD6 |
| **1952** | *Radix Salviae* | Threonin | Q9NZ08 | ERAP1 |
| **1953** | *Radix Salviae* | Threonin | Q9H2F3 | HSD3B7 |
| **1954** | *Radix Salviae* | Threonin | Q9H9S3 | SEC61A2 |
| **1955** | *Radix Salviae* | Threonin | P17980 | PSMC3 |
| **1956** | *Radix Salviae* | Threonin | P30101 | PDIA3 |
| **1957** | *Radix Salviae* | Threonin | P0CG47 | UBB |
| **1958** | *Radix Salviae* | Threonin | Q99460 | PSMD1 |
| **1959** | *Radix Salviae* | Threonin | Q13200 | PSMD2 |
| **1960** | *Radix Salviae* | Threonin | P62195 | PSMC5 |
| **1961** | *Radix Salviae* | Threonin | Q8TAA3 | PSMA8 |
| **1962** | *Radix Salviae* | Threonin | P55786 | NPEPPS |
| **1963** | *Radix Salviae* | Threonin | Q9UHI5 | SLC7A8 |
| **1964** | *Radix Salviae* | Threonin | P27797 | CALR |
| **1965** | *Radix Salviae* | Threonin | Q96KP4 | CNDP2 |
| **1966** | *Radix Salviae* | Threonin | Q92530 | PSMF1 |
| **1967** | *Radix Salviae* | Threonin | P60059 | SEC61G |
| **1968** | *Radix Salviae* | Threonin | P0CG48 | UBC |
| **1969** | *Radix Salviae* | Threonin | O00232 | PSMD12 |
| **1970** | *Radix Salviae* | Threonin | P40306 | PSMB10 |
| **1971** | *Radix Salviae* | Threonin | P17693 | HLA-G |
| **1972** | *Radix Salviae* | Threonin | P28074 | PSMB5 |
| **1973** | *Radix Salviae* | Threonin | P55036 | PSMD4 |
| **1974** | *Radix Salviae* | Threonin | P25774 | CTSS |
| **1975** | *Radix Salviae* | Threonin | P14060 | HSD3B1 |
| **1976** | *Radix Salviae* | Threonin | P26439 | HSD3B2 |
| **1977** | *Radix Salviae* | Threonin | O14818 | PSMA7 |
| **1978** | *Radix Salviae* | Threonin | P49721 | PSMB2 |
| **1979** | *Radix Salviae* | Threonin | P29144 | TPP2 |
| **1980** | *Radix Salviae* | Threonin | P30499 | HLA-C |
| **1981** | *Radix Salviae* | Threonin | P30443 | HLA-A |
| **1982** | *Radix Salviae* | Threonin | P08195 | SLC3A2 |
| **1983** | *Radix Salviae* | Threonin | Q06323 | PSME1 |
| **1984** | *Radix Salviae* | Threonin | Q14997 | PSME4 |
| **1985** | *Radix Salviae* | Threonin | A5LHX3 | PSMB11 |
| **1986** | *Radix Salviae* | Threonin | O00487 | PSMD14 |
| **1987** | *Radix Salviae* | Threonin | P62987 | UBA52 |
| **1988** | *Radix Salviae* | Threonin | Q9UNM6 | PSMD13 |
| **1989** | *Radix Salviae* | Threonin | Q13630 | TSTA3 |
| **1990** | *Radix Salviae* | Threonin | P01889 | HLA-B |
| **1991** | *Radix Salviae* | Threonin | Q6P179 | ERAP2 |
| **1992** | *Radix Salviae* | Threonin | P62333 | PSMC6 |
| **1993** | *Radix Salviae* | Threonin | P15151 | PVR |
| **1994** | *Radix Salviae* | Threonin | P25786 | PSMA1 |
| **1995** | *Radix Salviae* | Threonin | O00233 | PSMD9 |
| **1996** | *Radix Salviae* | Threonin | P61769 | B2M |
| **1997** | *Radix Salviae* | ursolic acid | P31749 | AKT1 |
| **1998** | *Radix Salviae* | ursolic acid | P42574 | CASP3 |
| **1999** | *Radix Salviae* | ursolic acid | P10415 | BCL2 |
| **2000** | *Radix Salviae* | ursolic acid | P35222 | CTNNB1 |
| **2001** | *Radix Salviae* | ursolic acid | Q14790 | CASP8 |
| **2002** | *Radix Salviae* | ursolic acid | P11387 | TOP1 |
| **2003** | *Radix Salviae* | ursolic acid | P42345 | MTOR |
| **2004** | *Radix Salviae* | ursolic acid | P23219 | PTGS1 |
| **2005** | *Radix Salviae* | ursolic acid | P09874 | PARP1 |
| **2006** | *Radix Salviae* | ursolic acid | P33261 | CYP2C19 |
| **2007** | *Radix Salviae* | ursolic acid | P11388 | TOP2A |
| **2008** | *Radix Salviae* | VIV | P05546 | SERPIND1 |
| **2009** | *Radix Salviae* | VIV | P28482 | MAPK1 |
| **2010** | *Radix Salviae* | VIV | P30519 | HMOX2 |
| **2011** | *Radix Salviae* | VIV | Q16698 | DECR1 |
| **2012** | *Radix Salviae* | VIV | P00390 | GSR |
| **2013** | *Radix Salviae* | VIV | P78329 | CYP4F2 |
| **2014** | *Radix Salviae* | VIV | P01137 | TGFB1 |
| **2015** | *Radix Salviae* | VIV | P05121 | SERPINE1 |
| **2016** | *Radix Salviae* | VIV | P04114 | APOB |
| **2017** | *Radix Salviae* | VIV | P04040 | CAT |
| **2018** | *Radix Salviae* | VIV | P02649 | APOE |
| **2019** | *Radix Salviae* | VIV | P05231 | IL6 |
| **2020** | *Radix Salviae* | VIV | Q8WTV0 | SCARB1 |
| **2021** | *Radix Salviae* | VIV | P27361 | MAPK3 |
| **2022** | *Radix Salviae* | VIV | P06276 | BCHE |
| **2023** | *Radix Salviae* | VIV | P04637 | TP53 |
| **2024** | *Radix Salviae* | VIV | P00441 | SOD1 |
| **2025** | *Radix Salviae* | VIV | P31749 | AKT1 |
| **2026** | *Radix Salviae* | VIV | O60235 | TMPRSS11D |
| **2027** | *Radix Salviae* | VIV | P37231 | PPARG |
| **2028** | *Radix Salviae* | VIV | O60760 | HPGDS |
| **2029** | *Radix Salviae* | VIV | P29474 | NOS3 |
| **2030** | *Radix Salviae* | VIV | P21399 | ACO1 |
| **2031** | *Radix Salviae* | VIV | P42574 | CASP3 |
| **2032** | *Radix Salviae* | VIV | P15559 | NQO1 |
| **2033** | *Radix Salviae* | VIV | Q15848 | ADIPOQ |
| **2034** | *Radix Salviae* | VIV | P55211 | CASP9 |
| **2035** | *Radix Salviae* | VIV | O75469 | NR1I2 |
| **2036** | *Radix Salviae* | VIV | P45983 | MAPK8 |
| **2037** | *Radix Salviae* | VIV | P01019 | AGT |
| **2038** | *Radix Salviae* | VIV | P05412 | JUN |
| **2039** | *Radix Salviae* | VIV | P09917 | ALOX5 |
| **2040** | *Radix Salviae* | VIV | P01106 | MYC |
| **2041** | *Radix Salviae* | VIV | P09211 | GSTP1 |
| **2042** | *Radix Salviae* | VIV | P21731 | TBXA2R |
| **2043** | *Radix Salviae* | VIV | P01375 | TNF |
| **2044** | *Radix Salviae* | VIV | P07203 | GPX1 |
| **2045** | *Radix Salviae* | VIV | P17252 | PRKCA |
| **2046** | *Radix Salviae* | VIV | P00739 | HPR |
| **2047** | *Folium Nelumbinis* | 3,4,5-trihydroxybenzoic acid | P08253 | MMP2 |
| **2048** | *Folium Nelumbinis* | 3,4,5-trihydroxybenzoic acid | P05121 | SERPINE1 |
| **2049** | *Folium Nelumbinis* | 3,4,5-trihydroxybenzoic acid | P05164 | MPO |
| **2050** | *Folium Nelumbinis* | 3,4,5-trihydroxybenzoic acid | P04114 | APOB |
| **2051** | *Folium Nelumbinis* | 3,4,5-trihydroxybenzoic acid | P04040 | CAT |
| **2052** | *Folium Nelumbinis* | 3,4,5-trihydroxybenzoic acid | P14679 | TYR |
| **2053** | *Folium Nelumbinis* | 3,4,5-trihydroxybenzoic acid | P08183 | ABCB1 |
| **2054** | *Folium Nelumbinis* | 3,4,5-trihydroxybenzoic acid | P31749 | AKT1 |
| **2055** | *Folium Nelumbinis* | 3,4,5-trihydroxybenzoic acid | Q13315 | ATM |
| **2056** | *Folium Nelumbinis* | 3,4,5-trihydroxybenzoic acid | P42574 | CASP3 |
| **2057** | *Folium Nelumbinis* | 3,4,5-trihydroxybenzoic acid | O75795 | UGT2B17 |
| **2058** | *Folium Nelumbinis* | 3,4,5-trihydroxybenzoic acid | P50225 | SULT1A1 |
| **2059** | *Folium Nelumbinis* | 3,4,5-trihydroxybenzoic acid | P55210 | CASP7 |
| **2060** | *Folium Nelumbinis* | 3,4,5-trihydroxybenzoic acid | O15111 | CHUK |
| **2061** | *Folium Nelumbinis* | 3,4,5-trihydroxybenzoic acid | P05412 | JUN |
| **2062** | *Folium Nelumbinis* | 3,4,5-trihydroxybenzoic acid | P14780 | MMP9 |
| **2063** | *Folium Nelumbinis* | 3,4,5-trihydroxybenzoic acid | P17252 | PRKCA |
| **2064** | *Folium Nelumbinis* | anonaine | P07101 | TH |
| **2065** | *Folium Nelumbinis* | CAM | P78329 | CYP4F2 |
| **2066** | *Folium Nelumbinis* | CAM | P05181 | CYP2E1 |
| **2067** | *Folium Nelumbinis* | CAM | P11712 | CYP2C9 |
| **2068** | *Folium Nelumbinis* | CAM | P08684 | CYP3A4 |
| **2069** | *Folium Nelumbinis* | CAM | P05177 | CYP1A2 |
| **2070** | *Folium Nelumbinis* | CAM | P10635 | CYP2D6 |
| **2071** | *Folium Nelumbinis* | CAM | P33261 | CYP2C19 |
| **2072** | *Folium Nelumbinis* | CAM | P04798 | CYP1A1 |
| **2073** | *Folium Nelumbinis* | catechol | P05164 | MPO |
| **2074** | *Folium Nelumbinis* | catechol | P04040 | CAT |
| **2075** | *Folium Nelumbinis* | catechol | P14679 | TYR |
| **2076** | *Folium Nelumbinis* | catechol | Q01959 | SLC6A3 |
| **2077** | *Folium Nelumbinis* | catechol | P42574 | CASP3 |
| **2078** | *Folium Nelumbinis* | catechol | P15559 | NQO1 |
| **2079** | *Folium Nelumbinis* | catechol | P50225 | SULT1A1 |
| **2080** | *Folium Nelumbinis* | catechol | P55211 | CASP9 |
| **2081** | *Folium Nelumbinis* | catechol | Q14790 | CASP8 |
| **2082** | *Folium Nelumbinis* | catechol | P23219 | PTGS1 |
| **2083** | *Folium Nelumbinis* | catechol | P55210 | CASP7 |
| **2084** | *Folium Nelumbinis* | catechol | P04798 | CYP1A1 |
| **2085** | *Folium Nelumbinis* | catechol | P07101 | TH |
| **2086** | *Folium Nelumbinis* | catechol | P09211 | GSTP1 |
| **2087** | *Folium Nelumbinis* | catechol | P01275 | GCG |
| **2088** | *Folium Nelumbinis* | citric acid | Q16698 | DECR1 |
| **2089** | *Folium Nelumbinis* | citric acid | P01137 | TGFB1 |
| **2090** | *Folium Nelumbinis* | citric acid | P30838 | ALDH3A1 |
| **2091** | *Folium Nelumbinis* | citric acid | O94788 | ALDH1A2 |
| **2092** | *Folium Nelumbinis* | citric acid | P23946 | CMA1 |
| **2093** | *Folium Nelumbinis* | citric acid | P53396 | ACLY |
| **2094** | *Folium Nelumbinis* | citric acid | P48448 | ALDH3B2 |
| **2095** | *Folium Nelumbinis* | citric acid | P01116 | KRAS |
| **2096** | *Folium Nelumbinis* | citric acid | P21589 | NT5E |
| **2097** | *Folium Nelumbinis* | citric acid | Q3SY69 | ALDH1L2 |
| **2098** | *Folium Nelumbinis* | citric acid | P05091 | ALDH2 |
| **2099** | *Folium Nelumbinis* | citric acid | P56192 | MARS |
| **2100** | *Folium Nelumbinis* | citric acid | P31040 | SDHA |
| **2101** | *Folium Nelumbinis* | citric acid | Q9H2A2 | ALDH8A1 |
| **2102** | *Folium Nelumbinis* | citric acid | Q16548 | BCL2A1 |
| **2103** | *Folium Nelumbinis* | citric acid | P04637 | TP53 |
| **2104** | *Folium Nelumbinis* | citric acid | P62979 | RPS27A |
| **2105** | *Folium Nelumbinis* | citric acid | P15121 | AKR1B1 |
| **2106** | *Folium Nelumbinis* | citric acid | P04035 | HMGCR |
| **2107** | *Folium Nelumbinis* | citric acid | P30038 | ALDH4A1 |
| **2108** | *Folium Nelumbinis* | citric acid | Q8IZ83 | ALDH16A1 |
| **2109** | *Folium Nelumbinis* | citric acid | Q9UNI1 | CELA1 |
| **2110** | *Folium Nelumbinis* | citric acid | P02768 | ALB |
| **2111** | *Folium Nelumbinis* | citric acid | O76081 | RGS20 |
| **2112** | *Folium Nelumbinis* | citric acid | P00352 | ALDH1A1 |
| **2113** | *Folium Nelumbinis* | citric acid | P30101 | PDIA3 |
| **2114** | *Folium Nelumbinis* | citric acid | Q07817 | BCL2L1 |
| **2115** | *Folium Nelumbinis* | citric acid | Q8N4T8 | CBR4 |
| **2116** | *Folium Nelumbinis* | citric acid | P15104 | GLUL |
| **2117** | *Folium Nelumbinis* | citric acid | P07477 | PRSS1 |
| **2118** | *Folium Nelumbinis* | citric acid | Q9BZR8 | BCL2L14 |
| **2119** | *Folium Nelumbinis* | citric acid | P21399 | ACO1 |
| **2120** | *Folium Nelumbinis* | citric acid | Q9UMX3 | BOK |
| **2121** | *Folium Nelumbinis* | citric acid | P51649 | ALDH5A1 |
| **2122** | *Folium Nelumbinis* | citric acid | P34896 | SHMT1 |
| **2123** | *Folium Nelumbinis* | citric acid | O15382 | BCAT2 |
| **2124** | *Folium Nelumbinis* | citric acid | P49841 | GSK3B |
| **2125** | *Folium Nelumbinis* | citric acid | P07237 | P4HB |
| **2126** | *Folium Nelumbinis* | citric acid | P30536 | TSPO |
| **2127** | *Folium Nelumbinis* | citric acid | P10415 | BCL2 |
| **2128** | *Folium Nelumbinis* | citric acid | P47895 | ALDH1A3 |
| **2129** | *Folium Nelumbinis* | citric acid | P49795 | RGS19 |
| **2130** | *Folium Nelumbinis* | citric acid | P28290 | CS |
| **2131** | *Folium Nelumbinis* | citric acid | Q96RQ9 | IL4I1 |
| **2132** | *Folium Nelumbinis* | citric acid | P63096 | GNAI1 |
| **2133** | *Folium Nelumbinis* | citric acid | P51648 | ALDH3A2 |
| **2134** | *Folium Nelumbinis* | citric acid | Q7L5Y1 | ENOSF1 |
| **2135** | *Folium Nelumbinis* | citric acid | P49189 | ALDH9A1 |
| **2136** | *Folium Nelumbinis* | citric acid | P12931 | SRC |
| **2137** | *Folium Nelumbinis* | citric acid | P35030 | PRSS3 |
| **2138** | *Folium Nelumbinis* | citric acid | P49802 | RGS7 |
| **2139** | *Folium Nelumbinis* | citric acid | Q08116 | RGS1 |
| **2140** | *Folium Nelumbinis* | citric acid | Q9NS28 | RGS18 |
| **2141** | *Folium Nelumbinis* | citric acid | Q07820 | MCL1 |
| **2142** | *Folium Nelumbinis* | citric acid | O43665 | RGS10 |
| **2143** | *Folium Nelumbinis* | citric acid | P55210 | CASP7 |
| **2144** | *Folium Nelumbinis* | citric acid | O43175 | PHGDH |
| **2145** | *Folium Nelumbinis* | citric acid | P08754 | GNAI3 |
| **2146** | *Folium Nelumbinis* | citric acid | P60484 | PTEN |
| **2147** | *Folium Nelumbinis* | citric acid | P00749 | PLAU |
| **2148** | *Folium Nelumbinis* | citric acid | P21912 | SDHB |
| **2149** | *Folium Nelumbinis* | citric acid | P29144 | TPP2 |
| **2150** | *Folium Nelumbinis* | citric acid | P30837 | ALDH1B1 |
| **2151** | *Folium Nelumbinis* | citric acid | O43324 | EEF1E1 |
| **2152** | *Folium Nelumbinis* | citric acid | P68106 | FKBP1B |
| **2153** | *Folium Nelumbinis* | citric acid | Q96BT7 | ALKBH8 |
| **2154** | *Folium Nelumbinis* | citric acid | O75891 | ALDH1L1 |
| **2155** | *Folium Nelumbinis* | citric acid | Q12904 | AIMP1 |
| **2156** | *Folium Nelumbinis* | citric acid | O43566 | RGS14 |
| **2157** | *Folium Nelumbinis* | citric acid | P49419 | ALDH7A1 |
| **2158** | *Folium Nelumbinis* | citric acid | Q5TF58 | IFFO2 |
| **2159** | *Folium Nelumbinis* | citric acid | P49798 | RGS4 |
| **2160** | *Folium Nelumbinis* | citric acid | Q13630 | TSTA3 |
| **2161** | *Folium Nelumbinis* | citric acid | O43709 | WBSCR22 |
| **2162** | *Folium Nelumbinis* | citric acid | P54687 | BCAT1 |
| **2163** | *Folium Nelumbinis* | citric acid | P49758 | RGS6 |
| **2164** | *Folium Nelumbinis* | citric acid | P61769 | B2M |
| **2165** | *Folium Nelumbinis* | Hexenal | P30838 | ALDH3A1 |
| **2166** | *Folium Nelumbinis* | Hexenal | P15121 | AKR1B1 |
| **2167** | *Folium Nelumbinis* | Hexenal | O60218 | AKR1B10 |
| **2168** | *Folium Nelumbinis* | higenamine | P42574 | CASP3 |
| **2169** | *Folium Nelumbinis* | higenamine | Q07812 | BAX |
| **2170** | *Folium Nelumbinis* | higenamine | P10415 | BCL2 |
| **2171** | *Folium Nelumbinis* | higenamine | P09601 | HMOX1 |
| **2172** | *Folium Nelumbinis* | higenamine | P35228 | NOS2 |
| **2173** | *Folium Nelumbinis* | Hirsutrin | P42574 | CASP3 |
| **2174** | *Folium Nelumbinis* | Hirsutrin | P04798 | CYP1A1 |
| **2175** | *Folium Nelumbinis* | Hirsutrin | P18825 | ADRA2C |
| **2176** | *Folium Nelumbinis* | isoliensinine | P00441 | SOD1 |
| **2177** | *Folium Nelumbinis* | isorhamnetin | P09601 | HMOX1 |
| **2178** | *Folium Nelumbinis* | isorhamnetin | Q9UBK8 | MTRR |
| **2179** | *Folium Nelumbinis* | isorhamnetin | P08183 | ABCB1 |
| **2180** | *Folium Nelumbinis* | isorhamnetin | P31749 | AKT1 |
| **2181** | *Folium Nelumbinis* | isorhamnetin | P29474 | NOS3 |
| **2182** | *Folium Nelumbinis* | isorhamnetin | P35228 | NOS2 |
| **2183** | *Folium Nelumbinis* | isorhamnetin | P45983 | MAPK8 |
| **2184** | *Folium Nelumbinis* | isorhamnetin | P04798 | CYP1A1 |
| **2185** | *Folium Nelumbinis* | isorhamnetin | P33527 | ABCC1 |
| **2186** | *Folium Nelumbinis* | isorhamnetin | P16435 | POR |
| **2187** | *Folium Nelumbinis* | kaempferol | P03372 | ESR1 |
| **2188** | *Folium Nelumbinis* | kaempferol | P28482 | MAPK1 |
| **2189** | *Folium Nelumbinis* | kaempferol | P09601 | HMOX1 |
| **2190** | *Folium Nelumbinis* | kaempferol | P25963 | NFKBIA |
| **2191** | *Folium Nelumbinis* | kaempferol | P08253 | MMP2 |
| **2192** | *Folium Nelumbinis* | kaempferol | P13500 | CCL2 |
| **2193** | *Folium Nelumbinis* | kaempferol | P35869 | AHR |
| **2194** | *Folium Nelumbinis* | kaempferol | P18054 | ALOX12 |
| **2195** | *Folium Nelumbinis* | kaempferol | P27361 | MAPK3 |
| **2196** | *Folium Nelumbinis* | kaempferol | P40763 | STAT3 |
| **2197** | *Folium Nelumbinis* | kaempferol | Q9UBK8 | MTRR |
| **2198** | *Folium Nelumbinis* | kaempferol | P36537 | UGT2B10 |
| **2199** | *Folium Nelumbinis* | kaempferol | P08183 | ABCB1 |
| **2200** | *Folium Nelumbinis* | kaempferol | Q14534 | SQLE |
| **2201** | *Folium Nelumbinis* | kaempferol | P24941 | CDK2 |
| **2202** | *Folium Nelumbinis* | kaempferol | P06400 | RB1 |
| **2203** | *Folium Nelumbinis* | kaempferol | P08069 | IGF1R |
| **2204** | *Folium Nelumbinis* | kaempferol | P04637 | TP53 |
| **2205** | *Folium Nelumbinis* | kaempferol | P31749 | AKT1 |
| **2206** | *Folium Nelumbinis* | kaempferol | Q13315 | ATM |
| **2207** | *Folium Nelumbinis* | kaempferol | P04141 | CSF2 |
| **2208** | *Folium Nelumbinis* | kaempferol | P29474 | NOS3 |
| **2209** | *Folium Nelumbinis* | kaempferol | P19224 | UGT1A6 |
| **2210** | *Folium Nelumbinis* | kaempferol | P22309 | UGT1A1 |
| **2211** | *Folium Nelumbinis* | kaempferol | P42574 | CASP3 |
| **2212** | *Folium Nelumbinis* | kaempferol | O75795 | UGT2B17 |
| **2213** | *Folium Nelumbinis* | kaempferol | P03956 | MMP1 |
| **2214** | *Folium Nelumbinis* | kaempferol | P35228 | NOS2 |
| **2215** | *Folium Nelumbinis* | kaempferol | P55211 | CASP9 |
| **2216** | *Folium Nelumbinis* | kaempferol | O75469 | NR1I2 |
| **2217** | *Folium Nelumbinis* | kaempferol | P08684 | CYP3A4 |
| **2218** | *Folium Nelumbinis* | kaempferol | P54855 | UGT2B15 |
| **2219** | *Folium Nelumbinis* | kaempferol | P05177 | CYP1A2 |
| **2220** | *Folium Nelumbinis* | kaempferol | Q9HAW8 | UGT1A10 |
| **2221** | *Folium Nelumbinis* | kaempferol | Q92731 | ESR2 |
| **2222** | *Folium Nelumbinis* | kaempferol | O60656 | UGT1A9 |
| **2223** | *Folium Nelumbinis* | kaempferol | P12931 | SRC |
| **2224** | *Folium Nelumbinis* | kaempferol | Q14994 | NR1I3 |
| **2225** | *Folium Nelumbinis* | kaempferol | O15111 | CHUK |
| **2226** | *Folium Nelumbinis* | kaempferol | P05412 | JUN |
| **2227** | *Folium Nelumbinis* | kaempferol | Q9HAW7 | UGT1A7 |
| **2228** | *Folium Nelumbinis* | kaempferol | Q9HAW9 | UGT1A8 |
| **2229** | *Folium Nelumbinis* | kaempferol | P04798 | CYP1A1 |
| **2230** | *Folium Nelumbinis* | kaempferol | P06493 | CDK1 |
| **2231** | *Folium Nelumbinis* | kaempferol | P09211 | GSTP1 |
| **2232** | *Folium Nelumbinis* | kaempferol | P33527 | ABCC1 |
| **2233** | *Folium Nelumbinis* | kaempferol | O75310 | UGT2B11 |
| **2234** | *Folium Nelumbinis* | kaempferol | P11166 | SLC2A1 |
| **2235** | *Folium Nelumbinis* | kaempferol | P35503 | UGT1A3 |
| **2236** | *Folium Nelumbinis* | kaempferol | P16435 | POR |
| **2237** | *Folium Nelumbinis* | liriodenine | P04637 | TP53 |
| **2238** | *Folium Nelumbinis* | Machiline | P21917 | DRD4 |
| **2239** | *Folium Nelumbinis* | MLT | P23946 | CMA1 |
| **2240** | *Folium Nelumbinis* | MLT | P53396 | ACLY |
| **2241** | *Folium Nelumbinis* | MLT | P49286 | MTNR1B |
| **2242** | *Folium Nelumbinis* | MLT | P31040 | SDHA |
| **2243** | *Folium Nelumbinis* | MLT | P02768 | ALB |
| **2244** | *Folium Nelumbinis* | MLT | P48039 | MTNR1A |
| **2245** | *Folium Nelumbinis* | MLT | P35558 | PCK1 |
| **2246** | *Folium Nelumbinis* | MLT | P28290 | CS |
| **2247** | *Folium Nelumbinis* | MLT | Q7L5Y1 | ENOSF1 |
| **2248** | *Folium Nelumbinis* | MLT | Q42521 | GAD1 |
| **2249** | *Folium Nelumbinis* | MLT | P21912 | SDHB |
| **2250** | *Folium Nelumbinis* | myricetin | Q96EB6 | SIRT1 |
| **2251** | *Folium Nelumbinis* | myricetin | P11712 | CYP2C9 |
| **2252** | *Folium Nelumbinis* | myricetin | Q8WTV0 | SCARB1 |
| **2253** | *Folium Nelumbinis* | myricetin | P42336 | PIK3CA |
| **2254** | *Folium Nelumbinis* | myricetin | P31749 | AKT1 |
| **2255** | *Folium Nelumbinis* | myricetin | Q9H2F3 | HSD3B7 |
| **2256** | *Folium Nelumbinis* | myricetin | P22303 | ACHE |
| **2257** | *Folium Nelumbinis* | myricetin | P42574 | CASP3 |
| **2258** | *Folium Nelumbinis* | myricetin | P55211 | CASP9 |
| **2259** | *Folium Nelumbinis* | myricetin | P08684 | CYP3A4 |
| **2260** | *Folium Nelumbinis* | myricetin | P42345 | MTOR |
| **2261** | *Folium Nelumbinis* | myricetin | P09874 | PARP1 |
| **2262** | *Folium Nelumbinis* | myricetin | P14060 | HSD3B1 |
| **2263** | *Folium Nelumbinis* | myricetin | P26439 | HSD3B2 |
| **2264** | *Folium Nelumbinis* | myricetin | P09917 | ALOX5 |
| **2265** | *Folium Nelumbinis* | myricetin | P04798 | CYP1A1 |
| **2266** | *Folium Nelumbinis* | myricetin | P33527 | ABCC1 |
| **2267** | *Folium Nelumbinis* | myricetin | P01375 | TNF |
| **2268** | *Folium Nelumbinis* | myricetin | Q13630 | TSTA3 |
| **2269** | *Folium Nelumbinis* | myricetin | P11388 | TOP2A |
| **2270** | *Folium Nelumbinis* | myricetin | P11166 | SLC2A1 |
| **2271** | *Folium Nelumbinis* | myristic acid | P01116 | KRAS |
| **2272** | *Folium Nelumbinis* | myristic acid | P37231 | PPARG |
| **2273** | *Folium Nelumbinis* | myristic acid | P02768 | ALB |
| **2274** | *Folium Nelumbinis* | myristic acid | P12931 | SRC |
| **2275** | *Folium Nelumbinis* | myristic acid | O00206 | TLR4 |
| **2276** | *Folium Nelumbinis* | myristic acid | P68106 | FKBP1B |
| **2277** | *Folium Nelumbinis* | myristic acid | P01275 | GCG |
| **2278** | *Folium Nelumbinis* | myristic acid | P15151 | PVR |
| **2279** | *Folium Nelumbinis* | myristic acid | P17252 | PRKCA |
| **2280** | *Folium Nelumbinis* | Nuciferin | P05177 | CYP1A2 |
| **2281** | *Folium Nelumbinis* | Pentenal | P15121 | AKR1B1 |
| **2282** | *Folium Nelumbinis* | PHB | P27169 | PON1 |
| **2283** | *Folium Nelumbinis* | PHB | Q14534 | SQLE |
| **2284** | *Folium Nelumbinis* | PHB | P50225 | SULT1A1 |
| **2285** | *Folium Nelumbinis* | quercetin | P13569 | CFTR |
| **2286** | *Folium Nelumbinis* | quercetin | P21917 | DRD4 |
| **2287** | *Folium Nelumbinis* | quercetin | P03372 | ESR1 |
| **2288** | *Folium Nelumbinis* | quercetin | Q96EB6 | SIRT1 |
| **2289** | *Folium Nelumbinis* | quercetin | P05546 | SERPIND1 |
| **2290** | *Folium Nelumbinis* | quercetin | P28482 | MAPK1 |
| **2291** | *Folium Nelumbinis* | quercetin | P09601 | HMOX1 |
| **2292** | *Folium Nelumbinis* | quercetin | P25963 | NFKBIA |
| **2293** | *Folium Nelumbinis* | quercetin | P08253 | MMP2 |
| **2294** | *Folium Nelumbinis* | quercetin | P00750 | PLAT |
| **2295** | *Folium Nelumbinis* | quercetin | P00390 | GSR |
| **2296** | *Folium Nelumbinis* | quercetin | P78329 | CYP4F2 |
| **2297** | *Folium Nelumbinis* | quercetin | P27169 | PON1 |
| **2298** | *Folium Nelumbinis* | quercetin | P43490 | NAMPT |
| **2299** | *Folium Nelumbinis* | quercetin | P05164 | MPO |
| **2300** | *Folium Nelumbinis* | quercetin | P13500 | CCL2 |
| **2301** | *Folium Nelumbinis* | quercetin | Q16539 | MAPK14 |
| **2302** | *Folium Nelumbinis* | quercetin | P04114 | APOB |
| **2303** | *Folium Nelumbinis* | quercetin | Q9UNQ0 | ABCG2 |
| **2304** | *Folium Nelumbinis* | quercetin | P04040 | CAT |
| **2305** | *Folium Nelumbinis* | quercetin | P35869 | AHR |
| **2306** | *Folium Nelumbinis* | quercetin | P38936 | CDKN1A |
| **2307** | *Folium Nelumbinis* | quercetin | P18054 | ALOX12 |
| **2308** | *Folium Nelumbinis* | quercetin | P05181 | CYP2E1 |
| **2309** | *Folium Nelumbinis* | quercetin | P01116 | KRAS |
| **2310** | *Folium Nelumbinis* | quercetin | P21589 | NT5E |
| **2311** | *Folium Nelumbinis* | quercetin | P05231 | IL6 |
| **2312** | *Folium Nelumbinis* | quercetin | O60603 | TLR2 |
| **2313** | *Folium Nelumbinis* | quercetin | P11712 | CYP2C9 |
| **2314** | *Folium Nelumbinis* | quercetin | Q07869 | PPARA |
| **2315** | *Folium Nelumbinis* | quercetin | P27361 | MAPK3 |
| **2316** | *Folium Nelumbinis* | quercetin | P14679 | TYR |
| **2317** | *Folium Nelumbinis* | quercetin | P01584 | IL1B |
| **2318** | *Folium Nelumbinis* | quercetin | P42336 | PIK3CA |
| **2319** | *Folium Nelumbinis* | quercetin | P06276 | BCHE |
| **2320** | *Folium Nelumbinis* | quercetin | P05362 | ICAM1 |
| **2321** | *Folium Nelumbinis* | quercetin | P36537 | UGT2B10 |
| **2322** | *Folium Nelumbinis* | quercetin | P08183 | ABCB1 |
| **2323** | *Folium Nelumbinis* | quercetin | Q14534 | SQLE |
| **2324** | *Folium Nelumbinis* | quercetin | P24941 | CDK2 |
| **2325** | *Folium Nelumbinis* | quercetin | P06400 | RB1 |
| **2326** | *Folium Nelumbinis* | quercetin | Q16548 | BCL2A1 |
| **2327** | *Folium Nelumbinis* | quercetin | P08069 | IGF1R |
| **2328** | *Folium Nelumbinis* | quercetin | P04637 | TP53 |
| **2329** | *Folium Nelumbinis* | quercetin | P31749 | AKT1 |
| **2330** | *Folium Nelumbinis* | quercetin | P00533 | EGFR |
| **2331** | *Folium Nelumbinis* | quercetin | O14763 | TNFRSF10B |
| **2332** | *Folium Nelumbinis* | quercetin | O60235 | TMPRSS11D |
| **2333** | *Folium Nelumbinis* | quercetin | P15121 | AKR1B1 |
| **2334** | *Folium Nelumbinis* | quercetin | Q9UHC9 | NPC1L1 |
| **2335** | *Folium Nelumbinis* | quercetin | Q07812 | BAX |
| **2336** | *Folium Nelumbinis* | quercetin | O60760 | HPGDS |
| **2337** | *Folium Nelumbinis* | quercetin | P04141 | CSF2 |
| **2338** | *Folium Nelumbinis* | quercetin | P29474 | NOS3 |
| **2339** | *Folium Nelumbinis* | quercetin | Q9H2F3 | HSD3B7 |
| **2340** | *Folium Nelumbinis* | quercetin | Q07817 | BCL2L1 |
| **2341** | *Folium Nelumbinis* | quercetin | P34932 | HSPA4 |
| **2342** | *Folium Nelumbinis* | quercetin | P19224 | UGT1A6 |
| **2343** | *Folium Nelumbinis* | quercetin | Q8N4T8 | CBR4 |
| **2344** | *Folium Nelumbinis* | quercetin | P22309 | UGT1A1 |
| **2345** | *Folium Nelumbinis* | quercetin | P35568 | IRS1 |
| **2346** | *Folium Nelumbinis* | quercetin | P02778 | CXCL10 |
| **2347** | *Folium Nelumbinis* | quercetin | P01100 | FOS |
| **2348** | *Folium Nelumbinis* | quercetin | P10145 | CXCL8 |
| **2349** | *Folium Nelumbinis* | quercetin | P99999 | CYCS |
| **2350** | *Folium Nelumbinis* | quercetin | Q9BZR8 | BCL2L14 |
| **2351** | *Folium Nelumbinis* | quercetin | P42574 | CASP3 |
| **2352** | *Folium Nelumbinis* | quercetin | Q9UMX3 | BOK |
| **2353** | *Folium Nelumbinis* | quercetin | P15559 | NQO1 |
| **2354** | *Folium Nelumbinis* | quercetin | O75795 | UGT2B17 |
| **2355** | *Folium Nelumbinis* | quercetin | Q15848 | ADIPOQ |
| **2356** | *Folium Nelumbinis* | quercetin | P14672 | SLC2A4 |
| **2357** | *Folium Nelumbinis* | quercetin | P50225 | SULT1A1 |
| **2358** | *Folium Nelumbinis* | quercetin | P03956 | MMP1 |
| **2359** | *Folium Nelumbinis* | quercetin | P49841 | GSK3B |
| **2360** | *Folium Nelumbinis* | quercetin | P35228 | NOS2 |
| **2361** | *Folium Nelumbinis* | quercetin | P10415 | BCL2 |
| **2362** | *Folium Nelumbinis* | quercetin | P55211 | CASP9 |
| **2363** | *Folium Nelumbinis* | quercetin | P48357 | LEPR |
| **2364** | *Folium Nelumbinis* | quercetin | O75469 | NR1I2 |
| **2365** | *Folium Nelumbinis* | quercetin | P08684 | CYP3A4 |
| **2366** | *Folium Nelumbinis* | quercetin | Q16665 | HIF1A |
| **2367** | *Folium Nelumbinis* | quercetin | P21397 | MAOA |
| **2368** | *Folium Nelumbinis* | quercetin | P54855 | UGT2B15 |
| **2369** | *Folium Nelumbinis* | quercetin | Q05397 | PTK2 |
| **2370** | *Folium Nelumbinis* | quercetin | P05177 | CYP1A2 |
| **2371** | *Folium Nelumbinis* | quercetin | Q9HAW8 | UGT1A10 |
| **2372** | *Folium Nelumbinis* | quercetin | Q92731 | ESR2 |
| **2373** | *Folium Nelumbinis* | quercetin | P35222 | CTNNB1 |
| **2374** | *Folium Nelumbinis* | quercetin | O60656 | UGT1A9 |
| **2375** | *Folium Nelumbinis* | quercetin | P12931 | SRC |
| **2376** | *Folium Nelumbinis* | quercetin | Q14790 | CASP8 |
| **2377** | *Folium Nelumbinis* | quercetin | P27540 | ARNT |
| **2378** | *Folium Nelumbinis* | quercetin | P45983 | MAPK8 |
| **2379** | *Folium Nelumbinis* | quercetin | P10635 | CYP2D6 |
| **2380** | *Folium Nelumbinis* | quercetin | P42345 | MTOR |
| **2381** | *Folium Nelumbinis* | quercetin | P23219 | PTGS1 |
| **2382** | *Folium Nelumbinis* | quercetin | P25929 | NPY1R |
| **2383** | *Folium Nelumbinis* | quercetin | P09874 | PARP1 |
| **2384** | *Folium Nelumbinis* | quercetin | Q14994 | NR1I3 |
| **2385** | *Folium Nelumbinis* | quercetin | Q07820 | MCL1 |
| **2386** | *Folium Nelumbinis* | quercetin | P55210 | CASP7 |
| **2387** | *Folium Nelumbinis* | quercetin | P14060 | HSD3B1 |
| **2388** | *Folium Nelumbinis* | quercetin | P26439 | HSD3B2 |
| **2389** | *Folium Nelumbinis* | quercetin | O15111 | CHUK |
| **2390** | *Folium Nelumbinis* | quercetin | P05412 | JUN |
| **2391** | *Folium Nelumbinis* | quercetin | P14780 | MMP9 |
| **2392** | *Folium Nelumbinis* | quercetin | P00749 | PLAU |
| **2393** | *Folium Nelumbinis* | quercetin | Q9HAW7 | UGT1A7 |
| **2394** | *Folium Nelumbinis* | quercetin | Q9HAW9 | UGT1A8 |
| **2395** | *Folium Nelumbinis* | quercetin | O00206 | TLR4 |
| **2396** | *Folium Nelumbinis* | quercetin | P09917 | ALOX5 |
| **2397** | *Folium Nelumbinis* | quercetin | P10275 | AR |
| **2398** | *Folium Nelumbinis* | quercetin | O95477 | ABCA1 |
| **2399** | *Folium Nelumbinis* | quercetin | P27338 | MAOB |
| **2400** | *Folium Nelumbinis* | quercetin | P05305 | EDN1 |
| **2401** | *Folium Nelumbinis* | quercetin | P47989 | XDH |
| **2402** | *Folium Nelumbinis* | quercetin | P04798 | CYP1A1 |
| **2403** | *Folium Nelumbinis* | quercetin | P42330 | AKR1C3 |
| **2404** | *Folium Nelumbinis* | quercetin | O60674 | JAK2 |
| **2405** | *Folium Nelumbinis* | quercetin | P06493 | CDK1 |
| **2406** | *Folium Nelumbinis* | quercetin | Q16236 | NFE2L2 |
| **2407** | *Folium Nelumbinis* | quercetin | P09211 | GSTP1 |
| **2408** | *Folium Nelumbinis* | quercetin | P33527 | ABCC1 |
| **2409** | *Folium Nelumbinis* | quercetin | P61073 | CXCR4 |
| **2410** | *Folium Nelumbinis* | quercetin | O75310 | UGT2B11 |
| **2411** | *Folium Nelumbinis* | quercetin | Q02880 | TOP2B |
| **2412** | *Folium Nelumbinis* | quercetin | P01375 | TNF |
| **2413** | *Folium Nelumbinis* | quercetin | Q13630 | TSTA3 |
| **2414** | *Folium Nelumbinis* | quercetin | P07203 | GPX1 |
| **2415** | *Folium Nelumbinis* | quercetin | P11388 | TOP2A |
| **2416** | *Folium Nelumbinis* | quercetin | P23560 | BDNF |
| **2417** | *Folium Nelumbinis* | quercetin | P11166 | SLC2A1 |
| **2418** | *Folium Nelumbinis* | quercetin | P35503 | UGT1A3 |
| **2419** | *Folium Nelumbinis* | quercetin | P16435 | POR |
| **2420** | *Folium Nelumbinis* | quercetin | P40313 | CTRL |
| **2421** | *Folium Nelumbinis* | Remerin | P21917 | DRD4 |
| **2422** | *Folium Nelumbinis* | RNG | P08253 | MMP2 |
| **2423** | *Folium Nelumbinis* | RNG | P30519 | HMOX2 |
| **2424** | *Folium Nelumbinis* | RNG | P05164 | MPO |
| **2425** | *Folium Nelumbinis* | RNG | P04040 | CAT |
| **2426** | *Folium Nelumbinis* | RNG | P38936 | CDKN1A |
| **2427** | *Folium Nelumbinis* | RNG | P05181 | CYP2E1 |
| **2428** | *Folium Nelumbinis* | RNG | P14679 | TYR |
| **2429** | *Folium Nelumbinis* | RNG | P06276 | BCHE |
| **2430** | *Folium Nelumbinis* | RNG | P24941 | CDK2 |
| **2431** | *Folium Nelumbinis* | RNG | P04637 | TP53 |
| **2432** | *Folium Nelumbinis* | RNG | P19224 | UGT1A6 |
| **2433** | *Folium Nelumbinis* | RNG | Q8N4T8 | CBR4 |
| **2434** | *Folium Nelumbinis* | RNG | P42574 | CASP3 |
| **2435** | *Folium Nelumbinis* | RNG | P15559 | NQO1 |
| **2436** | *Folium Nelumbinis* | RNG | P50225 | SULT1A1 |
| **2437** | *Folium Nelumbinis* | RNG | P55211 | CASP9 |
| **2438** | *Folium Nelumbinis* | RNG | P10635 | CYP2D6 |
| **2439** | *Folium Nelumbinis* | RNG | P09874 | PARP1 |
| **2440** | *Folium Nelumbinis* | RNG | P55210 | CASP7 |
| **2441** | *Folium Nelumbinis* | RNG | P05412 | JUN |
| **2442** | *Folium Nelumbinis* | RNG | Q9HAW7 | UGT1A7 |
| **2443** | *Folium Nelumbinis* | RNG | P04798 | CYP1A1 |
| **2444** | *Folium Nelumbinis* | RNG | P09211 | GSTP1 |
| **2445** | *Folium Nelumbinis* | RNG | Q04206 | RELA |
| **2446** | *Folium Nelumbinis* | RNG | P01375 | TNF |
| **2447** | *Folium Nelumbinis* | salicylic acid | P05164 | MPO |
| **2448** | *Folium Nelumbinis* | salicylic acid | P04040 | CAT |
| **2449** | *Folium Nelumbinis* | salicylic acid | P38936 | CDKN1A |
| **2450** | *Folium Nelumbinis* | salicylic acid | P18054 | ALOX12 |
| **2451** | *Folium Nelumbinis* | salicylic acid | P05181 | CYP2E1 |
| **2452** | *Folium Nelumbinis* | salicylic acid | Q8WTV0 | SCARB1 |
| **2453** | *Folium Nelumbinis* | salicylic acid | P04637 | TP53 |
| **2454** | *Folium Nelumbinis* | salicylic acid | O60235 | TMPRSS11D |
| **2455** | *Folium Nelumbinis* | salicylic acid | P02768 | ALB |
| **2456** | *Folium Nelumbinis* | salicylic acid | P99999 | CYCS |
| **2457** | *Folium Nelumbinis* | salicylic acid | P42574 | CASP3 |
| **2458** | *Folium Nelumbinis* | salicylic acid | P48357 | LEPR |
| **2459** | *Folium Nelumbinis* | salicylic acid | Q16665 | HIF1A |
| **2460** | *Folium Nelumbinis* | salicylic acid | Q9HAW8 | UGT1A10 |
| **2461** | *Folium Nelumbinis* | salicylic acid | P23219 | PTGS1 |
| **2462** | *Folium Nelumbinis* | salicylic acid | P09874 | PARP1 |
| **2463** | *Folium Nelumbinis* | salicylic acid | Q9HAW7 | UGT1A7 |
| **2464** | *Folium Nelumbinis* | salicylic acid | Q9HAW9 | UGT1A8 |
| **2465** | *Folium Nelumbinis* | salicylic acid | P47989 | XDH |
| **2466** | *Folium Nelumbinis* | salicylic acid | P42330 | AKR1C3 |
| **2467** | *Folium Nelumbinis* | salicylic acid | Q96BT7 | ALKBH8 |
| **2468** | *Folium Nelumbinis* | salicylic acid | O43709 | WBSCR22 |
| **2469** | *Folium Nelumbinis* | salicylic acid | P35503 | UGT1A3 |
| **2470** | *Folium Nelumbinis* | succinic acid | P21917 | DRD4 |
| **2471** | *Folium Nelumbinis* | succinic acid | P09622 | DLD |
| **2472** | *Folium Nelumbinis* | succinic acid | P05546 | SERPIND1 |
| **2473** | *Folium Nelumbinis* | succinic acid | P46663 | BDKRB1 |
| **2474** | *Folium Nelumbinis* | succinic acid | Q16698 | DECR1 |
| **2475** | *Folium Nelumbinis* | succinic acid | P00750 | PLAT |
| **2476** | *Folium Nelumbinis* | succinic acid | P00390 | GSR |
| **2477** | *Folium Nelumbinis* | succinic acid | P01137 | TGFB1 |
| **2478** | *Folium Nelumbinis* | succinic acid | P05121 | SERPINE1 |
| **2479** | *Folium Nelumbinis* | succinic acid | P01031 | C5 |
| **2480** | *Folium Nelumbinis* | succinic acid | P30838 | ALDH3A1 |
| **2481** | *Folium Nelumbinis* | succinic acid | P04040 | CAT |
| **2482** | *Folium Nelumbinis* | succinic acid | P00505 | GOT2 |
| **2483** | *Folium Nelumbinis* | succinic acid | P01024 | C3 |
| **2484** | *Folium Nelumbinis* | succinic acid | Q99705 | MCHR1 |
| **2485** | *Folium Nelumbinis* | succinic acid | O94788 | ALDH1A2 |
| **2486** | *Folium Nelumbinis* | succinic acid | P23946 | CMA1 |
| **2487** | *Folium Nelumbinis* | succinic acid | P53396 | ACLY |
| **2488** | *Folium Nelumbinis* | succinic acid | P48448 | ALDH3B2 |
| **2489** | *Folium Nelumbinis* | succinic acid | Q9GZQ4 | NMUR2 |
| **2490** | *Folium Nelumbinis* | succinic acid | P49286 | MTNR1B |
| **2491** | *Folium Nelumbinis* | succinic acid | P04083 | ANXA1 |
| **2492** | *Folium Nelumbinis* | succinic acid | Q3SY69 | ALDH1L2 |
| **2493** | *Folium Nelumbinis* | succinic acid | P05091 | ALDH2 |
| **2494** | *Folium Nelumbinis* | succinic acid | P48645 | NMU |
| **2495** | *Folium Nelumbinis* | succinic acid | P01189 | POMC |
| **2496** | *Folium Nelumbinis* | succinic acid | P31040 | SDHA |
| **2497** | *Folium Nelumbinis* | succinic acid | P01042 | KNG1 |
| **2498** | *Folium Nelumbinis* | succinic acid | Q9H2A2 | ALDH8A1 |
| **2499** | *Folium Nelumbinis* | succinic acid | Q13304 | GPR17 |
| **2500** | *Folium Nelumbinis* | succinic acid | P08913 | ADRA2A |
| **2501** | *Folium Nelumbinis* | succinic acid | Q969V1 | MCHR2 |
| **2502** | *Folium Nelumbinis* | succinic acid | O60235 | TMPRSS11D |
| **2503** | *Folium Nelumbinis* | succinic acid | P05067 | APP |
| **2504** | *Folium Nelumbinis* | succinic acid | P30038 | ALDH4A1 |
| **2505** | *Folium Nelumbinis* | succinic acid | Q8IZ83 | ALDH16A1 |
| **2506** | *Folium Nelumbinis* | succinic acid | Q9UNI1 | CELA1 |
| **2507** | *Folium Nelumbinis* | succinic acid | P02768 | ALB |
| **2508** | *Folium Nelumbinis* | succinic acid | O76081 | RGS20 |
| **2509** | *Folium Nelumbinis* | succinic acid | Q99527 | GPER1 |
| **2510** | *Folium Nelumbinis* | succinic acid | P00352 | ALDH1A1 |
| **2511** | *Folium Nelumbinis* | succinic acid | P48039 | MTNR1A |
| **2512** | *Folium Nelumbinis* | succinic acid | P02778 | CXCL10 |
| **2513** | *Folium Nelumbinis* | succinic acid | Q9HB89 | NMUR1 |
| **2514** | *Folium Nelumbinis* | succinic acid | P10145 | CXCL8 |
| **2515** | *Folium Nelumbinis* | succinic acid | P99999 | CYCS |
| **2516** | *Folium Nelumbinis* | succinic acid | P07477 | PRSS1 |
| **2517** | *Folium Nelumbinis* | succinic acid | P21399 | ACO1 |
| **2518** | *Folium Nelumbinis* | succinic acid | P51649 | ALDH5A1 |
| **2519** | *Folium Nelumbinis* | succinic acid | P34896 | SHMT1 |
| **2520** | *Folium Nelumbinis* | succinic acid | P07237 | P4HB |
| **2521** | *Folium Nelumbinis* | succinic acid | Q9H1C0 | LPAR5 |
| **2522** | *Folium Nelumbinis* | succinic acid | P20382 | PMCH |
| **2523** | *Folium Nelumbinis* | succinic acid | P47895 | ALDH1A3 |
| **2524** | *Folium Nelumbinis* | succinic acid | P49795 | RGS19 |
| **2525** | *Folium Nelumbinis* | succinic acid | P59768 | GNG2 |
| **2526** | *Folium Nelumbinis* | succinic acid | Q16665 | HIF1A |
| **2527** | *Folium Nelumbinis* | succinic acid | P25090 | FPR2 |
| **2528** | *Folium Nelumbinis* | succinic acid | P28290 | CS |
| **2529** | *Folium Nelumbinis* | succinic acid | P63096 | GNAI1 |
| **2530** | *Folium Nelumbinis* | succinic acid | P51648 | ALDH3A2 |
| **2531** | *Folium Nelumbinis* | succinic acid | P49189 | ALDH9A1 |
| **2532** | *Folium Nelumbinis* | succinic acid | P02751 | FN1 |
| **2533** | *Folium Nelumbinis* | succinic acid | P21730 | C5AR1 |
| **2534** | *Folium Nelumbinis* | succinic acid | P0DJI8 | SAA1 |
| **2535** | *Folium Nelumbinis* | succinic acid | Q42521 | GAD1 |
| **2536** | *Folium Nelumbinis* | succinic acid | Q92633 | LPAR1 |
| **2537** | *Folium Nelumbinis* | succinic acid | P35030 | PRSS3 |
| **2538** | *Folium Nelumbinis* | succinic acid | P25929 | NPY1R |
| **2539** | *Folium Nelumbinis* | succinic acid | Q8N5D0 | WDTC1 |
| **2540** | *Folium Nelumbinis* | succinic acid | P49802 | RGS7 |
| **2541** | *Folium Nelumbinis* | succinic acid | P01019 | AGT |
| **2542** | *Folium Nelumbinis* | succinic acid | Q08116 | RGS1 |
| **2543** | *Folium Nelumbinis* | succinic acid | Q9NS28 | RGS18 |
| **2544** | *Folium Nelumbinis* | succinic acid | O43665 | RGS10 |
| **2545** | *Folium Nelumbinis* | succinic acid | P08754 | GNAI3 |
| **2546** | *Folium Nelumbinis* | succinic acid | P17174 | GOT1 |
| **2547** | *Folium Nelumbinis* | succinic acid | Q9UBY5 | LPAR3 |
| **2548** | *Folium Nelumbinis* | succinic acid | P60484 | PTEN |
| **2549** | *Folium Nelumbinis* | succinic acid | P00749 | PLAU |
| **2550** | *Folium Nelumbinis* | succinic acid | P21912 | SDHB |
| **2551** | *Folium Nelumbinis* | succinic acid | Q5H8A3 | NMS |
| **2552** | *Folium Nelumbinis* | succinic acid | P30837 | ALDH1B1 |
| **2553** | *Folium Nelumbinis* | succinic acid | P68106 | FKBP1B |
| **2554** | *Folium Nelumbinis* | succinic acid | Q96BT7 | ALKBH8 |
| **2555** | *Folium Nelumbinis* | succinic acid | O75891 | ALDH1L1 |
| **2556** | *Folium Nelumbinis* | succinic acid | P09341 | CXCL1 |
| **2557** | *Folium Nelumbinis* | succinic acid | Q9HBW0 | LPAR2 |
| **2558** | *Folium Nelumbinis* | succinic acid | P18825 | ADRA2C |
| **2559** | *Folium Nelumbinis* | succinic acid | O43566 | RGS14 |
| **2560** | *Folium Nelumbinis* | succinic acid | P61073 | CXCR4 |
| **2561** | *Folium Nelumbinis* | succinic acid | P49419 | ALDH7A1 |
| **2562** | *Folium Nelumbinis* | succinic acid | Q5TF58 | IFFO2 |
| **2563** | *Folium Nelumbinis* | succinic acid | P35372 | OPRM1 |
| **2564** | *Folium Nelumbinis* | succinic acid | P49798 | RGS4 |
| **2565** | *Folium Nelumbinis* | succinic acid | P00739 | HPR |
| **2566** | *Folium Nelumbinis* | succinic acid | P49758 | RGS6 |
| **2567** | *Folium Nelumbinis* | succinic acid | P40313 | CTRL |
| **2568** | *Folium Nelumbinis* | Tar | P04040 | CAT |
| **2569** | *Folium Nelumbinis* | Tar | P23946 | CMA1 |
| **2570** | *Folium Nelumbinis* | Tar | Q7L5Y1 | ENOSF1 |
| **2571** | *Folium Nelumbinis* | Tar | O43175 | PHGDH |
| **2572** | *Folium Nelumbinis* | Tar | P17174 | GOT1 |
| **2573** | *Folium Nelumbinis* | Tar | P68106 | FKBP1B |
| **2574** | *Rhizoma Polygoni Cuspidati* | (+)-Limacine | P24385 | CCND1 |
| **2575** | *Rhizoma Polygoni Cuspidati* | (+)-Limacine | Q07075 | ENPEP |
| **2576** | *Rhizoma Polygoni Cuspidati* | (+)-Limacine | O60235 | TMPRSS11D |
| **2577** | *Rhizoma Polygoni Cuspidati* | 3,4,5-trihydroxybenzoic acid | P08253 | MMP2 |
| **2578** | *Rhizoma Polygoni Cuspidati* | 3,4,5-trihydroxybenzoic acid | P05121 | SERPINE1 |
| **2579** | *Rhizoma Polygoni Cuspidati* | 3,4,5-trihydroxybenzoic acid | P05164 | MPO |
| **2580** | *Rhizoma Polygoni Cuspidati* | 3,4,5-trihydroxybenzoic acid | P04114 | APOB |
| **2581** | *Rhizoma Polygoni Cuspidati* | 3,4,5-trihydroxybenzoic acid | P04040 | CAT |
| **2582** | *Rhizoma Polygoni Cuspidati* | 3,4,5-trihydroxybenzoic acid | P14679 | TYR |
| **2583** | *Rhizoma Polygoni Cuspidati* | 3,4,5-trihydroxybenzoic acid | P08183 | ABCB1 |
| **2584** | *Rhizoma Polygoni Cuspidati* | 3,4,5-trihydroxybenzoic acid | P31749 | AKT1 |
| **2585** | *Rhizoma Polygoni Cuspidati* | 3,4,5-trihydroxybenzoic acid | Q13315 | ATM |
| **2586** | *Rhizoma Polygoni Cuspidati* | 3,4,5-trihydroxybenzoic acid | P42574 | CASP3 |
| **2587** | *Rhizoma Polygoni Cuspidati* | 3,4,5-trihydroxybenzoic acid | O75795 | UGT2B17 |
| **2588** | *Rhizoma Polygoni Cuspidati* | 3,4,5-trihydroxybenzoic acid | P50225 | SULT1A1 |
| **2589** | *Rhizoma Polygoni Cuspidati* | 3,4,5-trihydroxybenzoic acid | P55210 | CASP7 |
| **2590** | *Rhizoma Polygoni Cuspidati* | 3,4,5-trihydroxybenzoic acid | O15111 | CHUK |
| **2591** | *Rhizoma Polygoni Cuspidati* | 3,4,5-trihydroxybenzoic acid | P05412 | JUN |
| **2592** | *Rhizoma Polygoni Cuspidati* | 3,4,5-trihydroxybenzoic acid | P14780 | MMP9 |
| **2593** | *Rhizoma Polygoni Cuspidati* | 3,4,5-trihydroxybenzoic acid | P17252 | PRKCA |
| **2594** | *Rhizoma Polygoni Cuspidati* | apigenin | P13569 | CFTR |
| **2595** | *Rhizoma Polygoni Cuspidati* | apigenin | P03372 | ESR1 |
| **2596** | *Rhizoma Polygoni Cuspidati* | apigenin | P28482 | MAPK1 |
| **2597** | *Rhizoma Polygoni Cuspidati* | apigenin | P24385 | CCND1 |
| **2598** | *Rhizoma Polygoni Cuspidati* | apigenin | P04040 | CAT |
| **2599** | *Rhizoma Polygoni Cuspidati* | apigenin | P27361 | MAPK3 |
| **2600** | *Rhizoma Polygoni Cuspidati* | apigenin | P01189 | POMC |
| **2601** | *Rhizoma Polygoni Cuspidati* | apigenin | P01133 | EGF |
| **2602** | *Rhizoma Polygoni Cuspidati* | apigenin | P08183 | ABCB1 |
| **2603** | *Rhizoma Polygoni Cuspidati* | apigenin | Q14534 | SQLE |
| **2604** | *Rhizoma Polygoni Cuspidati* | apigenin | P04637 | TP53 |
| **2605** | *Rhizoma Polygoni Cuspidati* | apigenin | P31749 | AKT1 |
| **2606** | *Rhizoma Polygoni Cuspidati* | apigenin | O14763 | TNFRSF10B |
| **2607** | *Rhizoma Polygoni Cuspidati* | apigenin | P19224 | UGT1A6 |
| **2608** | *Rhizoma Polygoni Cuspidati* | apigenin | P22303 | ACHE |
| **2609** | *Rhizoma Polygoni Cuspidati* | apigenin | P22309 | UGT1A1 |
| **2610** | *Rhizoma Polygoni Cuspidati* | apigenin | P42574 | CASP3 |
| **2611** | *Rhizoma Polygoni Cuspidati* | apigenin | P50225 | SULT1A1 |
| **2612** | *Rhizoma Polygoni Cuspidati* | apigenin | P10415 | BCL2 |
| **2613** | *Rhizoma Polygoni Cuspidati* | apigenin | P55211 | CASP9 |
| **2614** | *Rhizoma Polygoni Cuspidati* | apigenin | O75469 | NR1I2 |
| **2615** | *Rhizoma Polygoni Cuspidati* | apigenin | P08684 | CYP3A4 |
| **2616** | *Rhizoma Polygoni Cuspidati* | apigenin | Q16665 | HIF1A |
| **2617** | *Rhizoma Polygoni Cuspidati* | apigenin | P21397 | MAOA |
| **2618** | *Rhizoma Polygoni Cuspidati* | apigenin | P54855 | UGT2B15 |
| **2619** | *Rhizoma Polygoni Cuspidati* | apigenin | Q05397 | PTK2 |
| **2620** | *Rhizoma Polygoni Cuspidati* | apigenin | P05177 | CYP1A2 |
| **2621** | *Rhizoma Polygoni Cuspidati* | apigenin | Q9HAW8 | UGT1A10 |
| **2622** | *Rhizoma Polygoni Cuspidati* | apigenin | Q92731 | ESR2 |
| **2623** | *Rhizoma Polygoni Cuspidati* | apigenin | O60656 | UGT1A9 |
| **2624** | *Rhizoma Polygoni Cuspidati* | apigenin | Q42521 | GAD1 |
| **2625** | *Rhizoma Polygoni Cuspidati* | apigenin | P12931 | SRC |
| **2626** | *Rhizoma Polygoni Cuspidati* | apigenin | Q14790 | CASP8 |
| **2627** | *Rhizoma Polygoni Cuspidati* | apigenin | P09874 | PARP1 |
| **2628** | *Rhizoma Polygoni Cuspidati* | apigenin | P55210 | CASP7 |
| **2629** | *Rhizoma Polygoni Cuspidati* | apigenin | P14780 | MMP9 |
| **2630** | *Rhizoma Polygoni Cuspidati* | apigenin | Q9HAW7 | UGT1A7 |
| **2631** | *Rhizoma Polygoni Cuspidati* | apigenin | Q9HAW9 | UGT1A8 |
| **2632** | *Rhizoma Polygoni Cuspidati* | apigenin | P27338 | MAOB |
| **2633** | *Rhizoma Polygoni Cuspidati* | apigenin | P04798 | CYP1A1 |
| **2634** | *Rhizoma Polygoni Cuspidati* | apigenin | P06493 | CDK1 |
| **2635** | *Rhizoma Polygoni Cuspidati* | apigenin | P33527 | ABCC1 |
| **2636** | *Rhizoma Polygoni Cuspidati* | apigenin | P61073 | CXCR4 |
| **2637** | *Rhizoma Polygoni Cuspidati* | apigenin | Q02880 | TOP2B |
| **2638** | *Rhizoma Polygoni Cuspidati* | apigenin | P01375 | TNF |
| **2639** | *Rhizoma Polygoni Cuspidati* | apigenin | P11388 | TOP2A |
| **2640** | *Rhizoma Polygoni Cuspidati* | apigenin | P11166 | SLC2A1 |
| **2641** | *Rhizoma Polygoni Cuspidati* | beta-sitosterol | P02649 | APOE |
| **2642** | *Rhizoma Polygoni Cuspidati* | beta-sitosterol | P05362 | ICAM1 |
| **2643** | *Rhizoma Polygoni Cuspidati* | beta-sitosterol | Q9UHC9 | NPC1L1 |
| **2644** | *Rhizoma Polygoni Cuspidati* | beta-sitosterol | P42574 | CASP3 |
| **2645** | *Rhizoma Polygoni Cuspidati* | beta-sitosterol | P36956 | SREBF1 |
| **2646** | *Rhizoma Polygoni Cuspidati* | beta-sitosterol | O95477 | ABCA1 |
| **2647** | *Rhizoma Polygoni Cuspidati* | citric acid | Q16698 | DECR1 |
| **2648** | *Rhizoma Polygoni Cuspidati* | citric acid | P01137 | TGFB1 |
| **2649** | *Rhizoma Polygoni Cuspidati* | citric acid | P30838 | ALDH3A1 |
| **2650** | *Rhizoma Polygoni Cuspidati* | citric acid | O94788 | ALDH1A2 |
| **2651** | *Rhizoma Polygoni Cuspidati* | citric acid | P23946 | CMA1 |
| **2652** | *Rhizoma Polygoni Cuspidati* | citric acid | P53396 | ACLY |
| **2653** | *Rhizoma Polygoni Cuspidati* | citric acid | P48448 | ALDH3B2 |
| **2654** | *Rhizoma Polygoni Cuspidati* | citric acid | P01116 | KRAS |
| **2655** | *Rhizoma Polygoni Cuspidati* | citric acid | P21589 | NT5E |
| **2656** | *Rhizoma Polygoni Cuspidati* | citric acid | Q3SY69 | ALDH1L2 |
| **2657** | *Rhizoma Polygoni Cuspidati* | citric acid | P05091 | ALDH2 |
| **2658** | *Rhizoma Polygoni Cuspidati* | citric acid | P56192 | MARS |
| **2659** | *Rhizoma Polygoni Cuspidati* | citric acid | P31040 | SDHA |
| **2660** | *Rhizoma Polygoni Cuspidati* | citric acid | Q9H2A2 | ALDH8A1 |
| **2661** | *Rhizoma Polygoni Cuspidati* | citric acid | Q16548 | BCL2A1 |
| **2662** | *Rhizoma Polygoni Cuspidati* | citric acid | P04637 | TP53 |
| **2663** | *Rhizoma Polygoni Cuspidati* | citric acid | P62979 | RPS27A |
| **2664** | *Rhizoma Polygoni Cuspidati* | citric acid | P15121 | AKR1B1 |
| **2665** | *Rhizoma Polygoni Cuspidati* | citric acid | P04035 | HMGCR |
| **2666** | *Rhizoma Polygoni Cuspidati* | citric acid | P30038 | ALDH4A1 |
| **2667** | *Rhizoma Polygoni Cuspidati* | citric acid | Q8IZ83 | ALDH16A1 |
| **2668** | *Rhizoma Polygoni Cuspidati* | citric acid | Q9UNI1 | CELA1 |
| **2669** | *Rhizoma Polygoni Cuspidati* | citric acid | P02768 | ALB |
| **2670** | *Rhizoma Polygoni Cuspidati* | citric acid | O76081 | RGS20 |
| **2671** | *Rhizoma Polygoni Cuspidati* | citric acid | P00352 | ALDH1A1 |
| **2672** | *Rhizoma Polygoni Cuspidati* | citric acid | P30101 | PDIA3 |
| **2673** | *Rhizoma Polygoni Cuspidati* | citric acid | Q07817 | BCL2L1 |
| **2674** | *Rhizoma Polygoni Cuspidati* | citric acid | Q8N4T8 | CBR4 |
| **2675** | *Rhizoma Polygoni Cuspidati* | citric acid | P15104 | GLUL |
| **2676** | *Rhizoma Polygoni Cuspidati* | citric acid | P07477 | PRSS1 |
| **2677** | *Rhizoma Polygoni Cuspidati* | citric acid | Q9BZR8 | BCL2L14 |
| **2678** | *Rhizoma Polygoni Cuspidati* | citric acid | P21399 | ACO1 |
| **2679** | *Rhizoma Polygoni Cuspidati* | citric acid | Q9UMX3 | BOK |
| **2680** | *Rhizoma Polygoni Cuspidati* | citric acid | P51649 | ALDH5A1 |
| **2681** | *Rhizoma Polygoni Cuspidati* | citric acid | P34896 | SHMT1 |
| **2682** | *Rhizoma Polygoni Cuspidati* | citric acid | O15382 | BCAT2 |
| **2683** | *Rhizoma Polygoni Cuspidati* | citric acid | P49841 | GSK3B |
| **2684** | *Rhizoma Polygoni Cuspidati* | citric acid | P07237 | P4HB |
| **2685** | *Rhizoma Polygoni Cuspidati* | citric acid | P30536 | TSPO |
| **2686** | *Rhizoma Polygoni Cuspidati* | citric acid | P10415 | BCL2 |
| **2687** | *Rhizoma Polygoni Cuspidati* | citric acid | P47895 | ALDH1A3 |
| **2688** | *Rhizoma Polygoni Cuspidati* | citric acid | P49795 | RGS19 |
| **2689** | *Rhizoma Polygoni Cuspidati* | citric acid | P28290 | CS |
| **2690** | *Rhizoma Polygoni Cuspidati* | citric acid | Q96RQ9 | IL4I1 |
| **2691** | *Rhizoma Polygoni Cuspidati* | citric acid | P63096 | GNAI1 |
| **2692** | *Rhizoma Polygoni Cuspidati* | citric acid | P51648 | ALDH3A2 |
| **2693** | *Rhizoma Polygoni Cuspidati* | citric acid | Q7L5Y1 | ENOSF1 |
| **2694** | *Rhizoma Polygoni Cuspidati* | citric acid | P49189 | ALDH9A1 |
| **2695** | *Rhizoma Polygoni Cuspidati* | citric acid | P12931 | SRC |
| **2696** | *Rhizoma Polygoni Cuspidati* | citric acid | P35030 | PRSS3 |
| **2697** | *Rhizoma Polygoni Cuspidati* | citric acid | P49802 | RGS7 |
| **2698** | *Rhizoma Polygoni Cuspidati* | citric acid | Q08116 | RGS1 |
| **2699** | *Rhizoma Polygoni Cuspidati* | citric acid | Q9NS28 | RGS18 |
| **2700** | *Rhizoma Polygoni Cuspidati* | citric acid | Q07820 | MCL1 |
| **2701** | *Rhizoma Polygoni Cuspidati* | citric acid | O43665 | RGS10 |
| **2702** | *Rhizoma Polygoni Cuspidati* | citric acid | P55210 | CASP7 |
| **2703** | *Rhizoma Polygoni Cuspidati* | citric acid | O43175 | PHGDH |
| **2704** | *Rhizoma Polygoni Cuspidati* | citric acid | P08754 | GNAI3 |
| **2705** | *Rhizoma Polygoni Cuspidati* | citric acid | P60484 | PTEN |
| **2706** | *Rhizoma Polygoni Cuspidati* | citric acid | P00749 | PLAU |
| **2707** | *Rhizoma Polygoni Cuspidati* | citric acid | P21912 | SDHB |
| **2708** | *Rhizoma Polygoni Cuspidati* | citric acid | P29144 | TPP2 |
| **2709** | *Rhizoma Polygoni Cuspidati* | citric acid | P30837 | ALDH1B1 |
| **2710** | *Rhizoma Polygoni Cuspidati* | citric acid | O43324 | EEF1E1 |
| **2711** | *Rhizoma Polygoni Cuspidati* | citric acid | P68106 | FKBP1B |
| **2712** | *Rhizoma Polygoni Cuspidati* | citric acid | Q96BT7 | ALKBH8 |
| **2713** | *Rhizoma Polygoni Cuspidati* | citric acid | O75891 | ALDH1L1 |
| **2714** | *Rhizoma Polygoni Cuspidati* | citric acid | Q12904 | AIMP1 |
| **2715** | *Rhizoma Polygoni Cuspidati* | citric acid | O43566 | RGS14 |
| **2716** | *Rhizoma Polygoni Cuspidati* | citric acid | P49419 | ALDH7A1 |
| **2717** | *Rhizoma Polygoni Cuspidati* | citric acid | Q5TF58 | IFFO2 |
| **2718** | *Rhizoma Polygoni Cuspidati* | citric acid | P49798 | RGS4 |
| **2719** | *Rhizoma Polygoni Cuspidati* | citric acid | Q13630 | TSTA3 |
| **2720** | *Rhizoma Polygoni Cuspidati* | citric acid | O43709 | WBSCR22 |
| **2721** | *Rhizoma Polygoni Cuspidati* | citric acid | P54687 | BCAT1 |
| **2722** | *Rhizoma Polygoni Cuspidati* | citric acid | P49758 | RGS6 |
| **2723** | *Rhizoma Polygoni Cuspidati* | citric acid | P61769 | B2M |
| **2724** | *Rhizoma Polygoni Cuspidati* | coumarin | P21917 | DRD4 |
| **2725** | *Rhizoma Polygoni Cuspidati* | coumarin | P46663 | BDKRB1 |
| **2726** | *Rhizoma Polygoni Cuspidati* | coumarin | P01031 | C5 |
| **2727** | *Rhizoma Polygoni Cuspidati* | coumarin | P01024 | C3 |
| **2728** | *Rhizoma Polygoni Cuspidati* | coumarin | Q99705 | MCHR1 |
| **2729** | *Rhizoma Polygoni Cuspidati* | coumarin | Q9GZQ4 | NMUR2 |
| **2730** | *Rhizoma Polygoni Cuspidati* | coumarin | P49286 | MTNR1B |
| **2731** | *Rhizoma Polygoni Cuspidati* | coumarin | P04083 | ANXA1 |
| **2732** | *Rhizoma Polygoni Cuspidati* | coumarin | P48645 | NMU |
| **2733** | *Rhizoma Polygoni Cuspidati* | coumarin | P01189 | POMC |
| **2734** | *Rhizoma Polygoni Cuspidati* | coumarin | P01042 | KNG1 |
| **2735** | *Rhizoma Polygoni Cuspidati* | coumarin | Q13304 | GPR17 |
| **2736** | *Rhizoma Polygoni Cuspidati* | coumarin | P08913 | ADRA2A |
| **2737** | *Rhizoma Polygoni Cuspidati* | coumarin | Q969V1 | MCHR2 |
| **2738** | *Rhizoma Polygoni Cuspidati* | coumarin | P05067 | APP |
| **2739** | *Rhizoma Polygoni Cuspidati* | coumarin | O76081 | RGS20 |
| **2740** | *Rhizoma Polygoni Cuspidati* | coumarin | Q99527 | GPER1 |
| **2741** | *Rhizoma Polygoni Cuspidati* | coumarin | P48039 | MTNR1A |
| **2742** | *Rhizoma Polygoni Cuspidati* | coumarin | P02778 | CXCL10 |
| **2743** | *Rhizoma Polygoni Cuspidati* | coumarin | Q9HB89 | NMUR1 |
| **2744** | *Rhizoma Polygoni Cuspidati* | coumarin | P10145 | CXCL8 |
| **2745** | *Rhizoma Polygoni Cuspidati* | coumarin | Q9H1C0 | LPAR5 |
| **2746** | *Rhizoma Polygoni Cuspidati* | coumarin | P20382 | PMCH |
| **2747** | *Rhizoma Polygoni Cuspidati* | coumarin | P49795 | RGS19 |
| **2748** | *Rhizoma Polygoni Cuspidati* | coumarin | P59768 | GNG2 |
| **2749** | *Rhizoma Polygoni Cuspidati* | coumarin | P25090 | FPR2 |
| **2750** | *Rhizoma Polygoni Cuspidati* | coumarin | P63096 | GNAI1 |
| **2751** | *Rhizoma Polygoni Cuspidati* | coumarin | P21730 | C5AR1 |
| **2752** | *Rhizoma Polygoni Cuspidati* | coumarin | P0DJI8 | SAA1 |
| **2753** | *Rhizoma Polygoni Cuspidati* | coumarin | Q92633 | LPAR1 |
| **2754** | *Rhizoma Polygoni Cuspidati* | coumarin | P25929 | NPY1R |
| **2755** | *Rhizoma Polygoni Cuspidati* | coumarin | P49802 | RGS7 |
| **2756** | *Rhizoma Polygoni Cuspidati* | coumarin | P01019 | AGT |
| **2757** | *Rhizoma Polygoni Cuspidati* | coumarin | P09874 | PARP1 |
| **2758** | *Rhizoma Polygoni Cuspidati* | coumarin | Q08116 | RGS1 |
| **2759** | *Rhizoma Polygoni Cuspidati* | coumarin | Q9NS28 | RGS18 |
| **2760** | *Rhizoma Polygoni Cuspidati* | coumarin | O43665 | RGS10 |
| **2761** | *Rhizoma Polygoni Cuspidati* | coumarin | P08754 | GNAI3 |
| **2762** | *Rhizoma Polygoni Cuspidati* | coumarin | Q9UBY5 | LPAR3 |
| **2763** | *Rhizoma Polygoni Cuspidati* | coumarin | Q5H8A3 | NMS |
| **2764** | *Rhizoma Polygoni Cuspidati* | coumarin | P09341 | CXCL1 |
| **2765** | *Rhizoma Polygoni Cuspidati* | coumarin | Q9HBW0 | LPAR2 |
| **2766** | *Rhizoma Polygoni Cuspidati* | coumarin | P18825 | ADRA2C |
| **2767** | *Rhizoma Polygoni Cuspidati* | coumarin | O43566 | RGS14 |
| **2768** | *Rhizoma Polygoni Cuspidati* | coumarin | P61073 | CXCR4 |
| **2769** | *Rhizoma Polygoni Cuspidati* | coumarin | P35372 | OPRM1 |
| **2770** | *Rhizoma Polygoni Cuspidati* | coumarin | P49798 | RGS4 |
| **2771** | *Rhizoma Polygoni Cuspidati* | coumarin | P49758 | RGS6 |
| **2772** | *Rhizoma Polygoni Cuspidati* | Crysophanol | O60656 | UGT1A9 |
| **2773** | *Rhizoma Polygoni Cuspidati* | emodin | P03372 | ESR1 |
| **2774** | *Rhizoma Polygoni Cuspidati* | emodin | P40763 | STAT3 |
| **2775** | *Rhizoma Polygoni Cuspidati* | emodin | Q9UBK8 | MTRR |
| **2776** | *Rhizoma Polygoni Cuspidati* | emodin | Q16548 | BCL2A1 |
| **2777** | *Rhizoma Polygoni Cuspidati* | emodin | P04637 | TP53 |
| **2778** | *Rhizoma Polygoni Cuspidati* | emodin | P04626 | ERBB2 |
| **2779** | *Rhizoma Polygoni Cuspidati* | emodin | Q13315 | ATM |
| **2780** | *Rhizoma Polygoni Cuspidati* | emodin | P04141 | CSF2 |
| **2781** | *Rhizoma Polygoni Cuspidati* | emodin | P29474 | NOS3 |
| **2782** | *Rhizoma Polygoni Cuspidati* | emodin | Q07817 | BCL2L1 |
| **2783** | *Rhizoma Polygoni Cuspidati* | emodin | P22309 | UGT1A1 |
| **2784** | *Rhizoma Polygoni Cuspidati* | emodin | P99999 | CYCS |
| **2785** | *Rhizoma Polygoni Cuspidati* | emodin | Q9BZR8 | BCL2L14 |
| **2786** | *Rhizoma Polygoni Cuspidati* | emodin | P42574 | CASP3 |
| **2787** | *Rhizoma Polygoni Cuspidati* | emodin | Q9UMX3 | BOK |
| **2788** | *Rhizoma Polygoni Cuspidati* | emodin | Q15848 | ADIPOQ |
| **2789** | *Rhizoma Polygoni Cuspidati* | emodin | P14672 | SLC2A4 |
| **2790** | *Rhizoma Polygoni Cuspidati* | emodin | P35228 | NOS2 |
| **2791** | *Rhizoma Polygoni Cuspidati* | emodin | P10415 | BCL2 |
| **2792** | *Rhizoma Polygoni Cuspidati* | emodin | P54855 | UGT2B15 |
| **2793** | *Rhizoma Polygoni Cuspidati* | emodin | Q9HAW8 | UGT1A10 |
| **2794** | *Rhizoma Polygoni Cuspidati* | emodin | Q92731 | ESR2 |
| **2795** | *Rhizoma Polygoni Cuspidati* | emodin | O60656 | UGT1A9 |
| **2796** | *Rhizoma Polygoni Cuspidati* | emodin | P02751 | FN1 |
| **2797** | *Rhizoma Polygoni Cuspidati* | emodin | P12931 | SRC |
| **2798** | *Rhizoma Polygoni Cuspidati* | emodin | P09874 | PARP1 |
| **2799** | *Rhizoma Polygoni Cuspidati* | emodin | Q07820 | MCL1 |
| **2800** | *Rhizoma Polygoni Cuspidati* | emodin | P00749 | PLAU |
| **2801** | *Rhizoma Polygoni Cuspidati* | emodin | Q9HAW7 | UGT1A7 |
| **2802** | *Rhizoma Polygoni Cuspidati* | emodin | Q9HAW9 | UGT1A8 |
| **2803** | *Rhizoma Polygoni Cuspidati* | emodin | O60674 | JAK2 |
| **2804** | *Rhizoma Polygoni Cuspidati* | emodin | P61073 | CXCR4 |
| **2805** | *Rhizoma Polygoni Cuspidati* | emodin | P01375 | TNF |
| **2806** | *Rhizoma Polygoni Cuspidati* | emodin | P11388 | TOP2A |
| **2807** | *Rhizoma Polygoni Cuspidati* | emodin | P16435 | POR |
| **2808** | *Rhizoma Polygoni Cuspidati* | Hirsutrin | P42574 | CASP3 |
| **2809** | *Rhizoma Polygoni Cuspidati* | Hirsutrin | P04798 | CYP1A1 |
| **2810** | *Rhizoma Polygoni Cuspidati* | Hirsutrin | P18825 | ADRA2C |
| **2811** | *Rhizoma Polygoni Cuspidati* | luteolin | P28482 | MAPK1 |
| **2812** | *Rhizoma Polygoni Cuspidati* | luteolin | P09601 | HMOX1 |
| **2813** | *Rhizoma Polygoni Cuspidati* | luteolin | P01031 | C5 |
| **2814** | *Rhizoma Polygoni Cuspidati* | luteolin | P01024 | C3 |
| **2815** | *Rhizoma Polygoni Cuspidati* | luteolin | P27361 | MAPK3 |
| **2816** | *Rhizoma Polygoni Cuspidati* | luteolin | P01584 | IL1B |
| **2817** | *Rhizoma Polygoni Cuspidati* | luteolin | P40763 | STAT3 |
| **2818** | *Rhizoma Polygoni Cuspidati* | luteolin | Q9UBK8 | MTRR |
| **2819** | *Rhizoma Polygoni Cuspidati* | luteolin | P24941 | CDK2 |
| **2820** | *Rhizoma Polygoni Cuspidati* | luteolin | P04637 | TP53 |
| **2821** | *Rhizoma Polygoni Cuspidati* | luteolin | P04626 | ERBB2 |
| **2822** | *Rhizoma Polygoni Cuspidati* | luteolin | P31749 | AKT1 |
| **2823** | *Rhizoma Polygoni Cuspidati* | luteolin | P00533 | EGFR |
| **2824** | *Rhizoma Polygoni Cuspidati* | luteolin | O14763 | TNFRSF10B |
| **2825** | *Rhizoma Polygoni Cuspidati* | luteolin | P15121 | AKR1B1 |
| **2826** | *Rhizoma Polygoni Cuspidati* | luteolin | P37231 | PPARG |
| **2827** | *Rhizoma Polygoni Cuspidati* | luteolin | P29474 | NOS3 |
| **2828** | *Rhizoma Polygoni Cuspidati* | luteolin | Q07817 | BCL2L1 |
| **2829** | *Rhizoma Polygoni Cuspidati* | luteolin | P35568 | IRS1 |
| **2830** | *Rhizoma Polygoni Cuspidati* | luteolin | P01100 | FOS |
| **2831** | *Rhizoma Polygoni Cuspidati* | luteolin | P42574 | CASP3 |
| **2832** | *Rhizoma Polygoni Cuspidati* | luteolin | P35558 | PCK1 |
| **2833** | *Rhizoma Polygoni Cuspidati* | luteolin | P35228 | NOS2 |
| **2834** | *Rhizoma Polygoni Cuspidati* | luteolin | P55211 | CASP9 |
| **2835** | *Rhizoma Polygoni Cuspidati* | luteolin | Q05397 | PTK2 |
| **2836** | *Rhizoma Polygoni Cuspidati* | luteolin | Q92731 | ESR2 |
| **2837** | *Rhizoma Polygoni Cuspidati* | luteolin | P02751 | FN1 |
| **2838** | *Rhizoma Polygoni Cuspidati* | luteolin | P45983 | MAPK8 |
| **2839** | *Rhizoma Polygoni Cuspidati* | luteolin | P11387 | TOP1 |
| **2840** | *Rhizoma Polygoni Cuspidati* | luteolin | P42345 | MTOR |
| **2841** | *Rhizoma Polygoni Cuspidati* | luteolin | P01019 | AGT |
| **2842** | *Rhizoma Polygoni Cuspidati* | luteolin | P55210 | CASP7 |
| **2843** | *Rhizoma Polygoni Cuspidati* | luteolin | P05412 | JUN |
| **2844** | *Rhizoma Polygoni Cuspidati* | luteolin | P14780 | MMP9 |
| **2845** | *Rhizoma Polygoni Cuspidati* | luteolin | O00206 | TLR4 |
| **2846** | *Rhizoma Polygoni Cuspidati* | luteolin | P47989 | XDH |
| **2847** | *Rhizoma Polygoni Cuspidati* | luteolin | P04798 | CYP1A1 |
| **2848** | *Rhizoma Polygoni Cuspidati* | luteolin | Q16236 | NFE2L2 |
| **2849** | *Rhizoma Polygoni Cuspidati* | luteolin | P33527 | ABCC1 |
| **2850** | *Rhizoma Polygoni Cuspidati* | luteolin | P01375 | TNF |
| **2851** | *Rhizoma Polygoni Cuspidati* | luteolin | P35503 | UGT1A3 |
| **2852** | *Rhizoma Polygoni Cuspidati* | luteolin | P16435 | POR |
| **2853** | *Rhizoma Polygoni Cuspidati* | luteolin-7-o-glucoside | P09601 | HMOX1 |
| **2854** | *Rhizoma Polygoni Cuspidati* | luteolin-7-o-glucoside | Q9UBK8 | MTRR |
| **2855** | *Rhizoma Polygoni Cuspidati* | luteolin-7-o-glucoside | P29474 | NOS3 |
| **2856** | *Rhizoma Polygoni Cuspidati* | luteolin-7-o-glucoside | P35228 | NOS2 |
| **2857** | *Rhizoma Polygoni Cuspidati* | luteolin-7-o-glucoside | P16435 | POR |
| **2858** | *Rhizoma Polygoni Cuspidati* | MLT | P23946 | CMA1 |
| **2859** | *Rhizoma Polygoni Cuspidati* | MLT | P53396 | ACLY |
| **2860** | *Rhizoma Polygoni Cuspidati* | MLT | P49286 | MTNR1B |
| **2861** | *Rhizoma Polygoni Cuspidati* | MLT | P31040 | SDHA |
| **2862** | *Rhizoma Polygoni Cuspidati* | MLT | P02768 | ALB |
| **2863** | *Rhizoma Polygoni Cuspidati* | MLT | P48039 | MTNR1A |
| **2864** | *Rhizoma Polygoni Cuspidati* | MLT | P35558 | PCK1 |
| **2865** | *Rhizoma Polygoni Cuspidati* | MLT | P28290 | CS |
| **2866** | *Rhizoma Polygoni Cuspidati* | MLT | Q7L5Y1 | ENOSF1 |
| **2867** | *Rhizoma Polygoni Cuspidati* | MLT | Q42521 | GAD1 |
| **2868** | *Rhizoma Polygoni Cuspidati* | MLT | P21912 | SDHB |
| **2869** | *Rhizoma Polygoni Cuspidati* | oleanolic acid | P43490 | NAMPT |
| **2870** | *Rhizoma Polygoni Cuspidati* | oleanolic acid | Q07869 | PPARA |
| **2871** | *Rhizoma Polygoni Cuspidati* | oleanolic acid | P36537 | UGT2B10 |
| **2872** | *Rhizoma Polygoni Cuspidati* | oleanolic acid | P42574 | CASP3 |
| **2873** | *Rhizoma Polygoni Cuspidati* | oleanolic acid | P15559 | NQO1 |
| **2874** | *Rhizoma Polygoni Cuspidati* | oleanolic acid | P08684 | CYP3A4 |
| **2875** | *Rhizoma Polygoni Cuspidati* | oleanolic acid | P05177 | CYP1A2 |
| **2876** | *Rhizoma Polygoni Cuspidati* | oleanolic acid | Q14790 | CASP8 |
| **2877** | *Rhizoma Polygoni Cuspidati* | oleanolic acid | O60218 | AKR1B10 |
| **2878** | *Rhizoma Polygoni Cuspidati* | oleanolic acid | P11387 | TOP1 |
| **2879** | *Rhizoma Polygoni Cuspidati* | oleanolic acid | Q16236 | NFE2L2 |
| **2880** | *Rhizoma Polygoni Cuspidati* | oleanolic acid | O75310 | UGT2B11 |
| **2881** | *Rhizoma Polygoni Cuspidati* | oleanolic acid | P11388 | TOP2A |
| **2882** | *Rhizoma Polygoni Cuspidati* | physcion | O60656 | UGT1A9 |
| **2883** | *Rhizoma Polygoni Cuspidati* | physovenine | P06276 | BCHE |
| **2884** | *Rhizoma Polygoni Cuspidati* | physovenine | P22303 | ACHE |
| **2885** | *Rhizoma Polygoni Cuspidati* | polydatin | P28482 | MAPK1 |
| **2886** | *Rhizoma Polygoni Cuspidati* | polydatin | P13500 | CCL2 |
| **2887** | *Rhizoma Polygoni Cuspidati* | polydatin | P35869 | AHR |
| **2888** | *Rhizoma Polygoni Cuspidati* | polydatin | P27361 | MAPK3 |
| **2889** | *Rhizoma Polygoni Cuspidati* | polydatin | P14679 | TYR |
| **2890** | *Rhizoma Polygoni Cuspidati* | polydatin | P02778 | CXCL10 |
| **2891** | *Rhizoma Polygoni Cuspidati* | polydatin | P10145 | CXCL8 |
| **2892** | *Rhizoma Polygoni Cuspidati* | polydatin | P27540 | ARNT |
| **2893** | *Rhizoma Polygoni Cuspidati* | protocatechuic acid | P05164 | MPO |
| **2894** | *Rhizoma Polygoni Cuspidati* | quercetin | P13569 | CFTR |
| **2895** | *Rhizoma Polygoni Cuspidati* | quercetin | P21917 | DRD4 |
| **2896** | *Rhizoma Polygoni Cuspidati* | quercetin | P03372 | ESR1 |
| **2897** | *Rhizoma Polygoni Cuspidati* | quercetin | Q96EB6 | SIRT1 |
| **2898** | *Rhizoma Polygoni Cuspidati* | quercetin | P05546 | SERPIND1 |
| **2899** | *Rhizoma Polygoni Cuspidati* | quercetin | P28482 | MAPK1 |
| **2900** | *Rhizoma Polygoni Cuspidati* | quercetin | P09601 | HMOX1 |
| **2901** | *Rhizoma Polygoni Cuspidati* | quercetin | P25963 | NFKBIA |
| **2902** | *Rhizoma Polygoni Cuspidati* | quercetin | P08253 | MMP2 |
| **2903** | *Rhizoma Polygoni Cuspidati* | quercetin | P00750 | PLAT |
| **2904** | *Rhizoma Polygoni Cuspidati* | quercetin | P00390 | GSR |
| **2905** | *Rhizoma Polygoni Cuspidati* | quercetin | P78329 | CYP4F2 |
| **2906** | *Rhizoma Polygoni Cuspidati* | quercetin | P27169 | PON1 |
| **2907** | *Rhizoma Polygoni Cuspidati* | quercetin | P43490 | NAMPT |
| **2908** | *Rhizoma Polygoni Cuspidati* | quercetin | P05164 | MPO |
| **2909** | *Rhizoma Polygoni Cuspidati* | quercetin | P13500 | CCL2 |
| **2910** | *Rhizoma Polygoni Cuspidati* | quercetin | Q16539 | MAPK14 |
| **2911** | *Rhizoma Polygoni Cuspidati* | quercetin | P04114 | APOB |
| **2912** | *Rhizoma Polygoni Cuspidati* | quercetin | Q9UNQ0 | ABCG2 |
| **2913** | *Rhizoma Polygoni Cuspidati* | quercetin | P04040 | CAT |
| **2914** | *Rhizoma Polygoni Cuspidati* | quercetin | P35869 | AHR |
| **2915** | *Rhizoma Polygoni Cuspidati* | quercetin | P38936 | CDKN1A |
| **2916** | *Rhizoma Polygoni Cuspidati* | quercetin | P18054 | ALOX12 |
| **2917** | *Rhizoma Polygoni Cuspidati* | quercetin | P05181 | CYP2E1 |
| **2918** | *Rhizoma Polygoni Cuspidati* | quercetin | P01116 | KRAS |
| **2919** | *Rhizoma Polygoni Cuspidati* | quercetin | P21589 | NT5E |
| **2920** | *Rhizoma Polygoni Cuspidati* | quercetin | P05231 | IL6 |
| **2921** | *Rhizoma Polygoni Cuspidati* | quercetin | O60603 | TLR2 |
| **2922** | *Rhizoma Polygoni Cuspidati* | quercetin | P11712 | CYP2C9 |
| **2923** | *Rhizoma Polygoni Cuspidati* | quercetin | Q07869 | PPARA |
| **2924** | *Rhizoma Polygoni Cuspidati* | quercetin | P27361 | MAPK3 |
| **2925** | *Rhizoma Polygoni Cuspidati* | quercetin | P14679 | TYR |
| **2926** | *Rhizoma Polygoni Cuspidati* | quercetin | P01584 | IL1B |
| **2927** | *Rhizoma Polygoni Cuspidati* | quercetin | P42336 | PIK3CA |
| **2928** | *Rhizoma Polygoni Cuspidati* | quercetin | P06276 | BCHE |
| **2929** | *Rhizoma Polygoni Cuspidati* | quercetin | P05362 | ICAM1 |
| **2930** | *Rhizoma Polygoni Cuspidati* | quercetin | P36537 | UGT2B10 |
| **2931** | *Rhizoma Polygoni Cuspidati* | quercetin | P08183 | ABCB1 |
| **2932** | *Rhizoma Polygoni Cuspidati* | quercetin | Q14534 | SQLE |
| **2933** | *Rhizoma Polygoni Cuspidati* | quercetin | P24941 | CDK2 |
| **2934** | *Rhizoma Polygoni Cuspidati* | quercetin | P06400 | RB1 |
| **2935** | *Rhizoma Polygoni Cuspidati* | quercetin | Q16548 | BCL2A1 |
| **2936** | *Rhizoma Polygoni Cuspidati* | quercetin | P08069 | IGF1R |
| **2937** | *Rhizoma Polygoni Cuspidati* | quercetin | P04637 | TP53 |
| **2938** | *Rhizoma Polygoni Cuspidati* | quercetin | P31749 | AKT1 |
| **2939** | *Rhizoma Polygoni Cuspidati* | quercetin | P00533 | EGFR |
| **2940** | *Rhizoma Polygoni Cuspidati* | quercetin | O14763 | TNFRSF10B |
| **2941** | *Rhizoma Polygoni Cuspidati* | quercetin | O60235 | TMPRSS11D |
| **2942** | *Rhizoma Polygoni Cuspidati* | quercetin | P15121 | AKR1B1 |
| **2943** | *Rhizoma Polygoni Cuspidati* | quercetin | Q9UHC9 | NPC1L1 |
| **2944** | *Rhizoma Polygoni Cuspidati* | quercetin | Q07812 | BAX |
| **2945** | *Rhizoma Polygoni Cuspidati* | quercetin | O60760 | HPGDS |
| **2946** | *Rhizoma Polygoni Cuspidati* | quercetin | P04141 | CSF2 |
| **2947** | *Rhizoma Polygoni Cuspidati* | quercetin | P29474 | NOS3 |
| **2948** | *Rhizoma Polygoni Cuspidati* | quercetin | Q9H2F3 | HSD3B7 |
| **2949** | *Rhizoma Polygoni Cuspidati* | quercetin | Q07817 | BCL2L1 |
| **2950** | *Rhizoma Polygoni Cuspidati* | quercetin | P34932 | HSPA4 |
| **2951** | *Rhizoma Polygoni Cuspidati* | quercetin | P19224 | UGT1A6 |
| **2952** | *Rhizoma Polygoni Cuspidati* | quercetin | Q8N4T8 | CBR4 |
| **2953** | *Rhizoma Polygoni Cuspidati* | quercetin | P22309 | UGT1A1 |
| **2954** | *Rhizoma Polygoni Cuspidati* | quercetin | P35568 | IRS1 |
| **2955** | *Rhizoma Polygoni Cuspidati* | quercetin | P02778 | CXCL10 |
| **2956** | *Rhizoma Polygoni Cuspidati* | quercetin | P01100 | FOS |
| **2957** | *Rhizoma Polygoni Cuspidati* | quercetin | P10145 | CXCL8 |
| **2958** | *Rhizoma Polygoni Cuspidati* | quercetin | P99999 | CYCS |
| **2959** | *Rhizoma Polygoni Cuspidati* | quercetin | Q9BZR8 | BCL2L14 |
| **2960** | *Rhizoma Polygoni Cuspidati* | quercetin | P42574 | CASP3 |
| **2961** | *Rhizoma Polygoni Cuspidati* | quercetin | Q9UMX3 | BOK |
| **2962** | *Rhizoma Polygoni Cuspidati* | quercetin | P15559 | NQO1 |
| **2963** | *Rhizoma Polygoni Cuspidati* | quercetin | O75795 | UGT2B17 |
| **2964** | *Rhizoma Polygoni Cuspidati* | quercetin | Q15848 | ADIPOQ |
| **2965** | *Rhizoma Polygoni Cuspidati* | quercetin | P14672 | SLC2A4 |
| **2966** | *Rhizoma Polygoni Cuspidati* | quercetin | P50225 | SULT1A1 |
| **2967** | *Rhizoma Polygoni Cuspidati* | quercetin | P03956 | MMP1 |
| **2968** | *Rhizoma Polygoni Cuspidati* | quercetin | P49841 | GSK3B |
| **2969** | *Rhizoma Polygoni Cuspidati* | quercetin | P35228 | NOS2 |
| **2970** | *Rhizoma Polygoni Cuspidati* | quercetin | P10415 | BCL2 |
| **2971** | *Rhizoma Polygoni Cuspidati* | quercetin | P55211 | CASP9 |
| **2972** | *Rhizoma Polygoni Cuspidati* | quercetin | P48357 | LEPR |
| **2973** | *Rhizoma Polygoni Cuspidati* | quercetin | O75469 | NR1I2 |
| **2974** | *Rhizoma Polygoni Cuspidati* | quercetin | P08684 | CYP3A4 |
| **2975** | *Rhizoma Polygoni Cuspidati* | quercetin | Q16665 | HIF1A |
| **2976** | *Rhizoma Polygoni Cuspidati* | quercetin | P21397 | MAOA |
| **2977** | *Rhizoma Polygoni Cuspidati* | quercetin | P54855 | UGT2B15 |
| **2978** | *Rhizoma Polygoni Cuspidati* | quercetin | Q05397 | PTK2 |
| **2979** | *Rhizoma Polygoni Cuspidati* | quercetin | P05177 | CYP1A2 |
| **2980** | *Rhizoma Polygoni Cuspidati* | quercetin | Q9HAW8 | UGT1A10 |
| **2981** | *Rhizoma Polygoni Cuspidati* | quercetin | Q92731 | ESR2 |
| **2982** | *Rhizoma Polygoni Cuspidati* | quercetin | P35222 | CTNNB1 |
| **2983** | *Rhizoma Polygoni Cuspidati* | quercetin | O60656 | UGT1A9 |
| **2984** | *Rhizoma Polygoni Cuspidati* | quercetin | P12931 | SRC |
| **2985** | *Rhizoma Polygoni Cuspidati* | quercetin | Q14790 | CASP8 |
| **2986** | *Rhizoma Polygoni Cuspidati* | quercetin | P27540 | ARNT |
| **2987** | *Rhizoma Polygoni Cuspidati* | quercetin | P45983 | MAPK8 |
| **2988** | *Rhizoma Polygoni Cuspidati* | quercetin | P10635 | CYP2D6 |
| **2989** | *Rhizoma Polygoni Cuspidati* | quercetin | P42345 | MTOR |
| **2990** | *Rhizoma Polygoni Cuspidati* | quercetin | P23219 | PTGS1 |
| **2991** | *Rhizoma Polygoni Cuspidati* | quercetin | P25929 | NPY1R |
| **2992** | *Rhizoma Polygoni Cuspidati* | quercetin | P09874 | PARP1 |
| **2993** | *Rhizoma Polygoni Cuspidati* | quercetin | Q14994 | NR1I3 |
| **2994** | *Rhizoma Polygoni Cuspidati* | quercetin | Q07820 | MCL1 |
| **2995** | *Rhizoma Polygoni Cuspidati* | quercetin | P55210 | CASP7 |
| **2996** | *Rhizoma Polygoni Cuspidati* | quercetin | P14060 | HSD3B1 |
| **2997** | *Rhizoma Polygoni Cuspidati* | quercetin | P26439 | HSD3B2 |
| **2998** | *Rhizoma Polygoni Cuspidati* | quercetin | O15111 | CHUK |
| **2999** | *Rhizoma Polygoni Cuspidati* | quercetin | P05412 | JUN |
| **3000** | *Rhizoma Polygoni Cuspidati* | quercetin | P14780 | MMP9 |
| **3001** | *Rhizoma Polygoni Cuspidati* | quercetin | P00749 | PLAU |
| **3002** | *Rhizoma Polygoni Cuspidati* | quercetin | Q9HAW7 | UGT1A7 |
| **3003** | *Rhizoma Polygoni Cuspidati* | quercetin | Q9HAW9 | UGT1A8 |
| **3004** | *Rhizoma Polygoni Cuspidati* | quercetin | O00206 | TLR4 |
| **3005** | *Rhizoma Polygoni Cuspidati* | quercetin | P09917 | ALOX5 |
| **3006** | *Rhizoma Polygoni Cuspidati* | quercetin | P10275 | AR |
| **3007** | *Rhizoma Polygoni Cuspidati* | quercetin | O95477 | ABCA1 |
| **3008** | *Rhizoma Polygoni Cuspidati* | quercetin | P27338 | MAOB |
| **3009** | *Rhizoma Polygoni Cuspidati* | quercetin | P05305 | EDN1 |
| **3010** | *Rhizoma Polygoni Cuspidati* | quercetin | P47989 | XDH |
| **3011** | *Rhizoma Polygoni Cuspidati* | quercetin | P04798 | CYP1A1 |
| **3012** | *Rhizoma Polygoni Cuspidati* | quercetin | P42330 | AKR1C3 |
| **3013** | *Rhizoma Polygoni Cuspidati* | quercetin | O60674 | JAK2 |
| **3014** | *Rhizoma Polygoni Cuspidati* | quercetin | P06493 | CDK1 |
| **3015** | *Rhizoma Polygoni Cuspidati* | quercetin | Q16236 | NFE2L2 |
| **3016** | *Rhizoma Polygoni Cuspidati* | quercetin | P09211 | GSTP1 |
| **3017** | *Rhizoma Polygoni Cuspidati* | quercetin | P33527 | ABCC1 |
| **3018** | *Rhizoma Polygoni Cuspidati* | quercetin | P61073 | CXCR4 |
| **3019** | *Rhizoma Polygoni Cuspidati* | quercetin | O75310 | UGT2B11 |
| **3020** | *Rhizoma Polygoni Cuspidati* | quercetin | Q02880 | TOP2B |
| **3021** | *Rhizoma Polygoni Cuspidati* | quercetin | P01375 | TNF |
| **3022** | *Rhizoma Polygoni Cuspidati* | quercetin | Q13630 | TSTA3 |
| **3023** | *Rhizoma Polygoni Cuspidati* | quercetin | P07203 | GPX1 |
| **3024** | *Rhizoma Polygoni Cuspidati* | quercetin | P11388 | TOP2A |
| **3025** | *Rhizoma Polygoni Cuspidati* | quercetin | P23560 | BDNF |
| **3026** | *Rhizoma Polygoni Cuspidati* | quercetin | P11166 | SLC2A1 |
| **3027** | *Rhizoma Polygoni Cuspidati* | quercetin | P35503 | UGT1A3 |
| **3028** | *Rhizoma Polygoni Cuspidati* | quercetin | P16435 | POR |
| **3029** | *Rhizoma Polygoni Cuspidati* | quercetin | P40313 | CTRL |
| **3030** | *Rhizoma Polygoni Cuspidati* | resveratrol | Q96EB6 | SIRT1 |
| **3031** | *Rhizoma Polygoni Cuspidati* | resveratrol | P47989 | XDH |
| **3032** | *Rhizoma Polygoni Cuspidati* | resveratrol | P05164 | MPO |
| **3033** | *Rhizoma Polygoni Cuspidati* | resveratrol | P03372 | ESR1 |
| **3034** | *Rhizoma Polygoni Cuspidati* | resveratrol | Q92731 | ESR2 |
| **3035** | *Rhizoma Polygoni Cuspidati* | resveratrol | P04798 | CYP1A1 |
| **3036** | *Rhizoma Polygoni Cuspidati* | resveratrol | P35869 | AHR |
| **3037** | *Rhizoma Polygoni Cuspidati* | resveratrol | P04637 | TP53 |
| **3038** | *Rhizoma Polygoni Cuspidati* | resveratrol | P01100 | FOS |
| **3039** | *Rhizoma Polygoni Cuspidati* | resveratrol | P05412 | JUN |
| **3040** | *Rhizoma Polygoni Cuspidati* | resveratrol | P38936 | CDKN1A |
| **3041** | *Rhizoma Polygoni Cuspidati* | resveratrol | P24385 | CCND1 |
| **3042** | *Rhizoma Polygoni Cuspidati* | resveratrol | P42574 | CASP3 |
| **3043** | *Rhizoma Polygoni Cuspidati* | resveratrol | P10415 | BCL2 |
| **3044** | *Rhizoma Polygoni Cuspidati* | resveratrol | Q07817 | BCL2L1 |
| **3045** | *Rhizoma Polygoni Cuspidati* | resveratrol | P55211 | CASP9 |
| **3046** | *Rhizoma Polygoni Cuspidati* | resveratrol | Q07812 | BAX |
| **3047** | *Rhizoma Polygoni Cuspidati* | resveratrol | P06493 | CDK1 |
| **3048** | *Rhizoma Polygoni Cuspidati* | resveratrol | P24941 | CDK2 |
| **3049** | *Rhizoma Polygoni Cuspidati* | resveratrol | P28482 | MAPK1 |
| **3050** | *Rhizoma Polygoni Cuspidati* | resveratrol | P45983 | MAPK8 |
| **3051** | *Rhizoma Polygoni Cuspidati* | resveratrol | P12931 | SRC |
| **3052** | *Rhizoma Polygoni Cuspidati* | resveratrol | Q04206 | RELA |
| **3053** | *Rhizoma Polygoni Cuspidati* | resveratrol | P14780 | MMP9 |
| **3054** | *Rhizoma Polygoni Cuspidati* | resveratrol | P17252 | PRKCA |
| **3055** | *Rhizoma Polygoni Cuspidati* | resveratrol | Q9UNQ0 | ABCG2 |
| **3056** | *Rhizoma Polygoni Cuspidati* | resveratrol | P00750 | PLAT |
| **3057** | *Rhizoma Polygoni Cuspidati* | resveratrol | P00749 | PLAU |
| **3058** | *Rhizoma Polygoni Cuspidati* | resveratrol | P60484 | PTEN |
| **3059** | *Rhizoma Polygoni Cuspidati* | resveratrol | P25963 | NFKBIA |
| **3060** | *Rhizoma Polygoni Cuspidati* | resveratrol | P29474 | NOS3 |
| **3061** | *Rhizoma Polygoni Cuspidati* | resveratrol | P35228 | NOS2 |
| **3062** | *Rhizoma Polygoni Cuspidati* | resveratrol | P01375 | TNF |
| **3063** | *Rhizoma Polygoni Cuspidati* | resveratrol | Q07869 | PPARA |
| **3064** | *Rhizoma Polygoni Cuspidati* | resveratrol | P37231 | PPARG |
| **3065** | *Rhizoma Polygoni Cuspidati* | resveratrol | P27169 | PON1 |
| **3066** | *Rhizoma Polygoni Cuspidati* | resveratrol | P05231 | IL6 |
| **3067** | *Rhizoma Polygoni Cuspidati* | resveratrol | P10145 | CXCL8 |
| **3068** | *Rhizoma Polygoni Cuspidati* | resveratrol | P31749 | AKT1 |
| **3069** | *Rhizoma Polygoni Cuspidati* | resveratrol | P40763 | STAT3 |
| **3070** | *Rhizoma Polygoni Cuspidati* | resveratrol | Q16665 | HIF1A |
| **3071** | *Rhizoma Polygoni Cuspidati* | resveratrol | P08183 | ABCB1 |
| **3072** | *Rhizoma Polygoni Cuspidati* | resveratrol | P36956 | SREBF1 |
| **3073** | *Rhizoma Polygoni Cuspidati* | resveratrol | P05305 | EDN1 |
| **3074** | *Rhizoma Polygoni Cuspidati* | resveratrol | P00441 | SOD1 |
| **3075** | *Rhizoma Polygoni Cuspidati* | resveratrol | P15559 | NQO1 |
| **3076** | *Rhizoma Polygoni Cuspidati* | resveratrol | P04040 | CAT |
| **3077** | *Rhizoma Polygoni Cuspidati* | resveratrol | P35568 | IRS1 |
| **3078** | *Rhizoma Polygoni Cuspidati* | resveratrol | P14672 | SLC2A4 |
| **3079** | *Rhizoma Polygoni Cuspidati* | resveratrol | P01584 | IL1B |
| **3080** | *Rhizoma Polygoni Cuspidati* | resveratrol | P35222 | CTNNB1 |
| **3081** | *Rhizoma Polygoni Cuspidati* | resveratrol | P08253 | MMP2 |
| **3082** | *Rhizoma Polygoni Cuspidati* | resveratrol | P13500 | CCL2 |
| **3083** | *Rhizoma Polygoni Cuspidati* | resveratrol | P21730 | C5AR1 |
| **3084** | *Rhizoma Polygoni Cuspidati* | resveratrol | Q16236 | NFE2L2 |
| **3085** | *Rhizoma Polygoni Cuspidati* | resveratrol | P01137 | TGFB1 |
| **3086** | *Rhizoma Polygoni Cuspidati* | resveratrol | P42345 | MTOR |
| **3087** | *Rhizoma Polygoni Cuspidati* | resveratrol | P10275 | AR |
| **3088** | *Rhizoma Polygoni Cuspidati* | resveratrol | Q07820 | MCL1 |
| **3089** | *Rhizoma Polygoni Cuspidati* | resveratrol | O14763 | TNFRSF10B |
| **3090** | *Rhizoma Polygoni Cuspidati* | resveratrol | P27361 | MAPK3 |
| **3091** | *Rhizoma Polygoni Cuspidati* | resveratrol | Q16548 | BCL2A1 |
| **3092** | *Rhizoma Polygoni Cuspidati* | resveratrol | P01106 | MYC |
| **3093** | *Rhizoma Polygoni Cuspidati* | resveratrol | P15104 | GLUL |
| **3094** | *Rhizoma Polygoni Cuspidati* | resveratrol | P05362 | ICAM1 |
| **3095** | *Rhizoma Polygoni Cuspidati* | resveratrol | P23219 | PTGS1 |
| **3096** | *Rhizoma Polygoni Cuspidati* | resveratrol | P08069 | IGF1R |
| **3097** | *Rhizoma Polygoni Cuspidati* | resveratrol | P13569 | CFTR |
| **3098** | *Rhizoma Polygoni Cuspidati* | resveratrol | P01730 | CD4 |
| **3099** | *Rhizoma Polygoni Cuspidati* | resveratrol | P09601 | HMOX1 |
| **3100** | *Rhizoma Polygoni Cuspidati* | resveratrol | P30519 | HMOX2 |
| **3101** | *Rhizoma Polygoni Cuspidati* | resveratrol | P00390 | GSR |
| **3102** | *Rhizoma Polygoni Cuspidati* | resveratrol | P78329 | CYP4F2 |
| **3103** | *Rhizoma Polygoni Cuspidati* | resveratrol | P43490 | NAMPT |
| **3104** | *Rhizoma Polygoni Cuspidati* | resveratrol | P05121 | SERPINE1 |
| **3105** | *Rhizoma Polygoni Cuspidati* | resveratrol | P01031 | C5 |
| **3106** | *Rhizoma Polygoni Cuspidati* | resveratrol | Q16539 | MAPK14 |
| **3107** | *Rhizoma Polygoni Cuspidati* | resveratrol | P04114 | APOB |
| **3108** | *Rhizoma Polygoni Cuspidati* | resveratrol | P18054 | ALOX12 |
| **3109** | *Rhizoma Polygoni Cuspidati* | resveratrol | P05181 | CYP2E1 |
| **3110** | *Rhizoma Polygoni Cuspidati* | resveratrol | P01116 | KRAS |
| **3111** | *Rhizoma Polygoni Cuspidati* | resveratrol | O60603 | TLR2 |
| **3112** | *Rhizoma Polygoni Cuspidati* | resveratrol | P11712 | CYP2C9 |
| **3113** | *Rhizoma Polygoni Cuspidati* | resveratrol | P14679 | TYR |
| **3114** | *Rhizoma Polygoni Cuspidati* | resveratrol | P42336 | PIK3CA |
| **3115** | *Rhizoma Polygoni Cuspidati* | resveratrol | Q9UBK8 | MTRR |
| **3116** | *Rhizoma Polygoni Cuspidati* | resveratrol | P01189 | POMC |
| **3117** | *Rhizoma Polygoni Cuspidati* | resveratrol | P31040 | SDHA |
| **3118** | *Rhizoma Polygoni Cuspidati* | resveratrol | P01133 | EGF |
| **3119** | *Rhizoma Polygoni Cuspidati* | resveratrol | P06400 | RB1 |
| **3120** | *Rhizoma Polygoni Cuspidati* | resveratrol | P04626 | ERBB2 |
| **3121** | *Rhizoma Polygoni Cuspidati* | resveratrol | Q01959 | SLC6A3 |
| **3122** | *Rhizoma Polygoni Cuspidati* | resveratrol | P00533 | EGFR |
| **3123** | *Rhizoma Polygoni Cuspidati* | resveratrol | Q13315 | ATM |
| **3124** | *Rhizoma Polygoni Cuspidati* | resveratrol | O60235 | TMPRSS11D |
| **3125** | *Rhizoma Polygoni Cuspidati* | resveratrol | P05067 | APP |
| **3126** | *Rhizoma Polygoni Cuspidati* | resveratrol | P04035 | HMGCR |
| **3127** | *Rhizoma Polygoni Cuspidati* | resveratrol | P00352 | ALDH1A1 |
| **3128** | *Rhizoma Polygoni Cuspidati* | resveratrol | P34932 | HSPA4 |
| **3129** | *Rhizoma Polygoni Cuspidati* | resveratrol | Q8N4T8 | CBR4 |
| **3130** | *Rhizoma Polygoni Cuspidati* | resveratrol | P22309 | UGT1A1 |
| **3131** | *Rhizoma Polygoni Cuspidati* | resveratrol | P02778 | CXCL10 |
| **3132** | *Rhizoma Polygoni Cuspidati* | resveratrol | P35558 | PCK1 |
| **3133** | *Rhizoma Polygoni Cuspidati* | resveratrol | Q15848 | ADIPOQ |
| **3134** | *Rhizoma Polygoni Cuspidati* | resveratrol | P50225 | SULT1A1 |
| **3135** | *Rhizoma Polygoni Cuspidati* | resveratrol | P49841 | GSK3B |
| **3136** | *Rhizoma Polygoni Cuspidati* | resveratrol | O75469 | NR1I2 |
| **3137** | *Rhizoma Polygoni Cuspidati* | resveratrol | P08684 | CYP3A4 |
| **3138** | *Rhizoma Polygoni Cuspidati* | resveratrol | Q05397 | PTK2 |
| **3139** | *Rhizoma Polygoni Cuspidati* | resveratrol | P05177 | CYP1A2 |
| **3140** | *Rhizoma Polygoni Cuspidati* | resveratrol | P28290 | CS |
| **3141** | *Rhizoma Polygoni Cuspidati* | resveratrol | Q9HAW8 | UGT1A10 |
| **3142** | *Rhizoma Polygoni Cuspidati* | resveratrol | O60656 | UGT1A9 |
| **3143** | *Rhizoma Polygoni Cuspidati* | resveratrol | Q14790 | CASP8 |
| **3144** | *Rhizoma Polygoni Cuspidati* | resveratrol | P27540 | ARNT |
| **3145** | *Rhizoma Polygoni Cuspidati* | resveratrol | P10635 | CYP2D6 |
| **3146** | *Rhizoma Polygoni Cuspidati* | resveratrol | P01019 | AGT |
| **3147** | *Rhizoma Polygoni Cuspidati* | resveratrol | P09874 | PARP1 |
| **3148** | *Rhizoma Polygoni Cuspidati* | resveratrol | Q14994 | NR1I3 |
| **3149** | *Rhizoma Polygoni Cuspidati* | resveratrol | P55210 | CASP7 |
| **3150** | *Rhizoma Polygoni Cuspidati* | resveratrol | P33261 | CYP2C19 |
| **3151** | *Rhizoma Polygoni Cuspidati* | resveratrol | Q9HAW7 | UGT1A7 |
| **3152** | *Rhizoma Polygoni Cuspidati* | resveratrol | Q9HAW9 | UGT1A8 |
| **3153** | *Rhizoma Polygoni Cuspidati* | resveratrol | O00206 | TLR4 |
| **3154** | *Rhizoma Polygoni Cuspidati* | resveratrol | O60674 | JAK2 |
| **3155** | *Rhizoma Polygoni Cuspidati* | resveratrol | P09341 | CXCL1 |
| **3156** | *Rhizoma Polygoni Cuspidati* | resveratrol | P33527 | ABCC1 |
| **3157** | *Rhizoma Polygoni Cuspidati* | resveratrol | P21731 | TBXA2R |
| **3158** | *Rhizoma Polygoni Cuspidati* | resveratrol | P07203 | GPX1 |
| **3159** | *Rhizoma Polygoni Cuspidati* | resveratrol | P11388 | TOP2A |
| **3160** | *Rhizoma Polygoni Cuspidati* | resveratrol | P23560 | BDNF |
| **3161** | *Rhizoma Polygoni Cuspidati* | resveratrol | P16435 | POR |
| **3162** | *Rhizoma Polygoni Cuspidati* | rhein | Q07869 | PPARA |
| **3163** | *Rhizoma Polygoni Cuspidati* | rhein | P37231 | PPARG |
| **3164** | *Rhizoma Polygoni Cuspidati* | rhein | Q04206 | RELA |
| **3165** | *Rhizoma Polygoni Cuspidati* | Tar | P04040 | CAT |
| **3166** | *Rhizoma Polygoni Cuspidati* | Tar | P23946 | CMA1 |
| **3167** | *Rhizoma Polygoni Cuspidati* | Tar | Q7L5Y1 | ENOSF1 |
| **3168** | *Rhizoma Polygoni Cuspidati* | Tar | O43175 | PHGDH |
| **3169** | *Rhizoma Polygoni Cuspidati* | Tar | P17174 | GOT1 |
| **3170** | *Rhizoma Polygoni Cuspidati* | Tar | P68106 | FKBP1B |
| **3171** | *Herba Gynostemmatis* | (3S,5R,8R,9R,10R,12R,13R,14R,17S)-17-[(2S)-2-hydroxy-6-methylhept-5-en-2-yl]-4,4,8,10,14-pentamethyl-2,3,5,6,7,9,11,12,13,15,16,17-dodecahydro-1H-cyclopenta[a]phenanthrene-3,12-diol | P06400 | RB1 |
| **3172** | *Herba Gynostemmatis* | 3'-methyleriodictyol | P04141 | CSF2 |
| **3173** | *Herba Gynostemmatis* | 3'-methyleriodictyol | P04798 | CYP1A1 |
| **3174** | *Herba Gynostemmatis* | 3'-methyleriodictyol | P00533 | EGFR |
| **3175** | *Herba Gynostemmatis* | 3'-methyleriodictyol | P35869 | AHR |
| **3176** | *Herba Gynostemmatis* | 3'-methyleriodictyol | P02768 | ALB |
| **3177** | *Herba Gynostemmatis* | 3'-methyleriodictyol | P09917 | ALOX5 |
| **3178** | *Herba Gynostemmatis* | 3'-methyleriodictyol | P00390 | GSR |
| **3179** | *Herba Gynostemmatis* | 3'-methyleriodictyol | P09211 | GSTP1 |
| **3180** | *Herba Gynostemmatis* | 3'-methyleriodictyol | P04035 | HMGCR |
| **3181** | *Herba Gynostemmatis* | 3'-methyleriodictyol | P08069 | IGF1R |
| **3182** | *Herba Gynostemmatis* | 3'-methyleriodictyol | P01584 | IL1B |
| **3183** | *Herba Gynostemmatis* | 3'-methyleriodictyol | P05231 | IL6 |
| **3184** | *Herba Gynostemmatis* | 3'-methyleriodictyol | P10145 | CXCL8 |
| **3185** | *Herba Gynostemmatis* | 3'-methyleriodictyol | P02778 | CXCL10 |
| **3186** | *Herba Gynostemmatis* | 3'-methyleriodictyol | P35568 | IRS1 |
| **3187** | *Herba Gynostemmatis* | 3'-methyleriodictyol | P05412 | JUN |
| **3188** | *Herba Gynostemmatis* | 3'-methyleriodictyol | P27540 | ARNT |
| **3189** | *Herba Gynostemmatis* | 3'-methyleriodictyol | P14780 | MMP9 |
| **3190** | *Herba Gynostemmatis* | 3'-methyleriodictyol | P05164 | MPO |
| **3191** | *Herba Gynostemmatis* | 3'-methyleriodictyol | P53396 | ACLY |
| **3192** | *Herba Gynostemmatis* | 3'-methyleriodictyol | P25963 | NFKBIA |
| **3193** | *Herba Gynostemmatis* | 3'-methyleriodictyol | P35228 | NOS2 |
| **3194** | *Herba Gynostemmatis* | 3'-methyleriodictyol | P16435 | POR |
| **3195** | *Herba Gynostemmatis* | 3'-methyleriodictyol | P28482 | MAPK1 |
| **3196** | *Herba Gynostemmatis* | 3'-methyleriodictyol | P27361 | MAPK3 |
| **3197** | *Herba Gynostemmatis* | 3'-methyleriodictyol | Q07812 | BAX |
| **3198** | *Herba Gynostemmatis* | 3'-methyleriodictyol | P10415 | BCL2 |
| **3199** | *Herba Gynostemmatis* | 3'-methyleriodictyol | Q04206 | RELA |
| **3200** | *Herba Gynostemmatis* | 3'-methyleriodictyol | P13500 | CCL2 |
| **3201** | *Herba Gynostemmatis* | 3'-methyleriodictyol | P00441 | SOD1 |
| **3202** | *Herba Gynostemmatis* | 3'-methyleriodictyol | P21731 | TBXA2R |
| **3203** | *Herba Gynostemmatis* | 3'-methyleriodictyol | P01375 | TNF |
| **3204** | *Herba Gynostemmatis* | 3'-methyleriodictyol | P42574 | CASP3 |
| **3205** | *Herba Gynostemmatis* | 3'-methyleriodictyol | P55210 | CASP7 |
| **3206** | *Herba Gynostemmatis* | 3'-methyleriodictyol | P04040 | CAT |
| **3207** | *Herba Gynostemmatis* | 6-o-malonyl-beta-methyl-d-glucopyranoside | P42574 | CASP3 |
| **3208** | *Herba Gynostemmatis* | beta-sitosterol | P05412 | JUN |
| **3209** | *Herba Gynostemmatis* | beta-sitosterol | P27169 | PON1 |
| **3210** | *Herba Gynostemmatis* | beta-sitosterol | P17252 | PRKCA |
| **3211** | *Herba Gynostemmatis* | beta-sitosterol | P10415 | BCL2 |
| **3212** | *Herba Gynostemmatis* | beta-sitosterol | P01137 | TGFB1 |
| **3213** | *Herba Gynostemmatis* | beta-sitosterol | P42574 | CASP3 |
| **3214** | *Herba Gynostemmatis* | beta-sitosterol | P02649 | APOE |
| **3215** | *Herba Gynostemmatis* | beta-sitosterol | P05362 | ICAM1 |
| **3216** | *Herba Gynostemmatis* | beta-sitosterol | Q9UHC9 | NPC1L1 |
| **3217** | *Herba Gynostemmatis* | beta-sitosterol | P36956 | SREBF1 |
| **3218** | *Herba Gynostemmatis* | beta-sitosterol | O95477 | ABCA1 |
| **3219** | *Herba Gynostemmatis* | caprylic acid | P10145 | CXCL8 |
| **3220** | *Herba Gynostemmatis* | caprylic acid | Q07869 | PPARA |
| **3221** | *Herba Gynostemmatis* | caprylic acid | P51648 | ALDH3A2 |
| **3222** | *Herba Gynostemmatis* | caprylic acid | P49189 | ALDH9A1 |
| **3223** | *Herba Gynostemmatis* | caprylic acid | P30837 | ALDH1B1 |
| **3224** | *Herba Gynostemmatis* | caprylic acid | P01185 | AVP |
| **3225** | *Herba Gynostemmatis* | CLR | P01730 | CD4 |
| **3226** | *Herba Gynostemmatis* | CLR | P03372 | ESR1 |
| **3227** | *Herba Gynostemmatis* | CLR | P28482 | MAPK1 |
| **3228** | *Herba Gynostemmatis* | CLR | P30519 | HMOX2 |
| **3229** | *Herba Gynostemmatis* | CLR | Q16698 | DECR1 |
| **3230** | *Herba Gynostemmatis* | CLR | P00390 | GSR |
| **3231** | *Herba Gynostemmatis* | CLR | P27169 | PON1 |
| **3232** | *Herba Gynostemmatis* | CLR | P05121 | SERPINE1 |
| **3233** | *Herba Gynostemmatis* | CLR | P05164 | MPO |
| **3234** | *Herba Gynostemmatis* | CLR | P13500 | CCL2 |
| **3235** | *Herba Gynostemmatis* | CLR | Q16539 | MAPK14 |
| **3236** | *Herba Gynostemmatis* | CLR | P04114 | APOB |
| **3237** | *Herba Gynostemmatis* | CLR | Q9UNQ0 | ABCG2 |
| **3238** | *Herba Gynostemmatis* | CLR | P04040 | CAT |
| **3239** | *Herba Gynostemmatis* | CLR | P00505 | GOT2 |
| **3240** | *Herba Gynostemmatis* | CLR | P02649 | APOE |
| **3241** | *Herba Gynostemmatis* | CLR | P05231 | IL6 |
| **3242** | *Herba Gynostemmatis* | CLR | Q8WTV0 | SCARB1 |
| **3243** | *Herba Gynostemmatis* | CLR | Q07869 | PPARA |
| **3244** | *Herba Gynostemmatis* | CLR | P27361 | MAPK3 |
| **3245** | *Herba Gynostemmatis* | CLR | P01584 | IL1B |
| **3246** | *Herba Gynostemmatis* | CLR | P06276 | BCHE |
| **3247** | *Herba Gynostemmatis* | CLR | P01189 | POMC |
| **3248** | *Herba Gynostemmatis* | CLR | P05362 | ICAM1 |
| **3249** | *Herba Gynostemmatis* | CLR | Q9H2J7 | SLC6A15 |
| **3250** | *Herba Gynostemmatis* | CLR | P04626 | ERBB2 |
| **3251** | *Herba Gynostemmatis* | CLR | P31749 | AKT1 |
| **3252** | *Herba Gynostemmatis* | CLR | Q01959 | SLC6A3 |
| **3253** | *Herba Gynostemmatis* | CLR | P00533 | EGFR |
| **3254** | *Herba Gynostemmatis* | CLR | O60235 | TMPRSS11D |
| **3255** | *Herba Gynostemmatis* | CLR | P05067 | APP |
| **3256** | *Herba Gynostemmatis* | CLR | P37231 | PPARG |
| **3257** | *Herba Gynostemmatis* | CLR | P04035 | HMGCR |
| **3258** | *Herba Gynostemmatis* | CLR | Q9UHC9 | NPC1L1 |
| **3259** | *Herba Gynostemmatis* | CLR | P02768 | ALB |
| **3260** | *Herba Gynostemmatis* | CLR | Q99527 | GPER1 |
| **3261** | *Herba Gynostemmatis* | CLR | P29474 | NOS3 |
| **3262** | *Herba Gynostemmatis* | CLR | Q9H2F3 | HSD3B7 |
| **3263** | *Herba Gynostemmatis* | CLR | P35568 | IRS1 |
| **3264** | *Herba Gynostemmatis* | CLR | P01100 | FOS |
| **3265** | *Herba Gynostemmatis* | CLR | P99999 | CYCS |
| **3266** | *Herba Gynostemmatis* | CLR | Q15848 | ADIPOQ |
| **3267** | *Herba Gynostemmatis* | CLR | P14672 | SLC2A4 |
| **3268** | *Herba Gynostemmatis* | CLR | P03956 | MMP1 |
| **3269** | *Herba Gynostemmatis* | CLR | Q96N87 | SLC6A18 |
| **3270** | *Herba Gynostemmatis* | CLR | P07237 | P4HB |
| **3271** | *Herba Gynostemmatis* | CLR | P30536 | TSPO |
| **3272** | *Herba Gynostemmatis* | CLR | O75469 | NR1I2 |
| **3273** | *Herba Gynostemmatis* | CLR | Q92731 | ESR2 |
| **3274** | *Herba Gynostemmatis* | CLR | P36956 | SREBF1 |
| **3275** | *Herba Gynostemmatis* | CLR | P12931 | SRC |
| **3276** | *Herba Gynostemmatis* | CLR | Q14994 | NR1I3 |
| **3277** | *Herba Gynostemmatis* | CLR | P14060 | HSD3B1 |
| **3278** | *Herba Gynostemmatis* | CLR | P26439 | HSD3B2 |
| **3279** | *Herba Gynostemmatis* | CLR | P17174 | GOT1 |
| **3280** | *Herba Gynostemmatis* | CLR | P14780 | MMP9 |
| **3281** | *Herba Gynostemmatis* | CLR | O00206 | TLR4 |
| **3282** | *Herba Gynostemmatis* | CLR | P10275 | AR |
| **3283** | *Herba Gynostemmatis* | CLR | O95477 | ABCA1 |
| **3284** | *Herba Gynostemmatis* | CLR | P01275 | GCG |
| **3285** | *Herba Gynostemmatis* | CLR | P01375 | TNF |
| **3286** | *Herba Gynostemmatis* | CLR | P16435 | POR |
| **3287** | *Herba Gynostemmatis* | CLR | P00739 | HPR |
| **3288** | *Herba Gynostemmatis* | Guasol | P00390 | GSR |
| **3289** | *Herba Gynostemmatis* | Guasol | P04040 | CAT |
| **3290** | *Herba Gynostemmatis* | Gulutamine | P25789 | PSMA4 |
| **3291** | *Herba Gynostemmatis* | Gulutamine | P43686 | PSMC4 |
| **3292** | *Herba Gynostemmatis* | Gulutamine | P21917 | DRD4 |
| **3293** | *Herba Gynostemmatis* | Gulutamine | P09622 | DLD |
| **3294** | *Herba Gynostemmatis* | Gulutamine | Q16401 | PSMD5 |
| **3295** | *Herba Gynostemmatis* | Gulutamine | P48556 | PSMD8 |
| **3296** | *Herba Gynostemmatis* | Gulutamine | P25788 | PSMA3 |
| **3297** | *Herba Gynostemmatis* | Gulutamine | P46663 | BDKRB1 |
| **3298** | *Herba Gynostemmatis* | Gulutamine | Q9UL46 | PSME2 |
| **3299** | *Herba Gynostemmatis* | Gulutamine | O75832 | PSMD10 |
| **3300** | *Herba Gynostemmatis* | Gulutamine | P51665 | PSMD7 |
| **3301** | *Herba Gynostemmatis* | Gulutamine | P00390 | GSR |
| **3302** | *Herba Gynostemmatis* | Gulutamine | Q13155 | AIMP2 |
| **3303** | *Herba Gynostemmatis* | Gulutamine | P60468 | SEC61B |
| **3304** | *Herba Gynostemmatis* | Gulutamine | P01031 | C5 |
| **3305** | *Herba Gynostemmatis* | Gulutamine | P30838 | ALDH3A1 |
| **3306** | *Herba Gynostemmatis* | Gulutamine | Q9UIQ6 | LNPEP |
| **3307** | *Herba Gynostemmatis* | Gulutamine | P54136 | RARS |
| **3308** | *Herba Gynostemmatis* | Gulutamine | P04040 | CAT |
| **3309** | *Herba Gynostemmatis* | Gulutamine | P61619 | SEC61A1 |
| **3310** | *Herba Gynostemmatis* | Gulutamine | P00505 | GOT2 |
| **3311** | *Herba Gynostemmatis* | Gulutamine | P01024 | C3 |
| **3312** | *Herba Gynostemmatis* | Gulutamine | Q99705 | MCHR1 |
| **3313** | *Herba Gynostemmatis* | Gulutamine | O94788 | ALDH1A2 |
| **3314** | *Herba Gynostemmatis* | Gulutamine | P48448 | ALDH3B2 |
| **3315** | *Herba Gynostemmatis* | Gulutamine | Q9GZQ4 | NMUR2 |
| **3316** | *Herba Gynostemmatis* | Gulutamine | P49286 | MTNR1B |
| **3317** | *Herba Gynostemmatis* | Gulutamine | P04083 | ANXA1 |
| **3318** | *Herba Gynostemmatis* | Gulutamine | Q3SY69 | ALDH1L2 |
| **3319** | *Herba Gynostemmatis* | Gulutamine | Q99436 | PSMB7 |
| **3320** | *Herba Gynostemmatis* | Gulutamine | P62191 | PSMC1 |
| **3321** | *Herba Gynostemmatis* | Gulutamine | P60900 | PSMA6 |
| **3322** | *Herba Gynostemmatis* | Gulutamine | O00231 | PSMD11 |
| **3323** | *Herba Gynostemmatis* | Gulutamine | Q13867 | BLMH |
| **3324** | *Herba Gynostemmatis* | Gulutamine | P05091 | ALDH2 |
| **3325** | *Herba Gynostemmatis* | Gulutamine | P56192 | MARS |
| **3326** | *Herba Gynostemmatis* | Gulutamine | P20618 | PSMB1 |
| **3327** | *Herba Gynostemmatis* | Gulutamine | P42336 | PIK3CA |
| **3328** | *Herba Gynostemmatis* | Gulutamine | P14868 | DARS |
| **3329** | *Herba Gynostemmatis* | Gulutamine | P48645 | NMU |
| **3330** | *Herba Gynostemmatis* | Gulutamine | O43242 | PSMD3 |
| **3331** | *Herba Gynostemmatis* | Gulutamine | P01189 | POMC |
| **3332** | *Herba Gynostemmatis* | Gulutamine | P01042 | KNG1 |
| **3333** | *Herba Gynostemmatis* | Gulutamine | Q07075 | ENPEP |
| **3334** | *Herba Gynostemmatis* | Gulutamine | Q9H2A2 | ALDH8A1 |
| **3335** | *Herba Gynostemmatis* | Gulutamine | P28072 | PSMB6 |
| **3336** | *Herba Gynostemmatis* | Gulutamine | P28066 | PSMA5 |
| **3337** | *Herba Gynostemmatis* | Gulutamine | P62979 | RPS27A |
| **3338** | *Herba Gynostemmatis* | Gulutamine | Q13304 | GPR17 |
| **3339** | *Herba Gynostemmatis* | Gulutamine | O75223 | GGCT |
| **3340** | *Herba Gynostemmatis* | Gulutamine | P08913 | ADRA2A |
| **3341** | *Herba Gynostemmatis* | Gulutamine | Q969V1 | MCHR2 |
| **3342** | *Herba Gynostemmatis* | Gulutamine | P05067 | APP |
| **3343** | *Herba Gynostemmatis* | Gulutamine | P15121 | AKR1B1 |
| **3344** | *Herba Gynostemmatis* | Gulutamine | P28070 | PSMB4 |
| **3345** | *Herba Gynostemmatis* | Gulutamine | P30038 | ALDH4A1 |
| **3346** | *Herba Gynostemmatis* | Gulutamine | P35998 | PSMC2 |
| **3347** | *Herba Gynostemmatis* | Gulutamine | Q8IZ83 | ALDH16A1 |
| **3348** | *Herba Gynostemmatis* | Gulutamine | P61289 | PSME3 |
| **3349** | *Herba Gynostemmatis* | Gulutamine | P02768 | ALB |
| **3350** | *Herba Gynostemmatis* | Gulutamine | Q15008 | PSMD6 |
| **3351** | *Herba Gynostemmatis* | Gulutamine | Q9NZ08 | ERAP1 |
| **3352** | *Herba Gynostemmatis* | Gulutamine | O76081 | RGS20 |
| **3353** | *Herba Gynostemmatis* | Gulutamine | Q99527 | GPER1 |
| **3354** | *Herba Gynostemmatis* | Gulutamine | P00352 | ALDH1A1 |
| **3355** | *Herba Gynostemmatis* | Gulutamine | Q9H9S3 | SEC61A2 |
| **3356** | *Herba Gynostemmatis* | Gulutamine | P17980 | PSMC3 |
| **3357** | *Herba Gynostemmatis* | Gulutamine | P30101 | PDIA3 |
| **3358** | *Herba Gynostemmatis* | Gulutamine | P48039 | MTNR1A |
| **3359** | *Herba Gynostemmatis* | Gulutamine | P0CG47 | UBB |
| **3360** | *Herba Gynostemmatis* | Gulutamine | P02778 | CXCL10 |
| **3361** | *Herba Gynostemmatis* | Gulutamine | Q9HB89 | NMUR1 |
| **3362** | *Herba Gynostemmatis* | Gulutamine | P10145 | CXCL8 |
| **3363** | *Herba Gynostemmatis* | Gulutamine | P47897 | QARS |
| **3364** | *Herba Gynostemmatis* | Gulutamine | P15104 | GLUL |
| **3365** | *Herba Gynostemmatis* | Gulutamine | Q99460 | PSMD1 |
| **3366** | *Herba Gynostemmatis* | Gulutamine | Q13200 | PSMD2 |
| **3367** | *Herba Gynostemmatis* | Gulutamine | P62195 | PSMC5 |
| **3368** | *Herba Gynostemmatis* | Gulutamine | P42574 | CASP3 |
| **3369** | *Herba Gynostemmatis* | Gulutamine | Q8TAA3 | PSMA8 |
| **3370** | *Herba Gynostemmatis* | Gulutamine | P51649 | ALDH5A1 |
| **3371** | *Herba Gynostemmatis* | Gulutamine | P34896 | SHMT1 |
| **3372** | *Herba Gynostemmatis* | Gulutamine | P35558 | PCK1 |
| **3373** | *Herba Gynostemmatis* | Gulutamine | P55786 | NPEPPS |
| **3374** | *Herba Gynostemmatis* | Gulutamine | P27797 | CALR |
| **3375** | *Herba Gynostemmatis* | Gulutamine | O15382 | BCAT2 |
| **3376** | *Herba Gynostemmatis* | Gulutamine | Q15046 | KARS |
| **3377** | *Herba Gynostemmatis* | Gulutamine | Q96KP4 | CNDP2 |
| **3378** | *Herba Gynostemmatis* | Gulutamine | Q92530 | PSMF1 |
| **3379** | *Herba Gynostemmatis* | Gulutamine | Q9H1C0 | LPAR5 |
| **3380** | *Herba Gynostemmatis* | Gulutamine | P20382 | PMCH |
| **3381** | *Herba Gynostemmatis* | Gulutamine | P47895 | ALDH1A3 |
| **3382** | *Herba Gynostemmatis* | Gulutamine | P49795 | RGS19 |
| **3383** | *Herba Gynostemmatis* | Gulutamine | P59768 | GNG2 |
| **3384** | *Herba Gynostemmatis* | Gulutamine | P25090 | FPR2 |
| **3385** | *Herba Gynostemmatis* | Gulutamine | P60059 | SEC61G |
| **3386** | *Herba Gynostemmatis* | Gulutamine | P63096 | GNAI1 |
| **3387** | *Herba Gynostemmatis* | Gulutamine | P0CG48 | UBC |
| **3388** | *Herba Gynostemmatis* | Gulutamine | P51648 | ALDH3A2 |
| **3389** | *Herba Gynostemmatis* | Gulutamine | Q7L5Y1 | ENOSF1 |
| **3390** | *Herba Gynostemmatis* | Gulutamine | P49189 | ALDH9A1 |
| **3391** | *Herba Gynostemmatis* | Gulutamine | P21730 | C5AR1 |
| **3392** | *Herba Gynostemmatis* | Gulutamine | P17735 | TAT |
| **3393** | *Herba Gynostemmatis* | Gulutamine | O00232 | PSMD12 |
| **3394** | *Herba Gynostemmatis* | Gulutamine | P0DJI8 | SAA1 |
| **3395** | *Herba Gynostemmatis* | Gulutamine | Q42521 | GAD1 |
| **3396** | *Herba Gynostemmatis* | Gulutamine | P40306 | PSMB10 |
| **3397** | *Herba Gynostemmatis* | Gulutamine | Q92633 | LPAR1 |
| **3398** | *Herba Gynostemmatis* | Gulutamine | O60218 | AKR1B10 |
| **3399** | *Herba Gynostemmatis* | Gulutamine | P17693 | HLA-G |
| **3400** | *Herba Gynostemmatis* | Gulutamine | P25929 | NPY1R |
| **3401** | *Herba Gynostemmatis* | Gulutamine | Q8N5D0 | WDTC1 |
| **3402** | *Herba Gynostemmatis* | Gulutamine | P28074 | PSMB5 |
| **3403** | *Herba Gynostemmatis* | Gulutamine | P49802 | RGS7 |
| **3404** | *Herba Gynostemmatis* | Gulutamine | P01019 | AGT |
| **3405** | *Herba Gynostemmatis* | Gulutamine | P07814 | EPRS |
| **3406** | *Herba Gynostemmatis* | Gulutamine | Q08116 | RGS1 |
| **3407** | *Herba Gynostemmatis* | Gulutamine | Q9NS28 | RGS18 |
| **3408** | *Herba Gynostemmatis* | Gulutamine | P55036 | PSMD4 |
| **3409** | *Herba Gynostemmatis* | Gulutamine | P25774 | CTSS |
| **3410** | *Herba Gynostemmatis* | Gulutamine | O43665 | RGS10 |
| **3411** | *Herba Gynostemmatis* | Gulutamine | O43175 | PHGDH |
| **3412** | *Herba Gynostemmatis* | Gulutamine | P08754 | GNAI3 |
| **3413** | *Herba Gynostemmatis* | Gulutamine | P17174 | GOT1 |
| **3414** | *Herba Gynostemmatis* | Gulutamine | Q9UBY5 | LPAR3 |
| **3415** | *Herba Gynostemmatis* | Gulutamine | O14818 | PSMA7 |
| **3416** | *Herba Gynostemmatis* | Gulutamine | P49721 | PSMB2 |
| **3417** | *Herba Gynostemmatis* | Gulutamine | P09917 | ALOX5 |
| **3418** | *Herba Gynostemmatis* | Gulutamine | P41252 | IARS |
| **3419** | *Herba Gynostemmatis* | Gulutamine | P29144 | TPP2 |
| **3420** | *Herba Gynostemmatis* | Gulutamine | P30499 | HLA-C |
| **3421** | *Herba Gynostemmatis* | Gulutamine | P30443 | HLA-A |
| **3422** | *Herba Gynostemmatis* | Gulutamine | Q5H8A3 | NMS |
| **3423** | *Herba Gynostemmatis* | Gulutamine | P30837 | ALDH1B1 |
| **3424** | *Herba Gynostemmatis* | Gulutamine | P08195 | SLC3A2 |
| **3425** | *Herba Gynostemmatis* | Gulutamine | P05305 | EDN1 |
| **3426** | *Herba Gynostemmatis* | Gulutamine | O43324 | EEF1E1 |
| **3427** | *Herba Gynostemmatis* | Gulutamine | P01185 | AVP |
| **3428** | *Herba Gynostemmatis* | Gulutamine | P42330 | AKR1C3 |
| **3429** | *Herba Gynostemmatis* | Gulutamine | Q06323 | PSME1 |
| **3430** | *Herba Gynostemmatis* | Gulutamine | Q96BT7 | ALKBH8 |
| **3431** | *Herba Gynostemmatis* | Gulutamine | O75891 | ALDH1L1 |
| **3432** | *Herba Gynostemmatis* | Gulutamine | Q9P2J5 | LARS |
| **3433** | *Herba Gynostemmatis* | Gulutamine | Q12904 | AIMP1 |
| **3434** | *Herba Gynostemmatis* | Gulutamine | P09341 | CXCL1 |
| **3435** | *Herba Gynostemmatis* | Gulutamine | P33527 | ABCC1 |
| **3436** | *Herba Gynostemmatis* | Gulutamine | Q14997 | PSME4 |
| **3437** | *Herba Gynostemmatis* | Gulutamine | Q9HBW0 | LPAR2 |
| **3438** | *Herba Gynostemmatis* | Gulutamine | P18825 | ADRA2C |
| **3439** | *Herba Gynostemmatis* | Gulutamine | A5LHX3 | PSMB11 |
| **3440** | *Herba Gynostemmatis* | Gulutamine | O43566 | RGS14 |
| **3441** | *Herba Gynostemmatis* | Gulutamine | O00487 | PSMD14 |
| **3442** | *Herba Gynostemmatis* | Gulutamine | P61073 | CXCR4 |
| **3443** | *Herba Gynostemmatis* | Gulutamine | P49419 | ALDH7A1 |
| **3444** | *Herba Gynostemmatis* | Gulutamine | P01275 | GCG |
| **3445** | *Herba Gynostemmatis* | Gulutamine | Q5TF58 | IFFO2 |
| **3446** | *Herba Gynostemmatis* | Gulutamine | P62987 | UBA52 |
| **3447** | *Herba Gynostemmatis* | Gulutamine | P21731 | TBXA2R |
| **3448** | *Herba Gynostemmatis* | Gulutamine | P35372 | OPRM1 |
| **3449** | *Herba Gynostemmatis* | Gulutamine | Q9UNM6 | PSMD13 |
| **3450** | *Herba Gynostemmatis* | Gulutamine | P49798 | RGS4 |
| **3451** | *Herba Gynostemmatis* | Gulutamine | P01889 | HLA-B |
| **3452** | *Herba Gynostemmatis* | Gulutamine | Q6P179 | ERAP2 |
| **3453** | *Herba Gynostemmatis* | Gulutamine | O43709 | WBSCR22 |
| **3454** | *Herba Gynostemmatis* | Gulutamine | P62333 | PSMC6 |
| **3455** | *Herba Gynostemmatis* | Gulutamine | P15151 | PVR |
| **3456** | *Herba Gynostemmatis* | Gulutamine | P25786 | PSMA1 |
| **3457** | *Herba Gynostemmatis* | Gulutamine | O00233 | PSMD9 |
| **3458** | *Herba Gynostemmatis* | Gulutamine | P54687 | BCAT1 |
| **3459** | *Herba Gynostemmatis* | Gulutamine | P49758 | RGS6 |
| **3460** | *Herba Gynostemmatis* | Gulutamine | P61769 | B2M |
| **3461** | *Herba Gynostemmatis* | Hyacinthin | P30838 | ALDH3A1 |
| **3462** | *Herba Gynostemmatis* | Hyacinthin | P48448 | ALDH3B2 |
| **3463** | *Herba Gynostemmatis* | Hyacinthin | P05091 | ALDH2 |
| **3464** | *Herba Gynostemmatis* | Hyacinthin | P10145 | CXCL8 |
| **3465** | *Herba Gynostemmatis* | Hyacinthin | P47895 | ALDH1A3 |
| **3466** | *Herba Gynostemmatis* | Hyacinthin | P21397 | MAOA |
| **3467** | *Herba Gynostemmatis* | Hyacinthin | P51648 | ALDH3A2 |
| **3468** | *Herba Gynostemmatis* | Hyacinthin | P27338 | MAOB |
| **3469** | *Herba Gynostemmatis* | Hyacinthin | P01375 | TNF |
| **3470** | *Herba Gynostemmatis* | mli | P07203 | GPX1 |
| **3471** | *Herba Gynostemmatis* | mli | P49841 | GSK3B |
| **3472** | *Herba Gynostemmatis* | mli | Q07812 | BAX |
| **3473** | *Herba Gynostemmatis* | mli | P21912 | SDHB |
| **3474** | *Herba Gynostemmatis* | mli | P00441 | SOD1 |
| **3475** | *Herba Gynostemmatis* | mli | P12931 | SRC |
| **3476** | *Herba Gynostemmatis* | mli | P01730 | CD4 |
| **3477** | *Herba Gynostemmatis* | mli | P49419 | ALDH7A1 |
| **3478** | *Herba Gynostemmatis* | mli | Q16665 | HIF1A |
| **3479** | *Herba Gynostemmatis* | mli | P48357 | LEPR |
| **3480** | *Herba Gynostemmatis* | mli | P10415 | BCL2 |
| **3481** | *Herba Gynostemmatis* | mli | P42574 | CASP3 |
| **3482** | *Herba Gynostemmatis* | mli | P55211 | CASP9 |
| **3483** | *Herba Gynostemmatis* | o-thymol | P34932 | HSPA4 |
| **3484** | *Herba Gynostemmatis* | o-thymol | P42574 | CASP3 |
| **3485** | *Herba Gynostemmatis* | o-thymol | P54855 | UGT2B15 |
| **3486** | *Herba Gynostemmatis* | o-thymol | Q07869 | PPARA |
| **3487** | *Herba Gynostemmatis* | o-xylene | P04798 | CYP1A1 |
| **3488** | *Herba Gynostemmatis* | o-xylene | P05181 | CYP2E1 |
| **3489** | *Herba Gynostemmatis* | o-xylene | P22303 | ACHE |
| **3490** | *Herba Gynostemmatis* | quercetin | P35228 | NOS2 |
| **3491** | *Herba Gynostemmatis* | quercetin | P23219 | PTGS1 |
| **3492** | *Herba Gynostemmatis* | quercetin | P03372 | ESR1 |
| **3493** | *Herba Gynostemmatis* | quercetin | P10275 | AR |
| **3494** | *Herba Gynostemmatis* | quercetin | P10415 | BCL2 |
| **3495** | *Herba Gynostemmatis* | quercetin | P09917 | ALOX5 |
| **3496** | *Herba Gynostemmatis* | quercetin | P08253 | MMP2 |
| **3497** | *Herba Gynostemmatis* | quercetin | P01375 | TNF |
| **3498** | *Herba Gynostemmatis* | quercetin | P00533 | EGFR |
| **3499** | *Herba Gynostemmatis* | quercetin | Q92731 | ESR2 |
| **3500** | *Herba Gynostemmatis* | quercetin | P00749 | PLAU |
| **3501** | *Herba Gynostemmatis* | quercetin | P05231 | IL6 |
| **3502** | *Herba Gynostemmatis* | quercetin | P03956 | MMP1 |
| **3503** | *Herba Gynostemmatis* | quercetin | P28482 | MAPK1 |
| **3504** | *Herba Gynostemmatis* | quercetin | P27169 | PON1 |
| **3505** | *Herba Gynostemmatis* | quercetin | P05412 | JUN |
| **3506** | *Herba Gynostemmatis* | quercetin | P13500 | CCL2 |
| **3507** | *Herba Gynostemmatis* | quercetin | P01584 | IL1B |
| **3508** | *Herba Gynostemmatis* | quercetin | P49841 | GSK3B |
| **3509** | *Herba Gynostemmatis* | quercetin | P05164 | MPO |
| **3510** | *Herba Gynostemmatis* | quercetin | P00750 | PLAT |
| **3511** | *Herba Gynostemmatis* | quercetin | P15559 | NQO1 |
| **3512** | *Herba Gynostemmatis* | quercetin | P47989 | XDH |
| **3513** | *Herba Gynostemmatis* | quercetin | P00441 | SOD1 |
| **3514** | *Herba Gynostemmatis* | quercetin | P08684 | CYP3A4 |
| **3515** | *Herba Gynostemmatis* | quercetin | P04637 | TP53 |
| **3516** | *Herba Gynostemmatis* | quercetin | P05177 | CYP1A2 |
| **3517** | *Herba Gynostemmatis* | quercetin | P09211 | GSTP1 |
| **3518** | *Herba Gynostemmatis* | quercetin | P01133 | EGF |
| **3519** | *Herba Gynostemmatis* | quercetin | P16435 | POR |
| **3520** | *Herba Gynostemmatis* | quercetin | P11387 | TOP1 |
| **3521** | *Herba Gynostemmatis* | quercetin | P09601 | HMOX1 |
| **3522** | *Herba Gynostemmatis* | quercetin | P06400 | RB1 |
| **3523** | *Herba Gynostemmatis* | quercetin | P35869 | AHR |
| **3524** | *Herba Gynostemmatis* | quercetin | Q9UNQ0 | ABCG2 |
| **3525** | *Herba Gynostemmatis* | quercetin | Q07817 | BCL2L1 |
| **3526** | *Herba Gynostemmatis* | quercetin | P42574 | CASP3 |
| **3527** | *Herba Gynostemmatis* | quercetin | P02778 | CXCL10 |
| **3528** | *Herba Gynostemmatis* | quercetin | P04798 | CYP1A1 |
| **3529** | *Herba Gynostemmatis* | quercetin | P11388 | TOP2A |
| **3530** | *Herba Gynostemmatis* | quercetin | P24385 | CCND1 |
| **3531** | *Herba Gynostemmatis* | quercetin | Q16665 | HIF1A |
| **3532** | *Herba Gynostemmatis* | quercetin | O15111 | CHUK |
| **3533** | *Herba Gynostemmatis* | quercetin | P05362 | ICAM1 |
| **3534** | *Herba Gynostemmatis* | quercetin | P10145 | CXCL8 |
| **3535** | *Herba Gynostemmatis* | quercetin | P14780 | MMP9 |
| **3536** | *Herba Gynostemmatis* | quercetin | P01106 | MYC |
| **3537** | *Herba Gynostemmatis* | quercetin | P25963 | NFKBIA |
| **3538** | *Herba Gynostemmatis* | quercetin | P29474 | NOS3 |
| **3539** | *Herba Gynostemmatis* | quercetin | O75469 | NR1I2 |
| **3540** | *Herba Gynostemmatis* | quercetin | Q14994 | NR1I3 |
| **3541** | *Herba Gynostemmatis* | quercetin | Q07869 | PPARA |
| **3542** | *Herba Gynostemmatis* | quercetin | P37231 | PPARG |
| **3543** | *Herba Gynostemmatis* | quercetin | P05121 | SERPINE1 |
| **3544** | *Herba Gynostemmatis* | quercetin | P09874 | PARP1 |
| **3545** | *Herba Gynostemmatis* | quercetin | P17252 | PRKCA |
| **3546** | *Herba Gynostemmatis* | quercetin | P01100 | FOS |
| **3547** | *Herba Gynostemmatis* | quercetin | P55786 | NPEPPS |
| **3548** | *Herba Gynostemmatis* | quercetin | P31749 | AKT1 |
| **3549** | *Herba Gynostemmatis* | quercetin | P04626 | ERBB2 |
| **3550** | *Herba Gynostemmatis* | quercetin | P01137 | TGFB1 |
| **3551** | *Herba Gynostemmatis* | quercetin | P13569 | CFTR |
| **3552** | *Herba Gynostemmatis* | quercetin | P21917 | DRD4 |
| **3553** | *Herba Gynostemmatis* | quercetin | Q96EB6 | SIRT1 |
| **3554** | *Herba Gynostemmatis* | quercetin | P05546 | SERPIND1 |
| **3555** | *Herba Gynostemmatis* | quercetin | P00390 | GSR |
| **3556** | *Herba Gynostemmatis* | quercetin | P78329 | CYP4F2 |
| **3557** | *Herba Gynostemmatis* | quercetin | P43490 | NAMPT |
| **3558** | *Herba Gynostemmatis* | quercetin | Q16539 | MAPK14 |
| **3559** | *Herba Gynostemmatis* | quercetin | P04114 | APOB |
| **3560** | *Herba Gynostemmatis* | quercetin | P04040 | CAT |
| **3561** | *Herba Gynostemmatis* | quercetin | P38936 | CDKN1A |
| **3562** | *Herba Gynostemmatis* | quercetin | P18054 | ALOX12 |
| **3563** | *Herba Gynostemmatis* | quercetin | P05181 | CYP2E1 |
| **3564** | *Herba Gynostemmatis* | quercetin | P01116 | KRAS |
| **3565** | *Herba Gynostemmatis* | quercetin | P21589 | NT5E |
| **3566** | *Herba Gynostemmatis* | quercetin | O60603 | TLR2 |
| **3567** | *Herba Gynostemmatis* | quercetin | P11712 | CYP2C9 |
| **3568** | *Herba Gynostemmatis* | quercetin | P27361 | MAPK3 |
| **3569** | *Herba Gynostemmatis* | quercetin | P14679 | TYR |
| **3570** | *Herba Gynostemmatis* | quercetin | P42336 | PIK3CA |
| **3571** | *Herba Gynostemmatis* | quercetin | P06276 | BCHE |
| **3572** | *Herba Gynostemmatis* | quercetin | P36537 | UGT2B10 |
| **3573** | *Herba Gynostemmatis* | quercetin | P08183 | ABCB1 |
| **3574** | *Herba Gynostemmatis* | quercetin | Q14534 | SQLE |
| **3575** | *Herba Gynostemmatis* | quercetin | P24941 | CDK2 |
| **3576** | *Herba Gynostemmatis* | quercetin | Q16548 | BCL2A1 |
| **3577** | *Herba Gynostemmatis* | quercetin | P08069 | IGF1R |
| **3578** | *Herba Gynostemmatis* | quercetin | O14763 | TNFRSF10B |
| **3579** | *Herba Gynostemmatis* | quercetin | O60235 | TMPRSS11D |
| **3580** | *Herba Gynostemmatis* | quercetin | P15121 | AKR1B1 |
| **3581** | *Herba Gynostemmatis* | quercetin | Q9UHC9 | NPC1L1 |
| **3582** | *Herba Gynostemmatis* | quercetin | Q07812 | BAX |
| **3583** | *Herba Gynostemmatis* | quercetin | O60760 | HPGDS |
| **3584** | *Herba Gynostemmatis* | quercetin | P04141 | CSF2 |
| **3585** | *Herba Gynostemmatis* | quercetin | Q9H2F3 | HSD3B7 |
| **3586** | *Herba Gynostemmatis* | quercetin | P34932 | HSPA4 |
| **3587** | *Herba Gynostemmatis* | quercetin | P19224 | UGT1A6 |
| **3588** | *Herba Gynostemmatis* | quercetin | Q8N4T8 | CBR4 |
| **3589** | *Herba Gynostemmatis* | quercetin | P22309 | UGT1A1 |
| **3590** | *Herba Gynostemmatis* | quercetin | P35568 | IRS1 |
| **3591** | *Herba Gynostemmatis* | quercetin | P99999 | CYCS |
| **3592** | *Herba Gynostemmatis* | quercetin | Q9BZR8 | BCL2L14 |
| **3593** | *Herba Gynostemmatis* | quercetin | Q9UMX3 | BOK |
| **3594** | *Herba Gynostemmatis* | quercetin | O75795 | UGT2B17 |
| **3595** | *Herba Gynostemmatis* | quercetin | Q15848 | ADIPOQ |
| **3596** | *Herba Gynostemmatis* | quercetin | P14672 | SLC2A4 |
| **3597** | *Herba Gynostemmatis* | quercetin | P50225 | SULT1A1 |
| **3598** | *Herba Gynostemmatis* | quercetin | P55211 | CASP9 |
| **3599** | *Herba Gynostemmatis* | quercetin | P48357 | LEPR |
| **3600** | *Herba Gynostemmatis* | quercetin | P21397 | MAOA |
| **3601** | *Herba Gynostemmatis* | quercetin | P54855 | UGT2B15 |
| **3602** | *Herba Gynostemmatis* | quercetin | Q05397 | PTK2 |
| **3603** | *Herba Gynostemmatis* | quercetin | Q9HAW8 | UGT1A10 |
| **3604** | *Herba Gynostemmatis* | quercetin | P35222 | CTNNB1 |
| **3605** | *Herba Gynostemmatis* | quercetin | O60656 | UGT1A9 |
| **3606** | *Herba Gynostemmatis* | quercetin | P12931 | SRC |
| **3607** | *Herba Gynostemmatis* | quercetin | Q14790 | CASP8 |
| **3608** | *Herba Gynostemmatis* | quercetin | P27540 | ARNT |
| **3609** | *Herba Gynostemmatis* | quercetin | P45983 | MAPK8 |
| **3610** | *Herba Gynostemmatis* | quercetin | P10635 | CYP2D6 |
| **3611** | *Herba Gynostemmatis* | quercetin | P42345 | MTOR |
| **3612** | *Herba Gynostemmatis* | quercetin | P25929 | NPY1R |
| **3613** | *Herba Gynostemmatis* | quercetin | Q07820 | MCL1 |
| **3614** | *Herba Gynostemmatis* | quercetin | P55210 | CASP7 |
| **3615** | *Herba Gynostemmatis* | quercetin | P14060 | HSD3B1 |
| **3616** | *Herba Gynostemmatis* | quercetin | P26439 | HSD3B2 |
| **3617** | *Herba Gynostemmatis* | quercetin | Q9HAW7 | UGT1A7 |
| **3618** | *Herba Gynostemmatis* | quercetin | Q9HAW9 | UGT1A8 |
| **3619** | *Herba Gynostemmatis* | quercetin | O00206 | TLR4 |
| **3620** | *Herba Gynostemmatis* | quercetin | O95477 | ABCA1 |
| **3621** | *Herba Gynostemmatis* | quercetin | P27338 | MAOB |
| **3622** | *Herba Gynostemmatis* | quercetin | P05305 | EDN1 |
| **3623** | *Herba Gynostemmatis* | quercetin | P42330 | AKR1C3 |
| **3624** | *Herba Gynostemmatis* | quercetin | O60674 | JAK2 |
| **3625** | *Herba Gynostemmatis* | quercetin | P06493 | CDK1 |
| **3626** | *Herba Gynostemmatis* | quercetin | Q16236 | NFE2L2 |
| **3627** | *Herba Gynostemmatis* | quercetin | P33527 | ABCC1 |
| **3628** | *Herba Gynostemmatis* | quercetin | P61073 | CXCR4 |
| **3629** | *Herba Gynostemmatis* | quercetin | O75310 | UGT2B11 |
| **3630** | *Herba Gynostemmatis* | quercetin | Q02880 | TOP2B |
| **3631** | *Herba Gynostemmatis* | quercetin | Q13630 | TSTA3 |
| **3632** | *Herba Gynostemmatis* | quercetin | P07203 | GPX1 |
| **3633** | *Herba Gynostemmatis* | quercetin | P23560 | BDNF |
| **3634** | *Herba Gynostemmatis* | quercetin | P11166 | SLC2A1 |
| **3635** | *Herba Gynostemmatis* | quercetin | P35503 | UGT1A3 |
| **3636** | *Herba Gynostemmatis* | quercetin | P40313 | CTRL |
| **3637** | *Herba Gynostemmatis* | rutin | P35228 | NOS2 |
| **3638** | *Herba Gynostemmatis* | rutin | P09917 | ALOX5 |
| **3639** | *Herba Gynostemmatis* | rutin | P01375 | TNF |
| **3640** | *Herba Gynostemmatis* | rutin | P21731 | TBXA2R |
| **3641** | *Herba Gynostemmatis* | rutin | P05231 | IL6 |
| **3642** | *Herba Gynostemmatis* | rutin | P01584 | IL1B |
| **3643** | *Herba Gynostemmatis* | rutin | P00441 | SOD1 |
| **3644** | *Herba Gynostemmatis* | rutin | P09211 | GSTP1 |
| **3645** | *Herba Gynostemmatis* | rutin | P04040 | CAT |
| **3646** | *Herba Gynostemmatis* | rutin | P16435 | POR |
| **3647** | *Herba Gynostemmatis* | rutin | P04035 | HMGCR |
| **3648** | *Herba Gynostemmatis* | rutin | P42574 | CASP3 |
| **3649** | *Herba Gynostemmatis* | rutin | P10145 | CXCL8 |
| **3650** | *Herba Gynostemmatis* | rutin | P28482 | MAPK1 |
| **3651** | *Herba Gynostemmatis* | rutin | P00390 | GSR |
| **3652** | *Herba Gynostemmatis* | rutin | P13500 | CCL2 |
| **3653** | *Herba Gynostemmatis* | rutin | P35869 | AHR |
| **3654** | *Herba Gynostemmatis* | rutin | P05091 | ALDH2 |
| **3655** | *Herba Gynostemmatis* | rutin | P27361 | MAPK3 |
| **3656** | *Herba Gynostemmatis* | rutin | Q9UBK8 | MTRR |
| **3657** | *Herba Gynostemmatis* | rutin | P00533 | EGFR |
| **3658** | *Herba Gynostemmatis* | rutin | P08913 | ADRA2A |
| **3659** | *Herba Gynostemmatis* | rutin | O60235 | TMPRSS11D |
| **3660** | *Herba Gynostemmatis* | rutin | P29474 | NOS3 |
| **3661** | *Herba Gynostemmatis* | rutin | P34932 | HSPA4 |
| **3662** | *Herba Gynostemmatis* | rutin | P22303 | ACHE |
| **3663** | *Herba Gynostemmatis* | rutin | P02778 | CXCL10 |
| **3664** | *Herba Gynostemmatis* | rutin | P07237 | P4HB |
| **3665** | *Herba Gynostemmatis* | rutin | P36956 | SREBF1 |
| **3666** | *Herba Gynostemmatis* | rutin | P27540 | ARNT |
| **3667** | *Herba Gynostemmatis* | rutin | P55210 | CASP7 |
| **3668** | *Herba Gynostemmatis* | rutin | P42330 | AKR1C3 |
| **3669** | *Herba Gynostemmatis* | rutin | P18825 | ADRA2C |
| **3670** | *Herba Gynostemmatis* | sucrose | P15121 | AKR1B1 |
| **3671** | *Herba Gynostemmatis* | sucrose | P05177 | CYP1A2 |
| **3672** | *Herba Gynostemmatis* | sucrose | P15104 | GLUL |
| **3673** | *Herba Gynostemmatis* | sucrose | Q14994 | NR1I3 |
| **3674** | *Herba Gynostemmatis* | sucrose | P37231 | PPARG |
| **3675** | *Herba Gynostemmatis* | sucrose | P01100 | FOS |
| **3676** | *Herba Gynostemmatis* | sucrose | P01730 | CD4 |
| **3677** | *Herba Gynostemmatis* | sucrose | P09601 | HMOX1 |
| **3678** | *Herba Gynostemmatis* | sucrose | P30519 | HMOX2 |
| **3679** | *Herba Gynostemmatis* | sucrose | P00390 | GSR |
| **3680** | *Herba Gynostemmatis* | sucrose | P04040 | CAT |
| **3681** | *Herba Gynostemmatis* | sucrose | P05181 | CYP2E1 |
| **3682** | *Herba Gynostemmatis* | sucrose | P06276 | BCHE |
| **3683** | *Herba Gynostemmatis* | sucrose | P31040 | SDHA |
| **3684** | *Herba Gynostemmatis* | sucrose | P01133 | EGF |
| **3685** | *Herba Gynostemmatis* | sucrose | Q01959 | SLC6A3 |
| **3686** | *Herba Gynostemmatis* | sucrose | P62979 | RPS27A |
| **3687** | *Herba Gynostemmatis* | sucrose | P02768 | ALB |
| **3688** | *Herba Gynostemmatis* | sucrose | Q99527 | GPER1 |
| **3689** | *Herba Gynostemmatis* | sucrose | P29474 | NOS3 |
| **3690** | *Herba Gynostemmatis* | sucrose | P0CG47 | UBB |
| **3691** | *Herba Gynostemmatis* | sucrose | P35568 | IRS1 |
| **3692** | *Herba Gynostemmatis* | sucrose | P99999 | CYCS |
| **3693** | *Herba Gynostemmatis* | sucrose | P35228 | NOS2 |
| **3694** | *Herba Gynostemmatis* | sucrose | P30536 | TSPO |
| **3695** | *Herba Gynostemmatis* | sucrose | P20382 | PMCH |
| **3696** | *Herba Gynostemmatis* | sucrose | P0CG48 | UBC |
| **3697** | *Herba Gynostemmatis* | sucrose | Q42521 | GAD1 |
| **3698** | *Herba Gynostemmatis* | sucrose | P08195 | SLC3A2 |
| **3699** | *Herba Gynostemmatis* | sucrose | P01106 | MYC |
| **3700** | *Herba Gynostemmatis* | sucrose | P07101 | TH |
| **3701** | *Herba Gynostemmatis* | sucrose | P62987 | UBA52 |
| **3702** | *Herba Gynostemmatis* | sucrose | P35372 | OPRM1 |
| **3703** | *Herba Gynostemmatis* | sucrose | P28290 | CS |
| **3704** | *Herba Gynostemmatis* | sucrose | Q16539 | MAPK14 |
| **3705** | *Herba Gynostemmatis* | sucrose | P04798 | CYP1A1 |
| **3706** | *Herba Gynostemmatis* | sucrose | P01019 | AGT |
| **3707** | *Herba Gynostemmatis* | sucrose | P48357 | LEPR |
| **3708** | *Herba Gynostemmatis* | sucrose | P53396 | ACLY |
| **3709** | *Herba Gynostemmatis* | sucrose | Q07869 | PPARA |
| **3710** | *Herba Gynostemmatis* | sucrose | P28482 | MAPK1 |
| **3711** | *Herba Gynostemmatis* | sucrose | P27361 | MAPK3 |
| **3712** | *Herba Gynostemmatis* | sucrose | P14672 | SLC2A4 |
| **3713** | *Herba Gynostemmatis* | sucrose | P00441 | SOD1 |
| **3714** | *Herba Gynostemmatis* | sucrose | P36956 | SREBF1 |
| **3715** | *Herba Gynostemmatis* | sucrose | P42574 | CASP3 |
| **3716** | *Herba Gynostemmatis* | sucrose | Q8WTV0 | SCARB1 |

**Supplementary Table S3. Putative targets of JZG**

| **NO** | **Uniprot ID** | **Target Protein Name** | **Target Gene Name** |
| --- | --- | --- | --- |
| **1** | O95477 | ATP-binding cassette sub-family A member 1 | ABCA1 |
| **2** | P08183 | Multidrug resistance protein 1 | ABCB1 |
| **3** | P33527 | Multidrug resistance-associated protein 1 | ABCC1 |
| **4** | Q9UNQ0 | ATP-binding cassette sub-family G member 2 | ABCG2 |
| **5** | P22303 | Acetylcholinesterase | ACHE |
| **6** | P53396 | ATP-citrate synthase | ACLY |
| **7** | P21399 | Cytoplasmic aconitate hydratase | ACO1 |
| **8** | Q15848 | Adiponectin | ADIPOQ |
| **9** | P08913 | Alpha-2A adrenergic receptor | ADRA2A |
| **10** | P18825 | Alpha-2C adrenergic receptor | ADRA2C |
| **11** | P01019 | Angiotensinogen | AGT |
| **12** | P35869 | Aryl hydrocarbon receptor | AHR |
| **13** | Q12904 | Aminoacyl tRNA synthase complex-interacting multifunctional protein 1 | AIMP1 |
| **14** | Q13155 | Aminoacyl tRNA synthase complex-interacting multifunctional protein 2 | AIMP2 |
| **15** | P15121 | Aldose reductase | AKR1B1 |
| **16** | O60218 | Aldo-keto reductase family 1 member B10 | AKR1B10 |
| **17** | P42330 | Aldo-keto reductase family 1 member C3 | AKR1C3 |
| **18** | P31749 | RAC-alpha serine/threonine-protein kinase | AKT1 |
| **19** | P02768 | Serum albumin | ALB |
| **20** | Q8IZ83 | Aldehyde dehydrogenase family 16 member A1 | ALDH16A1 |
| **21** | P00352 | Retinal dehydrogenase 1 | ALDH1A1 |
| **22** | O94788 | Retinal dehydrogenase 2 | ALDH1A2 |
| **23** | P47895 | Aldehyde dehydrogenase family 1 member A3 | ALDH1A3 |
| **24** | P30837 | Aldehyde dehydrogenase X, mitochondria | ALDH1B1 |
| **25** | O75891 | Cytosolic 10-formyltetrahydrofolate dehydrogenase | ALDH1L1 |
| **26** | Q3SY69 | Mitochondrial 10-formyltetrahydrofolate dehydrogenase | ALDH1L2 |
| **27** | P05091 | Aldehyde dehydrogenase, mitochondrial | ALDH2 |
| **28** | P30838 | Aldehyde dehydrogenase, dimeric NADP-preferring | ALDH3A1 |
| **29** | P51648 | Fatty aldehyde dehydrogenase | ALDH3A2 |
| **30** | P48448 | Aldehyde dehydrogenase family 3 member B2 | ALDH3B2 |
| **31** | P30038 | Delta-1-pyrroline-5-carboxylate dehydrogenase, mitochondrial | ALDH4A1 |
| **32** | P51649 | Succinate-semialdehyde dehydrogenase, mitochondrial | ALDH5A1 |
| **33** | P49419 | Alpha-aminoadipic semialdehyde dehydrogenase | ALDH7A1 |
| **34** | Q9H2A2 | Aldehyde dehydrogenase family 8 member A1 | ALDH8A1 |
| **35** | P49189 | 4-trimethylaminobutyraldehyde dehydrogenase | ALDH9A1 |
| **36** | Q96BT7 | Alkylated DNA repair protein alkB homolog 8 | ALKBH8 |
| **37** | P18054 | Arachidonate 12-lipoxygenase, 12S-type | ALOX12 |
| **38** | P09917 | Arachidonate 5-lipoxygenase | ALOX5 |
| **39** | P04083 | Annexin A1 | ANXA1 |
| **40** | P04114 | Apolipoprotein B-100 | APOB |
| **41** | P02649 | Apolipoprotein E | APOE |
| **42** | P05067 | Amyloid-beta A4 protein | APP |
| **43** | P10275 | Androgen receptor | AR |
| **44** | P27540 | Aryl hydrocarbon receptor nuclear translocator | ARNT |
| **45** | Q13315 | Serine-protein kinase ATM | ATM |
| **46** | P01185 | Vasopressin-neurophysin 2-copeptin | AVP |
| **47** | P61769 | Beta-2-microglobulin | B2M |
| **48** | Q07812 | Apoptosis regulator BAX | BAX |
| **49** | P54687 | Branched-chain-amino-acid aminotransferase, cytosolic | BCAT1 |
| **50** | O15382 | Branched-chain-amino-acid aminotransferase, mitochondrial | BCAT2 |
| **51** | P06276 | Cholinesterase | BCHE |
| **52** | P10415 | Apoptosis regulator Bcl-2 | BCL2 |
| **53** | Q16548 | Bcl-2-related protein A1 | BCL2A1 |
| **54** | Q07817 | Bcl-2-like protein 1 | BCL2L1 |
| **55** | Q9BZR8 | Apoptosis facilitator Bcl-2-like protein 14 | BCL2L14 |
| **56** | P46663 | B1 bradykinin receptor | BDKRB1 |
| **57** | P23560 | Brain-derived neurotrophic factor | BDNF |
| **58** | Q13867 | Bleomycin hydrolase | BLMH |
| **59** | Q9UMX3 | Bcl-2-related ovarian killer protein | BOK |
| **60** | P01024 | Complement C3 | C3 |
| **61** | P01031 | Complement C5 | C5 |
| **62** | P21730 | C5a anaphylatoxin chemotactic receptor 1 | C5AR1 |
| **63** | P27797 | Calreticulin | CALR |
| **64** | P42574 | Caspase-3 | CASP3 |
| **65** | P55210 | Caspase-7 | CASP7 |
| **66** | Q14790 | Caspase-8 | CASP8 |
| **67** | P55211 | Caspase-9 | CASP9 |
| **68** | P04040 | Catalase | CAT |
| **69** | Q8N4T8 | Carbonyl reductase family member 4 | CBR4 |
| **70** | P13500 | C-C motif chemokine 2 | CCL2 |
| **71** | P24385 | G1/S-specific cyclin-D1 | CCND1 |
| **72** | P01730 | T-cell surface glycoprotein CD4 | CD4 |
| **73** | P06493 | Cyclin-dependent kinase 1 | CDK1 |
| **74** | P24941 | Cyclin-dependent kinase 2 | CDK2 |
| **75** | P38936 | Cyclin-dependent kinase inhibitor 1 | CDKN1A |
| **76** | Q9UNI1 | Chymotrypsin-like elastase family member 1 | CELA1 |
| **77** | P13569 | Cystic fibrosis transmembrane conductance regulator | CFTR |
| **78** | O15111 | Inhibitor of nuclear factor kappa-B kinase subunit alpha | CHUK |
| **79** | P23946 | Chymase | CMA1 |
| **80** | Q96KP4 | Cytosolic non-specific dipeptidase | CNDP2 |
| **81** | P28290 | Sperm-specific antigen 2 | CS |
| **82** | P04141 | Granulocyte-macrophage colony-stimulating factor | CSF2 |
| **83** | P35222 | Catenin beta-1 | CTNNB1 |
| **84** | P40313 | Chymotrypsin-like protease CTRL-1 | CTRL |
| **85** | P25774 | Cathepsin S | CTSS |
| **86** | P09341 | Growth-regulated alpha protein | CXCL1 |
| **87** | P02778 | C-X-C motif chemokine 10 | CXCL10 |
| **88** | P10145 | Interleukin-8 | CXCL8 |
| **89** | P61073 | C-X-C chemokine receptor type 4 | CXCR4 |
| **90** | P99999 | Cytochrome c | CYCS |
| **91** | P04798 | Cytochrome P450 1A1 | CYP1A1 |
| **92** | P05177 | Cytochrome P450 1A2 | CYP1A2 |
| **93** | P33261 | Cytochrome P450 2C19 | CYP2C19 |
| **94** | P11712 | Cytochrome P450 2C9 | CYP2C9 |
| **95** | P10635 | Cytochrome P450 2D6 | CYP2D6 |
| **96** | P05181 | Cytochrome P450 2E1 | CYP2E1 |
| **97** | P08684 | Cytochrome P450 3A4 | CYP3A4 |
| **98** | P78329 | Phylloquinone omega-hydroxylase CYP4F2 | CYP4F2 |
| **99** | P14868 | Aspartate--tRNA ligase, cytoplasmic | DARS |
| **100** | Q16698 | 2,4-dienoyl-CoA reductase, mitochondrial | DECR1 |
| **101** | P09622 | Dihydrolipoyl dehydrogenase, mitochondrial | DLD |
| **102** | P21917 | D(4) dopamine receptor | DRD4 |
| **103** | P05305 | Endothelin-1 | EDN1 |
| **104** | O43324 | Eukaryotic translation elongation factor 1 epsilon-1 | EEF1E1 |
| **105** | P01133 | Pro-epidermal growth factor | EGF |
| **106** | P00533 | Epidermal growth factor receptor | EGFR |
| **107** | Q7L5Y1 | Mitochondrial enolase superfamily member 1 | ENOSF1 |
| **108** | Q07075 | Glutamyl aminopeptidase | ENPEP |
| **109** | P07814 | Bifunctional glutamate/proline--tRNA ligase | EPRS |
| **110** | Q9NZ08 | Endoplasmic reticulum aminopeptidase 1 | ERAP1 |
| **111** | Q6P179 | Endoplasmic reticulum aminopeptidase 2 | ERAP2 |
| **112** | P04626 | Receptor tyrosine-protein kinase erbB-2 | ERBB2 |
| **113** | P03372 | Estrogen receptor | ESR1 |
| **114** | Q92731 | Estrogen receptor beta | ESR2 |
| **115** | P68106 | Peptidyl-prolyl cis-trans isomerase FKBP1B | FKBP1B |
| **116** | P02751 | Fibronectin | FN1 |
| **117** | P01100 | Proto-oncogene c-Fos | FOS |
| **118** | P25090 | N-formyl peptide receptor 2 | FPR2 |
| **119** | Q42521 | Glutamate decarboxylase 1 | GAD1 |
| **120** | P01275 | Glucagon | GCG |
| **121** | O75223 | Gamma-glutamylcyclotransferase | GGCT |
| **122** | P15104 | Glutamine synthetase | GLUL |
| **123** | P63096 | Guanine nucleotide-binding protein G(i) subunit alpha-1 | GNAI1 |
| **124** | P08754 | Guanine nucleotide-binding protein G(k) subunit alpha | GNAI3 |
| **125** | P59768 | Guanine nucleotide-binding protein G(I)/G(S)/G(O) subunit gamma-2 | GNG2 |
| **126** | P17174 | Aspartate aminotransferase, cytoplasmic | GOT1 |
| **127** | P00505 | Aspartate aminotransferase, mitochondrial | GOT2 |
| **128** | Q99527 | G-protein coupled estrogen receptor 1 | GPER1 |
| **129** | Q13304 | Uracil nucleotide/cysteinyl leukotriene receptor | GPR17 |
| **130** | P07203 | Glutathione peroxidase 1 | GPX1 |
| **131** | P49841 | Glycogen synthase kinase-3 beta | GSK3B |
| **132** | P00390 | Glutathione reductase, mitochondrial | GSR |
| **133** | P09211 | Glutathione S-transferase P | GSTP1 |
| **134** | Q16665 | Hypoxia-inducible factor 1-alpha | HIF1A |
| **135** | P30443 | HLA class I histocompatibility antigen, A-1 alpha chain | HLA-A |
| **136** | P01889 | HLA class I histocompatibility antigen, B-7 alpha chain | HLA-B |
| **137** | P30499 | HLA class I histocompatibility antigen, Cw-1 alpha chain | HLA-C |
| **138** | P17693 | HLA class I histocompatibility antigen, alpha chain G | HLA-G |
| **139** | P04035 | 3-hydroxy-3-methylglutaryl-coenzyme A reductase | HMGCR |
| **140** | P09601 | Heme oxygenase 1 | HMOX1 |
| **141** | P30519 | Heme oxygenase 2 | HMOX2 |
| **142** | O60760 | Hematopoietic prostaglandin D synthase | HPGDS |
| **143** | P00739 | Haptoglobin-related protein | HPR |
| **144** | P14060 | 3 beta-hydroxysteroid dehydrogenase/Delta 5-->4-isomerase type 1 | HSD3B1 |
| **145** | P26439 | 3 beta-hydroxysteroid dehydrogenase/Delta 5-->4-isomerase type 2 | HSD3B2 |
| **146** | Q9H2F3 | 3 beta-hydroxysteroid dehydrogenase type 7 | HSD3B7 |
| **147** | P34932 | Heat shock 70 kDa protein 4 | HSPA4 |
| **148** | P41252 | Isoleucine--tRNA ligase, cytoplasmic | IARS |
| **149** | P05362 | Intercellular adhesion molecule 1 | ICAM1 |
| **150** | Q5TF58 | Intermediate filament family orphan 2 | IFFO2 |
| **151** | P08069 | Insulin-like growth factor 1 receptor | IGF1R |
| **152** | P01584 | Interleukin-1 beta | IL1B |
| **153** | Q96RQ9 | L-amino-acid oxidase | IL4I1 |
| **154** | P05231 | Interleukin-6 | IL6 |
| **155** | P35568 | Insulin receptor substrate 1 | IRS1 |
| **156** | O60674 | Tyrosine-protein kinase JAK2 | JAK2 |
| **157** | P05412 | Transcription factor AP-1 | JUN |
| **158** | Q15046 | Lysine--tRNA ligase | KARS |
| **159** | P01042 | Kininogen-1 | KNG1 |
| **160** | P01116 | GTPase KRas | KRAS |
| **161** | Q9P2J5 | Leucine--tRNA ligase, cytoplasmic | LARS |
| **162** | P48357 | Leptin receptor | LEPR |
| **163** | Q9UIQ6 | Leucyl-cystinyl aminopeptidase | LNPEP |
| **164** | Q92633 | Lysophosphatidic acid receptor 1 | LPAR1 |
| **165** | Q9HBW0 | Lysophosphatidic acid receptor 2 | LPAR2 |
| **166** | Q9UBY5 | Lysophosphatidic acid receptor 3 | LPAR3 |
| **167** | Q9H1C0 | Lysophosphatidic acid receptor 5 | LPAR5 |
| **168** | P21397 | Amine oxidase [flavin-containing] A | MAOA |
| **169** | P27338 | Amine oxidase [flavin-containing] B | MAOB |
| **170** | P28482 | Mitogen-activated protein kinase 1 | MAPK1 |
| **171** | Q16539 | Mitogen-activated protein kinase 14 | MAPK14 |
| **172** | P27361 | Mitogen-activated protein kinase 3 | MAPK3 |
| **173** | P45983 | Mitogen-activated protein kinase 8 | MAPK8 |
| **174** | P56192 | Methionine--tRNA ligase, cytoplasmic | MARS |
| **175** | Q99705 | Melanin-concentrating hormone receptor 1 | MCHR1 |
| **176** | Q969V1 | Melanin-concentrating hormone receptor 2 | MCHR2 |
| **177** | Q07820 | Induced myeloid leukemia cell differentiation protein Mcl-1 | MCL1 |
| **178** | P03956 | Interstitial collagenase | MMP1 |
| **179** | P08253 | 72 kDa type IV collagenase | MMP2 |
| **180** | P14780 | Matrix metalloproteinase-9 | MMP9 |
| **181** | P05164 | Myeloperoxidase | MPO |
| **182** | P48039 | Melatonin receptor type 1A | MTNR1A |
| **183** | P49286 | Melatonin receptor type 1B | MTNR1B |
| **184** | P42345 | Serine/threonine-protein kinase mTOR | MTOR |
| **185** | Q9UBK8 | Methionine synthase reductase | MTRR |
| **186** | P01106 | Myc proto-oncogene protein | MYC |
| **187** | P43490 | Nicotinamide phosphoribosyltransferase | NAMPT |
| **188** | Q16236 | Nuclear factor erythroid 2-related factor 2 | NFE2L2 |
| **189** | P25963 | NF-kappa-B inhibitor alpha | NFKBIA |
| **190** | Q5H8A3 | Neuromedin-S | NMS |
| **191** | P48645 | Neuromedin-U | NMU |
| **192** | Q9HB89 | Neuromedin-U receptor 1 | NMUR1 |
| **193** | Q9GZQ4 | Neuromedin-U receptor 2 | NMUR2 |
| **194** | P35228 | Nitric oxide synthase, inducible | NOS2 |
| **195** | P29474 | Nitric oxide synthase, endothelial | NOS3 |
| **196** | Q9UHC9 | Niemann-Pick C1-like protein 1 | NPC1L1 |
| **197** | P55786 | Puromycin-sensitive aminopeptidase | NPEPPS |
| **198** | P25929 | Neuropeptide Y receptor type 1 | NPY1R |
| **199** | P15559 | NAD(P)H dehydrogenase [quinone] 1 | NQO1 |
| **200** | O75469 | Nuclear receptor subfamily 1 group I member 2 | NR1I2 |
| **201** | Q14994 | Nuclear receptor subfamily 1 group I member 3 | NR1I3 |
| **202** | P21589 | 5'-nucleotidase | NT5E |
| **203** | P35372 | Mu-type opioid receptor | OPRM1 |
| **204** | P07237 | Protein disulfide-isomerase | P4HB |
| **205** | P09874 | Poly [ADP-ribose] polymerase 1 | PARP1 |
| **206** | P35558 | Phosphoenolpyruvate carboxykinase, cytosolic [GTP] | PCK1 |
| **207** | P30101 | Protein disulfide-isomerase A3 | PDIA3 |
| **208** | O43175 | D-3-phosphoglycerate dehydrogenase | PHGDH |
| **209** | P42336 | Phosphatidylinositol 4,5-bisphosphate 3-kinase catalytic subunit alpha isoform | PIK3CA |
| **210** | P00750 | Tissue-type plasminogen activator | PLAT |
| **211** | P00749 | Urokinase-type plasminogen activator | PLAU |
| **212** | P20382 | Pro-MCH | PMCH |
| **213** | P01189 | Pro-opiomelanocortin | POMC |
| **214** | P27169 | Serum paraoxonase/arylesterase 1 | PON1 |
| **215** | P16435 | NADPH--cytochrome P450 reductase | POR |
| **216** | Q07869 | Peroxisome proliferator-activated receptor alpha | PPARA |
| **217** | P37231 | Peroxisome proliferator-activated receptor gamma | PPARG |
| **218** | P17252 | Protein kinase C alpha type | PRKCA |
| **219** | P07477 | Trypsin-1 | PRSS1 |
| **220** | P35030 | Trypsin-3 | PRSS3 |
| **221** | P25786 | Proteasome subunit alpha type-1 | PSMA1 |
| **222** | P25788 | Proteasome subunit alpha type-3 | PSMA3 |
| **223** | P25789 | Proteasome subunit alpha type-4 | PSMA4 |
| **224** | P28066 | Proteasome subunit alpha type-5 | PSMA5 |
| **225** | P60900 | Proteasome subunit alpha type-6 | PSMA6 |
| **226** | O14818 | Proteasome subunit alpha type-7 | PSMA7 |
| **227** | Q8TAA3 | Proteasome subunit alpha type-7-like | PSMA8 |
| **228** | P20618 | Proteasome subunit beta type-1 | PSMB1 |
| **229** | P40306 | Proteasome subunit beta type-10 | PSMB10 |
| **230** | A5LHX3 | Proteasome subunit beta type-11 | PSMB11 |
| **231** | P49721 | Proteasome subunit beta type-2 | PSMB2 |
| **232** | P28070 | Proteasome subunit beta type-4 | PSMB4 |
| **233** | P28074 | Proteasome subunit beta type-5 | PSMB5 |
| **234** | P28072 | Proteasome subunit beta type-6 | PSMB6 |
| **235** | Q99436 | Proteasome subunit beta type-7 | PSMB7 |
| **236** | P62191 | 26S proteasome regulatory subunit 4 | PSMC1 |
| **237** | P35998 | 26S proteasome regulatory subunit 7 | PSMC2 |
| **238** | P17980 | 26S proteasome regulatory subunit 6A | PSMC3 |
| **239** | P43686 | 26S proteasome regulatory subunit 6B | PSMC4 |
| **240** | P62195 | 26S proteasome regulatory subunit 8 | PSMC5 |
| **241** | P62333 | 26S proteasome regulatory subunit 10B | PSMC6 |
| **242** | Q99460 | 26S proteasome non-ATPase regulatory subunit 1 | PSMD1 |
| **243** | O75832 | 26S proteasome non-ATPase regulatory subunit 10 | PSMD10 |
| **244** | O00231 | 26S proteasome non-ATPase regulatory subunit 11 | PSMD11 |
| **245** | O00232 | 26S proteasome non-ATPase regulatory subunit 12 | PSMD12 |
| **246** | Q9UNM6 | 26S proteasome non-ATPase regulatory subunit 13 | PSMD13 |
| **247** | O00487 | 26S proteasome non-ATPase regulatory subunit 14 | PSMD14 |
| **248** | Q13200 | 26S proteasome non-ATPase regulatory subunit 2 | PSMD2 |
| **249** | O43242 | 26S proteasome non-ATPase regulatory subunit 3 | PSMD3 |
| **250** | P55036 | 26S proteasome non-ATPase regulatory subunit 4 | PSMD4 |
| **251** | Q16401 | 26S proteasome non-ATPase regulatory subunit 5 | PSMD5 |
| **252** | Q15008 | 26S proteasome non-ATPase regulatory subunit 6 | PSMD6 |
| **253** | P51665 | 26S proteasome non-ATPase regulatory subunit 7 | PSMD7 |
| **254** | P48556 | 26S proteasome non-ATPase regulatory subunit 8 | PSMD8 |
| **255** | O00233 | 26S proteasome non-ATPase regulatory subunit 9 | PSMD9 |
| **256** | Q06323 | Proteasome activator complex subunit 1 | PSME1 |
| **257** | Q9UL46 | Proteasome activator complex subunit 2 | PSME2 |
| **258** | P61289 | Proteasome activator complex subunit 3 | PSME3 |
| **259** | Q14997 | Proteasome activator complex subunit 4 | PSME4 |
| **260** | Q92530 | Proteasome inhibitor PI31 subunit | PSMF1 |
| **261** | P60484 | Phosphatidylinositol 3,4,5-trisphosphate 3-phosphatase and dual-specificity protein phosphatase PTEN | PTEN |
| **262** | P23219 | Prostaglandin G/H synthase 1 | PTGS1 |
| **263** | Q05397 | Focal adhesion kinase 1 | PTK2 |
| **264** | P15151 | Poliovirus receptor | PVR |
| **265** | P47897 | Glutamine--tRNA ligase | QARS |
| **266** | P54136 | Arginine--tRNA ligase, cytoplasmic | RARS |
| **267** | P06400 | Retinoblastoma-associated protein | RB1 |
| **268** | Q04206 | Transcription factor p65 | RELA |
| **269** | Q08116 | Regulator of G-protein signaling 1 | RGS1 |
| **270** | O43665 | Regulator of G-protein signaling 10 | RGS10 |
| **271** | O43566 | Regulator of G-protein signaling 14 | RGS14 |
| **272** | Q9NS28 | Regulator of G-protein signaling 18 | RGS18 |
| **273** | P49795 | Regulator of G-protein signaling 19 | RGS19 |
| **274** | O76081 | Regulator of G-protein signaling 20 | RGS20 |
| **275** | P49798 | Regulator of G-protein signaling 4 | RGS4 |
| **276** | P49758 | Regulator of G-protein signaling 6 | RGS6 |
| **277** | P49802 | Regulator of G-protein signaling 7 | RGS7 |
| **278** | P62979 | Ubiquitin-40S ribosomal protein S27a | RPS27A |
| **279** | P0DJI8 | Serum amyloid A-1 protein | SAA1 |
| **280** | Q8WTV0 | Scavenger receptor class B member 1 | SCARB1 |
| **281** | P31040 | Succinate dehydrogenase [ubiquinone] flavoprotein subunit, mitochondrial | SDHA |
| **282** | P21912 | Succinate dehydrogenase [ubiquinone] iron-sulfur subunit, mitochondrial | SDHB |
| **283** | P61619 | Protein transport protein Sec61 subunit alpha isoform 1 | SEC61A1 |
| **284** | Q9H9S3 | Protein transport protein Sec61 subunit alpha isoform 2 | SEC61A2 |
| **285** | P60468 | Protein transport protein Sec61 subunit beta | SEC61B |
| **286** | P60059 | Protein transport protein Sec61 subunit gamma | SEC61G |
| **287** | P05546 | Heparin cofactor 2 | SERPIND1 |
| **288** | P05121 | Plasminogen activator inhibitor 1 | SERPINE1 |
| **289** | P34896 | Serine hydroxymethyltransferase, cytosolic | SHMT1 |
| **290** | Q96EB6 | NAD-dependent protein deacetylase sirtuin-1 | SIRT1 |
| **291** | P11166 | Solute carrier family 2, facilitated glucose transporter member 1 | SLC2A1 |
| **292** | P14672 | Solute carrier family 2, facilitated glucose transporter member 4 | SLC2A4 |
| **293** | P08195 | 4F2 cell-surface antigen heavy chain | SLC3A2 |
| **294** | Q9H2J7 | Sodium-dependent neutral amino acid transporter B(0)AT2 | SLC6A15 |
| **295** | Q96N87 | Sodium-dependent neutral amino acid transporter B(0)AT3 | SLC6A18 |
| **296** | Q01959 | Sodium-dependent dopamine transporter | SLC6A3 |
| **297** | Q01650 | Large neutral amino acids transporter small subunit 1 | SLC7A5 |
| **298** | Q9UHI5 | Large neutral amino acids transporter small subunit 2 | SLC7A8 |
| **299** | P00441 | Superoxide dismutase [Cu-Zn] | SOD1 |
| **300** | Q14534 | Squalene monooxygenase | SQLE |
| **301** | P12931 | Proto-oncogene tyrosine-protein kinase Src | SRC |
| **302** | P36956 | Sterol regulatory element-binding protein 1 | SREBF1 |
| **303** | P40763 | Signal transducer and activator of transcription 3 | STAT3 |
| **304** | P50225 | Sulfotransferase 1A1 | SULT1A1 |
| **305** | P17735 | Tyrosine aminotransferase | TAT |
| **306** | P21731 | Thromboxane A2 receptor | TBXA2R |
| **307** | P01137 | Transforming growth factor beta-1 | TGFB1 |
| **308** | P07101 | Tyrosine 3-monooxygenase | TH |
| **309** | O60603 | Toll-like receptor 2 | TLR2 |
| **310** | O00206 | Toll-like receptor 4 | TLR4 |
| **311** | O60235 | Transmembrane protease serine 11D | TMPRSS11D |
| **312** | P01375 | Tumor necrosis factor | TNF |
| **313** | O14763 | Tumor necrosis factor receptor superfamily member 10B | TNFRSF10B |
| **314** | P11387 | DNA topoisomerase 1 | TOP1 |
| **315** | P11388 | DNA topoisomerase 2-alpha | TOP2A |
| **316** | Q02880 | DNA topoisomerase 2-beta | TOP2B |
| **317** | P04637 | Cellular tumor antigen p53 | TP53 |
| **318** | P29144 | Tripeptidyl-peptidase 2 | TPP2 |
| **319** | P30536 | Translocator protein | TSPO |
| **320** | Q13630 | GDP-L-fucose synthase | TSTA3 |
| **321** | P14679 | Tyrosinase | TYR |
| **322** | P62987 | Ubiquitin-60S ribosomal protein L40 | UBA52 |
| **323** | P0CG47 | Polyubiquitin-B | UBB |
| **324** | P0CG48 | Polyubiquitin-C | UBC |
| **325** | P22309 | UDP-glucuronosyltransferase 1-1 | UGT1A1 |
| **326** | Q9HAW8 | UDP-glucuronosyltransferase 1-10 | UGT1A10 |
| **327** | P35503 | UDP-glucuronosyltransferase 1-3 | UGT1A3 |
| **328** | P19224 | UDP-glucuronosyltransferase 1-6 | UGT1A6 |
| **329** | Q9HAW7 | UDP-glucuronosyltransferase 1-7 | UGT1A7 |
| **330** | Q9HAW9 | UDP-glucuronosyltransferase 1-8 | UGT1A8 |
| **331** | O60656 | UDP-glucuronosyltransferase 1-9 | UGT1A9 |
| **332** | P36537 | UDP-glucuronosyltransferase 2B10 | UGT2B10 |
| **333** | O75310 | UDP-glucuronosyltransferase 2B11 | UGT2B11 |
| **334** | P54855 | UDP-glucuronosyltransferase 2B15 | UGT2B15 |
| **335** | O75795 | UDP-glucuronosyltransferase 2B17 | UGT2B17 |
| **336** | O43709 | Probable 18S rRNA (guanine-N(7))-methyltransferase | WBSCR22 |
| **337** | Q8N5D0 | WD and tetratricopeptide repeats protein 1 | WDTC1 |
| **338** | P47989 | Xanthine dehydrogenase/oxidase | XDH |

**Supplementary Table S4. Known FLD-related targets**

| **NO** | **Uniprot ID** | **Target Protein Name** | **Target Gene Name** |
| --- | --- | --- | --- |
| **1** | O95477 | ATP-binding cassette sub-family A member 1 | ABCA1 |
| **2** | Q92887 | Canalicular multispecific organic anion transporter 1 | ABCC2 |
| **3** | Q9H222 | ATP-binding cassette sub-family G member 5 | ABCG5 |
| **4** | Q9H221 | ATP-binding cassette sub-family G member 8 | ABCG8 |
| **5** | P11310 | Medium-chain specific acyl-CoA dehydrogenase, mitochondrial | ACADM |
| **6** | P24752 | Acetyl-CoA acetyltransferase, mitochondrial | ACAT1 |
| **7** | O75908 | Sterol O-acyltransferase 2 | ACAT2 |
| **8** | P12821 | Angiotensin-converting enzyme | ACE |
| **9** | P35611 | Alpha-adducin | ADD1 |
| **10** | Q15848 | Adiponectin | ADIPOQ |
| **11** | P08913 | Alpha-2A adrenergic receptor | ADRA2A |
| **12** | P07550 | Beta-2 adrenergic receptor | ADRB2 |
| **13** | P13945 | Beta-3 adrenergic receptor | ADRB3 |
| **14** | P01019 | Angiotensinogen | AGT |
| **15** | P30556 | Type-1 angiotensin II receptor | AGTR1 |
| **16** | P50052 | Type-2 angiotensin II receptor | AGTR2 |
| **17** | P05091 | Aldehyde dehydrogenase, mitochondrial | ALDH2 |
| **18** | Q9Y5C1 | Angiopoietin-related protein 3 | ANGPTL3 |
| **19** | P02647 | Apolipoprotein A-I | APOA1 |
| **20** | P02652 | Apolipoprotein A-II | APOA2 |
| **21** | P06727 | Apolipoprotein A-IV | APOA4 |
| **22** | Q6Q788 | Apolipoprotein A-V | APOA5 |
| **23** | P04114 | Apolipoprotein B-100 | APOB |
| **24** | P02654 | Apolipoprotein C-I | APOC1 |
| **25** | P02655 | Apolipoprotein C-II | APOC2 |
| **26** | P02656 | Apolipoprotein C-III | APOC3 |
| **27** | P05090 | Apolipoprotein D | APOD |
| **28** | P02649 | Apolipoprotein E | APOE |
| **29** | P02749 | Beta-2-glycoprotein 1 | APOH |
| **30** | P10275 | Androgen receptor | AR |
| **31** | P18850 | Cyclic AMP-dependent transcription factor ATF-6 alpha | ATF6 |
| **32** | Q53HC0 | Coiled-coil domain-containing protein 92 | CCDC92 |
| **33** | Q9HCU4 | Cadherin EGF LAG seven-pass G-type receptor 2 | CELSR2 |
| **34** | P11597 | Cholesteryl ester transfer protein | CETP |
| **35** | P50416 | Carnitine O-palmitoyltransferase 1, liver isoform | CPT1A |
| **36** | P13498 | Cytochrome b-245 light chain | CYBA |
| **37** | P33261 | Cytochrome P450 2C19 | CYP2C19 |
| **38** | P11712 | Cytochrome P450 2C9 | CYP2C9 |
| **39** | P05181 | Cytochrome P450 2E1 | CYP2E1 |
| **40** | P08684 | Cytochrome P450 3A4 | CYP3A4 |
| **41** | P22680 | Cholesterol 7-alpha-monooxygenase | CYP7A1 |
| **42** | Q8IVF4 | Dynein heavy chain 10, axonemal | DNAH10 |
| **43** | P24855 | Deoxyribonuclease-1 | DNASE1 |
| **44** | P18146 | Early growth response protein 1 | EGR1 |
| **45** | P22413 | Ectonucleotide pyrophosphatase/phosphodiesterase family member 1 | ENPP1 |
| **46** | P03372 | Estrogen receptor | ESR1 |
| **47** | P13804 | Electron transfer flavoprotein subunit alpha, mitochondrial | ETFA |
| **48** | P38117 | Electron transfer flavoprotein subunit beta | ETFB |
| **49** | Q16134 | Electron transfer flavoprotein-ubiquinone oxidoreductase, mitochondrial | ETFDH |
| **50** | P00488 | Coagulation factor XIII A chain | F13A1 |
| **51** | P00734 | Prothrombin | F2 |
| **52** | P12259 | Coagulation factor V | F5 |
| **53** | P08709 | Coagulation factor VII | F7 |
| **54** | P00451 | Coagulation factor VIII | F8 |
| **55** | P12104 | Fatty acid-binding protein, intestinal | FABP2 |
| **56** | O60427 | Fatty acid desaturase 1 | FADS1 |
| **57** | O95864 | Fatty acid desaturase 2 | FADS2 |
| **58** | Q9Y5Q0 | Fatty acid desaturase 3 | FADS3 |
| **59** | P12318 | Low affinity immunoglobulin gamma Fc region receptor II-a | FCGR2A |
| **60** | P02675 | Fibrinogen beta chain | FGB |
| **61** | Q99958 | Forkhead box protein C2 | FOXC2 |
| **62** | Q16445 | Gamma-aminobutyric acid receptor subunit alpha-6 | GABRA6 |
| **63** | P22466 | Galanin peptides | GAL |
| **64** | Q10471 | Polypeptide N-acetylgalactosaminyltransferase 2 | GALNT2 |
| **65** | Q14397 | Glucokinase regulatory protein | GCKR |
| **66** | P36959 | GMP reductase 1 | GMPR |
| **67** | P16520 | Guanine nucleotide-binding protein G(I)/G(S)/G(T) subunit beta-3 | GNB3 |
| **68** | Q3T906 | N-acetylglucosamine-1-phosphotransferase subunits alpha/beta | GNPTAB |
| **69** | P09488 | Glutathione S-transferase Mu 1 | GSTM1 |
| **70** | P09211 | Glutathione S-transferase P | GSTP1 |
| **71** | P30711 | Glutathione S-transferase theta-1 | GSTT1 |
| **72** | P40939 | Trifunctional enzyme subunit alpha, mitochondrial | HADHA |
| **73** | P69905 | Hemoglobin subunit alpha | HBA1 |
| **74** | P68871 | Hemoglobin subunit beta | HBB |
| **75** | P28067 | HLA class II histocompatibility antigen, DM alpha chain | HLA-DMA |
| **76** | P28068 | HLA class II histocompatibility antigen, DM beta chain | HLA-DMB |
| **77** | P01909 | HLA class II histocompatibility antigen, DQ alpha 1 chain | HLA-DQA1 |
| **78** | P01920 | HLA class II histocompatibility antigen, DQ beta 1 chain | HLA-DQB1 |
| **79** | Q9GIY3 | HLA class II histocompatibility antigen, DRB1-14 beta chain | HLA-DRB1 |
| **80** | P04035 | 3-hydroxy-3-methylglutaryl-coenzyme A reductase | HMGCR |
| **81** | P20823 | Hepatocyte nuclear factor 1-alpha | HNF1A |
| **82** | P41235 | Hepatocyte nuclear factor 4-alpha | HNF4A |
| **83** | P00738 | Haptoglobin | HP |
| **84** | P28223 | 5-hydroxytryptamine receptor 2A | HTR2A |
| **85** | P17936 | Insulin-like growth factor-binding protein 3 | IGFBP3 |
| **86** | P22301 | Interleukin-10 | IL10 |
| **87** | P01584 | Interleukin-1 beta | IL1B |
| **88** | P05231 | Interleukin-6 | IL6 |
| **89** | P06213 | Insulin receptor | INSR |
| **90** | Q13568 | Interferon regulatory factor 5 | IRF5 |
| **91** | P05106 | Integrin beta-3 | ITGB3 |
| **92** | Q9UEF7 | Klotho | KL |
| **93** | P04180 | Phosphatidylcholine-sterol acyltransferase | LCAT |
| **94** | P01130 | Low-density lipoprotein receptor | LDLR |
| **95** | P41159 | Leptin | LEP |
| **96** | P48357 | Leptin receptor | LEPR |
| **97** | P38571 | Lysosomal acid lipase/cholesteryl ester hydrolase | LIPA |
| **98** | P11150 | Hepatic triacylglycerol lipase | LIPC |
| **99** | Q05469 | Hormone-sensitive lipase | LIPE |
| **100** | Q9Y5X9 | Endothelial lipase | LIPG |
| **101** | P02545 | Prelamin-A/C | LMNA |
| **102** | P08519 | Apolipoprotein(a) | LPA |
| **103** | P06858 | Lipoprotein lipase | LPL |
| **104** | Q07954 | Prolow-density lipoprotein receptor-related protein 1 | LRP1 |
| **105** | O75581 | Low-density lipoprotein receptor-related protein 6 | LRP6 |
| **106** | P30533 | Alpha-2-macroglobulin receptor-associated protein | LRPAP1 |
| **107** | P01374 | Lymphotoxin-alpha | LTA |
| **108** | Q9Y5Q3 | Transcription factor MafB | MAFB |
| **109** | P11226 | Mannose-binding protein C | MBL2 |
| **110** | P32245 | Melanocortin receptor 4 | MC4R |
| **111** | Q9GZU1 | Mucolipin-1 | MCOLN1 |
| **112** | P08254 | Stromelysin-1 | MMP3 |
| **113** | Q13126 | S-methyl-5'-thioadenosine phosphorylase | MTAP |
| **114** | P42898 | Methylenetetrahydrofolate reductase | MTHFR |
| **115** | P03891 | NADH-ubiquinone oxidoreductase chain 2 | MT-ND2 |
| **116** | Q99707 | Methionine synthase | MTR |
| **117** | P55157 | Microsomal triglyceride transfer protein large subunit | MTTP |
| **118** | Q03426 | Mevalonate kinase | MVK |
| **119** | O14594 | Neurocan core protein | NCAN |
| **120** | P29474 | Nitric oxide synthase, endothelial | NOS3 |
| **121** | Q9UHC9 | Niemann-Pick C1-like protein 1 | NPC1L1 |
| **122** | P01303 | Pro-neuropeptide Y | NPY |
| **123** | Q15761 | Neuropeptide Y receptor type 5 | NPY5R |
| **124** | Q13133 | Oxysterols receptor LXR-alpha | NR1H3 |
| **125** | Q96RI1 | Bile acid receptor | NR1H4 |
| **126** | P04150 | Glucocorticoid receptor | NR3C1 |
| **127** | P78380 | Oxidized low-density lipoprotein receptor 1 | OLR1 |
| **128** | P43490 | Nicotinamide phosphoribosyltransferase | PBEF1 |
| **129** | P05166 | Propionyl-CoA carboxylase beta chain, mitochondrial | PCCB |
| **130** | P04054 | Phospholipase A2 | PLA2G1B |
| **131** | P47712 | Cytosolic phospholipase A2 | PLA2G4A |
| **132** | Q9UM63 | Zinc finger protein PLAGL1 | PLAGL1 |
| **133** | O60240 | Perilipin-1 | PLIN |
| **134** | P55058 | Phospholipid transfer protein | PLTP |
| **135** | Q96AD5 | Patatin-like phospholipase domain-containing protein 2 | PNPLA2 |
| **136** | P01189 | Pro-opiomelanocortin | POMC |
| **137** | P27169 | Serum paraoxonase/arylesterase 1 | PON1 |
| **138** | Q15165 | Serum paraoxonase/arylesterase 2 | PON2 |
| **139** | Q07869 | Peroxisome proliferator-activated receptor alpha | PPARA |
| **140** | Q03181 | Peroxisome proliferator-activated receptor delta | PPARD |
| **141** | P37231 | Peroxisome proliferator-activated receptor gamma | PPARG |
| **142** | Q9UBK2 | Peroxisome proliferator-activated receptor gamma coactivator 1-alpha | PPARGC1A |
| **143** | Q16821 | Protein phosphatase 1 regulatory subunit 3A | PPP1R3A |
| **144** | Q9UGJ0 | 5'-AMP-activated protein kinase subunit gamma-2 | PRKAG2 |
| **145** | P35354 | Prostaglandin G/H synthase 2 | PTGS2 |
| **146** | P19793 | Retinoic acid receptor RXR-alpha | RXRA |
| **147** | Q12770 | Sterol regulatory element-binding protein cleavage-activating protein | SCAP |
| **148** | Q8WTV0 | Scavenger receptor class B member 1 | SCARB1 |
| **149** | P16581 | E-selectin | SELE |
| **150** | Q14242 | P-selectin glycoprotein ligand 1 | SELPLG |
| **151** | P05121 | Plasminogen activator inhibitor 1 | SERPINE1 |
| **152** | Q6PCB7 | Long-chain fatty acid transport protein 1 | SLC27A1 |
| **153** | O14543 | Suppressor of cytokine signaling 3 | SOCS3 |
| **154** | P36956 | Sterol regulatory element-binding protein 1 | SREBF1 |
| **155** | Q12772 | Sterol regulatory element-binding protein 2 | SREBF2 |
| **156** | P01375 | Tumor necrosis factor | TNF |
| **157** | Q5VTQ0 | Tetratricopeptide repeat protein 39B | TTC39B |
| **158** | P25874 | Mitochondrial brown fat uncoupling protein 1 | UCP1 |
| **159** | P55851 | Mitochondrial uncoupling protein 2 | UCP2 |
| **160** | P55916 | Mitochondrial uncoupling protein 3 | UCP3 |
| **161** | P22415 | Upstream stimulatory factor 1 | USF1 |

**Supplementary Figure S1. Full-length gels for the cropped groupings of blots**


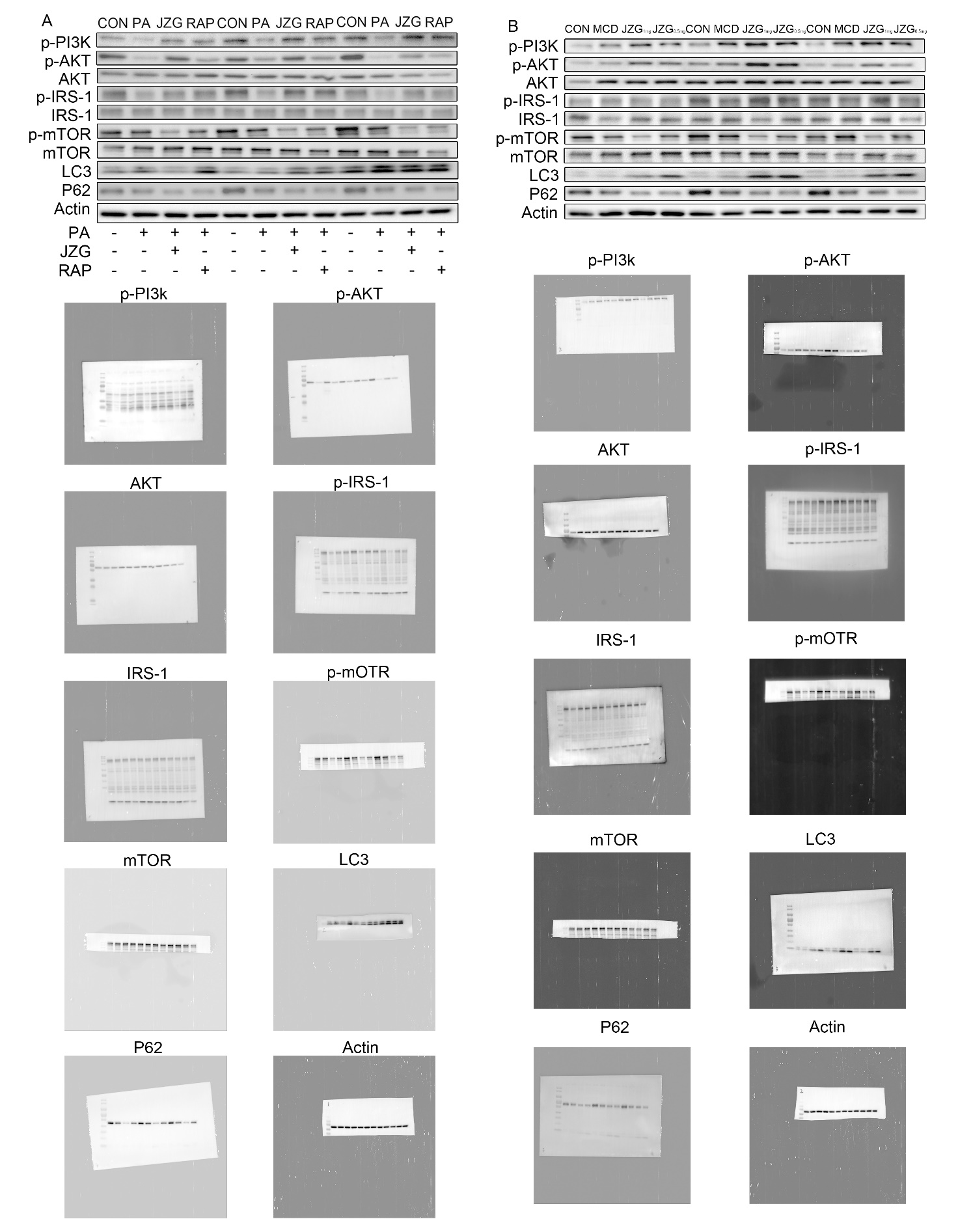


**Supplementary Figure S1. Full-length gels for the cropped groupings of blots.** A: Full-length gels for the cropped groupings of blots in figure 4A and 5A; B: Full-length gels for the cropped groupings of blots in figure 7A.
